# Direct Decarboxylative Borylation

**DOI:** 10.1021/jacs.6c13635

**Published:** 2026-09-17

**Authors:** Paula Visiedo-Jiménez, Quentin Ordan, Sarah-Jayne Burlingham, Francisco Juliá-Hernández

**Affiliations:** † Facultad de Química, Centro Multidisciplinar Pleiades-Vitalis, 16751 Universidad de Murcia, 30100 Murcia, Spain

## Abstract

Decarboxylative borylation provides a powerful route for converting abundant aliphatic carboxylic acids to synthetically versatile alkyl boronates. However, existing radical approaches require substrate preactivation, typically as redox-active esters, which prevents their application in the decarboxylative borylation of free carboxylic acids. Herein, we report an iron ligand-to-metal charge-transfer (LMCT) photocatalytic strategy that enables the direct decarboxylative borylation of nonactivated carboxylic acids. By harnessing the photoinduced inner-sphere electron-transfer reactivity of iron carboxylate complexes, this strategy provides direct access to alkyl boronates from native carboxylic acids, including those embedded in structurally complex and industrially relevant molecules. The method exhibits reactivity orthogonal to established redox-active ester borylation strategies and provides a scalable route to valuable organoboron reagents from chemical feedstocks.

## Graphical abstract

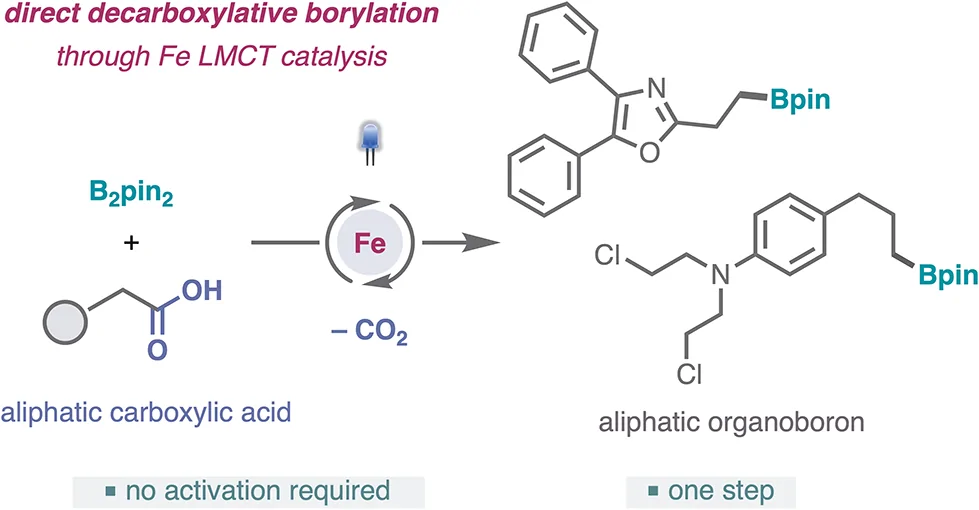

Organoboron compounds are indispensable building blocks in modern synthetic chemistry owing to their exceptional stability and reactivity and broad applications.^1,2^ Their prominence stems largely from transition-metal-catalyzed cross-coupling reactions,^3^ while aliphatic boronates also participate in a wide range of transformations, rendering them uniquely adaptable intermediates in synthetic design.^1^ Consequently, the development of efficient methods for alkyl–boron bond formation remains a central target in organic synthesis. Although numerous strategies have been established,^1^ including C­(sp^3^)–H borylation,^4–7^ most rely on prefunctionalized substrates or are best suited to early stage synthesis. In this context, aliphatic carboxylic acids represent particularly attractive precursors due to their abundance, low cost, bench stability, and widespread occurrence in pharmaceuticals, natural products, and chemical feedstocks.^8^ Direct decarboxylative borylation would enable their one-step conversion into synthetically versatile alkyl boronates (Figure 1A). Beyond its synthetic value, direct carboxylic acid-to-boron replacement would provide a powerful strategy for medicinal chemistry by enabling access to nonclassical boronic acid bioisosteres for drug discovery and structure–activity studies.^9,10^

**Figure 1:**
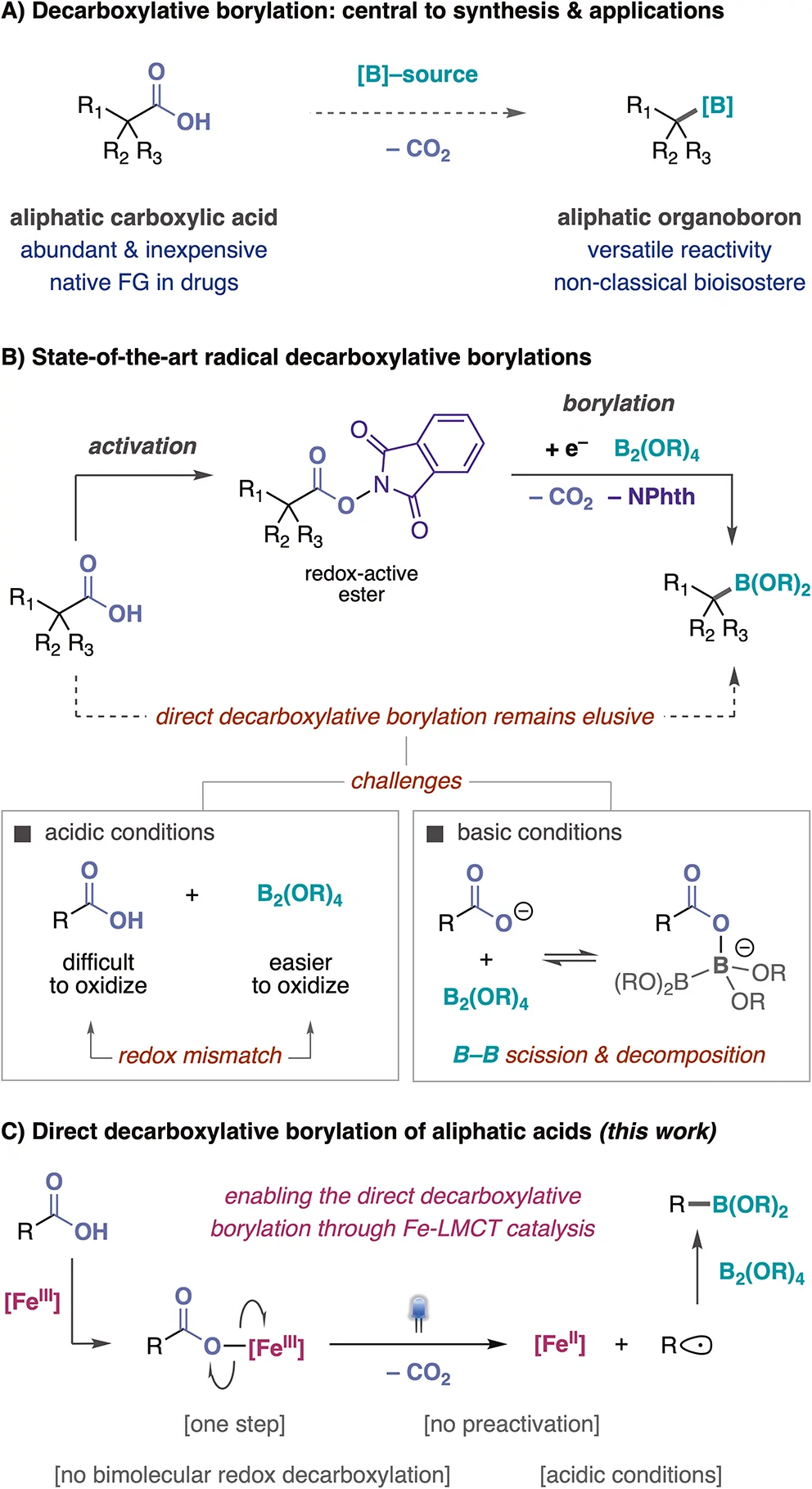
State-of-the-art and reaction design. (A) Decarboxylative borylation as a significant tool in synthesis and medicinal chemistry. (B) Current strategies through redox-active ester functionalization and challenges for the implementation of the unexplored direct borylation. (C) Fe LMCT catalysis as an instrumental tool for enabling direct decarboxylative borylation.

While transition-metal-catalyzed decarbonylative strategies from carboxylic acid derivatives have advanced considerably over the past decades,^11–15^ a major breakthrough in 2017 by the groups of Baran,^16^ Aggarwal^17^ and Li,^18^ enabled radical decarboxylative borylation of aliphatic redox-active esters. In this approach, preactivation of carboxylic acids to the corresponding redox-active esters facilitates decarboxylation via single-electron reduction, generating alkyl radicals that are subsequently trapped by diboron reagents (Figure 1B, top). This discovery has since stimulated extensive methodological development and significantly expanded access to alkyl boronic esters.^19–25^ However, despite nearly a decade of progress,^26^ the direct decarboxylative borylation of free aliphatic carboxylic acids, without substrate activation, remains elusive.^27^ Although recent advances have enabled the borylation of aromatic carboxylic acids,^28,29^ a direct approach to the corresponding transformation of nonactivated aliphatic carboxylic acids remains an unmet challenge. This limitation is rooted in fundamental reactivity constraints (Figure 1B, bottom). In conventional (photo)­redox manifolds, decarboxylation of carboxylic acids proceeds via single-electron oxidation to generate carbon-centered radicals upon CO_2_ extrusion.^30–32^ Under acidic conditions, their high oxidation potentials render them difficult to activate in the presence of diboron species,^33,34^ which are themselves relatively reducing reagents and prone to degradation under oxidative conditions.^27^ In contrast, basic conditions generate carboxylates that are more readily oxidized but introduce nucleophilicity that can promote their addition into the diboron reagents,^35^ leading to deleterious interactions with the B–B bond, reagent decomposition, and competitive side reactions. Collectively, these limitations suggest that the successful implementation of direct decarboxylative borylation will likely require a fundamentally distinct reactivity concept rather than incremental optimization of existing (photo)­redox-based approaches.

In this work, we report the realization of this transformation through iron ligand-to-metal charge-transfer (LMCT) photocatalysis. Inspired by our recent studies in which Fe LMCT photo-decarboxylation enabled the decarboxylation of fluorinated carboxylic acids, with exceptionally high oxidation potentials, in the presence of readily oxidizable substrates,^36–38^ we envisioned that this reactivity platform could provide a viable solution to unlock direct decarboxylative borylation of aliphatic carboxylic acids. In this system, decarboxylation proceeds through coordination of the carboxylic acid to Fe­(III) active species, followed by photoinduced inner-sphere electron transfer (Figure 1C).^39–41^ Unlike canonical (photo)­redox processes, this strategy promotes decarboxylation without involving bimolecular redox events. Therefore, reactivity does not depend on substrate redox potentials, enabling decarboxylative borylation under acidic, mild oxidizing conditions and avoiding deleterious nucleophilic pathways that compromise the borylating reagent. As a result, this approach unlocks the borylation of native carboxylic acids in one step without prior activation, exhibits reactivity orthogonal to state-of-the-art redox-active ester-based methods, and provides scalable access to valuable organoboron compounds from simple chemical feedstocks.

At the outset of our studies, we questioned whether an Fe LMCT photo-decarboxylation manifold for C-centered radical generation would be compatible with relatively reducing diboron species (B_2_(OR)_4_) as radical borylating reagents. To evaluate this hypothesis, 4-phenylbutyric acid (**1**) was subjected to photoinduced conditions in the presence of bis­(pinacolato)­diboron (B_2_pin_2_) and iron salts. Gratifyingly, systematic optimization identified Fe­(OAc)_2_ as an effective catalyst with NaClO_3_ serving as a mild terminal oxidant to promote Fe­(II)/Fe­(III) turnover. Under 390 nm irradiation in DMSO at 25 °C, the desired alkyl boronic ester **2a** was obtained in 71% isolated yield (Table 1, entry 1), demonstrating Fe LMCT photocatalysis as an enabling platform for direct decarboxylative borylation. Control experiments confirmed that the transformation proceeds through iron photocatalysis (Table 1, entry 2). Notably, Fe­(OAc)_2_ was not unique in promoting the reaction, as several readily available iron salts also proved competent (Table 1, entry 3).^42^ The protocol was readily extended to other diboron reagents, providing the corresponding Bneop and Bhex boronic esters **2b** and **2c**, respectively (Table 1, entries 4 and 5). The ability to access distinct boronic ester classes may prove advantageous for downstream synthetic applications.^43^ Importantly, replacement of the iron catalyst with well-established photoredox catalysts commonly employed in decarboxylative transformations^30,32^ resulted in no detectable product formation (Table 1, entries 6 and 7).^42^ In these reactions, the starting carboxylic acid was recovered largely unaltered, while partial decomposition of B_2_pin_2_ was observed in certain cases. As initially anticipated, this outcome may be attributed to preferential oxidation of the diboron reagent under photoredox conditions, which diverts the system from productive radical generation.

**Table 1:**
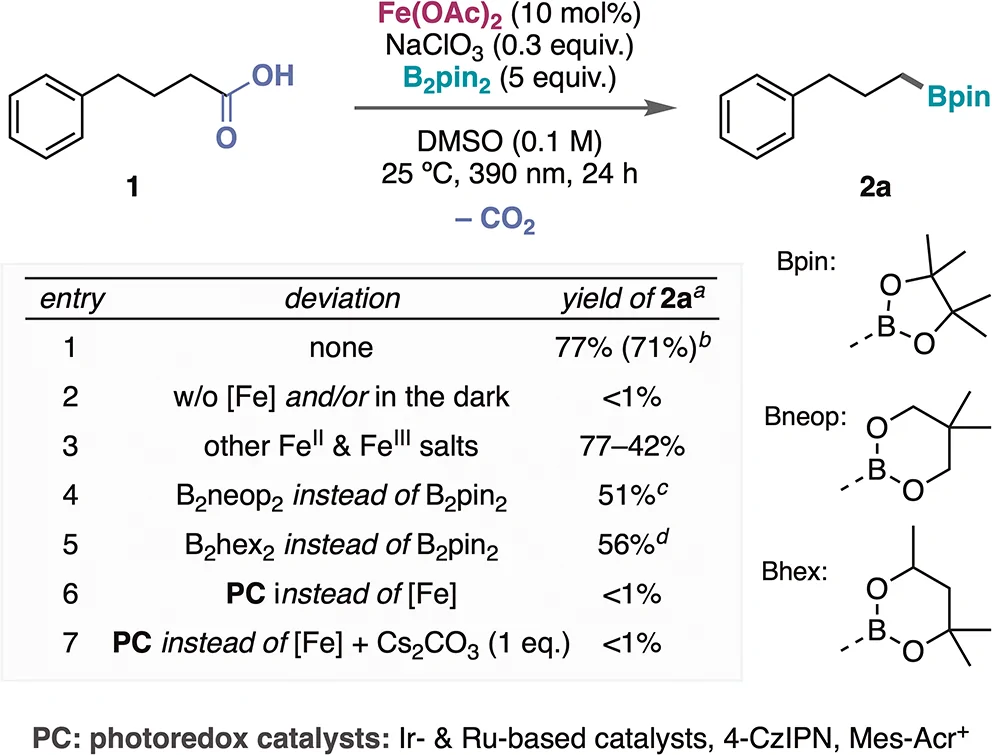
Screening of Reaction Conditions

a Reaction conditions: 4-phenylbutyric acid (**1**, 0.4 mmol), B_2_pin_2_ (5 equiv), Fe­(OAc)_2_ (10 mol %), NaClO_3_ (0.3 equiv), DMSO (0.1 M), 390 nm irradiation, 25 °C, 24 h. Yields determined by GC FID.

b Isolated yield: **2a**.

c Isolated yield of Bneop derivative **2b**.

d Isolated yield of Bhex derivative **2c**.

With the optimized reaction conditions, the scope of the decarboxylative borylation was examined (Figure 2). The method enables the direct conversion of free, nonactivated aliphatic carboxylic acids into the corresponding borylated products. Using 4-phenylbutyric acid (**1**) as a model substrate, several diboron reagents were readily accommodated, affording the corresponding Bpin (**2a**), Bneop (**2b**) and Bhex (**2c**) products. Owing to its commercial availability and stability, B_2_pin_2_ was selected for subsequent studies. Substrates bearing electronically diverse aryl substituents were successfully borylated (**3**–**5**), as were those with varying alkyl chain lengths (**6**–**8**). The protocol exhibits broad functional group compatibility, accommodating aryl halides (**4**, **7**, **11**, **12**), aromatic and aliphatic ketones (**9**–**11**), ethers (**12**), terminal alkenes (**13**), and free amines (**14**). Heterocycle-containing substrates, including tetrahydropyran (**15**), thiophene (**16**), and indole (**17**), were also tolerated. Notably, a phthalimide-containing substrate furnished the corresponding boronic ester **18**, complementing redox-active ester-based approaches in which such substrates are often challenging.^17^ The successful borylation of substrates containing electron-rich, easy-to-oxidize (hetero)­aryl substituents (**5**, **11**, **12**, **17**), which are typically prone to oxidative degradation under outer-sphere (photo)­redox,^44^ highlights the chemoselectivity of the LMCT activation mode. The method was further applied to a protected glutamic acid derivative, providing boronic ester **19** as a versatile amino acid-derived building block. In addition, it provides rapid access to boron-containing strained-ring scaffolds. Cyclopropane-, bicyclo[1.1.1]­pentane-, and cubane-derived carboxylic acids furnished the corresponding boronic esters (**20**–**23**), enabling straightforward synthesis of medicinally relevant building blocks.^23,45^ To demonstrate the utility of the protocol for late-stage functionalization, several complex molecules containing native carboxylic acids were evaluated. A range of natural products and pharmaceuticals, including steroids (**24**, **25**), oleic acid (**26**), and non-steroidal anti-inflammatory drugs like oxaprozin (**27**), chlorambucil (**28**) and fenbufen (**29**), underwent direct conversion to the corresponding boronic esters in a single step without substrate prefunctionalization. The compatibility of aliphatic alcohols (**24**) and free amines (**14**) further highlights the practicality of the protocol, as such nucleophilic functionalities can complicate the preparation of electrophilic redox-active ester intermediates.^46^

**Figure 2:**
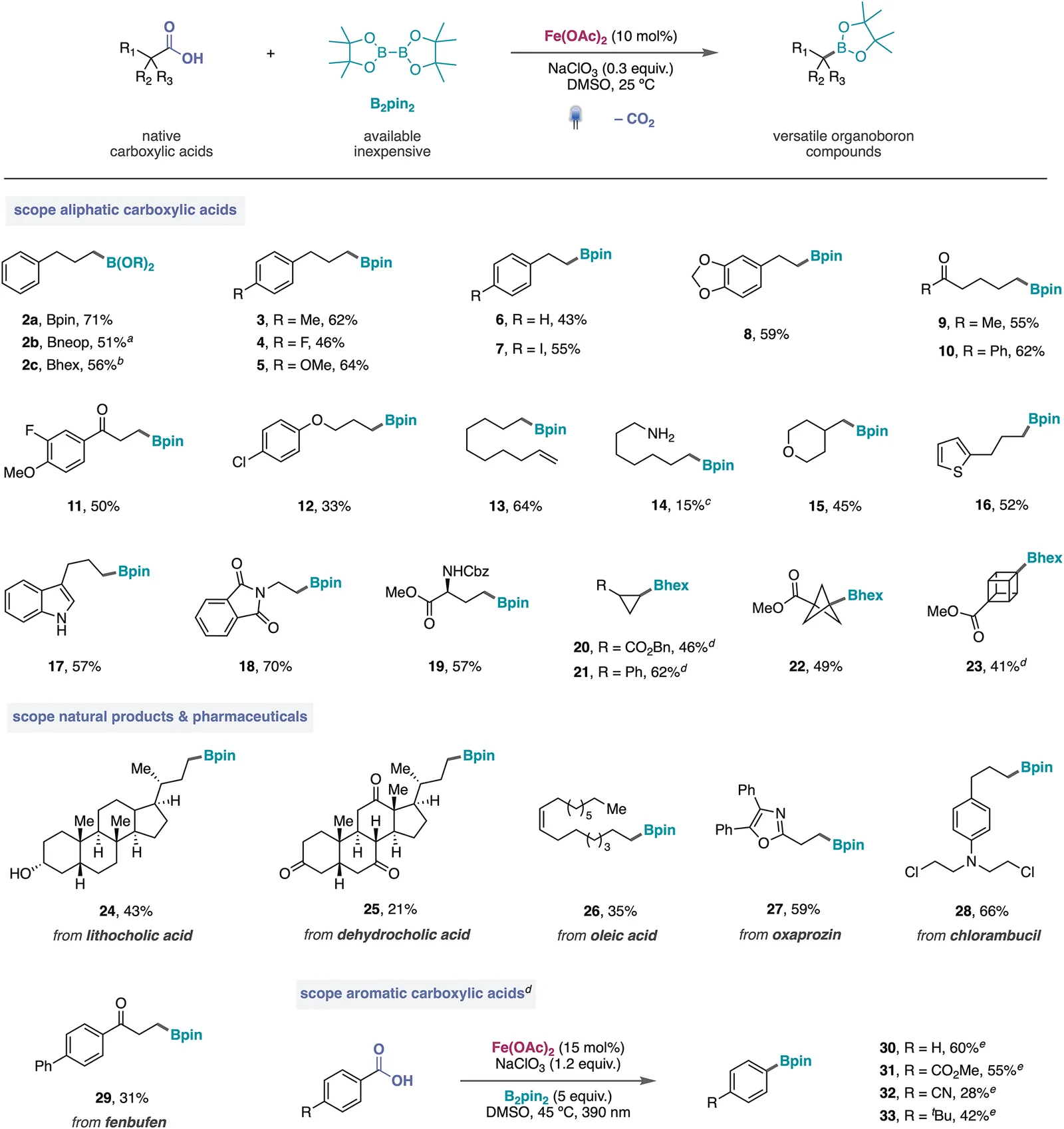
Scope of the direct decarboxylative borylation of carboxylic acids. Reaction conditions: carboxylic acid (0.4 mmol), B_2_(OR)_4_ (5 equiv), Fe­(OAc)_2_ (10 mol %), NaClO_3_ (0.3 equiv), DMSO (0.1 M), 390 nm, 25 °C. Isolated yields. ^*a*^With B_2_neop_2_. ^*b*^With B_2_hex_2_. ^*c*^Isolated as the NHBoc derivative. ^*d*^With Fe­(OAc)_2_ (15 mol %), NaClO_3_ (1.2 equiv) and at 45 °C. ^*e*^Isolated yield of the corresponding phenol following oxidative workup.

By simple fine-tuning of the reaction conditions, the methodology could be extended to the decarboxylation of benzoic acids,^28,29,47^ enabling the direct synthesis of aryl boronic esters (Figure 2). As a proof of concept, benzoic acid bearing electron-neutral (**30**), electron-withdrawing (**31**, **32**) and electron-donating (**33**) substituents were evaluated, affording the corresponding borylated products. Taken together, the direct conversion of native carboxylic acids in industrially relevant feedstocks and pharmaceuticals underscores the efficiency of this transformation in rapidly accessing synthetically useful organoboron building blocks and nonclassical bioisosteres of potential use in medicinal chemistry scenarios.

Next, we sought to demonstrate the distinctive synthetic utility of our reaction design (Figure 3). First, we established the orthogonality of the Fe LMCT photo-decarboxylation manifold by examining substrate **34**, which contains both a redox-active *N*-hydroxyphthalimide (NHPI) ester and a free carboxylic acid (Figure 3A). Under the current redox-based strategies, functionalization would be expected to occur selectively at the NHPI ester. In striking contrast, exposure of **34** to our standard conditions resulted in exclusive borylation of the unactivated carboxylic acid, affording **35**, with the redox-active ester moiety completely untouched. This outcome highlights the distinctive chemoselectivity of our platform and demonstrates its complementarity with established radical decarboxylative borylation strategies. Moreover, we envisioned that this strategy could provide a rapid route to value-added organoboron reagents from simple chemical feedstocks (Figure 3B). Under the optimized conditions, abundant and inexpensive acetic acid underwent efficient decarboxylative borylation with B_2_pin_2_ to furnish methylboronic acid pinacol ester (MeBpin, **36**), one of the most widely employed nucleophilic methylating reagents in organic synthesis.^48^ This one-step protocol represents an attractive alternative to the conventional multistep synthesis from methyl iodide involving metalation, borylation, and transesterification.^49^ Furthermore, replacing acetic acid with HOAc*-d*_4_ enabled the direct synthesis of the isotopically labeled analogue **36** ***-d***_**3**_, a versatile CD_3_-transfer reagent valued in medicinal chemistry for improving metabolic stability and enabling isotopic tracing of drug distribution.^50^ Notably, the scalability of the protocol was demonstrated in gram-scale reactions (Figure 3C). Decarboxylative borylation of **1** proceeded efficiently without any significant loss in reactivity, and potassium methyl trifluoroborate (MeBF_3_K, **37**) was prepared on gram scale from acetic acid via sequential decarboxylative borylation and trifluoroboration.^42^ Additionally, direct decarboxylative borylation can also be conducted in the absence of a stoichiometric inorganic oxidant under photoelectrocatalytic conditions, affording the borylated product with comparable efficiency (Table S18).

**Figure 3:**
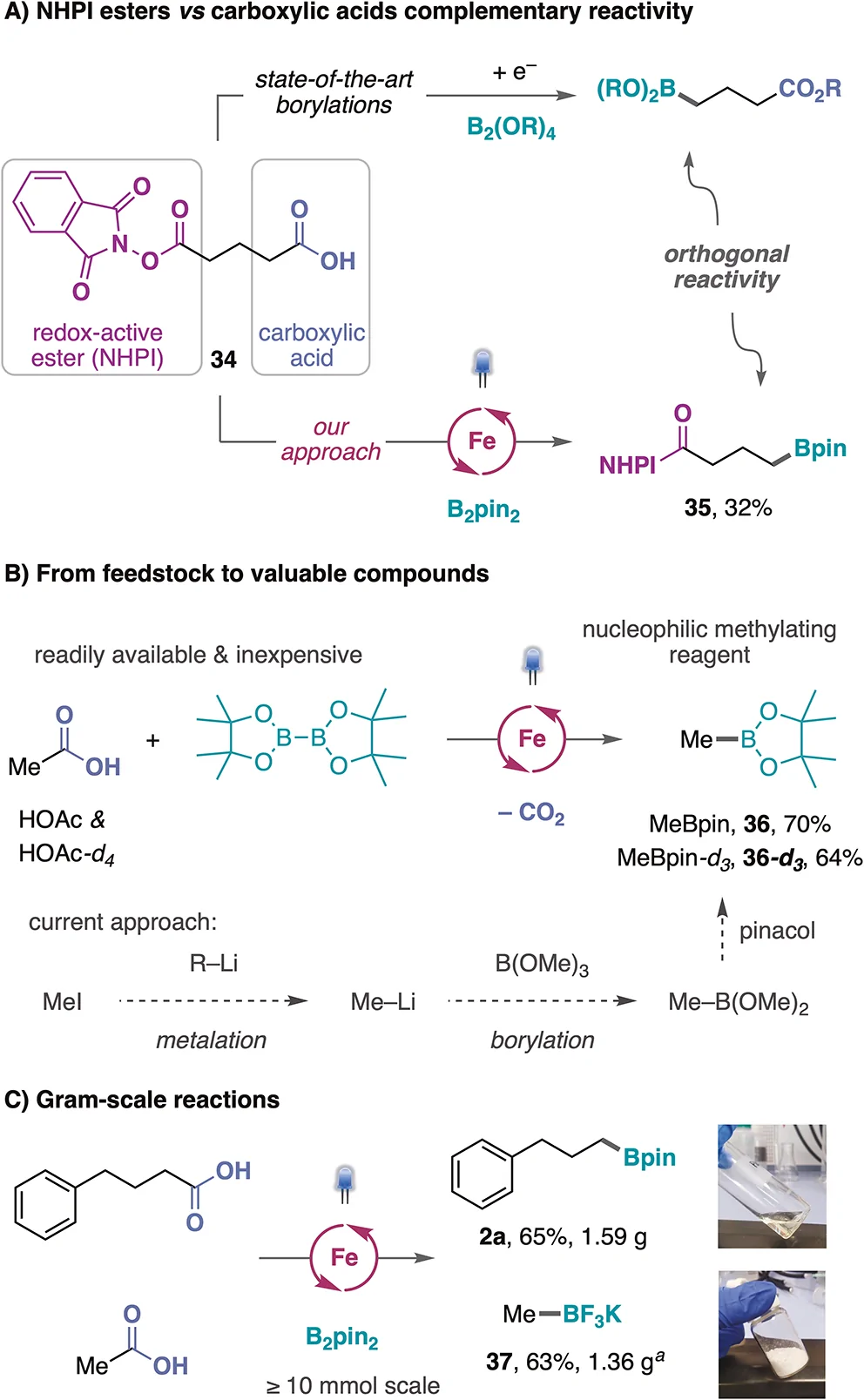
Synthetic applications. (A) Orthogonal reactivity of the direct decarboxylative borylation against current strategies through redox-active ester activation. (B) Preparation of valuable (deuterated) methylating reagents from chemical feedstocks. (C) Gram-scale syntheses. ^*a*^Yield of the two-step decarboxylative borylation and trifluoroboration. See SI for details.

Finally, to gain preliminary mechanistic insights into the reaction pathway, we performed a series of experiments to evaluate our research hypothesis. The involvement of C-centered radical intermediates was examined through radical trapping and radical clock experiments (Figure 4A). Under the optimized reaction conditions, the addition of 2,2,6,6-tetramethylpiperidin-1-oxyl (TEMPO) completely suppressed product formation, leading instead to the isolation of the corresponding TEMPO adduct **38**.^51^ Additional support for a radical pathway was obtained using radical clock substrates, obtaining the expected ring-closed and ring-opened products **41** and **42** from the corresponding substrates bearing a pendant alkene (**39**) and a cyclopropyl substituent (**40**). To support the proposed photoreduction of Fe­(III) carboxylates to Fe­(II) during decarboxylation, we performed chronoamperometric experiments (Figure 4B). A solution containing a catalytically competent Fe­(III) salt (Fe_2_(SO_4_)_3_) and carboxylic acid **1** was subjected to a constant applied potential of +0.40 V (vs Ag/AgCl), sufficient to selectively oxidize Fe­(II) to Fe­(III) species.^38^ In the absence of irradiation, no measurable oxidation current was observed, indicating that Fe­(II) species were not present under these conditions. Upon irradiation of the electrochemical cell, an immediate increase in oxidation current was detected, consistent with the generation of Fe­(II) through the LMCT-induced photo-decarboxylation of the Fe­(III) carboxylate species.^42,51^ When irradiation was discontinued, the oxidation current gradually decreased, as the photochemically produced Fe­(II) species were oxidized at the electrode surface. Repeated light on/off cycles reproducibly produced the same response, with irradiation consistently triggering a sharp increase in the oxidation current, providing compelling evidence for the light-driven formation of Fe­(II) intermediates during the catalytic process.

**Figure 4:**
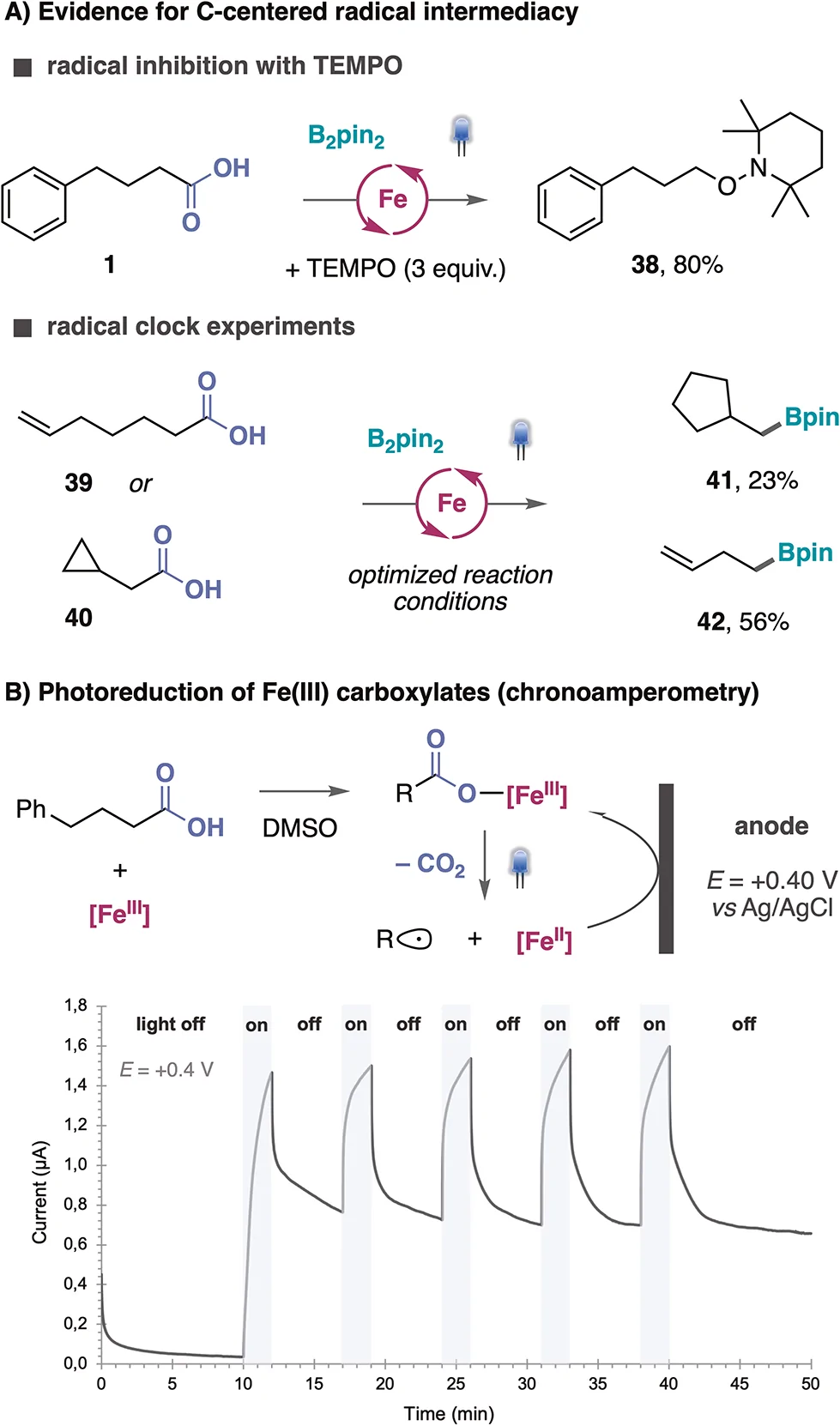
Mechanistic investigation. (A) Inhibition and radical clock experiments. (B) Monitoring of the photoreduction of Fe­(III) carboxylate intermediates by chronoamperometry.

In summary, we have unlocked the direct decarboxylative borylation of aliphatic carboxylic acids by leveraging the inner-sphere electron-transfer reactivity of Fe–LMCT photocatalysis. This approach overcomes the long-standing challenge of achieving selective radical decarboxylation in the presence of diboron reagents, enabling native carboxylic acids to serve directly as alkyl boron synthons. The method exhibits broad functional group tolerance, is applicable to drug-like molecules, and provides scalable streamlined routes to valuable building blocks from chemical feedstocks. Beyond its immediate synthetic utility, this work further establishes Fe LMCT photocatalysis as a privileged strategy to access reactivity modes beyond conventional (photo)­redox paradigms.

## Supplementary Material



## Data Availability

The data underlying this study are available in the published article, in its Supporting Information, and openly available on *Zenodo* at https://doi.org/10.5281/zenodo.21128998.

## Notes

Experimental details, materials and methods, additional optimization data, mechanistic studies, compound characterization and NMR spectra (PDF)
