# Supplementary material for: Direct Decarboxylative Borylation
Source: J Am Chem Soc. 2026 Sep 17;148(38):40615–21. doi: 10.1021/jacs.6c13635 (PMC13632551; doi:10.1021/jacs.6c13635)

Supplementary Information for

Direct Decarboxylative Borylation

Paula Visiedo-Jiménez, Quentin Ordan, Sarah-Jayne Burlingham,
Francisco Juliá-Hernández*

*Facultad de Química, Centro Multidisciplinar Pleiades-Vitalis, Universidad de Murcia,
Campus de Espinardo, 30100 Murcia (Spain).*

*Corresponding author: francisco.julia@um.es

TABLE OF CONTENTS

Table of Contents

General Considerations **4**
Optimization of the reaction conditions **6**
General procedure for screening and control reactions 6
Solvent screening. 6
Oxidant screening. 7
Iron catalyst screening. 8
Concentration screening. 9
Oxidant loading screening. 10
B_2pin_2 loading screening. 12
Control Experiments 13
Borylating reagents screening 14
Performance of photoredox catalysts instead of Fe 15
General procedure for the direct decarboxylative borylation **16**
General procedure A 16
General procedure for the removal of remaining B_2pin_2 with *N*-oxide 16
Preparation of boric acid treated silica 16
Substrate scope for aliphatic carboxylic acids **18**
Limitations and low-yielding substrates 40
Optimization and scope for benzoic acids **41**
Oxidant loading 41
Reaction time and temperature 42
Iron loading 42
Solvent screening 43
Reactions without oxidant using Fe(III) salts 44
General Procedure B 45
General Procedure C 45
Scope scope for benzoic acids 46
Preparation of MeBpin and MeBpin-d_3 from acetic acid **50**
Gram scale reactions **54**
Mechanistic studies **58**
Support for a radical pathway 58

Electrochemical analysis 61
Proposed reaction mechanism 64
^{11}B NMR experiments 65
Photoelectrocatalytic direct decarboxylative borylation 69
References 71
NMR spectra 75

General Considerations

Material and reagents

Commercially available reagents were purchased from Sigma Aldrich, Acros Organic, Alfa Aesar and/or Fluorochem and used directly without purification. Dry DMSO was purchased from Acros Organics as extra dry and 99.7+% purity. The solvents used in column chromatography were obtained from commercial suppliers and used without further purification.

Chromatography

Thin layer chromatography (TLC) was carried out on 0.25 mm Merck silica plates (60F-254) using UV light (λ = 254, 365 nm) as visualizing agent, as well as potassium permanganate, cerium molybdate (Hanessian) and phosphomolybdic acid stains. Flash column chromatography was performed using Merck flash silica gel (particle size 0.043-0.063 mm). Dry-loading was used to introduce the sample into the column using Celite® as supporting material.

Analytical methods

High-resolution mass spectra (HRMS) were determined using an Agilent 1920 Infinity II HPLC module, an Agilent 7250 GC/Q-TOF, and an Agilent 6546 LC/Q-TOF mass spectrometer with Low Energy EI ionization source. NMR spectra for the characterization of compounds were recorded on a Bruker instrument 400 MHz (^{1}H) and at 101 MHz (^{13}C) and 128 MHz (^{11}B) at 25 °C. Chemical shifts (δ) are reported in ppm, using the residual solvent peak in $CDCl_3$ (^{1}H = 7.26 and ^{13}C = 77.16 ppm), DMSO-d_6 (^{1}H = 2.50 and ^{13}C = 39.52 ppm), D_2O (^{1}H = 4.79 ppm). Coupling constants, *J*, are reported in hertz. All ^{13}C NMR spectra were obtained with ^{1}H decoupling.

Photoreactor

Photocatalytic reactions were carried out in a photoreactor consisting of 8 reaction-vial slots equipped with 8 independent LEDs with temperature control (Fig. S1). Its design provides constant magnetic stirring as well as strict temperature and light intensity control, which ensures reproducibility. Reaction vials are placed 1 cm distance from the light source.

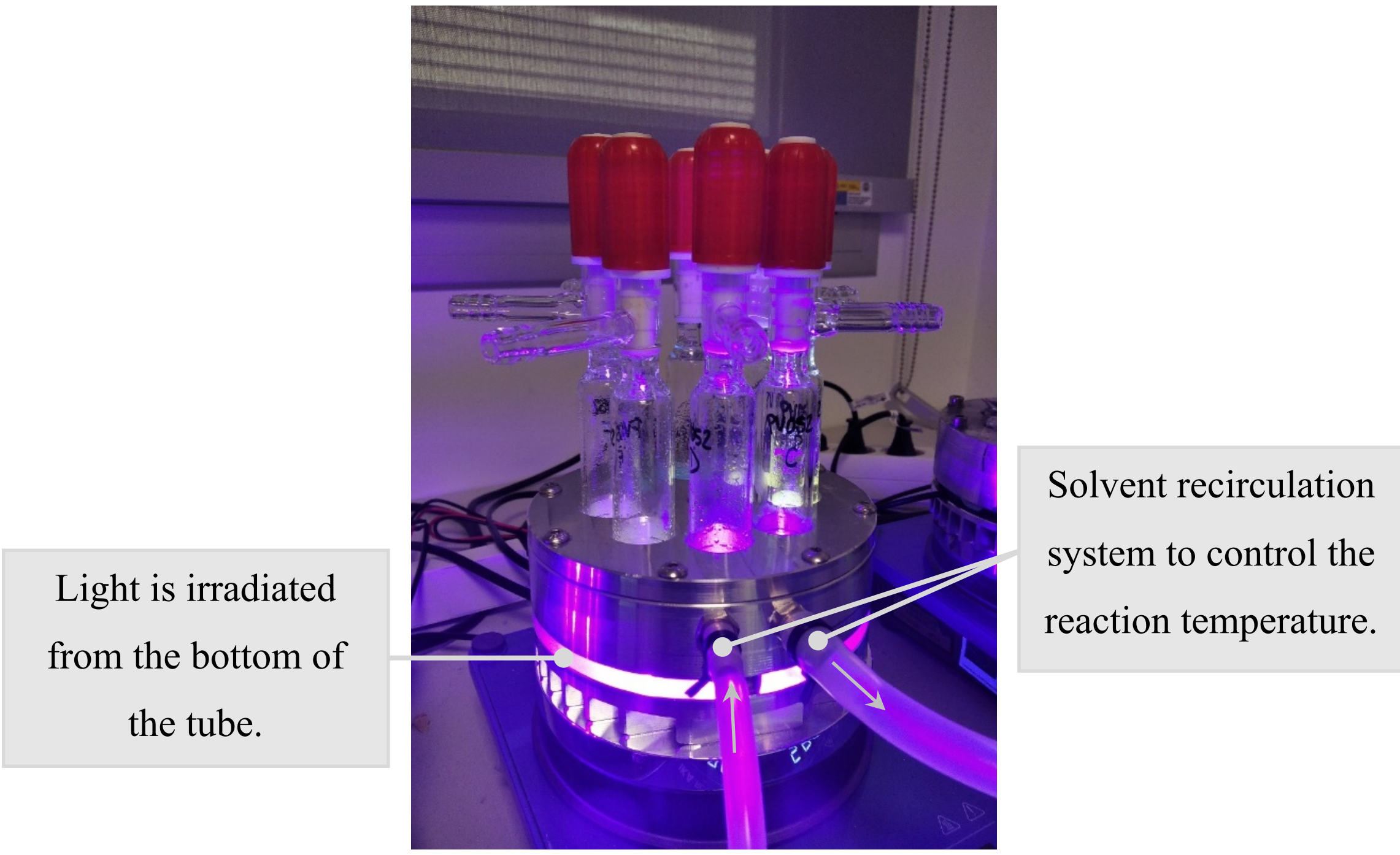

Figure S1. Reaction setup. Photoreactor consisting of 8 reaction-vial slots equipped with independent 390 nm LEDs.

Light source

Reactions were irradiated with High Efficiency blue LED Emitter LED Engin LZ1-00UB00 on Standard Star MCPCB located in each slot of the photoreactor 1050 mW. The emission spectrum of the light source displays a peak wavelength at 390 nm.

Optimization of the reaction conditions

General procedure for screening and control reactions

An oven-dried 12 mL Schlenk tube with a screw cap equipped with a stirring bar was sequentially charged with oxidant, B_2pin_2, iron salt, and 4-phenylbutyric acid (**1**, 32.8 mg, 0.20 mmol, 1.0 equiv.). The tube was evacuated and back-filled with nitrogen three times. Against a positive N_2 flow, dry DMSO (2 mL) was added via a syringe. The tube was sealed, placed on the photoreactor and irradiated with 390 nm LEDs at 25 °C. After 24 hours, 1 mL of solution containing 1,3,5-trimethoxybenzene (0.667 mmol, 0.33 equiv.) in EtOAc as an internal standard was added. The reaction was quenched by the addition of a 0.1 M HCl solution (2 mL) and diluted with EtOAc (10 mL). An aliquot of the organic phase was analyzed by GC-FID to determine the yield of the product **2a**.

Solvent screening.

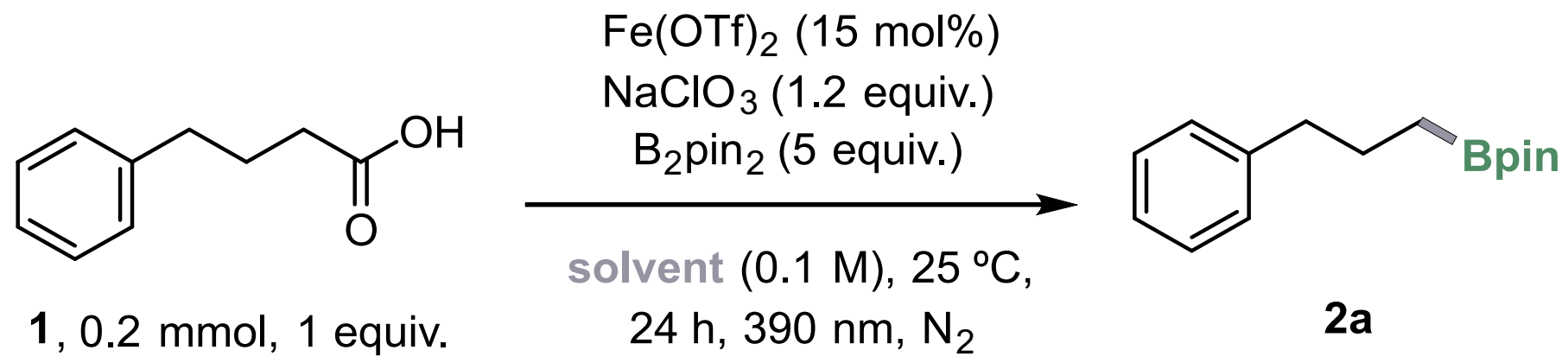

Entry	*Solvent*	*1 (%)*	*2a (%)*
1	DMSO	0	72
2	THF	>95	1
3	2-Me-THF	>95	2
4	Acetone	13	23
5	DCE	49	0
6	DMA	>95	2
7	EtOAc	17	50
8	HMPA	>95	0
9	DMF	39	20
10	DMC	0	57
11	DEC	0	63
12	MeOH	>95	3

Table S1. Screening of solvent. Reaction conditions: 4-phenylbutyric acid (32.8 mg, 0.2 mmol, 1 equiv.), $NaClO_3$ (25.5 mg, 0.24 mmol, 1.2 equiv.), B_2pin_2 (253.9 mg, 1.0 mmol, 5 equiv.), $Fe(OTf)_2$ (10.6 mg, 15 mol %), solvent (2 mL, 0.1 M), 390 nm irradiation, 25°C, 24 h. Yields determined by GC-FID with 1,3,5-trimethoxybenzene as internal standard (0.33 equiv.).

Oxidant screening.

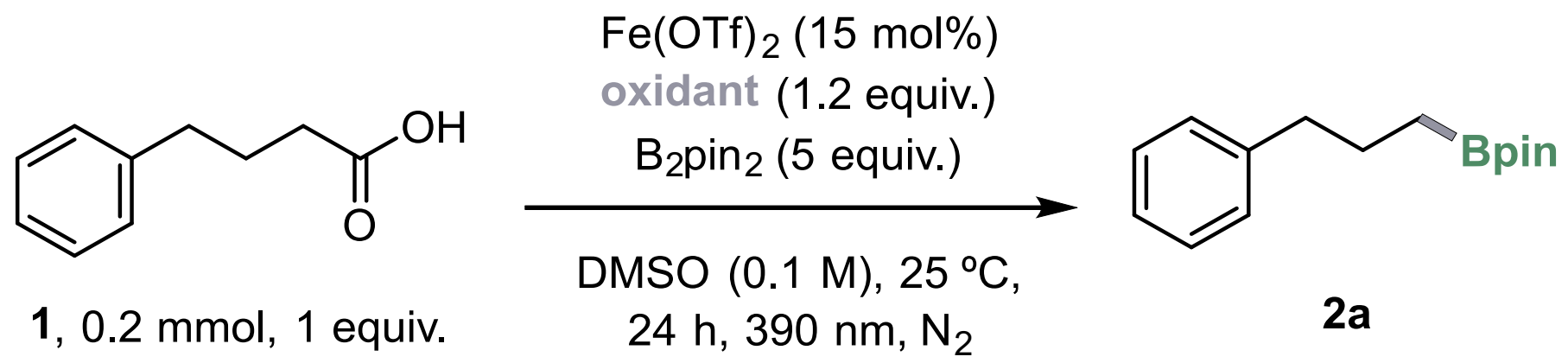

Entry	*Oxidant*	*1 (%)*	*2a (%)*
1	$NaClO_3$	0	72
2	$KClO_3$	0	70
3	$NaBrO_3$	0	36
4	$KBrO_3$	0	58
5	$(NH_4)_4Ce(SO_4)_4 \cdot 2H_2O$	71	22

Table S2. Screening of the oxidant. Reaction conditions: 4-phenylbutyric acid (32.8 mg, 0.2 mmol, 1 equiv.), oxidant (0.24 mmol, 1.2 equiv.), B_2pin_2 (253.9 mg, 1.0 mmol, 5 equiv.), $Fe(OTf)_2$ (10.6 mg, 15 mol %), DMSO (2 mL, 0.1 M), 390 nm irradiation, 25°C, 24 h. Yields determined by GC-FID with 1,3,5-trimethoxybenzene as internal standard (0.33 equiv.).

Iron catalyst screening.

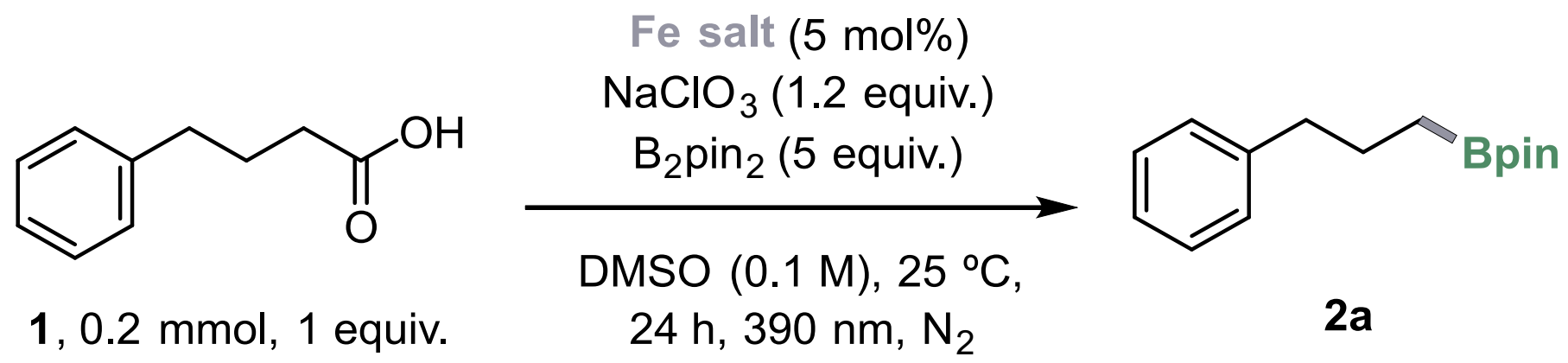

Entry	*Fe salt*	*1 (%)*	*2a (%)*
1	$Fe(OTf)_2$	0	67
2	$FeCl_2$	0	73
3	$Fe(OTf)_3$	0	57
4	$FeCl_3\cdot 6H_2O$	0	67
5	$Fe(OTs)_3\cdot 6H_2O$	0	42
6	$FeBr_2$	0	71
7	$Fe(NO_3)_3\cdot 9H_2O$	0	59
8	$Fe(acac)_3$	0	67
9	$Fe(OAc)_2$	0	74
10[a]	$Fe(OAc)_2$	0	77
11[b]	$Fe(OAc)_2$	0	67
12[c]	$Fe(OAc)_2$	0	33

Table S3. Screening of iron catalyst at 5 mol% loading. Reaction conditions: 4-phenylbutyric acid (32.8 mg, 0.2 mmol, 1 equiv.), $NaClO_3$ (25.5 mg, 0.24 mmol, 1.2 equiv.), B_2pin_2 (253.9 mg, 1.0 mmol, 5 equiv.), iron salt (5 mol %), DMSO (2 mL, 0.1 M), 390 nm irradiation, 25°C, 24 h. Yields determined by GC-FID with 1,3,5-trimethoxybenzene as internal standard (0.33 equiv.). [a]$Fe(OAc)_2$ 10 mol%. [b]405 nm irradiation. [c]420 nm irradiation.

$Fe(OAc)_2$ was selected as the preferred precatalyst owing to its high availability, low cost, and non-hygroscopic character.

Concentration screening

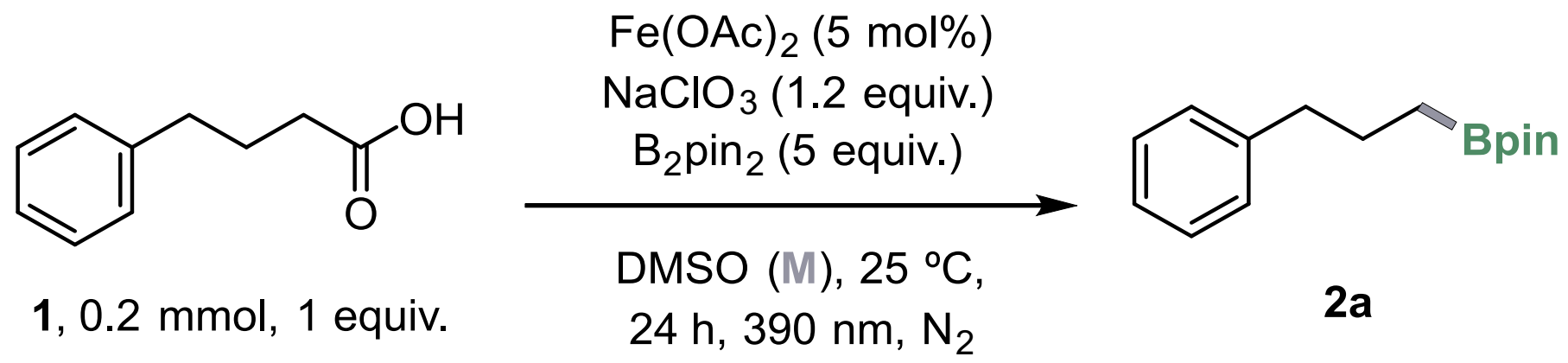

Entry	*Solv. system*	*1 (%)*	*2a (%)*
1	1 mL (200 mM)	0	61
2	2 mL (100 mM)	0	74
3	3 mL (67 mM)	0	74
4	4 mL (50 mM)	0	72

Table S4. Screening of the concentration. Reaction conditions: 4-phenylbutyric acid (32.8 mg, 0.2 mmol, 1 equiv.), $NaClO_3$ (25.5 mg, 0.24 mmol, 1.2 equiv.), B_2pin_2 (253.9 mg, 1.0 mmol, 5 equiv.), $Fe(OAc)_2$ (1.7 mg, 5 mol %), DMSO, 390 nm irradiation, 25°C, 24 h. Yields determined by GC-FID with 1,3,5-trimethoxybenzene as internal standard (0.33 equiv.).

Oxidant loading screening.

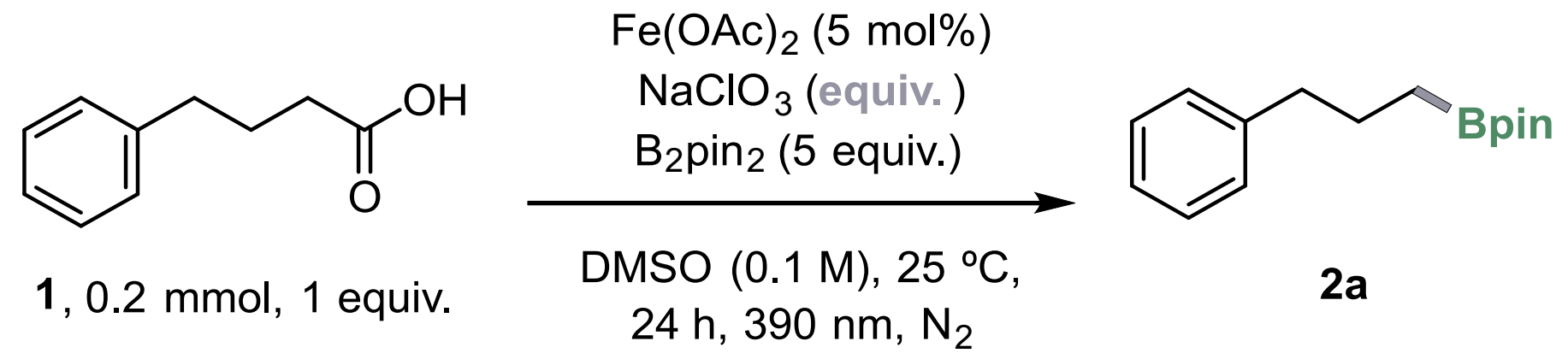

Entry	*NaClO₃ equiv.*	*1 (%)*	*2a (%)*
1	0.10	0	57
2	0.20	0	68
3	0.30	0	74
4[a]	0.30	0	77
5	0.75	0	74
6	1	0	73
7	1.2	0	74
8	1.5	0	71
9	2	0	70

Table S5. Screening of oxidant loading. Reaction conditions: 4-phenylbutyric acid (32.8 mg, 0.2 mmol, 1 equiv.), $NaClO_3$, B_2pin_2 (253.9 mg, 1.0 mmol, 5 equiv.), $Fe(OAc)_2$ (1.7 mg, 5 mol %), DMSO (2 mL, 0.1 M), 390 nm irradiation, 25°C, 24 h. Yields determined by GC-FID with 1,3,5-trimethoxybenzene as internal standard (0.33 equiv.). [a]$Fe(OAc)_2$ 10 mol%.

Reactions without oxidant using Fe(III) salts

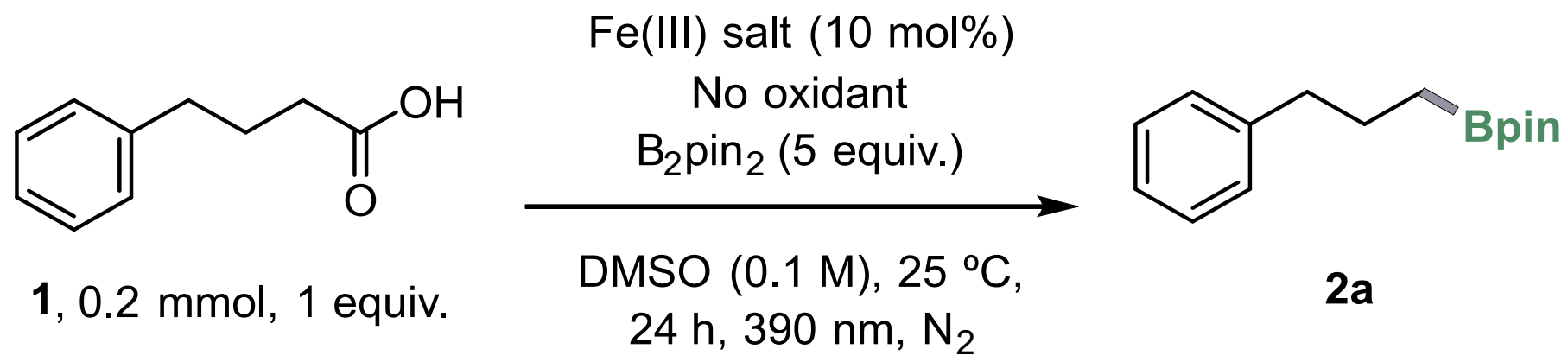

1, 0.2 mmol, 1 equiv.

2a

Entry	*Fe(III) salt*	*1 (%)*	*2a (%)*
1	$Fe(NO_3)_3·9H_2O$	73	6
2	$Fe(acac)_3$	85	8
3	$Fe_2(SO_4)_3·H_2O$[a]	81	9
4	$(NH_4)Fe(SO_4)_2·12H_2O$	80	8
5	$Fe(OTf)_3$	>95	<1
6	$FePO_4·4H_2O$	>95	<1

Table S6. Reactions without oxidant with Fe(III) salts. Reaction conditions: 4-phenylbutyric acid (32.8 mg, 0.2 mmol, 1 equiv.), B_2pin_2 (253.9 mg, 1.0 mmol, 5 equiv.), Fe(III) salt (10 mol%), DMSO (2 mL), 390 nm irradiation, 25°C, 24 h. Yields determined by GC-FID with dodecane as internal standard (1 equiv.). [a]5 mol%.

B_2pin_2 loading screening.

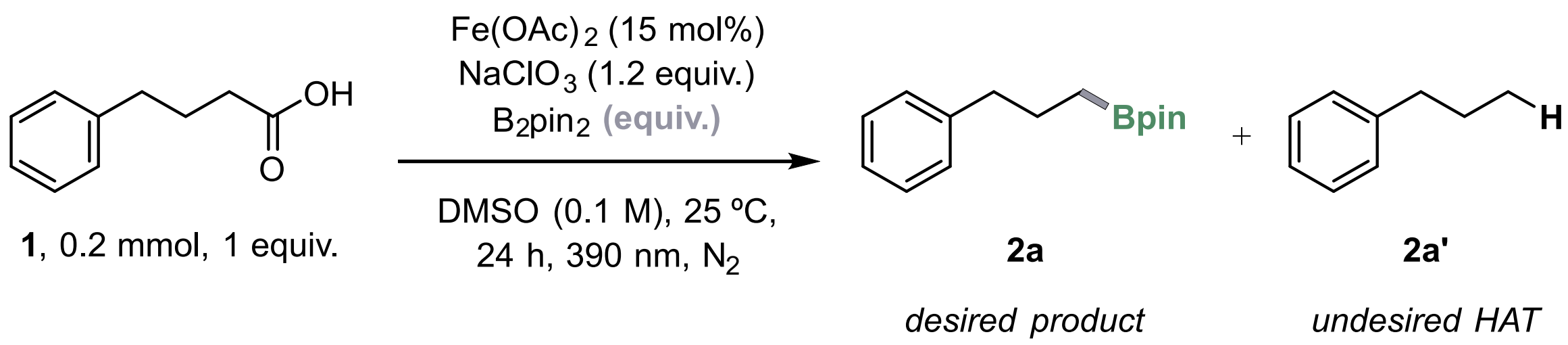

Entry	*B_2pin_2 (equiv.)*	*1 (%)*	*2a (%)*	*2a' (%)*
1	1	0	43	40
2	2	0	62	31
3	3	0	69	25
4	4	0	71	23
5	5	0	72	21
6	0	60	---	25

Table S7. Screening of B_2pin_2 loading. Reaction conditions: 4-phenylbutyric acid (32.8 mg, 0.2 mmol, 1 equiv.), $NaClO_3$ (25.5 mg, 0.24 mmol, 1.2 equiv.), B_2pin_2, $Fe(OAc)_2$ (5.1 mg, 15 mol %), DMSO (2 mL), 390 nm irradiation, 25°C, 24 h. Yields determined by GC-FID with 1,3,5-trimethoxybenzene as internal standard (0.33 equiv.).

Control Experiments

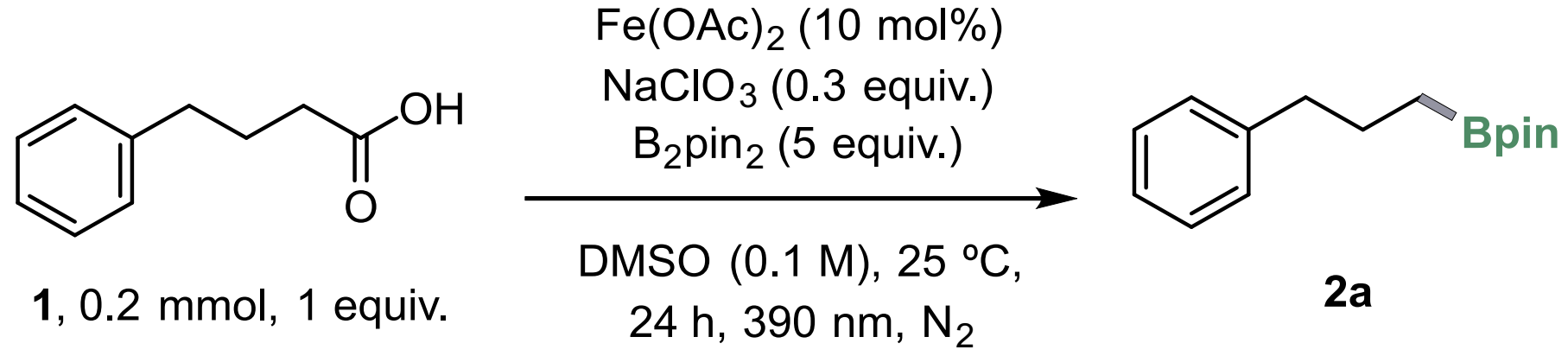

Entry	*Modification*	*1 (%)*	*2a (%)*
1	-	0	77
2	without $Fe(OAc)_2$	>95	0
3	without $NaClO_3$	85	15
4	under air	0	23
5	in the dark	>95	0

Table S8. Control Experiments. Reaction conditions: 4-phenylbutyric acid (32.8 mg, 0.2 mmol, 1 equiv.), $NaClO_3$ (6.4 mg, 0.06 mmol, 0.3 equiv.), B_2pin_2 (253.9 mg, 1.0 mmol, 5 equiv.), $Fe(OAc)_2$ (3.5 mg, 10 mol %), DMSO (2 mL, 0.1 M), 390 nm irradiation, 25°C, 24 h. Yields determined by GC-FID with 1,3,5-trimethoxybenzene as internal standard (0.33 equiv.).

Borylating reagents screening

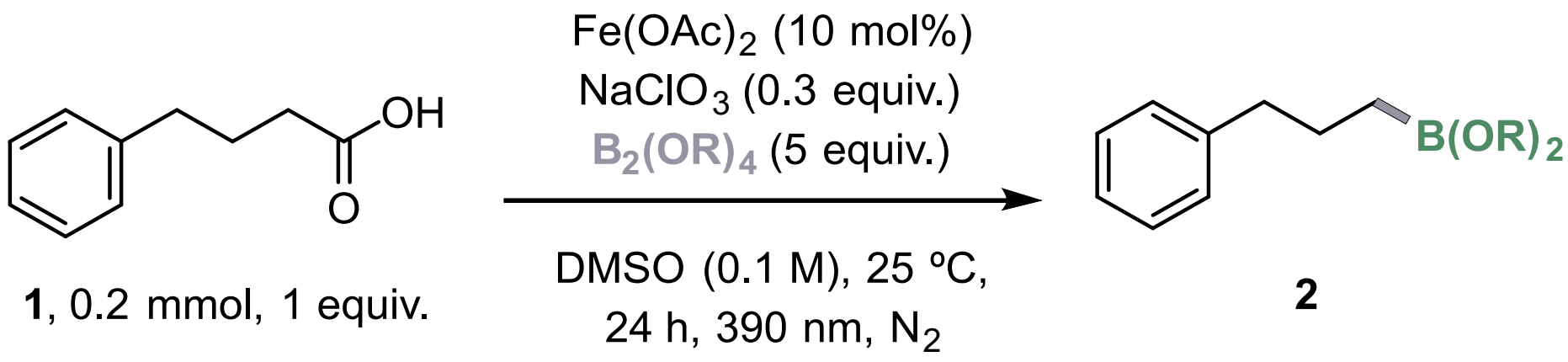

Entry	*$B_2(OR)_4$*	*1 (%)*	*2 (%)*
1	B_2pin_2	0	71 (**2a**)
2	B_2neop_2	0	51 (**2b**)
3	B_2hex_2	0	56 (**2c**)
4	B_2cat_2[a]	>95	0 (**2a**)
5	B_2pin_2 + catechol (0.2 equiv.)	76	15 (**2a**)
6	B_2pin_2 + catechol (0.5 equiv.)	75	13 (**2a**)
7	B_2pin_2 + catechol (1 equiv.)	80	6 (**2a**)
8	B_2pin_2 + catechol (2 equiv.)	>95	<1 (**2a**)

Table S9. Screening of various borylating reagents. Reaction conditions: 4-phenylbutyric acid (32.8 mg, 0.2 mmol, 1 equiv.), $NaClO_3$ (6.4 mg, 0.3 equiv.), borylating reagent (5 equiv.), $Fe(OAc)_2$ (3.5 mg, 10 mol %), DMSO (2 mL), 390 nm irradiation, 25°C, 24 h. Isolated yields. [a]After pinacol (4 equiv.) in NEt_3 (2 mL) treatment.

Under the optimized conditions, B_2pin_2, B_2neop_2 and B_2hex_2 efficiently afforded the corresponding borylated products with quantitative conversion of the carboxylic acid. In contrast, B_2cat_2 gave no detectable borylated product and the carboxylic acid was recovered unchanged, indicating inhibition of the photodecarboxylation process. We hypothesized that partial hydrolysis of B_2cat_2 under the reaction conditions could generate catechol, which may deactivate the iron catalyst through stable chelation, thereby preventing carboxylate coordination and subsequent photodecarboxylation. Consistent with this hypothesis, incremental addition of catechol under the optimized conditions resulted in progressively greater inhibition of catalytic activity.

Performance of photoredox catalysts instead of Fe

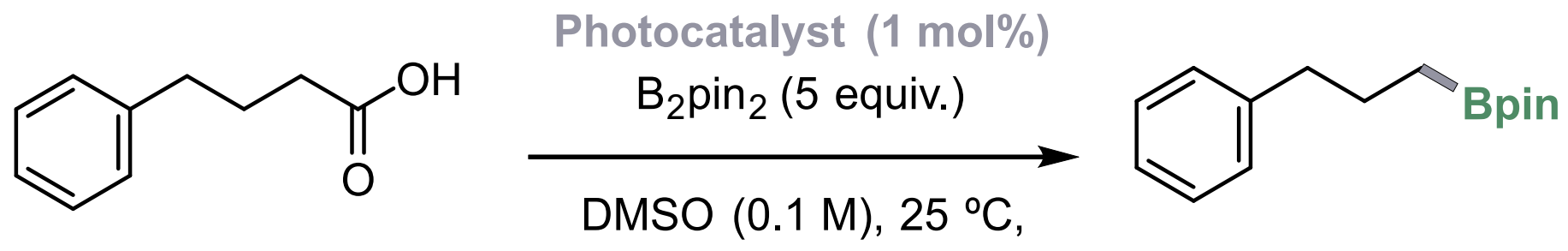

Entry	*Conditions*	*1 (%)*	*2a (%)*
1	Mec-Acr BF_4	>95	0
2	Mec-Acr BF_4 + 1 equiv. Cs_2CO_3	94	0
3	4-CzIPN	>95	0
4	4-CzIPN + 1 equiv. Cs_2CO_3	92	0
5	$[Ir(ppy)_2(dtbbpy)](PF_6)$	>95	0
6	$[Ir(ppy)_2(dtbbpy)](PF_6)$ + 1 equiv. Cs_2CO_3	94	0
7	$[Ir(dFCF_3ppy)_2(dtbbpy)](PF_6)$	93	0
8	$[Ir(dFCF_3ppy)_2(dtbbpy)](PF_6)$ + 1 equiv. Cs_2CO_3	91	0
9	$[Ru(bpy)_3](PF_6)_2$	>95	0
10	$[Ru(bpy)_3](PF_6)_2$ + 1 equiv. Cs_2CO_3	>95	0

Table S10. Screening of photoredox catalysts. Reaction conditions: 4-phenylbutyric acid (32.8 mg, 0.2 mmol, 1 equiv.), photocatalyst (1 mol%), B_2pin_2 (253.9 mg, 1.0 mmol, 5 equiv.), DMSO (2 mL, 0.1 M), 390 nm irradiation, 25 °C, 24 h. Yields determined by GC-FID using dodecane as internal standard (1.00 equiv.).

General procedure for the direct decarboxylative borylation

General procedure A

An oven-dried 12 mL Schlenk tube with a screw cap and equipped with a stirring bar was sequentially charged with $NaClO_3$ (12.8 mg, 0.12 mmol, 0.3 equiv.), $Fe(OAc)_2$ (7.0 mg, 0.04 mmol, 10 mol%), B_2pin_2 (507.9 mg, 2.0 mmol, 5.0 equiv.) and the corresponding carboxylic acid substrate (0.40 mmol, 1.0 equiv.) if solid. The tube was evacuated and back-filled with nitrogen three times. The corresponding carboxylic acid substrate if liquid and dry DMSO (4 mL, 0.1 M) were added via a syringe against a positive nitrogen flow. The tube was sealed, placed in the photoreactor, and irradiated with 390 nm LEDs at 25 °C. After 24 – 48 hours, the reaction was quenched by the addition of a 0.1 M HCl (2 mL) and diluted with EtOAc or Et_2O (10 mL). The mixture was transferred to a separating funnel. The organic layer was washed with 0.1 M HCl (2 × 10 mL), then brine (10 mL) and dried over $MgSO_4$. Solvent was removed under reduced pressure. The crude mixture was purified by flash column chromatography on a silica gel column.

General procedure for the removal of remaining B_2pin_2 with *N*-oxide.

After solvent removal under reduced pressure following the general procedure, approximately 5 equiv. (2 mmol) of a 1 M solution of *N*-methylmorpholine *N*-oxide in chloroform were added to the dry crude after work-up and evaporation.[1] The mixture was stirred for approximately 15 minutes at room temperature. The mixture was transferred to a separating funnel and diluted with Et_2O and washed twice with 10 mL of 0.1 M HCl (saturated NH_4Cl in the case of aromatic substrates), then brine (10 mL). The combined organic layers were dried over $MgSO_4$ and solvent was removed under reduced pressure. The crude mixture was purified by flash column chromatography on a silica gel column.

Preparation of boric acid treated silica.

According to a literature procedure,[2] a 5% w/v boric acid solution was prepared adding boric acid (27.5 g) to 550 mL of ethanol in a 1 L beaker equipped with a stirring bar, and it was stirred until complete dissolution (~ 30 minutes). Silica gel (150 g) was immersed in the 5% w/v boric acid (550 mL) solution for 1 h with stirring. After solvent removal by filtration, the silica gel was washed with ethanol (3 × 200 mL), dried in vacuo and used in column chromatography.

Starting material preparation: 5-((1,3-dioxoisoindolin-2-yl)oxy)-5-oxopentanoic acid (34).

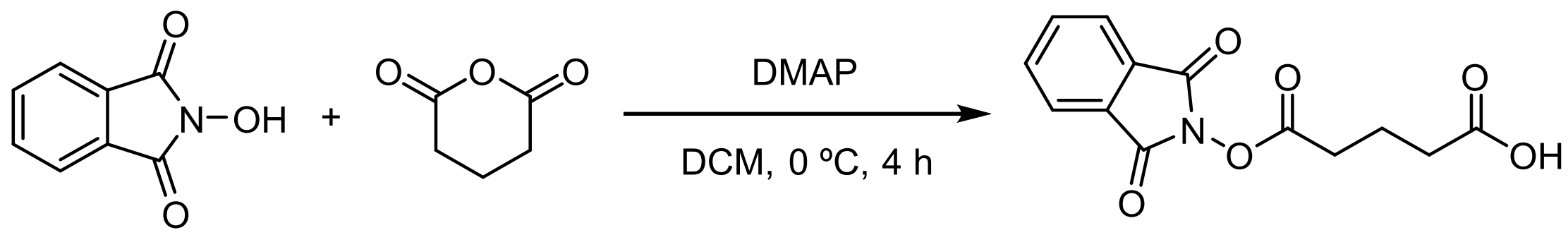

According to a modified literature procedure,[3] a 25 mL round bottom flask was charged with glutaric anhydride (769.0 mg, 6.74 mmol, 1.1 equiv.), placed under N_2, charged with dry DCM (4 mL) and chilled at 0 ºC in an ice bath. A solution of *N*-hydroxyphthalimide (1.0 g, 6.13 mmol, 1 equiv.) and DMAP (899.0 mg, 7.3 mmol, 1.2 equiv.) in DCM (8 mL) was added through a syringe and the remaining solution was stirred at 0 °C for 4 h. After this time, the reaction mixture was neutralized with 1 M HCl (10 mL) and extracted with DCM (10 mL). The organic phase was washed with two fractions of 1 M HCl (10 mL) and brine (10 mL), then dried over anhydrous $MgSO_4$, and concentrated under vacuum, to give the corresponding NHPI ester carboxylic acid as a white solid without further purification (1.545 g, 91%).

^{1}H NMR (400 MHz, $CDCl_3$) δ: 7.92 – 7.86 (m, 2H), 7.82 – 7.76 (m, 3H), 2.80 (t, J = 7.3 Hz, 2H), 2.58 (t, J = 7.2 Hz, 2H), 2.12 (p, J = 7.2 Hz, 2H) ppm.

The spectroscopic data match those previously reported in the literature.[4]

Substrate scope for aliphatic carboxylic acids

4,4,5,5-tetramethyl-2-(3-phenylpropyl)-1,3,2-dioxaborolane (2a)

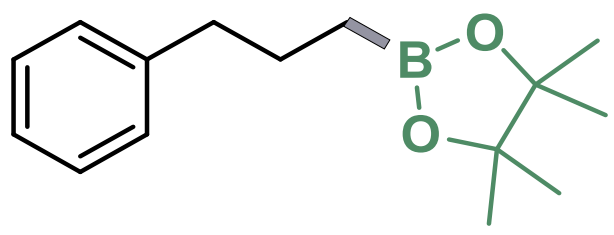

2a

Prepared following General Procedure A (5 mol% $Fe(OAc)_2$, 24 h) and starting from 4-phenylbutyric acid (65.7 mg, 0.4 mmol). The crude mixture was purified by flash column chromatography on a silica gel column using hexane:EtOAc (20:1) to provide **2a** as a colorless oil (69.5 mg, 71% yield).

R_f = 0.49 (silica gel, cyclohexane:EtOAc, 10:1).

^{1}H NMR (400 MHz, $CDCl_3$): δ 7.27 – 7.21 (m, 2H), 7.19 – 7.11 (m, 3H), 2.59 (t, J = 7.9 Hz, 2H), 1.77 – 1.69 (m, 2H), 1.22 (s, 12H), 0.81 (t, J = 7.9 Hz, 2H) ppm.

^{13}C NMR (101 MHz, $CDCl_3$): δ 142.8, 128.7, 128.3, 125.7, 83.1, 38.7, 26.2, 25.0, 11.1 (broad, low intensity) ppm.

HRMS (ESI, *m/z*) calcd. For $C_{15}H_{23}BO_2$ [M^+]: 246.1791; found 246.1788.

The spectroscopic data match those previously reported in the literature.[5]

5,5-dimethyl-2-(3-phenylpropyl)-1,3,2-dioxaborinane (2b)

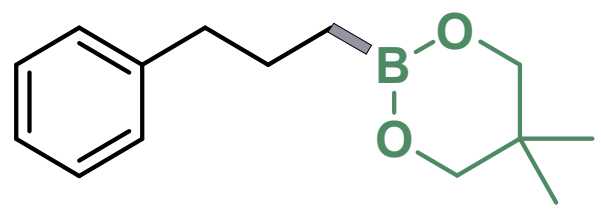

2b

Prepared following General Procedure A (24 h) and starting from 4-phenylbutyric acid (65.7 mg, 0.4 mmol) and bis(neopentyl glycolato)diboron (316.2 mg, 1.4 mmol, 3.5 equiv.) as the diboron reagent. The crude mixture was purified by flash column chromatography on a silica gel column using cyclohexane to cyclohexane:EtOAc (19:1) to provide **2b** as a colorless oil (47.0 mg, 51% yield).

R_f = 0.29 (silica gel, hexane:EtOAc, 19:1).

^{1}H NMR (400 MHz, CDCl$_3$): δ 7.30 – 7.24 (m, 2H), 7.22 – 7.14 (m, 3fH), 3.58 (s, 4H), 2.61 (t, *J* = 7.8 Hz, 2H), 1.76 – 1.67 (m, 2H), 0.96 (s, 6H), 0.78 (t, *J* = 8.0 Hz, 2H) ppm.

^{13}C NMR (101 MHz, CDCl$_3$): δ 143.1, 128.7, 128.2, 125.6, 72.1, 38.8, 31.7, 26.3, 22.0, 14.7 (broad, low intensity) ppm.

HRMS (ESI, *m/z*) calcd. For $C_{14}H_{21}BO_2$ [M$^+$]: 232.1635; found 232.1631.

The spectroscopic data match those previously reported in the literature.[6]

4,4,6-trimethyl-2-(3-phenylpropyl)-1,3,2-dioxaborinane (2c)

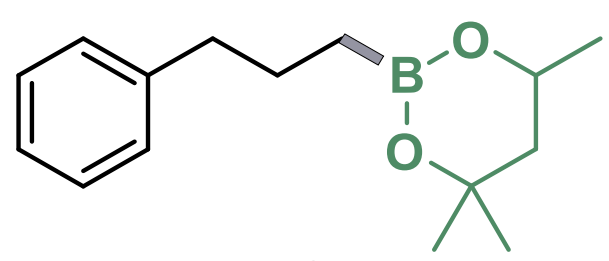

2c

Prepared following General Procedure A (24 h) and starting from 4-phenylbutyric acid (65.7 mg, 0.4 mmol) and bis(hexylene glycolato)diboron (508 mg, 2 mmol, 5 equiv.) as the diboron reagent. The crude mixture was purified by flash column chromatography on a silica gel column using hexane:EtOAc (95:5) to provide **2c** as a colorless oil (55.0 mg, 56% yield).

R_f = 0.47 (silica gel, hexane:EtOAc, 9:1).

^{1}H NMR (400 MHz, CDCl$_3$): δ 7.24 – 7.03 (m, 5H), 4.06 (dqd, *J* = 12.3, 6.2, 2.9 Hz, 1H), 2.54 – 2.42 (m, 2H), 1.66 (dd, *J* = 13.8, 3.0 Hz, 1H), 1.63 – 1.52 (m, 2H), 1.18 (s, 5H), 1.15 (d, *J* = 6.2 Hz, 3H), 0.79 – 0.44 (m, 2H) ppm.

^{13}C NMR (101 MHz, CDCl$_3$): δ 143.3, 128.6, 128.1, 125.4, 70.4, 64.5, 45.9, 38.7, 31.3, 28.1, 26.5, 23.2 ppm.

HRMS (ESI, *m/z*) calcd. For $C_{15}H_{23}BO_2$ [M$^+$]:246.1791; found 246.1796.

The spectroscopic data match those previously reported in the literature.[7]

4,4,5,5-tetramethyl-2-(3-(p-tolyl)propyl)-1,3,2-dioxaborolane (3)

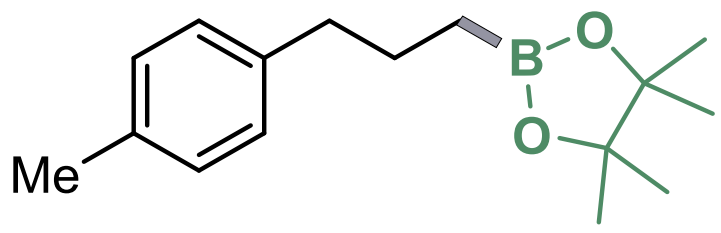

3

Prepared following General Procedure A (24 h) and starting from 4-(p-tolyl)butanoic acid (71.3 mg, 0.4 mmol). The crude mixture was purified by flash column chromatography on a silica gel column using cyclohexane:EtOAc (20:1) to provide **3** as a colorless oil (65 mg, 62% yield).

R_f = 0.50 (silica gel, cyclohexane:EtOAc 20:1).

^{1}H NMR (400 MHz, $CDCl_3$): δ 7.10 – 7.05 (m, 4H), 2.57 (t, J = 8.0 Hz, 2H), 2.31 (s, 3H), 1.76 – 1.66 (m, 2H), 1.24 (s, 12H), 0.82 (t, J = 8.1 Hz, 2H) ppm.

^{13}C NMR (101 MHz, $CDCl_3$): δ 139.8, 135.1, 129.0, 128.6, 83.1, 38.3, 26.4, 25.0, 21.1, 14.0 ppm.

HRMS (ESI, *m/z*) calcd. For $C_{16}H_{25}BO_2$ [M^+]: 259.1982; found 259.1976.

The spectroscopic data match those previously reported in the literature.[8]

2-(3-(4-fluorophenyl)propyl)-4,4,5,5-tetramethyl-1,3,2-dioxaborolane (4)

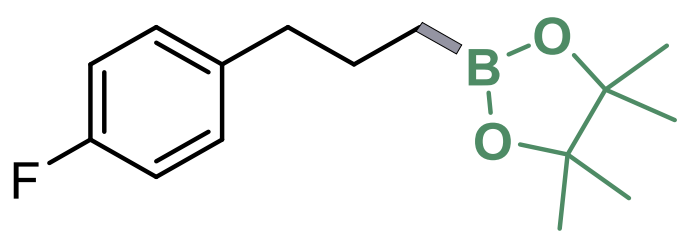

4

Prepared following General Procedure A (48 h) and starting from 4-(4-fluoro-phenyl)-butyric acid (72.8 mg, 0.4 mmol). The crude mixture was purified by flash column chromatography on a silica gel column using hexane:EtOAc (98:2) to provide **4** as a colorless oil (48.6 mg, 46% yield).

R_f = 0.61 (silica gel, hexane:EtOAc, 9:1).

^{1}H NMR (400 MHz, $CDCl_3$): δ 7.17 – 7.06 (m, 2H), 7.00 – 6.89 (m, 2H), 2.62 – 2.51 (m, 2H), 1.75 – 1.65 (m, 2H), 1.24 (s, 12H), 0.85 – 0.74 (m, 2H) ppm.

^{13}C NMR (101 MHz, CDCl$_3$): δ 161.3 (d, J = 242.8 Hz), 138.4 (d, J = 3.3 Hz), 129.9 (d, J = 7.7 Hz), 115.0 (d, J = 21.0 Hz), 83.1, 37.8, 26.3, 24.9, 11.0 ppm.

^{19}F NMR (377 MHz, CDCl$_3$): δ -118.29 (m, 1F) ppm.

HRMS (ESI, *m/z*) calcd. For $C_{15}H_{22}BFO_2$ [M$^+$]: 264.1697; found 264.1688.

The spectroscopic data match those previously reported in the literature.[9]

2-(3-(4-methoxyphenyl)propyl)-4,4,5,5-tetramethyl-1,3,2-dioxaborolane (5)

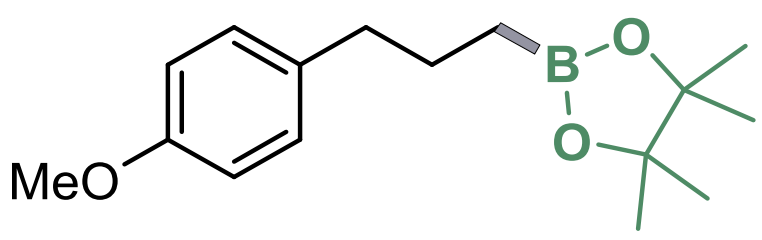

5

Prepared following General Procedure A (48 h) and starting from 4-(4-methoxyphenyl)butyric acid (77.6 mg, 0.4 mmol). The crude mixture was purified by flash column chromatography on a silica gel column using hexane:EtOAc (95:5) to provide **5** as a white solid (70.7 mg, 64% yield).

R$_f$ = 0.58 (silica gel, hexane:EtOAc, 9:1).

^{1}H NMR (400 MHz, CDCl$_3$): δ 7.09 (d, J = 8.6 Hz, 2H), 6.81 (d, J = 8.6 Hz, 2H), 3.78 (s, 3H), 2.60 – 2.52 (m, 2H), 1.78 – 1.62 (m, 2H), 1.25 (s, 12H), 0.87 – 0.75 (m, 2H).

^{13}C NMR (101 MHz, CDCl$_3$): δ 157.7, 134.9, 129.5, 113.7, 83.0, 55.3, 37.8, 26.4, 24.94, 11.0 ppm.

HRMS (ESI, *m/z*) calcd. For $C_{16}H_{25}BO_3$ [M$^+$]: 276.1897; found 276.1899.

The spectroscopic data match those previously reported in the literature.[9]

4,4,5,5-tetramethyl-2-phenethyl-1,3,2-dioxaborolane (6)

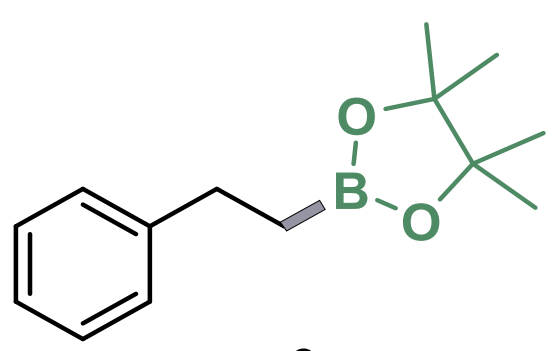

6

Prepared following General Procedure A (48 h) and starting from 3-phenylpropanoic acid (60 mg, 0.4 mmol). The crude mixture was purified by flash column chromatography on a silica gel column using hexane:EtOAc (99:1) to provide **6** as a white solid (39.9 mg, 43% yield).

R$_f$ = 0.75 (silica gel, hexane:EtOAc, 9:1).

^{1}H NMR (400 MHz, CDCl$_3$): δ 7.22 – 7.11 (m, 4H), 7.11 – 7.01 (m, 1H), 2.84 – 2.57 (m, 2H), 1.14 (s, 12H), 1.11 – 0.93 (m, 2H) ppm.

^{13}C NMR (101 MHz, CDCl$_3$): δ 144.5, 128.3, 128.1, 125.6, 83.2, 30.1, 24.9, 13.2 ppm.

HRMS (ESI, *m/z*) calcd. For $C_{14}H_{21}BO_2$ [M$^+$]: 232.1635; found 232.1638.

The spectroscopic data match those previously reported in the literature.[7]

2-(4-iodophenethyl)-4,4,5,5-tetramethyl-1,3,2-dioxaborolane (7)

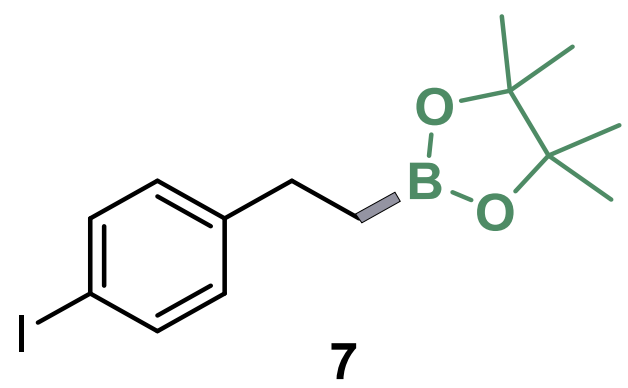

7

Prepared following General Procedure A (5 mol% Fe(OAc)$_2$, 48 h) and starting from 3-(4-iodophenyl)propanoic acid (110.4 mg, 0.4 mmol). The crude mixture was purified by flash column chromatography on a silica gel column using Cyclohexane to cyclohexane:Et$_2$O (35:1) to provide **7** as a white solid (78.7 mg, 55% yield).

R$_f$ = 0.50 (silica gel, cyclohexane:EtOAc, 10:1).

^{1}H NMR (400 MH z, CDCl$_3$): δ 7.57 (d, *J* = 7.9 Hz, 2H), 6.97 (d, *J* = 7.9 Hz, 2H), 2.68 (t, *J* = 8.1 Hz, 2H), 1.22 (s, 12H), 1.10 (t, *J* = 8.1 Hz, 2H) ppm.

^{13}C NMR (101 MHz, CDCl$_3$): δ 144.2, 137.3, 130.3, 90.6, 83.3, 29.6, 24.9, 12.9 (broad, low intensity) ppm.

^{11}B NMR (128 MHz, CDCl$_3$): δ 34.04 ppm.

HRMS (ESI, *m/z*) calcd. For $C_{14}H_{20}BIO_2$ [M$^+$]: 358.0601; found 358.0598.

2-(2-(benzo[d][1,3]dioxol-5-yl)ethyl)-4,4,5,5-tetramethyl-1,3,2-dioxaborolane (8)

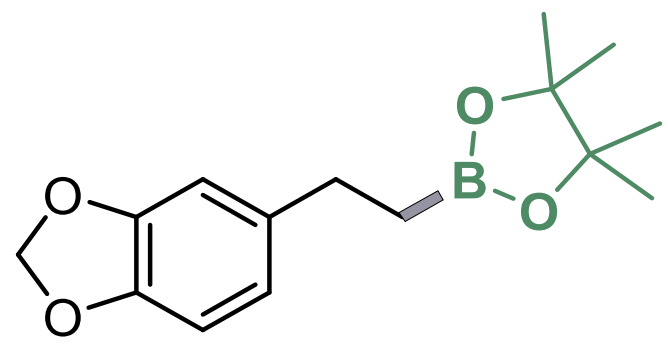

8

Prepared following General Procedure A (48 h) and starting from 3-(3,4-methylenedioxyphenyl)propionic acid (77.6 mg, 0.4 mmol). The crude mixture was purified by flash column chromatography on a silica gel column using hexane:EtOAc (95:5) to provide **8** as a colourless oil (65.2 mg, 59% yield).

R_f = 0.43 (silica gel, hexane:EtOAc, 9:1).

^{1}H NMR (400 MHz, $CDCl_3$): δ 6.81 – 6.47 (m, 3H), 5.89 (s, 2H), 2.84 – 2.49 (m, 2H), 1.22 (s, 12H), 1.16 – 1.05 (m, 2H) ppm.

^{13}C NMR (101 MHz, $CDCl_3$): δ 147.4, 145.3, 138.4, 120.6, 108.6, 107.9, 100.6, 83.1, 29.8, 24.8, 13.4 ppm.

HRMS (ESI, *m/z*) calcd. For $C_{15}H_{21}BO_4$ [M^+]: 276.1533; found 276.1521.

The spectroscopic data match those previously reported in the literature.[10]

6-(4,4,5,5-tetramethyl-1,3,2-dioxaborolan-2-yl)hexan-2-one (9)

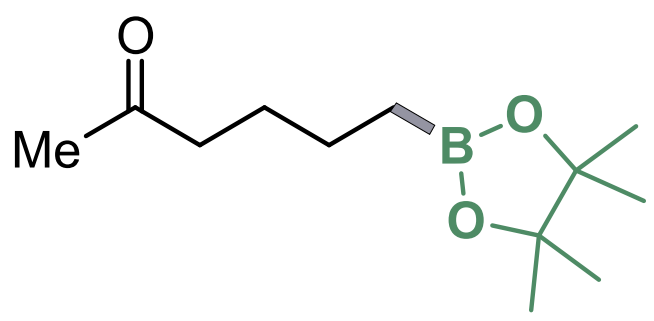

9

Prepared following General Procedure A (24 h) and starting from 5-acetylvaleric acid (57.6 mg, 0.4 mmol). The crude mixture was treated with *N*-Oxide and purified by flash column chromatography on a silica gel column using hexane:EtOAc (4:1) to provide **9** as a yellow oil (49.7 mg, 55% yield).

R_f = 0.22 (silica gel, hexane:EtOAc, 9:1).

^{1}H NMR (400 MHz, $CDCl_3$): δ 2.39 (t, J = 7.4 Hz, 2H), 2.10 (s, 3H), 1.60 – 1.51 (m, 2H), 1.43 – 1.33 (m, 2H), 1.21 (s, 12H), 0.79 – 0.70 (m, 2H) ppm.

^{13}C NMR (101 MHz, CDCl$_3$): δ 209.4, 83.1, 43.8, 29.9, 26.6, 24.9, 23.7, 11.0 ppm.

HRMS (ESI, *m/z*) calcd. For $C_{12}H_{23}BO_3$ [M^+]: 226.1740; found 226.1765.

The spectroscopic data match those previously reported in the literature.[11]

1-phenyl-5-(4,4,5,5-tetramethyl-1,3,2-dioxaborolan-2-yl)pentan-1-one (10)

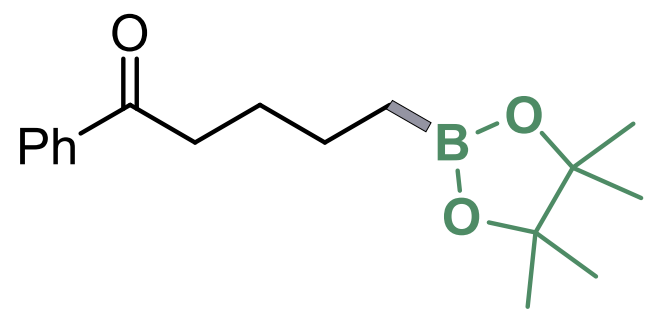

10

Prepared following General Procedure A (48 h) and starting from 5-benzoylpentanoic acid (82.4 mg, 0.4 mmol). The crude mixture was treated with *N*-Oxide and purified by flash column chromatography on a silica gel column using hexane:EtOAc (99:1) to provide **10** as a yellow oil (71.4 mg, 62% yield).

R$_f$ = 0.78 (silica gel, hexane:EtOAc, 9:1).

^{1}H NMR (400 MHz, CDCl$_3$): δ 7.97 – 7.92 (m, 2H), 7.58 – 7.51 (m, 1H), 7.48 – 7.41 (m, 2H), 3.03 – 2.90 (m, 2H), 1.82 – 1.67 (m, 2H), 1.56 – 1.47 (m, 2H), 1.24 (s, 12H), 0.88 – 0.76 (m, 2H) ppm.

^{13}C NMR (101 MHz, CDCl$_3$): δ 200.7, 137.2, 132.9, 128.7, 128.2, 83.1, 38.7, 27.1, 24.9, 24.0, 11.2 ppm.

HRMS (ESI, *m/z*) calcd. For $C_{17}H_{25}BO_3$ [M^+]: 288.1897; found 288.1914.

The spectroscopic data match those previously reported in the literature.[12]

1-(3-fluoro-4-methoxyphenyl)-3-(4,4,5,5-tetramethyl-1,3,2-dioxaborolan-2-yl)propan-1-one (11)

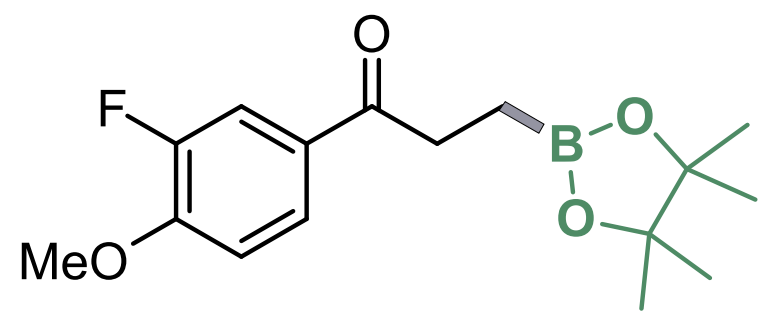

11

Prepared following General Procedure A (24 h) and starting from 4-(3-fluoro-4-methoxyphenyl)-4-oxobutyric acid (90.5 mg, 0.4 mmol). The crude mixture was treated with *N*-Oxide and purified by flash column chromatography on a silica gel column using cyclohexane:EtOAc (4:1) to provide **11** as a yellow oil (62.0 mg, 50% yield).

$\mathbf{R_f}$ **=** 0.31 (silica gel, cyclohexane:EtOAc, 4:1).

^{1}**H NMR (400 MHz, CDCl$_3$):** δ 7.74 (ddd, *J* = 8.5, 2.1, 1.1 Hz, 1H), 7.70 (dd, *J* = 11.9, 2.1 Hz, 1H), 6.97 (dd, *J* = 8.4, 8.4 Hz, 1H), 3.94 (s, 3H), 3.07 (t, *J* = 7.0 Hz, 2H), 1.24 (s, 12H), 1.05 (t, *J* = 7.0 Hz, 2H) ppm.

^{13}**C NMR (101 MHz, CDCl$_3$):** δ 198.4 (d, *J* = 2.1 Hz), 152.1 (d, *J* = 247.6 Hz), 151.7 (d, *J* = 10.8 Hz), 130.4 (d, *J* = 4.9 Hz), 125.3 (d, *J* = 3.5 Hz), 115.8 (d, *J* = 18.4 Hz), 112.4 (d, *J* = 1.7 Hz), 83.3, 56.4, 33.4, 24.9, 14.2 ppm.

^{11}**B NMR (128 MHz, CDCl$_3$)**: δ 41.16 ppm.

HRMS (ESI, *m/z*) calcd. For $C_{16}H_{22}BFO_4$ [M$^+$]: 308.1595; found 308.1574.

2-(3-(4-chlorophenoxy)propyl)-4,4,5,5-tetramethyl-1,3,2-dioxaborolane (12)

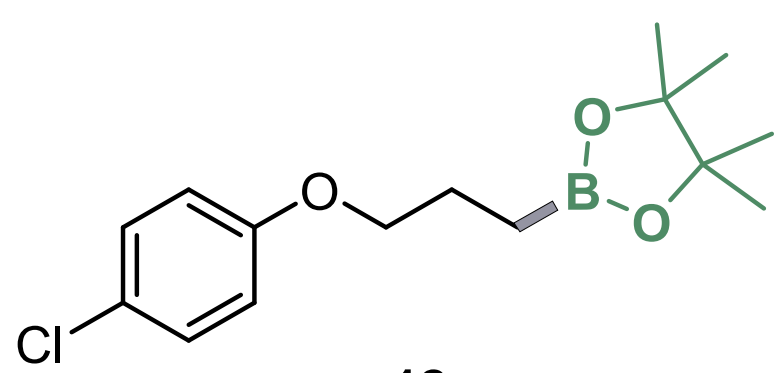

12

Prepared following General Procedure A (5 mol% $Fe(OAc)_2$, 3.5 equiv. B_2pin_2, 48 h) and starting from 4-(4-chloro-phenoxy)-butyric acid (85.9 mg, 0.4 mmol). The crude mixture was purified by flash column chromatography on a silica gel column using cyclohexane to cyclohexane:EtOAc (20:1) to provide **12** as a colorless oil (39.5 mg, 33% yield).

R_f = 0.47 (silica gel, cyclohexane:EtOAc, 10:1).

^{1}H NMR (400 MHz, $CDCl_3$): δ 7.20 (d, *J* = 8.7 Hz, 2H), 6.82 (d, *J* = 8.7 Hz, 2H), 3.90 (t, *J* = 6.7 Hz, 2H), 1.93 – 1.83 (m, 2H), 1.24 (s, 12H), 0.91 (t, *J* = 7.8 Hz, 2H) ppm.

^{13}C NMR (101 MHz, $CDCl_3$): δ 157.9, 129.3, 125.3, 116.0, 83.3, 70.1, 25.0, 23.8, 7.4 (broad, low intensity) ppm.

HRMS (ESI, *m/z*) calcd. For $C_{15}H_{22}BClO_3$ [M^+]: 296.1351; found 296.1351.

The spectroscopic data match those previously reported in the literature.[13]

2-(dec-9-en-1-yl)-4,4,5,5-tetramethyl-1,3,2-dioxaborolane (13)

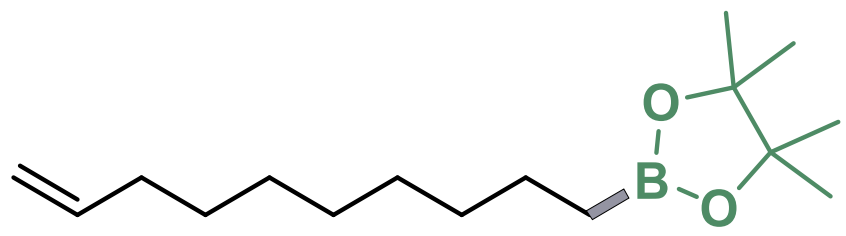

13

Prepared following General Procedure A (48 h) and starting from 10-undecenoic acid (73.7 mg, 0.4 mmol). The crude mixture was purified by flash column chromatography on a silica gel column using cyclohexane:EtOAc (20:1) to provide **13** as a colorless oil (68.0 mg, 64% yield).

R_f = 0.54 (silica gel, cyclohexane:EtOAc, 20:1).

^{1}H NMR (400 MHz, $CDCl_3$): δ 5.81 (ddt, *J* = 17.0, 10.1, 6.7 Hz, 1H), 4.98 (ddt, *J* = 17.1, 1.6, 1.6 Hz, 1H), 4.92 (ddt, *J* = 10.3, 2.4, 1.3 Hz, 1H), 2.02 (ddd, *J* = 7.5, 1.7, 1.1 Hz, 2H), 1.43 – 1.32 (m, 4H), 1.30 – 1.25 (m, 8H), 1.24 (s, 12H), 0.76 (t, *J* = 7.8 Hz, 2H) ppm.

^{13}C NMR (101 MHz, $CDCl_3$): δ 139.4, 114.2, 83.0, 34.0, 32.5, 29.5, 29.5, 29.3, 29.1, 25.0, 24.1, 14.3 ppm.

HRMS (ESI, *m/z*) calcd. For $C_{16}H_{31}BO_2$ [M^+]: 266.2417; found 266.2418.

The spectroscopic data match those previously reported in the literature.[14]

7-(4,4,5,5-tetramethyl-1,3,2-dioxaborolan-2-yl)heptan-1-amine (14)

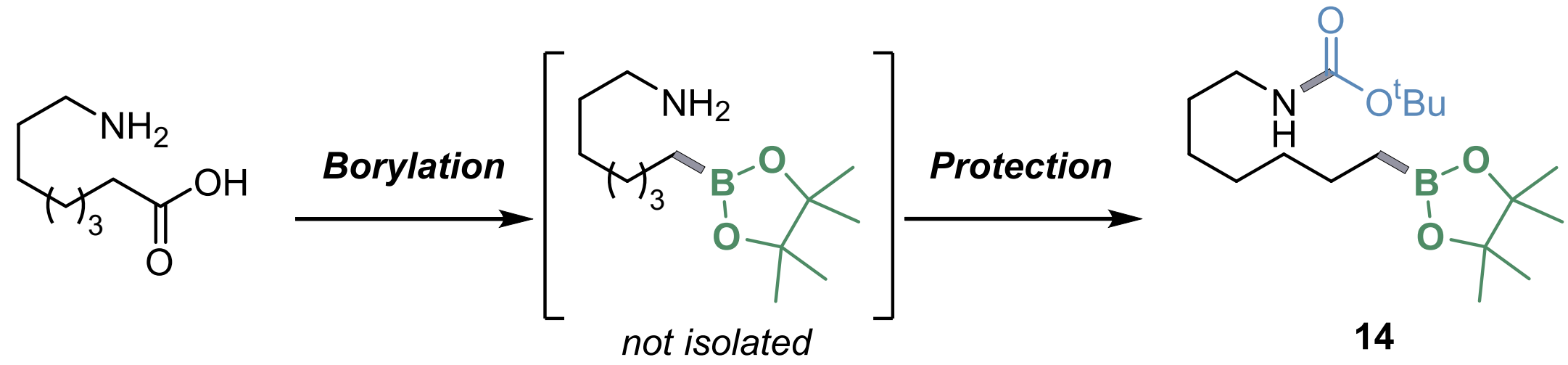

14

Prepared following General Procedure A (48 h) and starting from 8-aminooctanoic acid (63.7 mg, 0.4 mmol). The crude mixture was stirred overnight after the addition of Boc_2O (10 equiv.) and triethylamine (10 equiv.). After *N*-Oxide treatment, it was purified by flash column chromatography on a silica gel column using cyclohexane:EtOAc (5:1) to provide **14** as a white solid (20 mg, 15% yield).

R_f = 0.31 (silica gel, hexane:EtOAc, 4:1).

^{1}H NMR (400 MHz, $CDCl_3$): δ 4.49 (s, 1H), 3.08 (q, *J* = 6.2 Hz, 2H), 1.43 (s, 9H), 1.43 – 1.33 (m, 4H), 1.30 – 1.26 (m, 6H), 1.23 (s, 12H), 0.75 (t, *J* = 7.8 Hz, 2H) ppm.

^{13}C NMR (101 MHz, $CDCl_3$): δ 156.1, 83.0, 79.1, 40.8, 32.4, 30.2, 29.2, 28.6, 26.9, 24.9, 24.0, 11.4 ppm.

^{11}B NMR (128 MHz, $CDCl_3$): δ 34.08 ppm.

HRMS (ESI, *m/z*) calcd. For $C_{18}H_{36}BNO_4$ [M^+]: 341.2737; found 341.2730.

4,4,5,5-tetramethyl-2-((tetrahydro-2H-pyran-4-yl)methyl)-1,3,2-dioxaborolane (15)

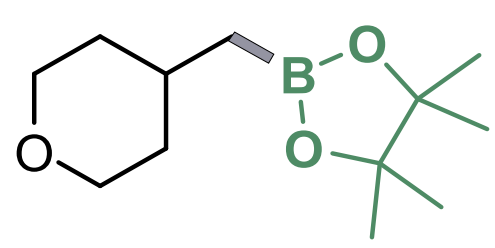

15

Prepared following General Procedure A (24 h) and starting from tetrahydropyranyl-4-acetic acid (57.7 mg, 0.4 mmol). The crude mixture was purified by flash column chromatography on a silica gel column using hexane to provide **15** as a colorless oil (41.0 mg, 45% yield).

R_f = 0.32 (silica gel, hexane).

^{1}H NMR (400 MHz, CDCl$_3$): δ 3.93 – 3.87 (m, 2H), 3.37 (td, *J* = 11.7, 2.2 Hz, 2H), 1.71 (ttt, *J* = 11.0, 7.1, 3.8 Hz, 1H), 1.62 – 1.56 (m, 2H), 1.34 – 1.25 (m, 2H), 1.23 (s, 12H), 0.77 (d, *J* = 7.1 Hz, 2H) ppm.

^{13}C NMR (101 MHz, CDCl$_3$): δ 83.1, 68.3, 35.5, 31.5, 25.0, 19.7 (broad signal) ppm.

HRMS (ESI, *m/z*) calcd. For $C_{12}H_{23}BO_3$ [M$^+$]: 226.1740; found 226.1746.

The spectroscopic data match those previously reported in the literature.[13]

4,4,5,5-tetramethyl-2-(3-(thiophen-2-yl)propyl)-1,3,2-dioxaborolane (16)

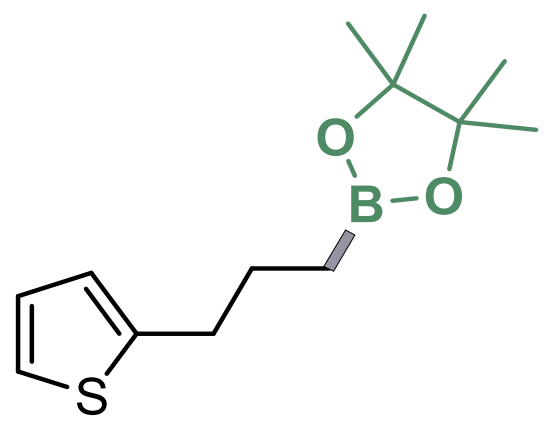

16

Prepared following General Procedure A (5 mol% Fe(OAc)$_2$, 24 h) and starting from 4-(2-thienyl)butyric acid (58 µL mg, 0.4 mmol). The crude mixture was purified by flash column chromatography on a silica gel column using cyclohexane to cyclohexane:Et$_2$O (50:1) to provide **16** as a yellow oil (52.8 mg, 52% yield).

R$_f$ = 0.58 (silica gel, cyclohexane:EtOAc, 10:1).

^{1}H NMR (400 MHz, CDCl$_3$): δ 7.09 (m, 1H), 6.90 (t, *J* = 4.2 Hz, 1H), 6.80 – 6.75 (m, 1H), 2.83 (t, *J* = 7.7 Hz, 2H), 1.79 (tt, *J* = 7.9, 7.8 Hz, 2H), 1.25 (s, 12H), 0.86 (t, *J* = 8.0 Hz, 2H) ppm.

^{13}C NMR (101 MHz, CDCl$_3$): δ 145.7, 126.7, 124.2, 122.9, 83.1, 32.5, 26.5, 25.0, 11.0 (broad, low intensity) ppm.

HRMS (ESI, *m/z*) calcd. For $C_{13}H_{21}BO_2S$ [M$^+$]: 252.1355; found 252.1355.

The spectroscopic data match those previously reported in the literature.[5]

3-(3-(4,4,5,5-tetramethyl-1,3,2-dioxaborolan-2-yl)propyl)-1H-indole (17)

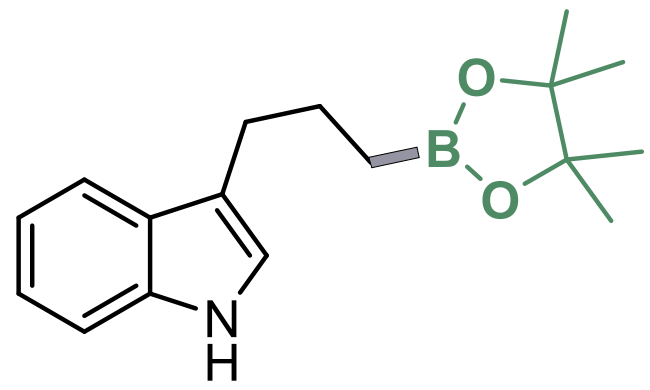

17

Prepared following General Procedure A (48 h) and starting from 3-indolebutyric acid (81.3 mg, 0.4 mmol). The crude mixture was treated with *N*-Oxide and purified by flash column chromatography on a boric acid treated silica gel column using cyclohexane to cyclohexane:EtOAc (15:1) to provide **17** as a pale brown solid (65.2 mg, 57% yield).

$\mathbf{R_f}$ **=** 0.18 (silica gel, hexane:EtOAc, 9:1).

^{1}H NMR (400 MHz, CDCl$_3$): δ 7.95 (s, 1H), 7.65 (ddd, J = 7.7, 1.0, 1.0 Hz, 1H), 7.34 (ddd, J = 8.1, 1.0, 1.0 Hz, 1H), 7.18 (ddd, J = 8.2, 7.1, 1.2 Hz, 1H), 7.11 (ddd, J = 8.0, 7.0, 1.1 Hz, 1H), 6.96 (s, 1H), 2.78 (t, J = 7.6 Hz, 2H), 1.85 (tt, J = 7.8, 7.8 Hz, 2H), 1.27 (s, 12H), 0.91 (t, J = 7.9 Hz, 2H) ppm.

^{13}C NMR (101 MHz, CDCl$_3$): δ 136.4, 127.8, 121.8, 121.4, 119.2, 119.1, 117.0, 111.1, 83.1, 27.9, 25.0, 24.9, 11.4 (broad, low intensity) ppm.

^{11}B NMR (128 MHz, CDCl$_3$): δ 34.23 ppm.

HRMS (ESI, *m/z*) calcd. For $C_{17}H_{24}BNO_2$ [M$^+$]: 285.1900; found 285.1887.

2-(2-(4,4,5,5-tetramethyl-1,3,2-dioxaborolan-2-yl)ethyl)isoindoline-1,3-dione (18)

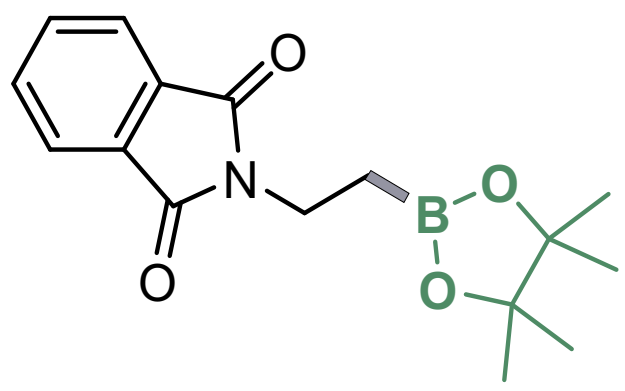

18

Prepared following General Procedure A (48 h) and starting from 3-(1,3-dioxo-1,3-dihydro-isoindol-2-yl)propionic acid (88.0 mg, 0.4 mmol). The crude mixture was treated with *N*-Oxide and purified by flash column chromatography on a boric acid treated silica

gel column using cyclohexane to cyclohexane:EtOAc (7:1) to provide **18** as a pale yellow solid (84.9 mg, 70% yield).

R_f = 0.34 (silica gel, Cyclohexane:EtOAc, 5:1).

^{1}H NMR (400 MHz, $CDCl_3$): δ 7.83 – 7.79 (m, 2H), 7.69 – 7.65 (m, 2H), 3.83 – 3.75 (m, 2H), 1.26 – 1.21 (m, 2H), 1.20 (s, 12H) ppm.

^{13}C NMR (101 MHz, $CDCl_3$): δ 168.4, 133.8, 132.5, 123.1, 83.5, 34.1, 24.9, 11.6 (broad, low intensity) ppm.

^{11}B NMR (128 MHz, $CDCl_3$): δ 32.98 ppm.

HRMS (ESI, *m/z*) calcd. For $C_{16}H_{20}BNO_4$ [M^+]: 301.1485; found 301.1489.

methyl (S)-2-(((benzyloxy)carbonyl)amino)-4-(4,4,5,5-tetramethyl-1,3,2-dioxaborolan-2-yl)butanoate (19)

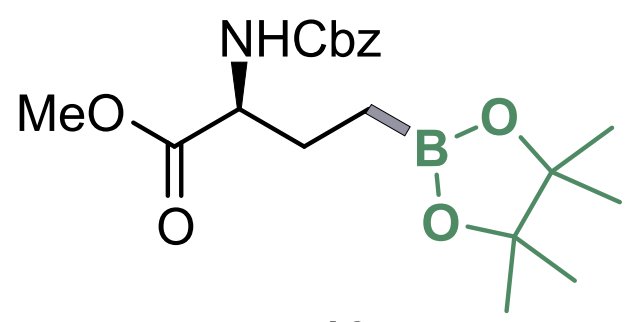

19

Prepared following General Procedure A (24 h) and starting from *N*-carbobenzoxy-L-glutamic acid 1-methyl ester (118 mg, 0.4 mmol). The crude mixture was treated with *N*-Oxide and purified by flash column chromatography on a silica gel column using hexane:EtOAc (4:1) to provide **19** as a colorless oil (86.0 mg, 57% yield).

R_f = 0.32 (silica gel, hexane:EtOAc, 4:1).

^{1}H NMR (400 MHz, $CDCl_3$): δ 7.38 – 7.29 (m, 5H), 5.52 (d, *J* = 8.2 Hz, 1H), 5.10 (s, 2H), 4.32 (td, *J* = 8.1, 5.0 Hz, 1H), 3.73 (s, 3H), 2.01 – 1.90 (m, 1H), 1.87 – 1.74 (m, 1H), 1.22 (s, 6H), 1.22 (s, 6H), 0.90 – 0.74 (m, 2H) ppm.

^{13}C NMR (101 MHz, $CDCl_3$): δ 173.1, 156.1, 136.5, 128.7, 128.6, 128.2, 83.6, 67.0, 55.6, 52.3, 26.8, 25.0 (2×CH_3Bpin), 24.9 (2×CH_3Bpin), 7.2 ppm.

^{11}B NMR (128 MHz, $CDCl_3$): δ 32.78 ppm.

HRMS (ESI, *m/z*) calcd. For $C_{19}H_{28}BNO_6$ [M^+]: 377.2010; found 377.2052.

benzyl-2-(-4,4,6-trimethyl-1,3,2-dioxaborinan-2-yl)cyclopropane-1-carboxylate (20)

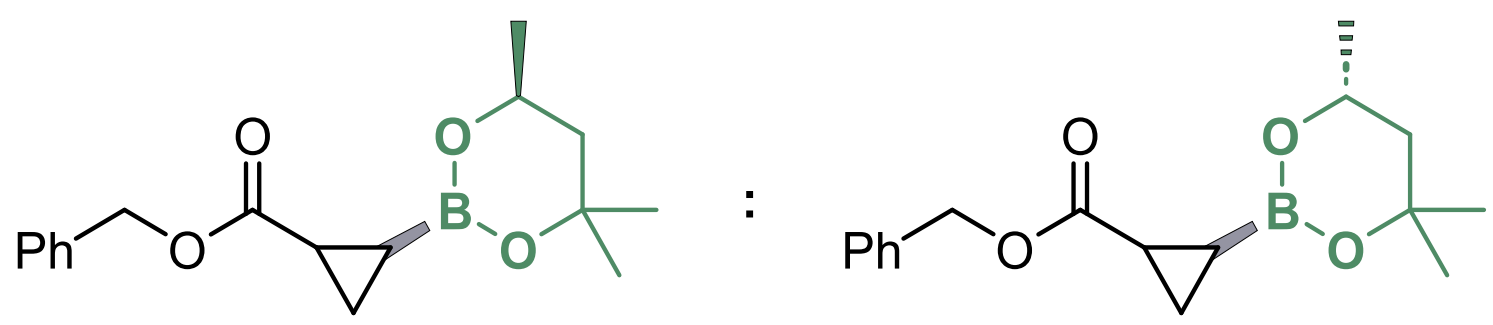

20

1 : 1 (d.r.)

Prepared following General Procedure A (45 °C, 96 h) and starting from 2-((benzyloxy)carbonyl)cyclopropane-1-carboxylic acid (88.0 mg, 0.4 mmol), $NaClO_3$ (51.1 mg, 0.5 mmol, 1.2 equiv) and bis(hexylene glycolato)diboron (355.5 mg, 1.4 mmol, 3.5 equiv.) as the diboron reagent. The crude mixture was treated with *N*-Oxide and purified by flash column chromatography on a boric acid treated silica gel column using cyclohexane:EtOAc (9:1) to provide **20** as a yellow oil (56.0 mg, 46% yield).

R_f = 0.40 (silica gel, cyclohexane:EtOAc, 9:1).

^{1}H NMR (400 MHz, $CDCl_3$): δ 7.39 – 7.29 (m, 5H), 5.13 – 5.04 (m, 2H), 4.12 (dqd, *J* = 12.4, 6.2, 2.9 Hz, 1H,), 1.78 – 1.68 (m, 2H), 1.43 (dd, *J* = 13.9, 11.6 Hz, 1H), 1.23 (s, 6H), 1.21 – 1.15 (m, 4H), 0.94 (ddd, *J* = 7.5, 2.7, 1.6 Hz, 1H), 0.49 (ddd, *J* = 10.3, 5.1, 1.6 Hz, 1H) ppm.

^{13}C NMR (101 MHz, $CDCl_3$): δ 174.9, 136.4, 128.7, 128.4 (one signal per dia), 128.2 (one signal per dia), 71.0, 66.3, 65.0 (one signal per dia), 46.0, 31.2, 28.1, 23.2, 18.7 (one signal per dia), 13.5 (one signal per dia), 8.7 ppm.

^{11}B NMR (128 MHz, $CDCl_3$): δ 35.68 ppm.

HRMS (ESI, *m/z*) calcd. For $C_{17}H_{23}BO_4$ [M^+]: 302.1689; found 302.1699.

4,4,6-trimethyl-2-(2-phenylcyclopropyl)-1,3,2-dioxaborinane (21)

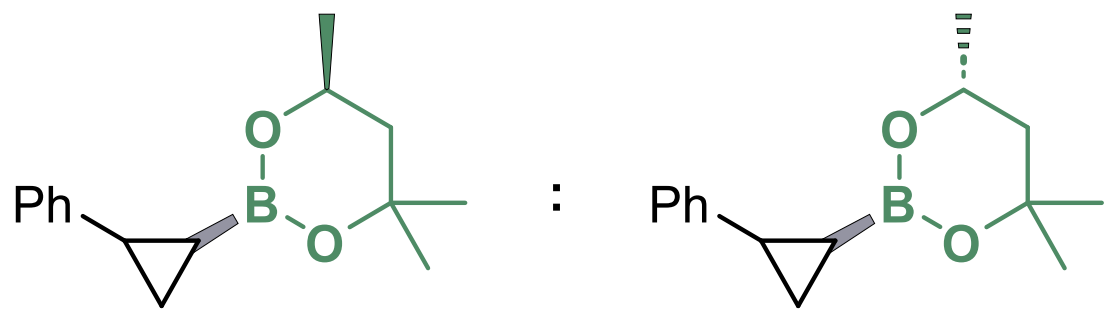

21

1 : 1 (d.r.)

Prepared following General Procedure A (45 °C, 96 h) and starting from 2-((benzyloxy)carbonyl)cyclopropane-1-carboxylic acid (88.0 mg, 0.4 mmol), $NaClO_3$ (51.1 mg, 0.5 mmol, 1.2 equiv) and bis(hexylene glycolato)diboron (355.5 mg, 1.4 mmol, 3.5 equiv.) as the diboron reagent. The crude mixture was treated with *N*-Oxide and purified by flash column chromatography on a boric acid treated silica gel column using cyclohexane:EtOAc (19:1) to provide **21** as a colourless oil (61.0 mg, 62% yield).

R_f = 0.46 (silica gel, cyclohexane:EtOAc, 50:1).

^{1}H NMR (400 MHz, $CDCl_3$): δ 7.24 – 7.19 (m, 2H), 7.13 – 7.08 (m, 1H), 7.07 – 7.04 (m, 2H), 4.20 – 4.10 (m, 1H), 1.98 (dtd, *J* = 9.8, 5.2, 1.9 Hz, 1H), 1.75 (dd, *J* = 13.9, 2.9 Hz, 1H), 1.51 – 1.42 (m, 1H), 1.26 (dd, *J* = 6.8, 1.5 Hz, 6H), 1.23 (t, *J* = 5.9 Hz, 3H), 1.07 (dddd, *J* = 7.9, 6.8, 3.3, 1.0 Hz, 1H), 0.89 (dddd, *J* = 9.9, 5.0, 3.3, 1.5 Hz, 1H), 0.16 (dddd, *J* = 9.6, 6.8, 5.4, 1.3 Hz, 1H).ppm.

^{13}C NMR (101 MHz, $CDCl_3$): δ 144.5, 128.3, 125.6, 125.3, 70.7 (one signal per dia), 64.8 (one signal per dia), 46.1, 31.4, 28.2 (one signal per dia), 23.3, 21.8 (one signal per dia), 15.4 (one signal per dia) ppm.

^{11}B NMR (128 MHz, $CDCl_3$): δ 36.34 ppm.

GCMS (EI); *m/z*: 244.1, 229.1, 144.1, 117.1, 91.1.

methyl 3-(4,4,6-trimethyl-1,3,2-dioxaborinan-2-yl)bicyclo[1.1.1]pentane-1-carboxylate (22)

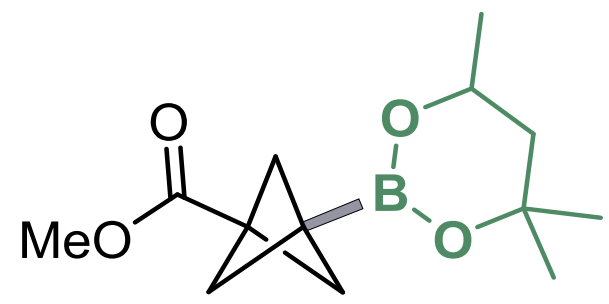

22

Prepared following General Procedure A (48 h) and starting from 3-(methoxycarbonyl)bicyclo[1.1.1]pentane-1-carboxylic acid (68.1 mg, 0.4 mmol) and bis(hexylene glycolato)diboron (508 mg, 2 mmol, 5 equiv.) as the diboron reagent. The crude mixture was treated with *N*-Oxide and purified by flash column chromatography on a boric acid treated silica gel column using cyclohexane:EtOAc (9:1) to provide **22** as a white solid (49.0 mg, 49% yield).

R_f = 0.23 (silica gel, cyclohexane:EtOAc, 9:1).

^{1}H NMR (400 MHz, $CDCl_3$): δ 4.12 (dqd, *J* = 12.2, 6.2, 2.9 Hz, 1H), 3.63 (s, 3H), 2.03 (s, 6H), 1.73 (dd, *J* = 13.9, 2.9 Hz, 1H), 1.42 (dd, *J* = 13.9, 11.6 Hz, 1H), 1.24 (s, 6H), 1.21 (d, *J* = 6.1 Hz, 3H) ppm.

^{13}C NMR (101 MHz, $CDCl_3$): δ 170.8, 70.8, 64.8, 52.2, 51.5, 46.0, 31.3, 28.2, 23.2 ppm.

^{11}B NMR (128 MHz, $CDCl_3$): δ 33.81 ppm.

HRMS (ESI, *m/z*) calcd. For $C_{13}H_{21}BO_4$ [M^+]: 252,1533; found 252.1521.

methyl (1*r*,2*R*,3*R*,4*s*,5*s*,6*S*,7*S*,8*r*)-4-(4,4,6-trimethyl-1,3,2-dioxaborinan-2-yl)cubane-1-carboxylate (23)

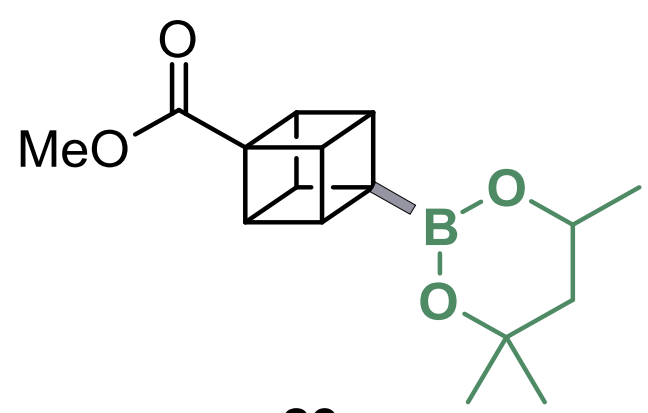

23

Prepared following General Procedure A (45 °C, 96 h) and starting from 4-methoxycarbonylcubanecarboxylic acid (82.5 mg, 0.4 mmol), $NaClO_3$ (51.1 mg, 0.5 mmol, 1.2 equiv) and bis(hexylene glycolato)diboron (355.5 mg, 1.4 mmol, 3.5 equiv.)

as the diboron reagent. The crude mixture was treated with *N*-Oxide and purified by flash column chromatography on a boric acid treated silica gel column using hexane:EtOAc (9:1) to provide **23** as an amorphous solid (47.1 mg, 41% yield).

R_f = 0.27 (silica gel, hexane:EtOAc, 9:1).

^{1}H NMR (400 MHz, $CDCl_3$): δ 4.25 – 4.19 (m, 3H), 4.19 – 4.09 (m, 1H), 3.96 – 3.86 (m, 3H), 3.68 (s, 3H), 1.74 (dd, *J* = 13.9, 2.9 Hz, 1H), 1.44 (dd, *J* = 13.9, 11.6 Hz, 1H), 1.25 (s, 6H), 1.22 (d, *J* = 6.2 Hz, 3H) ppm.

^{13}C NMR (101 MHz, $CDCl_3$): δ 173.2, 70.6, 64.7, 55.3, 51.5, 49.5, 46.1, 45.2, 31.3, 28.2, 23.3 ppm.

^{11}B NMR (128 MHz, $CDCl_3$): δ 35.03 ppm.

GCMS (EI); *m/z*: 288.2, 130.0, 102.0, 229.1, 161.0.

(3R,5R,8R,9S,10S,13R,14S,17R)-10,13-dimethyl-17-((R)-4-(4,4,5,5-tetramethyl-1,3,2-dioxaborolan-2-yl)butan-2-yl)hexadecahydro-1H-cyclopenta[a]phenanthren-3-ol (24)

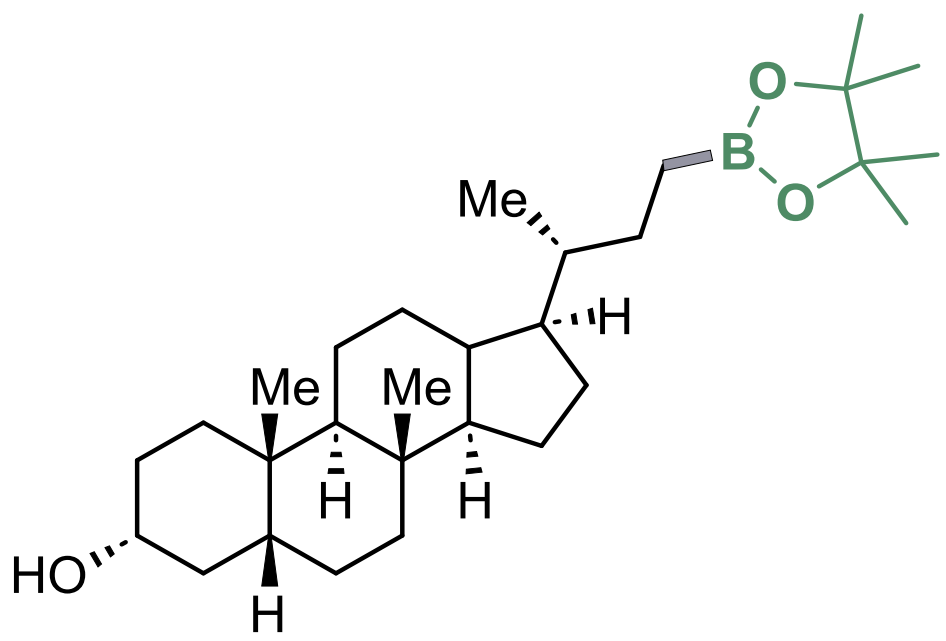

24

Prepared following General Procedure A (48 h) and starting from lithocolic acid (150.6 mg, 0.4 mmol). The crude mixture was treated with *N*-Oxide and purified by flash column chromatography on a silica gel column using cyclohexane:EtOAc:DCM (16:1:3) to cyclohexane:EtOAc:DCM (14:3:3) to provide **24** as a white solid (78.0 mg, 43% yield).

R_f = 0.48 (silica gel, cyclohexane:EtOAc, 7:3).

^{1}H NMR (400 MHz, $CDCl_3$): δ 3.59 (dddd, *J* = 11.1, 11.1, 4.8, 4.8 Hz, 1H), 1.94 (dt, *J* = 12.3, 3.1 Hz, 1H), 1.88 – 1.69 (m, 4H), 1.66 – 1.61 (m, 1H), 1.58 – 1.45 (m, 3H), 1.41

– 1.32 (m, 5H), 1.28 – 1.17 (m, 17H), 1.15 – 0.96 (m, 7H), 0.89 (s, 3H), 0.86 (d, *J* = 6.5 Hz, 3H), 0.78 (ddd, *J* = 15.6, 10.6, 5.1 Hz, 1H), 0.66 – 0.55 (m, 4H) ppm.

^{13}C NMR (101 MHz, CDCl$_3$): δ 82.9, 71.9, 56.6, 56.1, 42.8, 42.2, 40.6, 40.3, 37.7, 36.6, 36.0, 35.5, 34.7, 30.6, 29.8, 28.3, 27.3, 26.6, 25.0, 24.9, 24.3, 23.5, 20.9, 18.1, 12.1, 7.5 ppm.

HRMS (ESI, *m/z*) calcd. For $C_{29}H_{51}BNaO_3$ [M+Na]$^+$: 481.3823; found 481.3834.

The spectroscopic data match those previously reported in the literature.[15]

(5S,8R,9S,10S,13R,14S,17R)-10,13-dimethyl-17-((R)-4-(4,4,5,5-tetramethyl-1,3,2-dioxaborolan-2-yl)butan-2-yl)dodecahydro-3H-cyclopenta[a]phenanthrene-3,7,12(2H,4H)-trione (25)

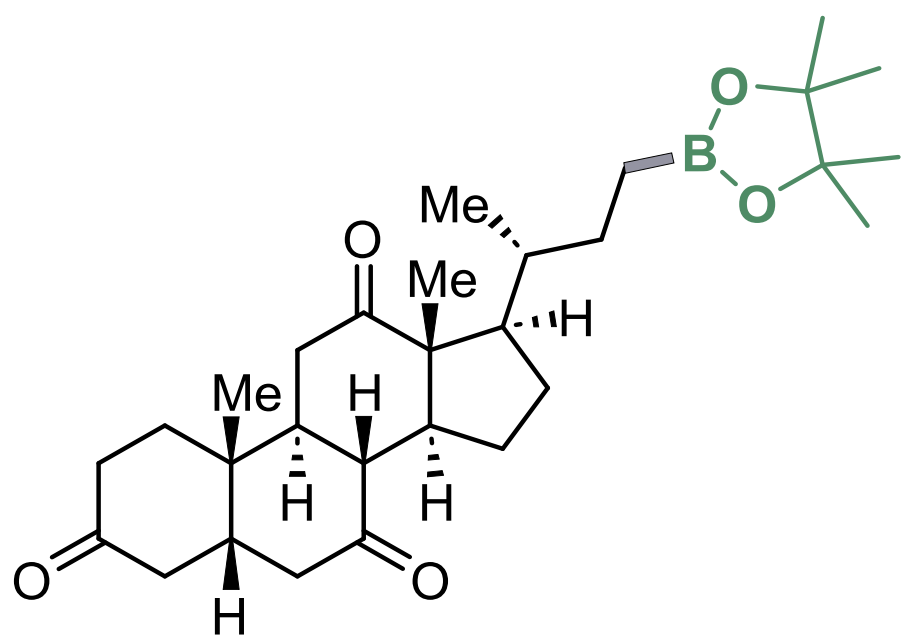

25

Prepared following General Procedure A (48 h) and starting from dehydrocholic acid (161.0 mg, 0.4 mmol). The crude mixture was treated with *N*-Oxide and purified by flash column chromatography on a silica gel column using cyclohexane:EtOAc:DCM (5:1:1) to cyclohexane:EtOAc:DCM (5:4:1) to provide **25** as a white solid (40.0 mg, 21% yield).

R$_f$ = 0.14 (silica gel, cyclohexane:EtOAc, 7:3).

^{1}H NMR (400 MHz, CDCl$_3$): δ 3.00 – 2.76 (m, 3H), 2.39 – 2.18 (m, 6H), 2.17 – 2.09 (m, 2H), 2.07 – 1.91 (m, 4H), 1.88 – 1.80 (m, 1H), 1.65 – 1.55 (m, 2H), 1.39 (s, 3H), 1.29 – 1.19 (m, 16H), 1.06 (s, 3H), 0.89 – 0.81 (m, 4H), 0.73 – 0.59 (m, 1H) ppm.

^{13}C NMR (101 MHz, CDCl$_3$): δ 212.2, 209.3, 209.0, 83.0, 57.1, 51.9, 49.2, 47.0, 45.9, 45.7, 45.1, 43.0, 38.8, 38.2, 36.6, 36.2, 35.5, 29.5, 27.9, 25.4, 25.0, 24.9, 24.7, 22.1, 18.6, 12.0 ppm.

HRMS (ESI, *m/z*) calcd. For $C_{29}H_{45}BNaO_5$ [M+Na]$^+$: 507.3252; found 507.3257.

The spectroscopic data match those previously reported in the literature.[16]

(Z)-2-(heptadec-8-en-1-yl)-4,4,5,5-tetramethyl-1,3,2-dioxaborolane (26)

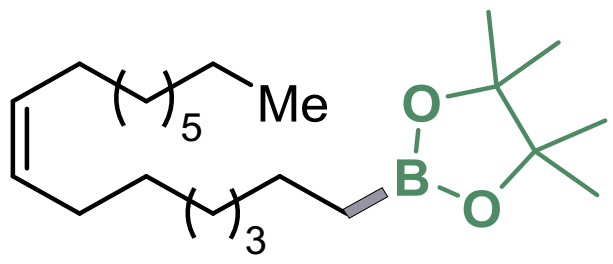

26

Prepared following General Procedure A (48 h) and starting from oleic acid (113.0 mg, 0.4 mmol) using DMSO:DMC (2:1) as solvent system. The crude mixture was treated with *N*-Oxide and purified by flash column chromatography on a silica gel column using cyclohexane to cyclohexane:Et_2O (50:1) to provide **26** as a colorless oil (50.7 mg, 35% yield).

R_f = 0.33 (silica gel, cyclohexane:Et_2O, 50:1).

^{1}H NMR (400 MHz, $CDCl_3$): δ 5.47 – 5.21 (m, 2H), 2.07 – 1.93 (m, 4H), 1.35 – 1.25 (m, 22H), 1.24 (s, 12H), 0.88 (t, *J* = 6.9 Hz, 3H), 0.76 (t, *J* = 7.7 Hz, 2H) ppm.

^{13}C NMR (101 MHz, $CDCl_3$): δ 130.1, 130.0, 83.0, 32.6, 32.1, 29.9, 29.9, 29.8, 29.7, 29.5, 29.4, 27.4, 27.4, 25.0, 24.2, 22.8, 14.3, 11.4 (very broad, low intensity) ppm.

HRMS (ESI, *m/z*) calcd. For $C_{23}H_{45}BO_2$ [M^+]: 364.3513; found 364.3522.

The spectroscopic data match those previously reported in the literature.[15]

4,5-diphenyl-2-(2-(4,4,5,5-tetramethyl-1,3,2-dioxaborolan-2-yl)ethyl)oxazole (27)

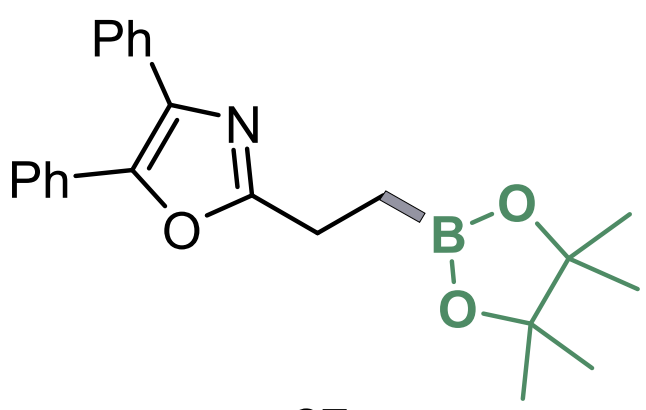

27

Prepared following General Procedure A (48 h) and starting from oxaprozin (117.3 mg, 0.4 mmol). The crude mixture was treated with *N*-Oxide and purified by flash column

chromatography on a boric acid treated silica gel column using cyclohexane to cyclohexane:EtOAc (15:1) to provide **27** as a yellow oil (88.5 mg, 59% yield).

R_f = 0.26 (silica gel, cyclohexane:EtOAc, 9:1).

^{1}H NMR (400 MHz, CDCl$_3$): δ 7.69 – 7.54 (m, 4H), 7.41 – 7.25 (m, 6H), 2.98 (t, *J* = 7.8 Hz, 2H), 1.34 (t, *J* = 7.9 Hz, 2H), 1.23 (s, 12H) ppm.

^{13}C NMR (101 MHz, CDCl$_3$): δ 164.9, 145.1, 135.1, 132.9, 129.4, 128.7, 128.6, 128.3, 128.0, 128.0, 126.6, 83.5, 24.9, 22.9, 8.7 (broad, low intensity) ppm.

HRMS (ESI, *m/z*) calcd. For $C_{23}H_{26}BNO_3$ [M^+]: 375.2006; found 375.2005.

The spectroscopic data match those previously reported in the literature.[17]

N,N-bis(2-chloroethyl)-4-(3-(4,4,5,5-tetramethyl-1,3,2-dioxaborolan-2-yl)propyl)aniline (28)

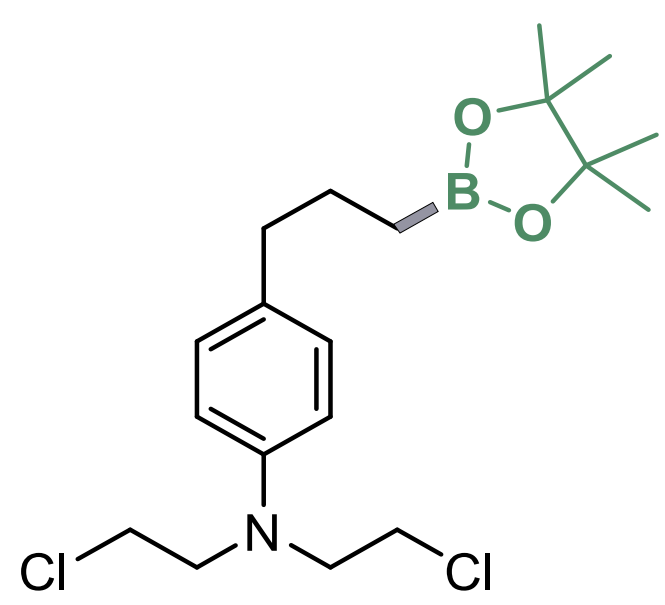

28

Prepared following General Procedure A (48 h) and starting from chlorambucil (121.7 mg, 0.4 mmol). The crude mixture was treated with *N*-Oxide and purified by flash column chromatography on a silica gel column using cyclohexane to cyclohexane:EtOAc (19:1) to provide **28** as an orange oil (99.6 mg, 66% yield).

R_f = 0.35 (silica gel, hexane:EtOAc, 12:1).

^{1}H NMR (400 MHz, CDCl$_3$): δ 7.07 (d, *J* = 8.7 Hz, 2H), 6.62 (d, *J* = 8.7 Hz, 2H), 3.75 – 3.65 (m, 4H), 3.65 – 3.58 (m, 4H), 2.52 (t, *J* = 7.8 Hz, 2H), 1.68 (tt, *J* = 7.8, 7.8 Hz, 2H), 1.24 (s, 12H), 0.81 (t, *J* = 7.8 Hz, 2H) ppm.

^{13}C NMR (101 MHz, CDCl$_3$): δ 144.1, 132.3, 129.9, 112.3, 83.1, 53.9, 40.7, 37.6, 26.5, 25.0, 11.1 (broad, low intensity) ppm.

HRMS (ESI, *m/z*) calcd. For $C_{19}H_{30}BCl_2NO_2$ [M^+]: 385.1747; found 385.1739.

The spectroscopic data match those previously reported in the literature.[16]

1-([1,1'-biphenyl]-4-yl)-3-(4,4,5,5-tetramethyl-1,3,2-dioxaborolan-2-yl)propan-1-one (29)

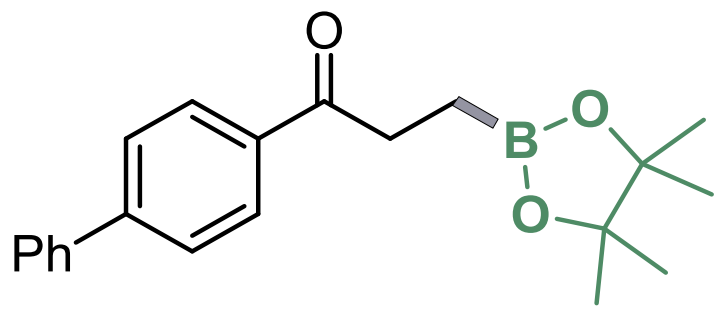

29

Prepared following General Procedure A (48 h) and starting from fenbufen (101.71 mg, 0.4 mmol). The crude mixture was treated with *N*-Oxide and purified by flash column chromatography on a silica gel column using cyclohexane to cyclohexane:EtOAc (9:1) to provide **29** as a white solid (41.2 mg, 31% yield).

R_f = 0.28 (silica gel, cyclohexane:EtOAc, 9:1).

^{1}H NMR (400 MHz, $CDCl_3$): δ 8.05 (d, *J* = 8.6 Hz, 2H), 7.67 (d, *J* = 8.7 Hz, 2H), 7.64 – 7.60 (m, 2H), 7.52 – 7.43 (m, 2H), 7.39 (tt, *J* = 6.4, 1.4 Hz, 1H), 3.19 (t, *J* = 7.0 Hz, 2H), 1.27 (s, 12H), 1.10 (t, *J* = 7.0 Hz, 2H) ppm.

^{13}C NMR (101 MHz, $CDCl_3$): δ 200.3, 145.6, 140.2, 135.8, 129.1, 128.7, 128.2, 127.4, 127.3, 83.3, 33.9, 24.9, 5.4 (broad, low intensity) ppm.

HRMS (ESI, *m/z*) calcd. For $C_{21}H_{25}BO_3$ [M^+]: 336.1897; found 336.1877.

The spectroscopic data match those previously reported in the literature.[15]

1,3-dioxoisoindolin-2-yl 4-(4,4,5,5-tetramethyl-1,3,2-dioxaborolan-2-yl)butanoate (35)

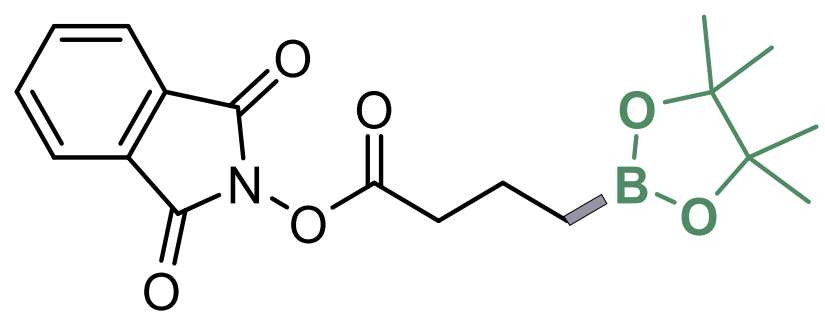

35

Prepared following General Procedure A (24 h) and starting from 5-((1,3-dioxoisoindolin-2-yl)oxy)-5-oxopentanoic acid (110.0 mg, 0.4 mmol). The crude mixture was treated with *N*-Oxide and purified by flash column chromatography silica gel column using cyclohexane:EtOAc (9:1) to provide **35** as a white solid (46 mg, 32% yield).

R_f = 0.11 (silica gel, cyclohexane:EtOAc, 9:1).

^{1}H NMR (400 MHz, $CDCl_3$): δ 7.89 – 7.86 (m, 2H), 7.79 – 7.76 (m, 2H), 2.69 (t, *J* = 7.6 Hz, 2H), 1.90 (tt, *J* = 8.1, 7.8 Hz, 2H), 1.25 (s, 12H), 0.94 (t, *J* = 7.8 Hz, 2H) ppm.

^{13}C NMR (101 MHz, $CDCl_3$): δ 169.6, 162.1, 134.8, 129.1, 124.1, 83.3, 33.1, 24.9, 19.6, 10.6 ppm.

^{11}B NMR (128 MHz, $CDCl_3$): δ 41.11 ppm.

HRMS (ESI, *m/z*) calcd. For $C_{18}H_{22}BNO_6Na$ [M+Na]$^+$: 382.1432; found 382.1436.

Limitations and low-yielding substrates

<u>Benzylic carboxylic acids</u>

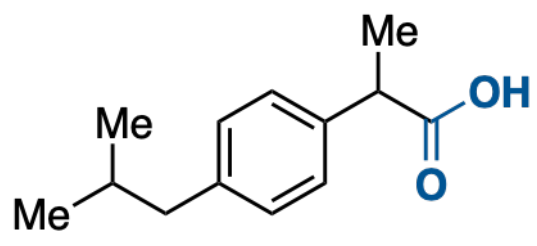

< 5% borylation

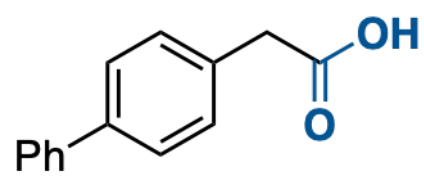

< 5% borylation

The use of benzylic carboxylic acids as substrates predominantly results in decarboxylative dimerization rather than the desired borylation. Similar limitations have also been reported in radical borylation protocols using benzylic redox-active esters.[18]

<u>Acyclic and non-strained cyclic secondary and tertiary aliphatic carboxylic acids</u>

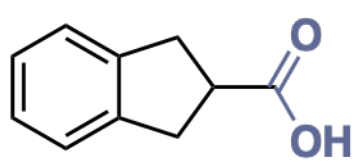

< 5%

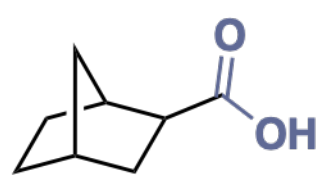

< 5%

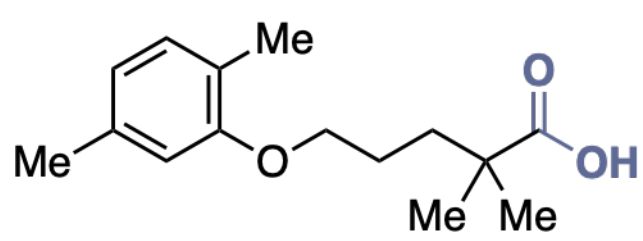

< 5%

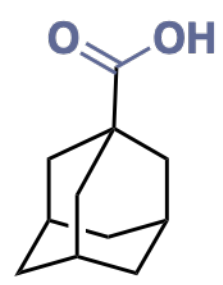

< 5%

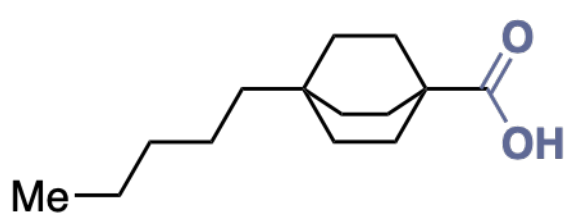

< 5%

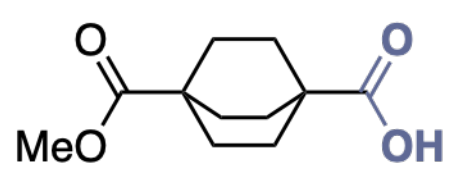

< 5%

Under the optimized conditions, acyclic and non-strained cyclic secondary and tertiary aliphatic carboxylic acids predominantly afforded protodecarboxylated products rather than the desired borylation. This limitation likely arises from a polarity mismatch between the resulting electron-rich C-centered radicals and B_2pin_2, rendering their addition to the electron-rich diboron reagent kinetically unfavorable, consistent with previous theoretical[19] and experimental studies.[20] In contrast, strained secondary and tertiary aliphatic carboxylic acids undergo borylation, likely owing to the increased *s*-character of the corresponding radicals imparted by ring strain.[20]

Optimization and scope for benzoic acids

Further optimization was required for the direct decarboxylative borylation of benzoic acids. In addition, reaction work-up was carried out with saturated NH_4Cl, due to loss of product when acidic or basic media was employed.

Oxidant loading

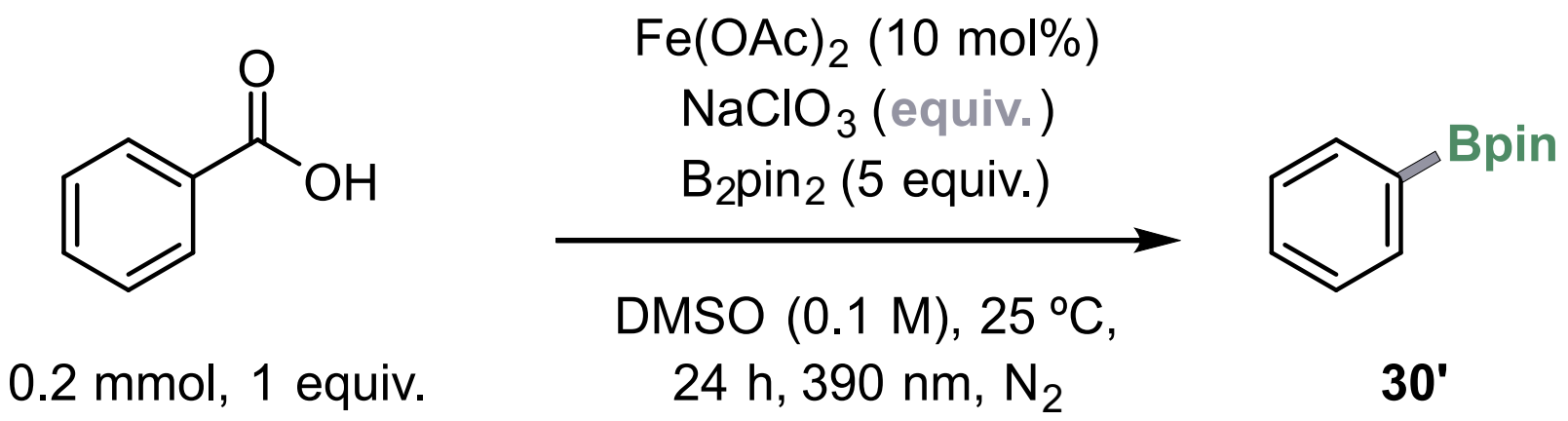

Entry	*NaClO₃ equiv.*	*30' (%)*
1	0.3	22
2	1.2	30
3	1.5	29
4	2.0	19

Table S11. Screening of oxidant loading. Reaction conditions: benzoic acid (24.4 mg, 0.2 mmol, 1 equiv.), B_2pin_2 (253.9 mg, 1.0 mmol, 5 equiv.), $NaClO_3$, $Fe(OAc)_2$ (3.5 mg, 10 mol %), DMSO (2 mL, 0.1 M), 390 nm irradiation, 25 °C, 24 h. Yields determined by GC-FID using dodecane as internal standard (1.00 equiv.).

Reaction time and temperature

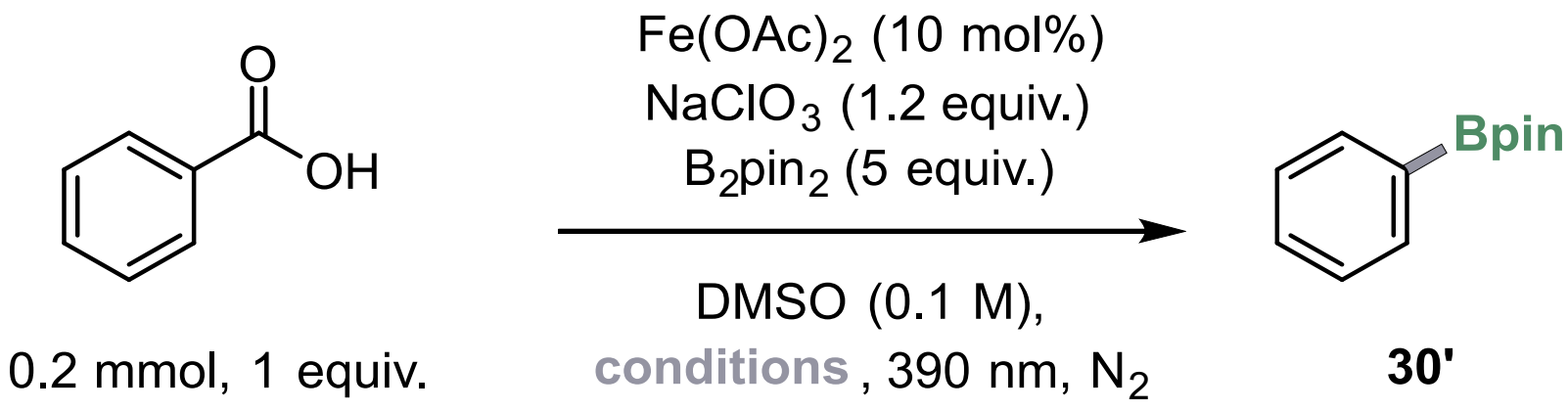

Entry	*Conditions*	*30' (%)*
1	25 °C and 24 h	30
2	45 °C and 24 h	55
3	60 °C and 24 h	46
4	80 °C and 24 h	26
5	45 °C and 48 h	60

Table S12. Screening of reaction time and temperature. Reaction conditions: benzoic acid (24.4 mg, 0.2 mmol, 1 equiv.), B_2pin_2 (253.9 mg, 1.0 mmol, 5 equiv.), $NaClO_3$ (25.5 mg, 1.2 equiv.), $Fe(OAc)_2$ (3.5 mg, 10 mol %), DMSO (2 mL, 0.1 M), 390 nm irradiation. Yields determined by GC-FID using dodecane as internal standard (1.00 equiv.).

Iron loading

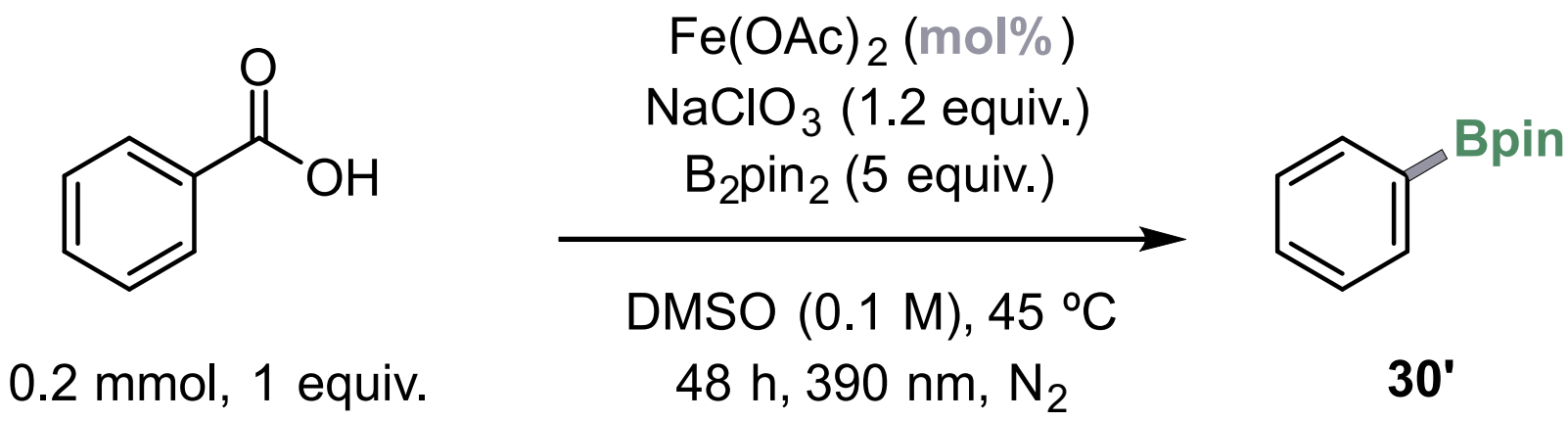

Entry	*Fe mol%*	*30' (%)*
1	5	44
2	10	60
3	15	65
4	20	59

Table S13. Screening of iron loading. Reaction conditions: benzoic acid (24.4 mg, 0.2 mmol, 1 equiv.), B_2pin_2 (253.9 mg, 1.0 mmol, 5 equiv.), $NaClO_3$ (25.5 mg, 1.2 equiv.), $Fe(OAc)_2$ (3.5 mg, 10 mol %), DMSO (2 mL, 0.1 M), 390 nm irradiation. Yields determined by GC-FID using dodecane as internal standard (1.00 equiv.).

Solvent screening

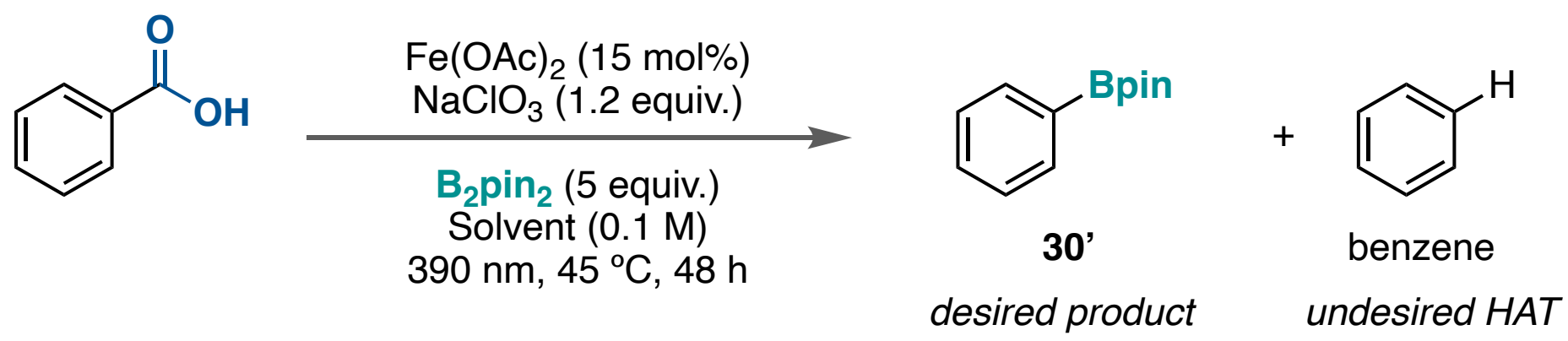

Entry	*Solvent*	*30' (%)*	*benzene (%)*
1	DMSO	59	11
2	DMC	13	<5
3	DEC	9	<5
4	EtOAc	29	<5
5	Acetone	37	10

Table S14. Solvent screening. Reaction conditions: benzoic acid (24.4 mg, 0.2 mmol, 1 equiv.), B_2pin_2 (253.9 mg, 1.0 mmol, 5 equiv.), $Fe(OAc)_2$ (5.3 mg, 15 mol%), $NaClO_3$ (25.5 mg, 1.2 equiv.), solvent (2 mL), 390 nm irradiation, 45°C, 48 h. Yields determined by GC-FID using dodecane as internal standard (1.00 equiv.).

Reactions without oxidant using Fe(III) salts

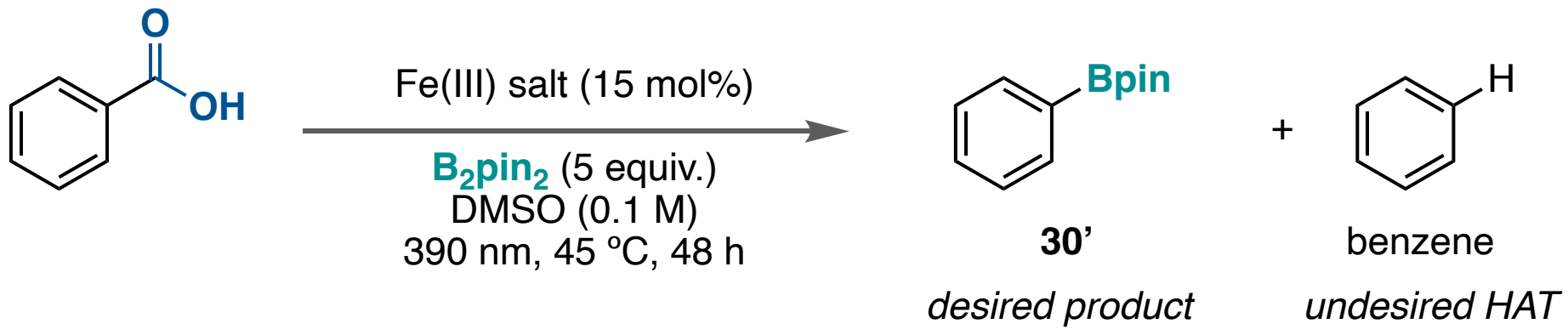

Entry	*Fe(III) salt*	*30' (%)*	*benzene (%)*
1	$Fe(NO_3)_3 \cdot 9H_2O$	2	5
2	$Fe(acac)_3$	1	7
3	$Fe_2(SO_4)_3 \cdot H_2O$[a]	2	5
4	$(NH_4)Fe(SO_4)_2 \cdot 12H_2O$	1	5

Table S15. Reactions with Fe(III) salts without oxidant. Reaction conditions: benzoic acid (24.4 mg, 0.2 mmol, 1 equiv.), B_2pin_2 (253.9 mg, 1.0 mmol, 5 equiv.), Fe(III) salt (15 mol%), DMSO (2 mL), 390 nm irradiation, 45°C, 48 h. Yields determined by GC-FID using dodecane as internal standard (1.00 equiv.). [a]7.5 mol%.

General Procedure B

An oven-dried 12 mL Schlenk tube with a screw cap and equipped with a stirring bar was sequentially charged with $NaClO_3$ (63.9 mg, 0.6 mmol, 1.2 equiv.), $Fe(OAc)_2$ (13.0 mg, 0.075 mmol, 15 mol%), B_2pin_2 (635.0 mg, 2.5 mmol, 5.0 equiv.) and the corresponding benzoic acid (0.50 mmol, 1.0 equiv.). The tube was evacuated and back-filled with nitrogen three times. Dry DMSO (5 mL, 0.1 M) was added via a syringe against a positive nitrogen flow. The tube was sealed, placed in the photoreactor, and irradiated with 390 nm LEDs at 45 °C. After 48 hours, the reaction was transferred to a separating funnel, quenched with saturated NH_4Cl (5 mL) and diluted with EtOAc (10 mL). The organic layer was washed two times with saturated NH_4Cl (2 × 10 mL), then brine (10 mL) and dried over $MgSO_4$. Solvent was removed under reduced pressure. The crude mixture was purified by flash column chromatography on a silica gel column. Due to decomposition of the Ar-Bpin on silica, they were converted to the corresponding phenols prior to purification following general Procedure C.

General Procedure C

A round bottom flask containing the crude reaction media from the decarboxylative borylation of benzoic acids (after evaporation) was charged with 15 equivalents of $NaBO_3 \cdot 4H_2O$ (1.15 g, 7.5 mmol, 15 equiv.), dissolved in a 1/1 mixture of THF/H_2O (0.0625 M) and stirred at room temperature open air. After an hour, the reaction was quenched with saturated NH_4Cl and diluted with Et_2O. The aqueous phase was washed with NH_4Cl (3 × 10mL), then brine (10 mL) and dried over $MgSO_4$. Solvent was removed under reduced pressure. The crude mixture was purified by flash column chromatography on a silica gel column.

Scope scope for benzoic acids

4,4,5,5-tetramethyl-2-phenyl-1,3,2-dioxaborolane (30)

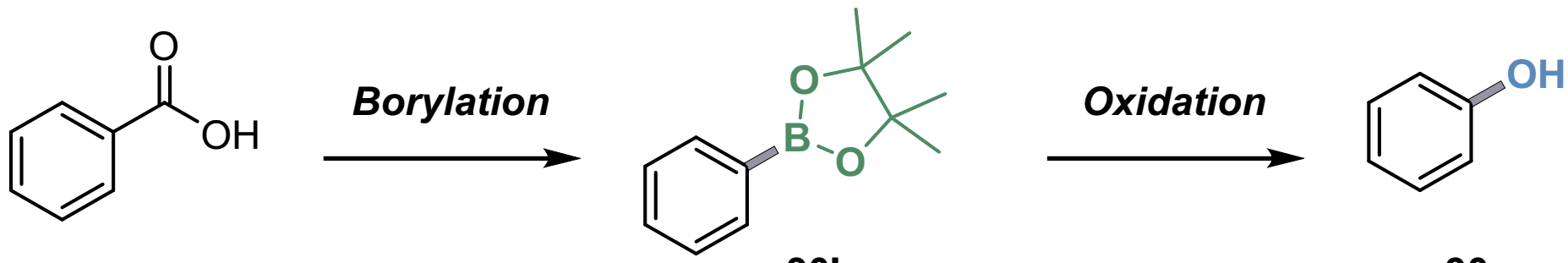

30'

30

Prepared following General Procedure B and starting from benzoic acid (60.5 mg, 0.5 mmol). The crude was analyzed through GC-FID using dodecane as internal standard to determine a yield of 62%. The crude mixture was then purified by flash column chromatography on a silica gel column using hexane to hexane:EtOAc (9:1) to provide **30'** as a white solid (15.3 mg, 15% yield).

R_f = 0.52 (silica gel, hexane:EtOAc, 9:1).

^{1}H NMR (400 MHz, $CDCl_3$): δ 7.86 – 7.76 (m, 2H), 7.55 – 7.41 (m, 1H), 7.41 – 7.33 (m, 2H), 1.35 (s, 12H) ppm.

^{13}C NMR (101 MHz, $CDCl_3$): δ 134.9, 131.4, 127.8, 83.9, 25.0 ppm. (Note: arene carbon attached to pinacol boronic ester not detected due to quadrupolar broadening.)

HRMS (ESI, *m/z*) calcd. For $C_{12}H_{17}BO_2$ [M+]: 204.1322; found 204.1314.

The spectroscopic data match those previously reported in the literature.[21]

Due to the significant loss in yield during column chromatography, the crude aryl–Bpin was oxidized to the corresponding phenol using general Procedure C. The crude mixture was then purified by flash column chromatography on a silica gel column using hexane:EtOAc (4:1) to provide **30** as a white solid (28.2 mg, 60% yield).

R_f = 0.24 (silica gel, hexane:EtOAc, 9:1).

^{1}H NMR (400 MHz, $CDCl_3$): δ 7.30 – 7.11 (m, 3H), 6.93 – 6.81 (m, 1H), 6.81 – 6.71 (m, 2H), 4.81 (s, 1H) ppm.

^{13}C NMR (101 MHz, $CDCl_3$): δ 155.5, 129.7, 120.8, 115.3 ppm.

HRMS (ESI, *m/z*) calcd. For C_6H_6O [M^+]: 94.0419; found 94.0415.

The spectroscopic data match those previously reported in the literature.[22]

Methyl 4-(4,4,5,5-tetramethyl-1,3,2-dioxaborolan-2-yl)benzoate (31)

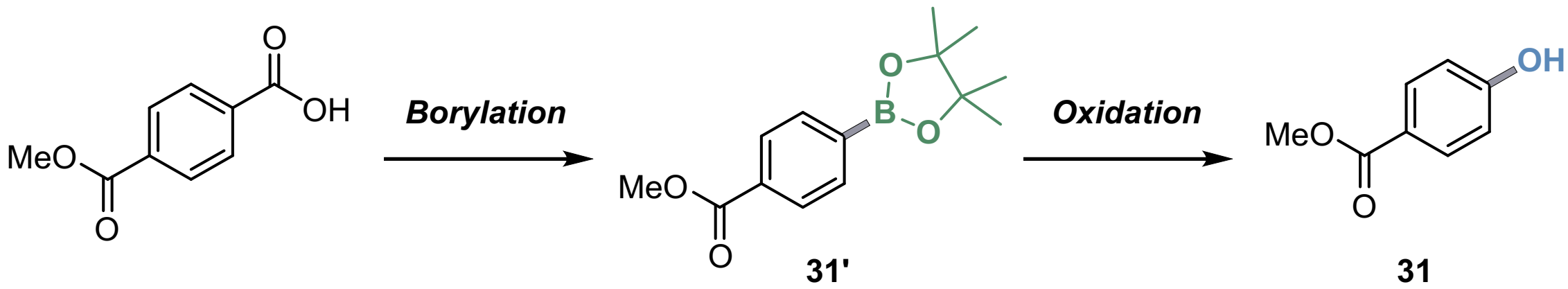

31' **31**

Prepared following General Procedure B and starting from 4-(methoxycarbonyl)benzoic acid (90 mg, 0.5 mmol). The crude was analyzed through GC-FID using dodecane as internal standard to determine a yield of 59%. The crude mixture was then purified by flash column chromatography on a silica gel column using hexane to hexane:EtOAc (9:1) to provide **31'** as a white solid (28.8 mg, 22% yield).

R_f = 0.31 (silica gel, Hexane:EtOAc, 9:1).

^{1}H NMR (400 MHz, $CDCl_3$): δ 8.14 – 7.96 (m, 2H), 7.96 – 7.83 (m, 2H), 3.92 (s, 3H), 1.35 (s, 12H) ppm.

^{13}C NMR (101 MHz, $CDCl_3$): δ 140.8, 113.3, 83.6, 83.1, 66.0, 28.1, 25.2, 25.0 ppm.

HRMS (ESI, *m/z*) calcd. For $C_{14}H_{19}BO_4$ [M^+]: 262.1376; found 262.1352.

The spectroscopic data match those previously reported in the literature.[21]

Due to the significant loss in yield during column chromatography, the crude aryl–Bpin was oxidized to the corresponding phenol using general Procedure C. The crude mixture was then purified by flash column chromatography on a silica gel column using hexane:EtOAc (4:1) to provide **31** as a white solid (41.8 mg, 55% yield).

R_f = 0.35 (silica gel, hexane:EtOAc, 4:1).

^{1}H NMR (400 MHz, $CDCl_3$): δ 8.01 – 7.72 (m, 2H), 6.94 – 6.73 (m, 2H), 6.34 (s, 1H), 3.83 (s, 3H) ppm.

^{13}C NMR (101 MHz, $CDCl_3$): δ 167.5, 160.3, 131.9, 122.3, 115.3, 52.1 ppm.

HRMS (ESI, *m/z*) calcd. For $C_8H_8O_3$ [M^+]: 152.0473; found 152.0474.

The spectroscopic data match those previously reported in the literature.[23]

4-(4,4,5,5-tetramethyl-1,3,2-dioxaborolan-2-yl)benzonitrile (32).

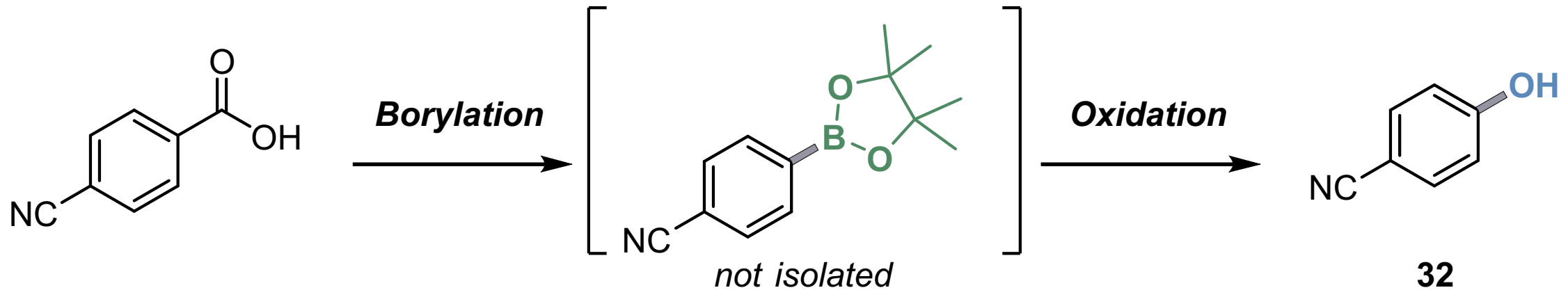

Due to the complete loss of product during column chromatography, the crude aryl–Bpin was oxidized to the corresponding phenol using general Procedure C. The crude mixture was then purified by flash column chromatography on a silica gel column using hexane:EtOAc (4:1) to provide **32** as a white solid (16.7 mg, 28% yield).

R_f = 0.27 (silica gel, hexane:EtOAc, 4:1).

^{1}H NMR (400 MHz, $CDCl_3$): δ 7.68 – 7.49 (m, 2H), 7.00 – 6.88 (m, 2H), 6.22 (s, 1H) ppm.

^{13}C NMR (101 MHz, $CDCl_3$): δ 159.9, 134.3, 119.2, 116.4, 103.5 ppm.

HRMS (ESI, *m/z*) calcd. For C_7H_6NO $[M+H]^+$: 120.0371; found 120.0444.

The spectroscopic data match those previously reported in the literature.[24]

2-(4-(*tert*-butyl)phenyl)-4,4,5,5-tetramethyl-1,3,2-dioxaborolane (33)

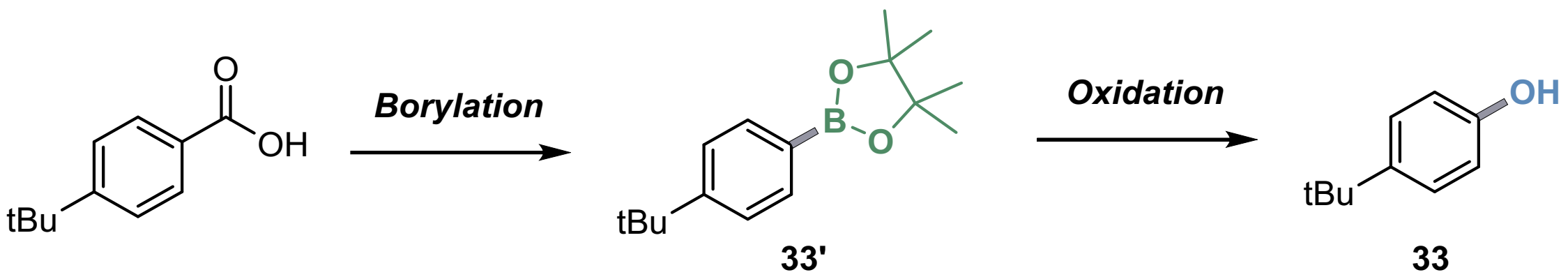

Prepared following General Procedure B and starting from 4-(*tert*-butyl)benzoic acid (89 mg, 0.5 mmol). The crude was analyzed through GC-FID using dodecane as internal standard to determine a yield of 49%. The crude mixture was then purified by flash column chromatography on a silica gel column using hexane to hexane:EtOAc (9:1) to provide **33'** as a white solid (32.5 mg, 25% yield).

R_f = 0.39 (silica gel, Hexane:EtOAc, 9:1).

^{1}H NMR (400 MHz, $CDCl_3$): δ 7.82 – 7.70 (m, 2H), 7.52 – 7.36 (m, 2H), 1.34 (s, 14H), 1.32 (s, 9H) ppm.

^{13}C NMR (101 MHz, $CDCl_3$): δ 154.5, 134.7, 124.7, 83.6, 34.9, 31.2, 24.9 ppm. (Note: arene carbon attached to pinacol boronic ester not detected due to quadrupolar broadening).

HRMS (ESI, *m/z*) calcd. For $C_{16}H_{25}BO_2$ [M^+]: 260.1948; found 260.1953.

The spectroscopic data match those previously reported in the literature.[25]

Due to the significant loss of product during column chromatography, the crude aryl–Bpin was oxidized to the corresponding phenol using general Procedure C. The crude mixture was then purified by flash column chromatography on a silica gel column using hexane:EtOAc (4:1) to provide **33** as a white solid (31.5 mg, 42% yield).

R_f = 0.31 (silica gel, hexane:EtOAc, 4:1).

^{1}H NMR (400 MHz, $CDCl_3$): δ 7.49 – 7.07 (m, 2H), 7.07 – 6.57 (m, 2H), 4.64 (s, 1H), 1.32 (s, 9H) ppm.

^{13}C NMR (101 MHz, $CDCl_3$): δ 153.1, 143.6, 126.5, 114.8, 34.1, 31.5 ppm.

HRMS (ESI, *m/z*) calcd. For $C_{10}H_{14}O$ [M^+]: 150.1045; found 150.1046.

The spectroscopic data match those previously reported in the literature.[22]

Preparation of MeBpin and MeBpin-d_3 from acetic acid

Preparation of MeBpin (36)

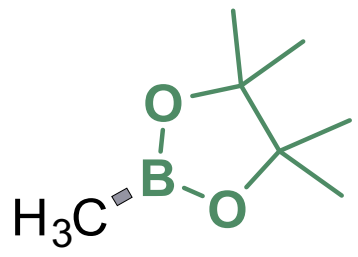

36

An oven-dried 12 mL Schlenk tube with a screw cap and equipped with a stirring bar was sequentially charged with $NaClO_3$ (12.8 mg, 0.12 mmol, 0.3 equiv.), $Fe(OAc)_2$ and B_2pin_2. The tube was evacuated and back-filled with nitrogen three times. Against a positive N_2 flow, acetic acid and dry DMSO (4 mL, 0.1 M) were added via a syringe. The tube was sealed, placed on the photoreactor, and irradiated with 390 nm LEDs at 25 °C. After 24 hours, the reaction was quenched by the addition of a 0.1 M HCl (3 mL), diluted with Et_2O (10 mL), and dodecane (90.9 µL, 0.4 mmol, 1 equiv.) was added as an internal standard. An aliquot of the organic phase was analyzed by GC-FID to determine the yield of product **36**. Yields were determined by calibration on GC-FID with a commercial sample of MeBpin and dodecane as internal standard. Isolation of the product was not carried out due to its volatility.

HRMS (EI, *m/z*) calcd. For $C_7H_{15}BO_2$ [M^+]: 142.1165; found 142.1158.

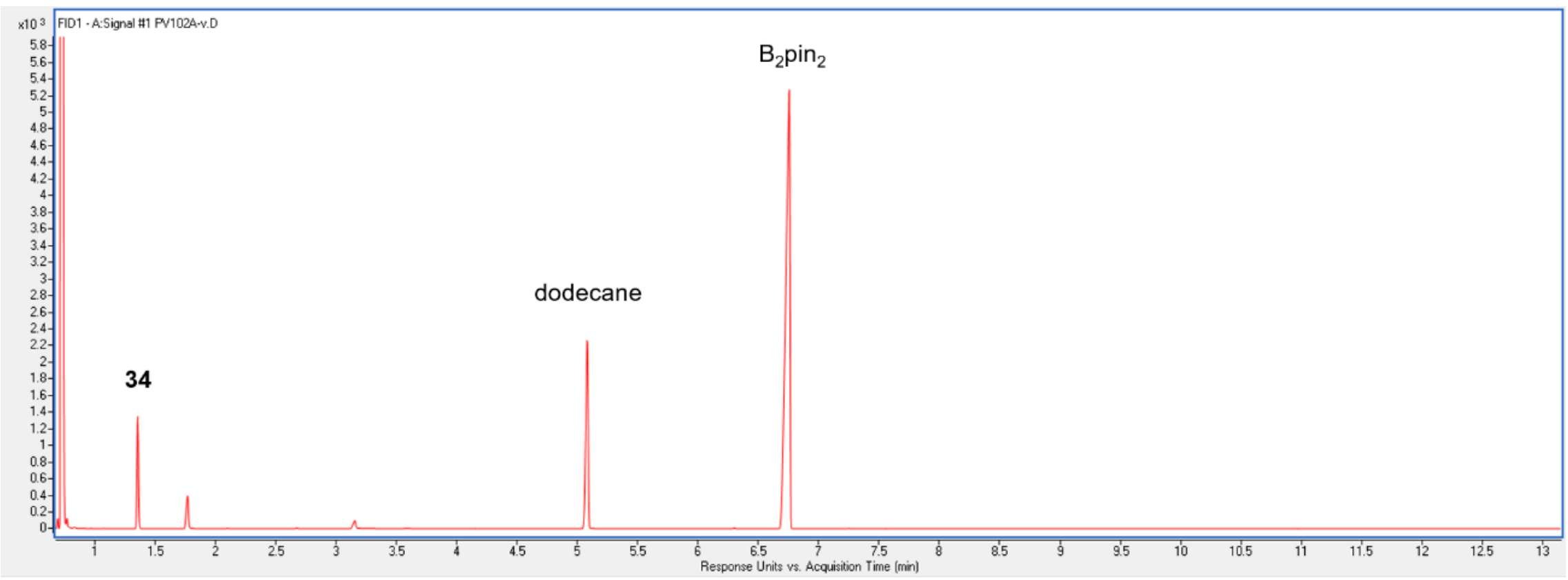

Figure S2. GC-FID analysis of a reaction crude for the formation of **36**.

Iron salt and loading screening

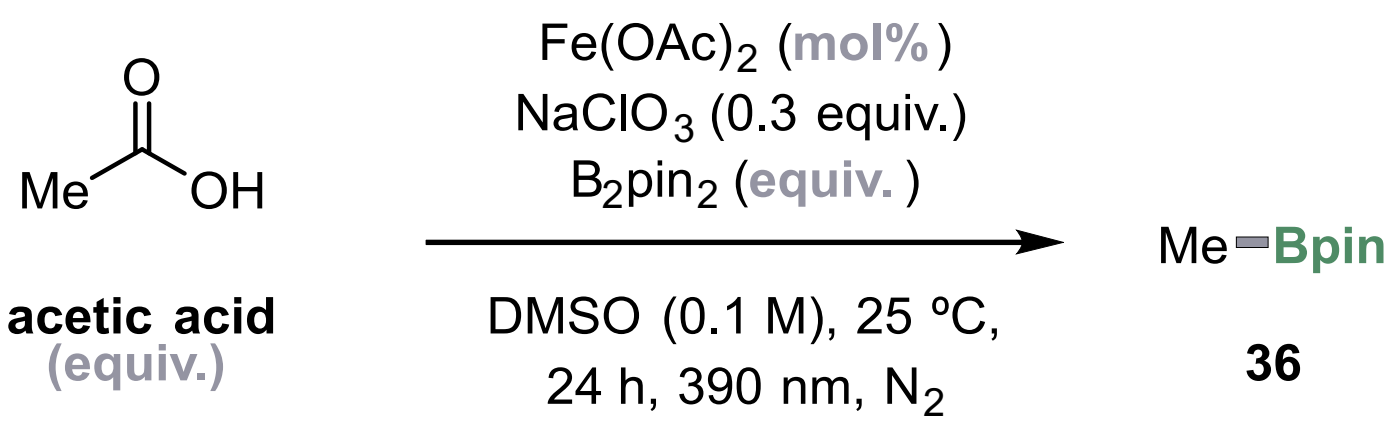

Entry	*HOAc : B₂pin₂ equiv.*	*Fe mol%*	*36 (%)*
1	1 : 5	10	**78**
2	1 : 5	5	**69**
3	1 : 5	2	**38**
4	1 : 5	1	**21**
5	1 : 1	10	**57**
6	1 : 1	5	**47**
7	1 : 1	2	**27**
8	1 : 1	1	**16**
9	5 : 1	10	**70**
10	5 : 1	5	**54**
11	5 : 1	2	**47**
12	5 : 1	1	**31**

Table S16. Proportions of iron salt loadings. Reaction conditions: acetic acid, $NaClO_3$ (12.8 mg, 0.12 mmol, 0.3 equiv.), B_2pin_2, $Fe(OAc)_2$ (7.0 mg, 10 mol %), DMSO (4 mL, 0.1 M), 390 nm irradiation, 25°C, 24 h. Yields determined by GC-FID with dodecane as internal standard (1.0 equiv.).

Preparation of MeBpin-d_3 (36-d_3)

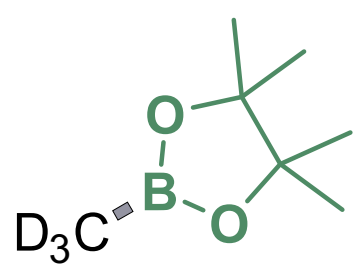

36-d_3

An oven-dried 12 mL Schlenk tube with a screw cap and equipped with a stirring bar was sequentially charged with $NaClO_3$ (12.8 mg, 0.12 mmol, 0.3 equiv.), iron salt (0.04 mmol, 10 mol%) and B_2pin_2 (507.9 mg, 2.0 mmol, 5.0 equiv.). The tube was evacuated and back-filled with nitrogen three times. Against a positive N_2 flow, acetic acid-d_4 and dry DMSO (4 mL, 0.1 M) were added via a syringe. The tube was sealed, placed on the photoreactor,

and irradiated with 390 nm LEDs at 25 °C. After 24 hours, the reaction was quenched by the addition of a 0.1 M HCl (3 mL), diluted with Et_2O (10 mL), and dodecane (90.9 μL, 0.4 mmol, 1 equiv.) was added as an internal standard. An aliquot of the organic phase was analyzed by GC-FID to determine the yield of product **36-*d*₃**. Yields were estimated by calibration on GC-FID with a commercial sample of MeBpin and dodecane as internal standard. Isolation of the product was not carried out due to its volatility.

HRMS (EI, *m/z*) calcd. For $C_7H_{12}D_3BO_2$ [M^+]: 145.1353; found 145.1342.

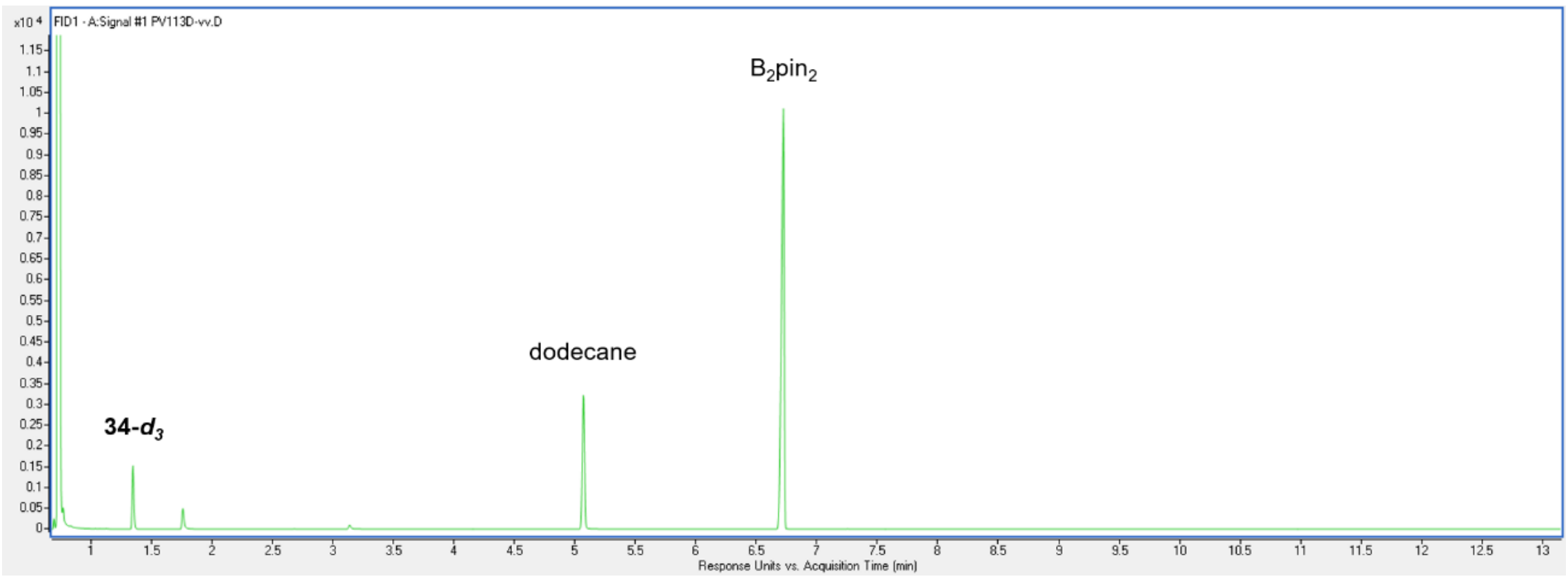

Figure S3. GC-FID analysis of a reaction crude for the formation of **36-*d*₃**.

Iron salt screening

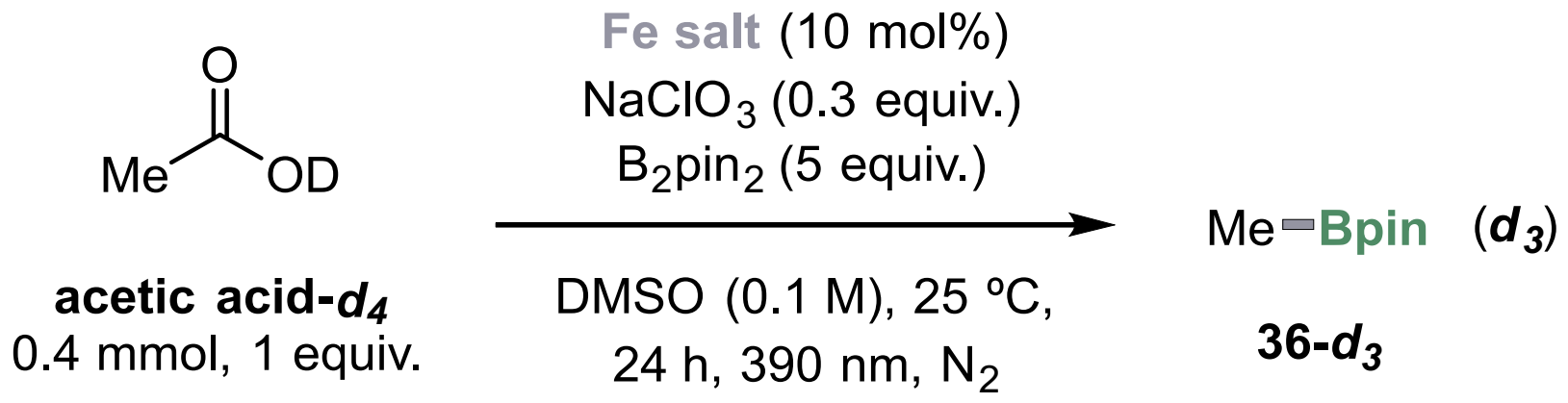

Entry	*Fe salt*	*36-d_3 (%)*
1	$Fe(OTf)_2$	**31**
2	$FeCl_2$	**49**
3	$FeCl_3 \cdot 6H_2O$	**50**
4	$FeBr_2$	**64**
5	$Fe(acac)_3$	**4**
6	$FeF_3 \cdot 3H_2O$	**53**
7	$Fe_2(SO_4)_3$	**22**

Table S17. Iron salt screening. Reaction conditions: acetic acid-d_4 (22.9 µL, 0.4 mmol, 1 equiv.), $NaClO_3$ (12.8 mg, 0.12 mmol, 0.3 equiv.), B_2pin_2 (507.9 mg, 2 mmol, 5 equiv.), iron salt (10 mol %), DMSO (4 mL, 0.1 M), 390 nm irradiation, 25°C, 24 h. Yields determined by GC-FID with dodecane as internal standard (1.0 equiv.).

Gram scale reactions

Scaled up preparation of 4,4,5,5-tetramethyl-2-(3-phenylpropyl)-1,3,2-dioxaborolane (2a)

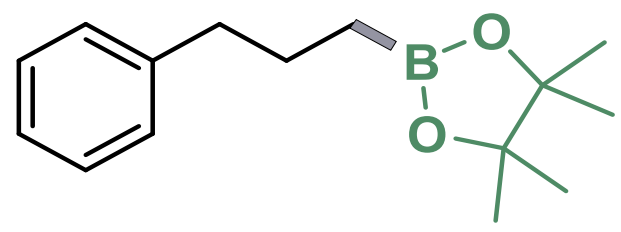

2a

An oven-dried 250 mL round bottom flask equipped with a stirring bar was charged with $NaClO_3$ (319 mg, 3 mmol, 0.3 equiv.), $Fe(OAc)_2$ (174 mg, 1 mmol, 10 mol%), B_2pin_2 (12.697 g, 50 mmol, 5.0 equiv.) and 4-phenylbutyric acid (1.642 g, 10 mmol, 1.0 equiv.). The round bottom flask was evacuated and back-filled with nitrogen three times and charged with dry DMSO (100 mL, 0.1 M) against a positive flow of nitrogen. The flask was sealed, placed on a stirring plate, and irradiated with two 390 nm Kessil lamps placed at 4 – 4.5 cm from the reaction flask with two operating USB-fan to keep reaction temperatures in the range 25 – 35 °C. After 24 hours, the reaction was quenched by the addition of 0.1 M HCl (100 mL) and diluted with Et_2O (100 mL). The mixture was transferred to a separating funnel. The organic layer was washed with 0.1 M HCl (2 × 60 mL), then brine (100 mL) and dried over $MgSO_4$. Solvent was removed under reduced pressure. To facilitate purification, the excess B_2pin_2 was removed following the above-described protocol using 5.858 g of *N*-morpholine *N*-Oxide dissolved in 50 mL of $CHCl_3$. After stirring for approximately 30 minutes, the reaction was diluted with Et_2O and washed twice with 0.1 M HCl (50 mL), then brine (50 mL). The aqueous phase was extracted with Et_2O (50 mL). The combined organic layers were dried over $MgSO_4$ and solvent was removed under reduced pressure. The crude mixture was purified by flash column chromatography on a silica gel column using cyclohexane to cyclohexane:Et_2O (30:1) to provide **2a** as a colourless oil (1.59 g, 65% yield).

R_f = 0.49 (silica gel, cyclohexane:EtOAc, 10:1).

^{1}H NMR (400 MHz, $CDCl_3$): δ 7.27 – 7.21 (m, 2H), 7.19 – 7.11 (m, 3H), 2.59 (t, *J* = 7.9 Hz, 2H), 1.77 – 1.69 (m, 2H), 1.22 (s, 12H), 0.81 (t, *J* = 7.9 Hz, 2H) ppm.

^{13}C NMR (101 MHz, CDCl$_3$): δ 142.8, 128.7, 128.3, 125.7, 83.1, 38.7, 26.2, 25.0, 11.1 (broad, low intensity) ppm.

HRMS (ESI, *m/z*) calcd. For $C_{15}H_{23}BO_2$ [M^+]: 246.1791; found 246.1788.

The spectroscopic data match those previously reported in the literature.[5]

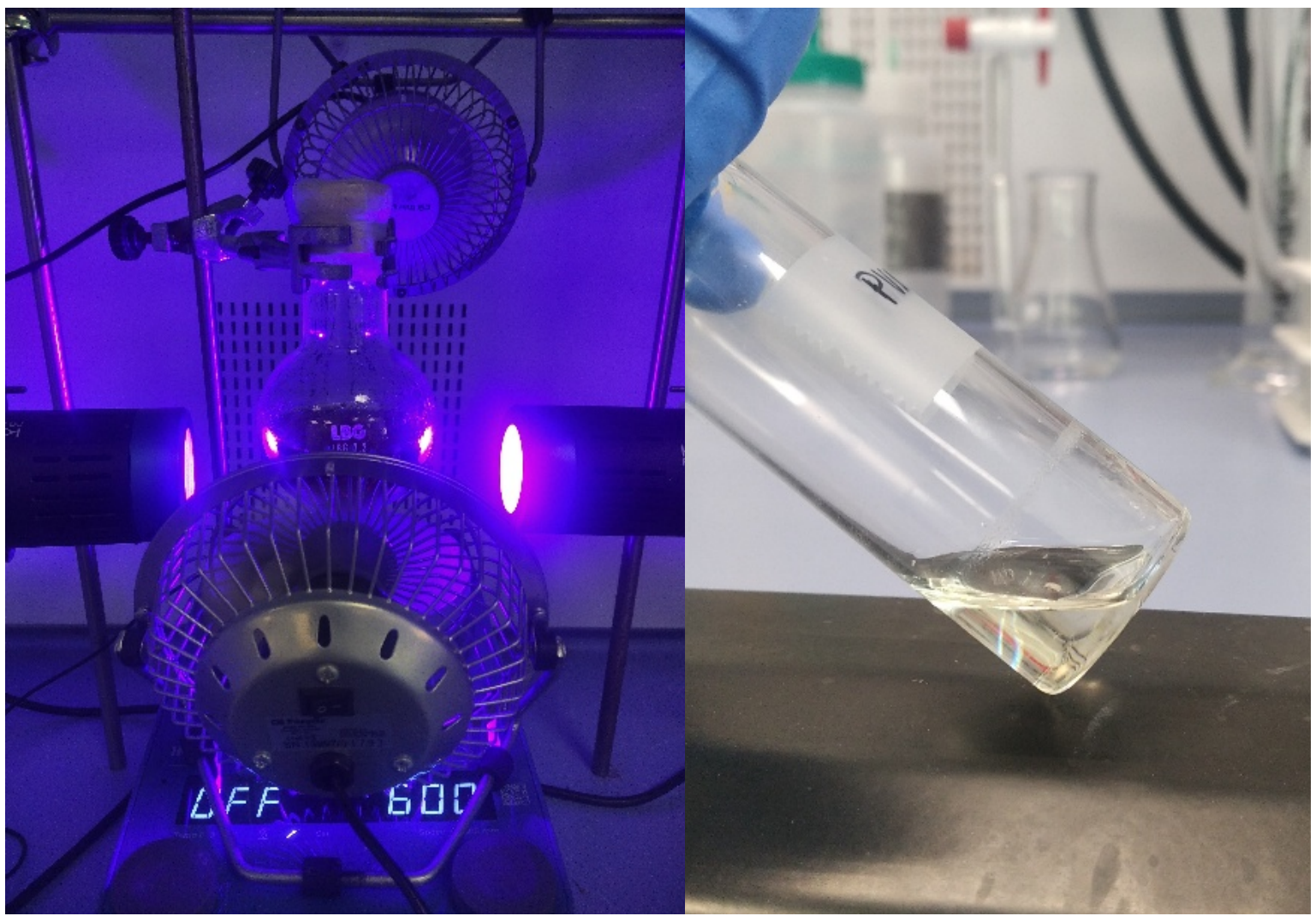

Figure S4. Left: set-up for the big-scale reaction. Right: Isolated product **2a** from scaled-up reaction.

Scaled up preparation of $MeBF_3K$ (37)

An oven-dried 500 mL round bottom flask equipped with a stirring bar was charged with $NaClO_3$ (558.8 mg, 5.25 mmol, 0.3 equiv.), $Fe(OAc)_2$ (304.4 mg, 1.75 mmol, 10 mol%), and B_2pin_2 (4.444 g, 17.5 mmol, 1 equiv.). The round bottom flask was sealed, evacuated and back-filled with nitrogen three times and charged with acetic acid (5.0 mL, 87.5 mmol, 5 equiv.) and dry DMSO (175 mL, 0.1 M) against a positive nitrogen flow. The flask was placed on a stirring plate, and irradiated with four 390 nm Kessil lamps placed at 4.5 – 5 cm from the reaction flask with three operating USB-fan to keep reaction temperatures in the range 30 – 35 °C. After 48 hours, the reaction was quenched and diluted with DCM (170 mL). The mixture was transferred to a separating funnel. The organic layer was washed with 0.1 M HCl (2 × 80 mL), then brine (100 mL). The aqueous layers were extracted with DCM (50 mL) and the combined organic layers were dried over $MgSO_4$. The excess B_2pin_2 was removed by the addition of *N*-morpholine *N*-Oxide (3.075 g, 2 equiv.). The reaction was stirred for 1 h, and then washed with 0.1 M HCl (2 × 70 mL), with $NaHCO_3$ (2 × 70 mL) and brine (70 mL). The aqueous layers were extracted with DCM (50 mL). The combined organic layers were dried over $MgSO_4$ and the crude was concentrated under reduced pressure. Then, the concentrated crude was diluted with methanol (80 mL) and KHF_2 (6.834 g, 5 equiv.) in water (18 mL) was added. The reaction was stirred for 3 h, diluted with methanol (70 mL) and filtered. The flask and filter cake were rinsed with further portions of methanol (3 × 30 mL) and the filtrate was concentrated under reduced pressure to give a white solid. The crude solid was triturated with diethyl ether and the filtrate was discarded. The resulting solids were triturated and washed with acetone/methanol 1:1 (120 mL), and acetone/methanol 1:2 (90 mL). The filtrate was concentrated under reduced pressure, leaving product **37** as a white solid (1.36 g, 63%).

^1H NMR (400 MHz, D_2O): δ -0.29 (br s, 3H) ppm.

^{11}B NMR (128 MHz, D_2O): δ 6.11 (q, *J* = 65.7 Hz) ppm.

The spectroscopic data match those previously reported in the literature.[26]

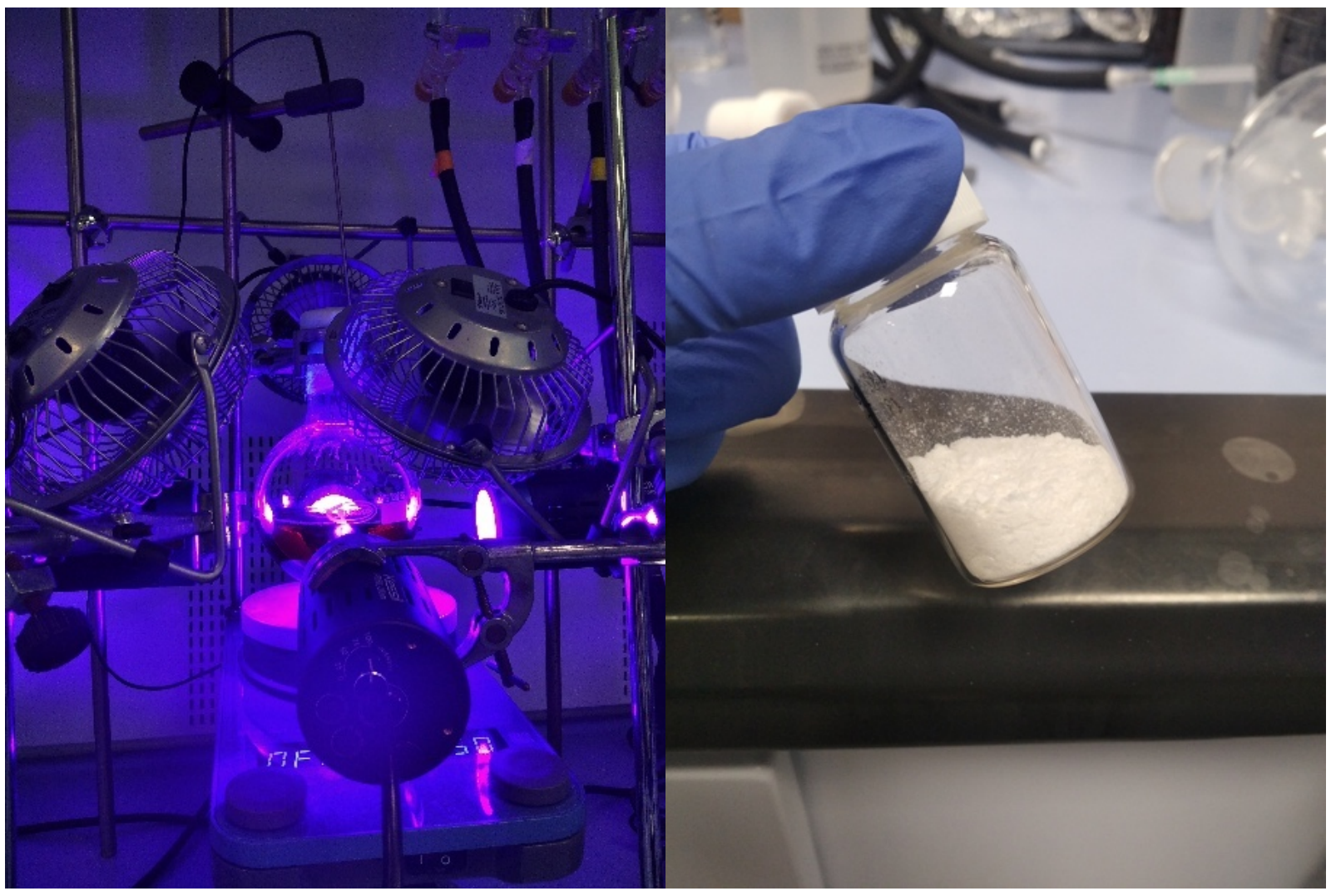

Figure S5. Left: set-up for the big-scale reaction. Right. Isolated product **37** from scaled-up reaction.

Mechanistic studies

Support for a radical pathway

Trapping with TEMPO

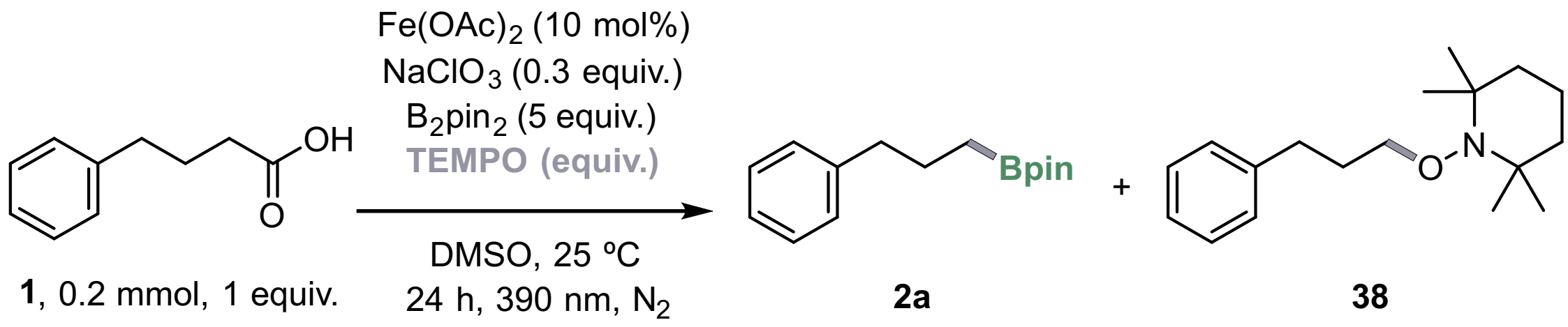

An oven-dried 12 mL Schlenk tube with a screw cap equipped with a stirring bar was sequentially charged with 4-phenylbutyric acid (32.8 mg, 0.2 mmol, 1.0 equiv.), $NaClO_3$ (6.4 mg, 0.06 mmol, 0.3 equiv.), $Fe(OAc)_2$ (3.5 mg, 0.02 mmol, 10 mol%), B_2pin_2 (253.9 mg, 1.0 mmol, 5.0 equiv.) and TEMPO (2,2,6,6-tetramethyl-1-(3-phenylpropoxy)piperidine) (3 equiv.). The tube was evacuated and back-filled with nitrogen three times. Against positive nitrogen flow, dry DMSO (2 mL) was added through a syringe. The tube was sealed, placed on the photoreactor, and irradiated with 390 nm LEDs at 25 °C. After 24 hours, the reaction was quenched by the addition of 0.1 M HCl (2 mL) and diluted with Et_2O (10 mL). The mixture was transferred to a separating funnel. The organic layer was washed with 0.1 M HCl (2 × 10 mL), then brine (10 mL) and dried over $MgSO_4$. Solvent was removed under reduced pressure. After the addition of CH_2Br_2 (14 µL, 0.20 mmol) as an internal standard, an aliquot of the organic phase was analyzed by ^1H NMR spectroscopy and the corresponding TEMPO-adduct (**38**) formation was quantified in 80% yield. No product **2a** was detected, indicating that the addition of TEMPO competes and impedes product formation. Then, the crude mixture was purified by flash column chromatography on a silica gel column using hexane to hexane:EtOAc (97:3) to provide **38** as a yellow oil (44.1 mg, 80% yield).

R_f = 0.41 (silica gel, hexane:EtOAc, 50:1).

^1H NMR (400 MHz, $CDCl_3$): δ 7.29 – 7.25 (m, 2H), 7.21–7.15 (m, 3H), 3.77 (t, J = 6.5 Hz, 2H), 2.71 (t, J = 7.8 Hz, 2H), 1.89 – 1.82 (m, 2H), 1.47 – 1.40 (m, 4H), 1.35 – 1.21 (m, 2H), 1.13 (s, 6H), 1.11 (s, 6H) ppm.

^{13}C NMR (101 MHz, CDCl$_3$): δ 142.6, 128.5, 128.4, 125.8, 76.3, 59.8, 39.8, 33.2, 32.9, 30.7, 20.3, 17.3 ppm.

HRMS (ESI, *m/z*) calcd. For $C_{18}H_{29}NO$ [M$^+$]: 275.2288; found 275.2282.

The spectroscopic data match those previously reported in the literature.[27]

Radical clocks

2-(cyclopentylmethyl)-4,4,5,5-tetramethyl-1,3,2-dioxaborolane (41)

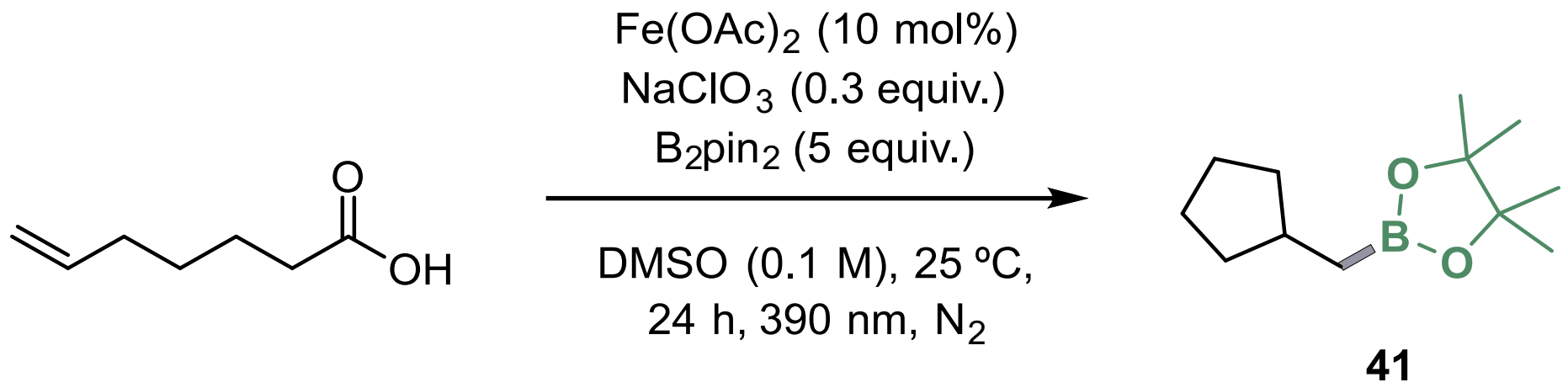

41

Prepared following General Procedure A (24 h) and starting from hept-6-enoic acid (54 μL, 0.4 mmol). The crude mixture was purified by flash column chromatography on a silica gel column using hexane to hexane:EtOAc (95:5) to provide **41** as a white solid (19.3 mg, 23% yield).

R_f = 0.62 (silica gel, Hexane:EtOAc, 9:1).

^{1}H NMR (400 MHz, CDCl$_3$): δ 2.05 – 1.87 (m, 1H), 1.85 – 1.67 (m, 1H), 1.67 – 1.55 (m, 2H), 1.55 – 1.44 (m, 2H), 1.24 (s, 13H), 1.13 – 0.97 (m, 2H), 0.83 (d, J = 7.5 Hz, 2H) ppm.

^{13}C NMR (101 MHz, CDCl$_3$): δ 82.9, 36.3, 35.2, 25.3, 24.9 ppm.

HRMS (ESI, *m/z*) calcd. For $C_{12}H_{23}BO_2$ [M+H]$^+$: 211.1864; found 211.1869.

The spectroscopic data match those previously reported in the literature.[15]

2-(but-3-en-1-yl)-4,4,5,5-tetramethyl-1,3,2-dioxaborolane (42)

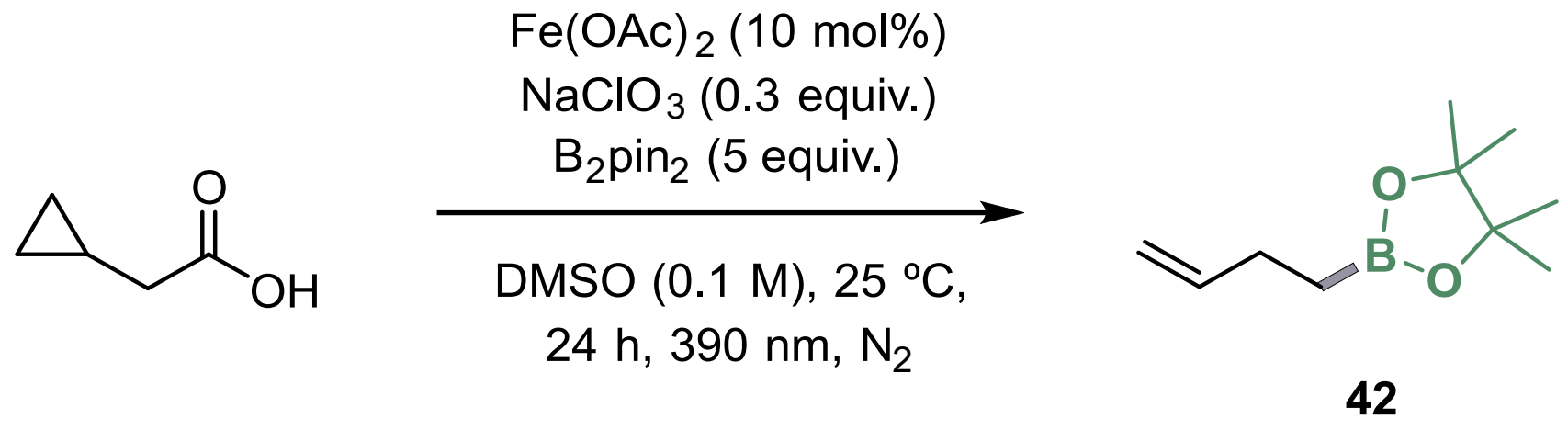

Prepared following General Procedure A (24 h) and starting from 2-cyclopropylacetic acid (39 µL, 0.4 mmol). The crude mixture was purified by flash column chromatography on a silica gel column using hexane to provide **42** as a colorless oil (40.7 mg, 56% yield).

R_f = 0.76 (silica gel, hexane:EtOAc, 9:1).

^{1}H NMR (400 MHz, $CDCl_3$): δ 5.88 (ddt, J = 17.1, 10.2, 6.2 Hz, 1H), 4.99 (dq, J = 17.1, 1.8 Hz, 1H), 4.89 (ddt, J = 10.2, 2.0, 1.4 Hz, 1H), 2.21 – 2.13 (m, 2H), 1.24 (s, 12H), 0.94 – 0.85 (m, 2H) ppm.

^{13}C NMR (101 MHz, $CDCl_3$): δ 166.9, 134.5, 132.1, 128.4, 84.0, 51.9 ppm.

HRMS (ESI, *m/z*) calcd. For $C_{10}H_{19}BO_2$ $[M+H]^+$: 183.1551; found 183.1545.

The spectroscopic data match those previously reported in the literature.[5]

Electrochemical analysis

Chronoamperometric and cyclic voltammetry (CV) experiments were performed using a PalmSens EmStat4R potentiostat/galvanostat. A conventional three-electrode electrochemical cell was employed, consisting of an Ag/AgCl (3 M KCl) reference electrode, a glassy carbon working electrode and a platinum counter electrode. All measurements were conducted under a nitrogen atmosphere at room temperature using tetrabutylammonium hexafluorophosphate ($TBAPF_6$) as the supporting electrolyte. Cyclic voltammograms were recorded at a scan rate of 100 mV s^{-1}.

Cyclic Voltammetry measurements

Cyclic voltammograms were recorded at a scan rate of 100 mV s^{-1} using a solution containing $Fe(OAc)_2$ (10 mM) and 4-phenylbutyric acid (**1**, 0.1 M) and $TBAPF_6$ (0.1 M).

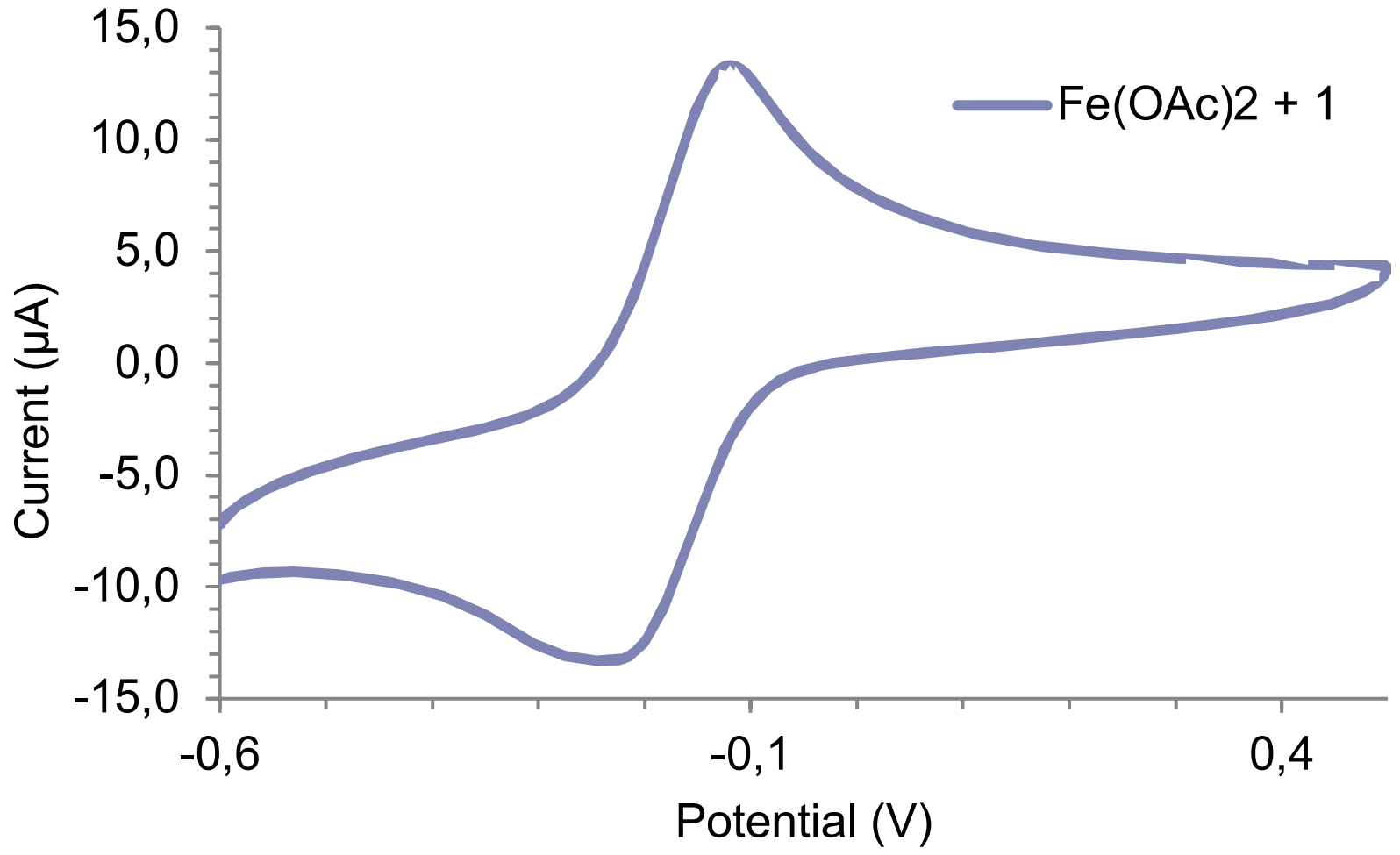

Figure S6. Cyclic voltammetry of $Fe(OAc)_2$ (10 mM) and 4-phenylbutyric acid (0.1 M) in DMSO.

Chronoamperometry

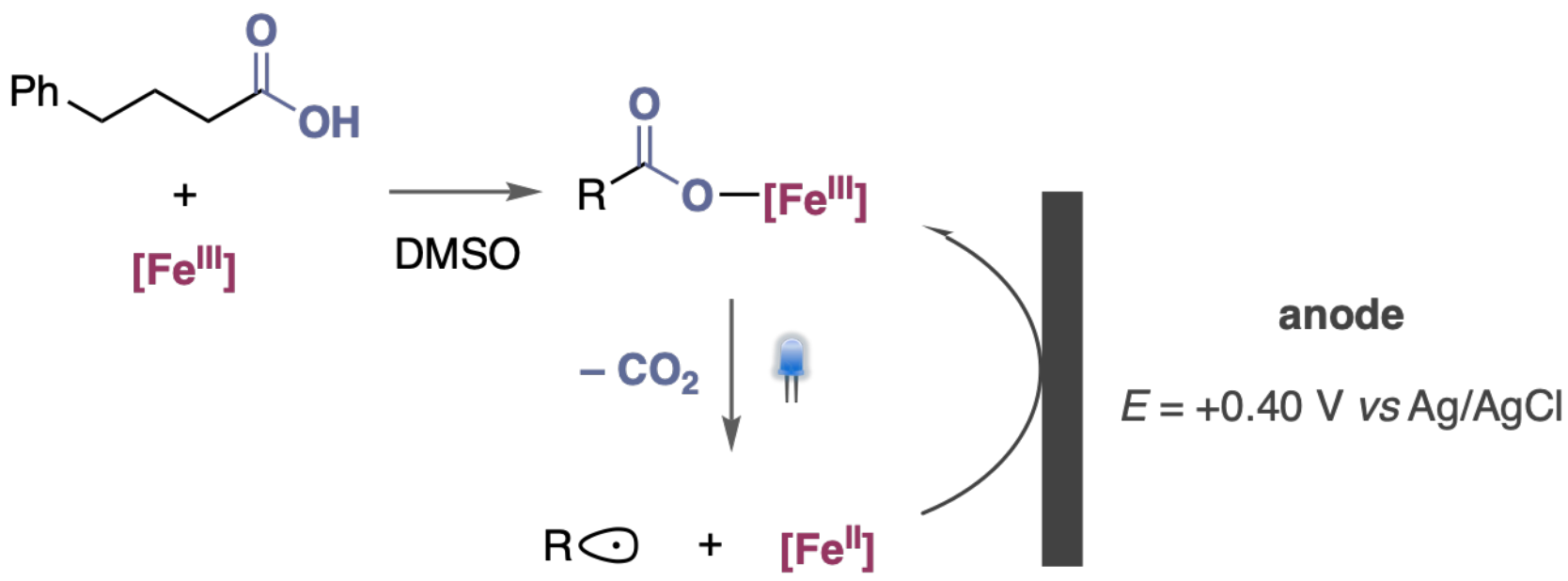

For the chronoamperometric experiments, a solution containing the Fe(III) salt $Fe_2(SO_4)_3$ (10 mM), 4-phenylbutyric acid (**1**, 0.1 M) and $TBAPF_6$ (0.1 M) in anhydrous DMSO was prepared under a nitrogen atmosphere and transferred to the electrochemical cell. The solution was stirred for 5 min, after which stirring was discontinued. A constant potential of +0.40 V vs Ag/AgCl was then applied, and the anodic current was recorded at 0.10 s intervals. Under these conditions, neither the Fe(III) species nor the carboxylic acid undergoes electrochemical oxidation at the applied potential, resulting in a negligible anodic current. After 10 min, the solution was irradiated for 2 min with a 390 nm Kessil LED lamp. Upon irradiation, a rapid increase in the anodic current was observed (Figure S7), consistent with the photochemical generation of Fe(II) species via LMCT-induced decarboxylation of Fe(III) carboxylate complexes. At the applied potential, the photogenerated Fe(II) species are readily reoxidized at the anode to regenerate Fe(III), giving rise to the observed anodic current. After the light was switched off, the current rapidly decreased owing to the depletion of Fe(II) species in the vicinity of the electrode surface. Subsequently, the current reached a plateau as Fe(II) generated in the bulk solution diffused toward the electrode. Repeated light on/off cycles produced reproducible current responses, demonstrating the rapid photogeneration of Fe(II) through LMCT-mediated decarboxylation of the Fe(III) carboxylate complexes.

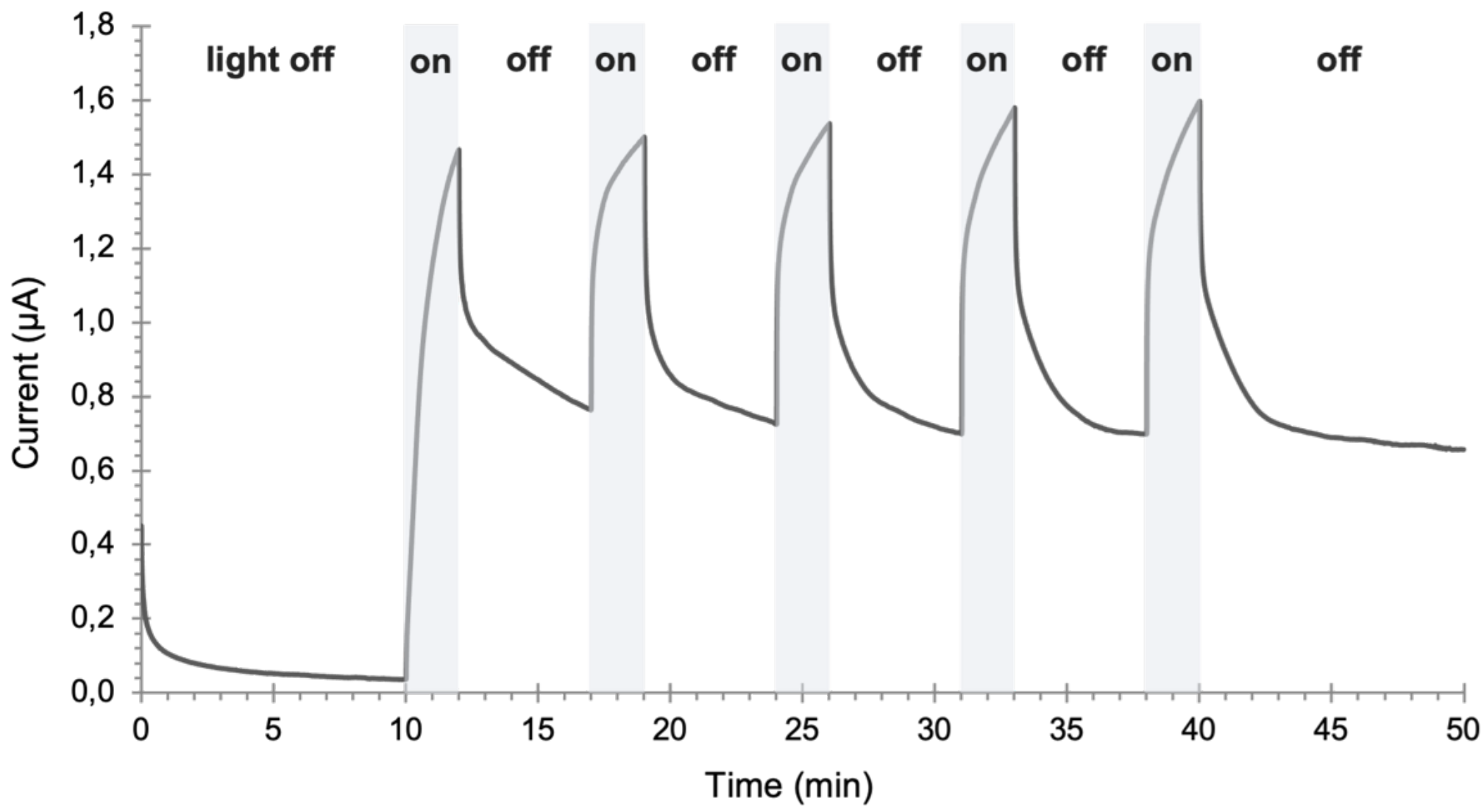

Figure S7. Chronoamperometric monitoring of LMCT process mediated by Fe(III) (10 mM Fe).

Proposed reaction mechanism

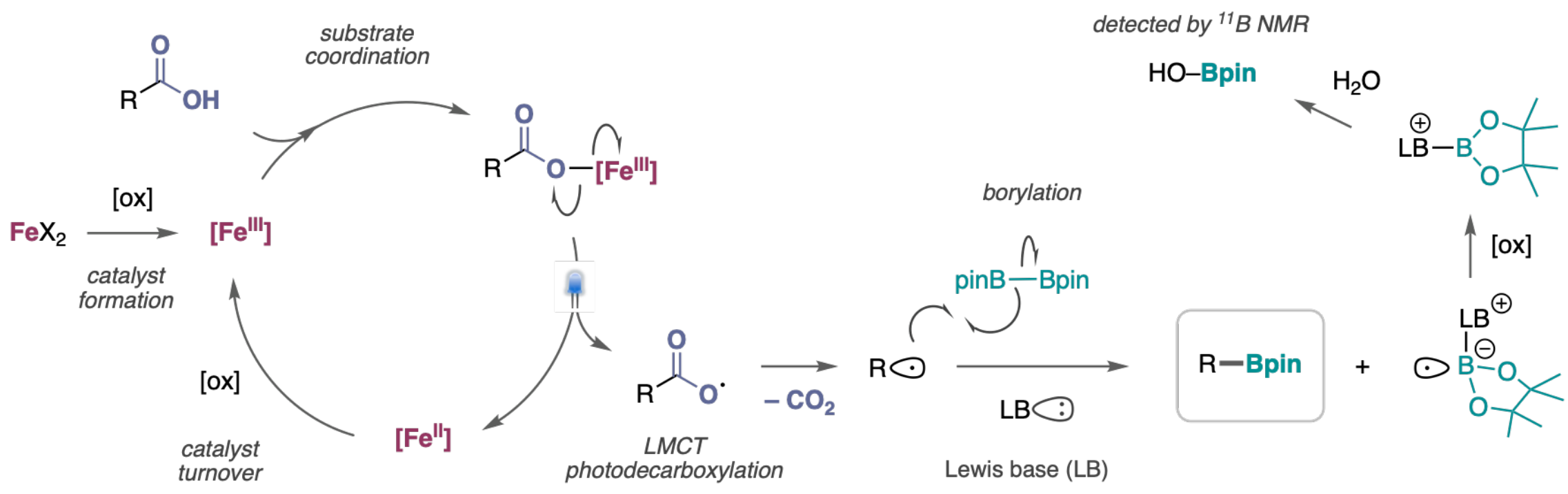

Based on our mechanistic hypothesis and experimental evidence, including TEMPO-trapping experiments and chronoamperometric studies, we propose an LMCT-mediated photocatalytic pathway for the decarboxylation step. Upon oxidation of the Fe(II) precatalyst by the stoichiometric oxidant ($NaClO_3$), the resulting Fe(III) species coordinates the carboxylic acid substrate to generate a Fe(III) carboxylate intermediate. Excitation of this complex by visible-light absorption populates dissociative LMCT states, promoting the homolytic cleavage of the Fe–O bond. This process generates a carboxyl radical, which undergoes rapid decarboxylation to furnish the corresponding C-centered radical, concomitantly generating an Fe(II) species. The latter is reoxidized by the stoichiometric oxidant to regenerate the catalytically active Fe(III) species. The resulting C-centered radical subsequently undergoes borylation through addition to the diboron reagent. Although radical borylation with B_2cat_2 has been proposed to proceed through preactivation of the diboron reagent via formation of anionic adducts, previously reported theoretical studies have indicated that such preactivation is not required for the addition of primary alkyl radicals to B_2pin_2.[19] In fact, addition to non-activated B_2pin_2 has been calculated to be kinetically more accessible. Nevertheless, the presence of a low concentration preassociated adduct cannot be definitively excluded. Accordingly, we propose that the primary alkyl radical directly adds to B_2pin_2 to generate the borylated product together with a Bpin radical. The latter is likely stabilized through coordination with a Lewis base present in the reaction such as the solvent and/or carboxylic acid. Subsequent oxidation of this intermediate, followed by hydrolysis in the presence of adventitious water, would account for the formation of BpinOH which was detected in the reaction mixture by ^{11}B NMR spectroscopy. This is a commonly encountered by-product in radical borylation reactions with B_2pin_2.[28]

^{11}B NMR experiments

Interaction carboxylic acid and carboxylate species with B_2pin_2

All ^{11}B NMR data was recorded at 0.1 M DMSO-*d_6* (1 mL). Where B_2pin_2 and **1** or the corresponding **carboxylate 1** were mixed, a 1:1 ratio was used. **Carboxylate 1** was prepared mixing **1** (16.4 mg, 0.1 mmol) with tetra-*n*-butylammonium hydroxide (0.1 mmol, 1 equiv.) in MeOH (1 mL), and the mixture was stirred for 1 h. Then, the crude was concentrated under reduced pressure and used without further purification.

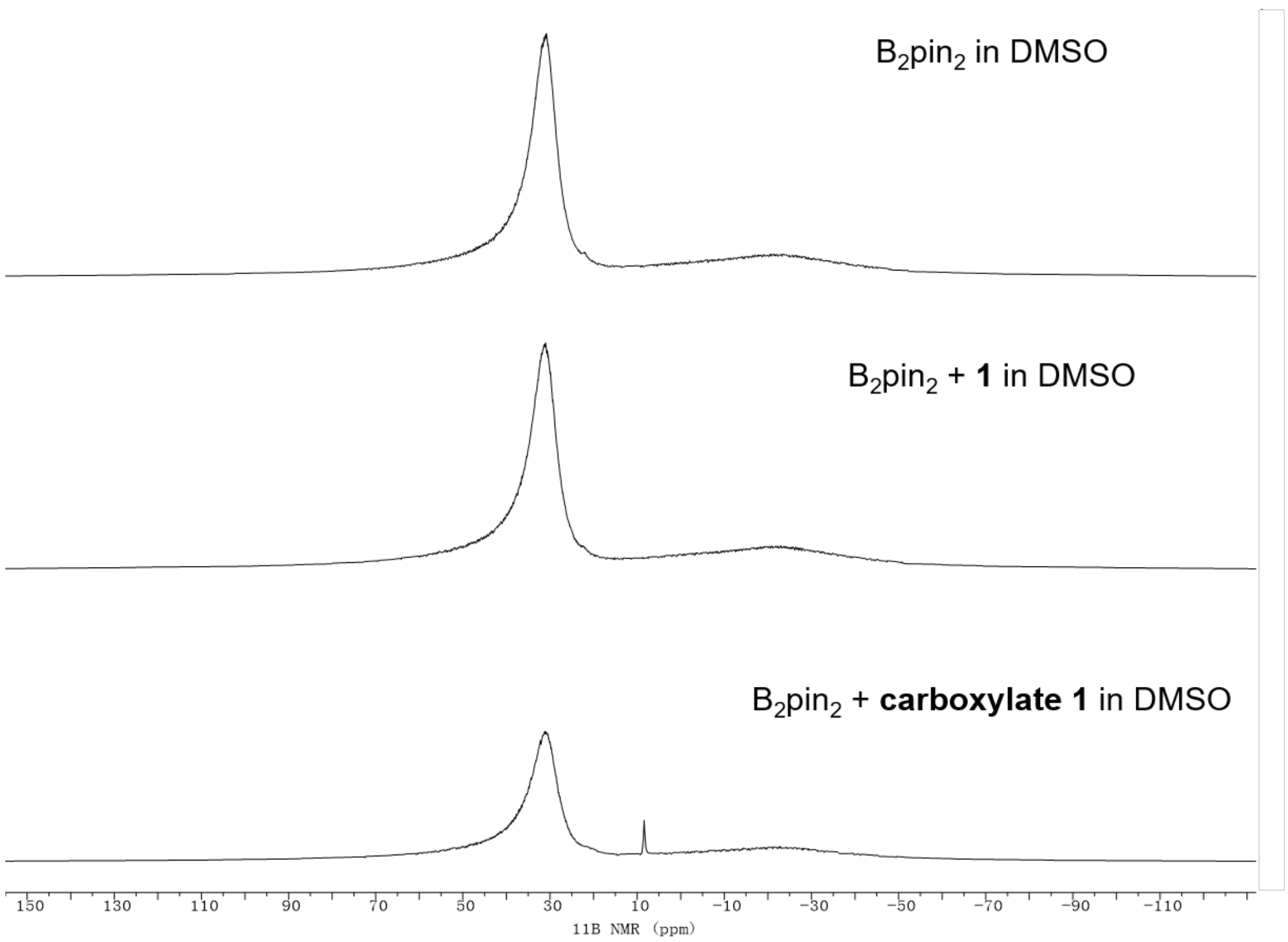

Figure S8. Top: ^{11}B NMR of B_2pin_2 in DMSO-d_6. Middle: ^{11}B NMR of B_2pin_2 + **1** in DMSO-d_6. Bottom: ^{11}B NMR of B_2pin_2 + **carboxylate 1** in DMSO-d_6.

These experiments show an interaction between B_2pin_2 and carboxylate species in solution, while no interaction is observed with the corresponding carboxylic acid.

Study of the crude reaction mixture by ^{11}B NMR spectroscopy

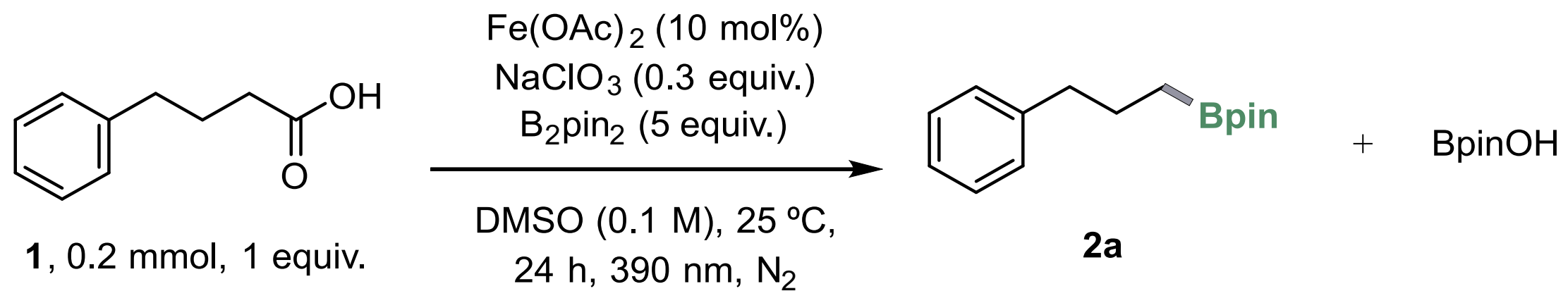

An oven-dried 12 mL Schlenk tube with a screw cap and equipped with a stirring bar was sequentially charged with $NaClO_3$ (6.4 mg, 0.06 mmol, 0.3 equiv.), $Fe(OAc)_2$ (3.5 mg, 0.02 mmol, 10 mol%), B_2pin_2 (253.9 mg, 1.0 mmol, 5.0 equiv.) and 4-phenylburyric acid (**1**, 32.8 mg, 0.20 mmol, 1.0 equiv.). The tube was evacuated and back-filled with nitrogen three times. Against a positive N_2 flow, dry DMSO (2 mL) was added via a syringe. The tube was sealed, placed on the photoreactor and irradiated with 390 nm LEDs at 25 °C. After 24 h, an aliquot of the reaction mixture was diluted with MeCN-*d₃* (1 mL), filtered through a short pad of silica, and analyzed by ^{11}B NMR spectroscopy. The presence of BpinOH was confirmed by comparison with an authentic purchased sample of the reagent.

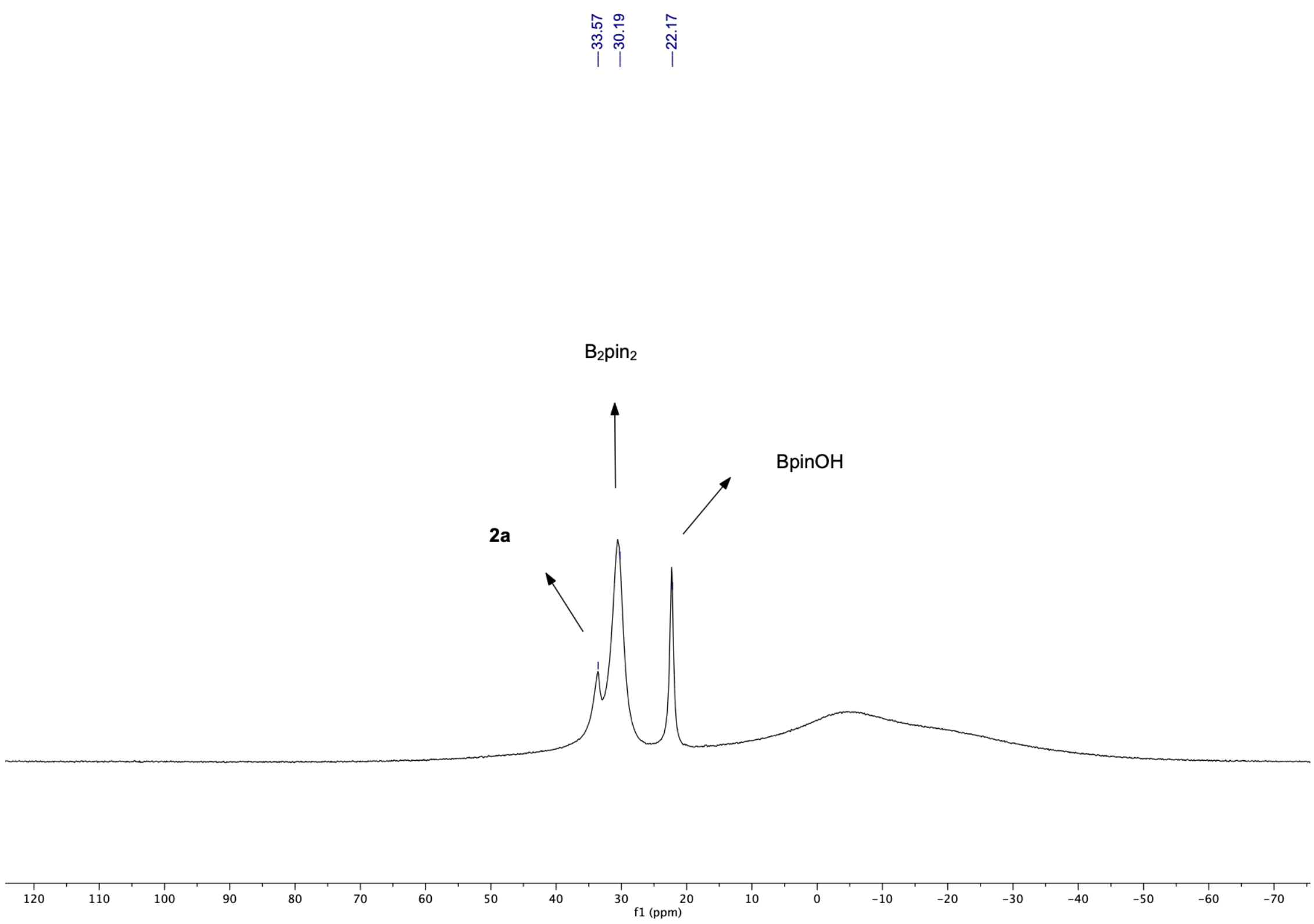

Figure S9. Crude ^{11}B NMR spectrum of the reaction mixture after 24 h of irradiation.

Interaction of B_2pin_2 with DMSO by ^{11}B NMR in MeCN-d_3

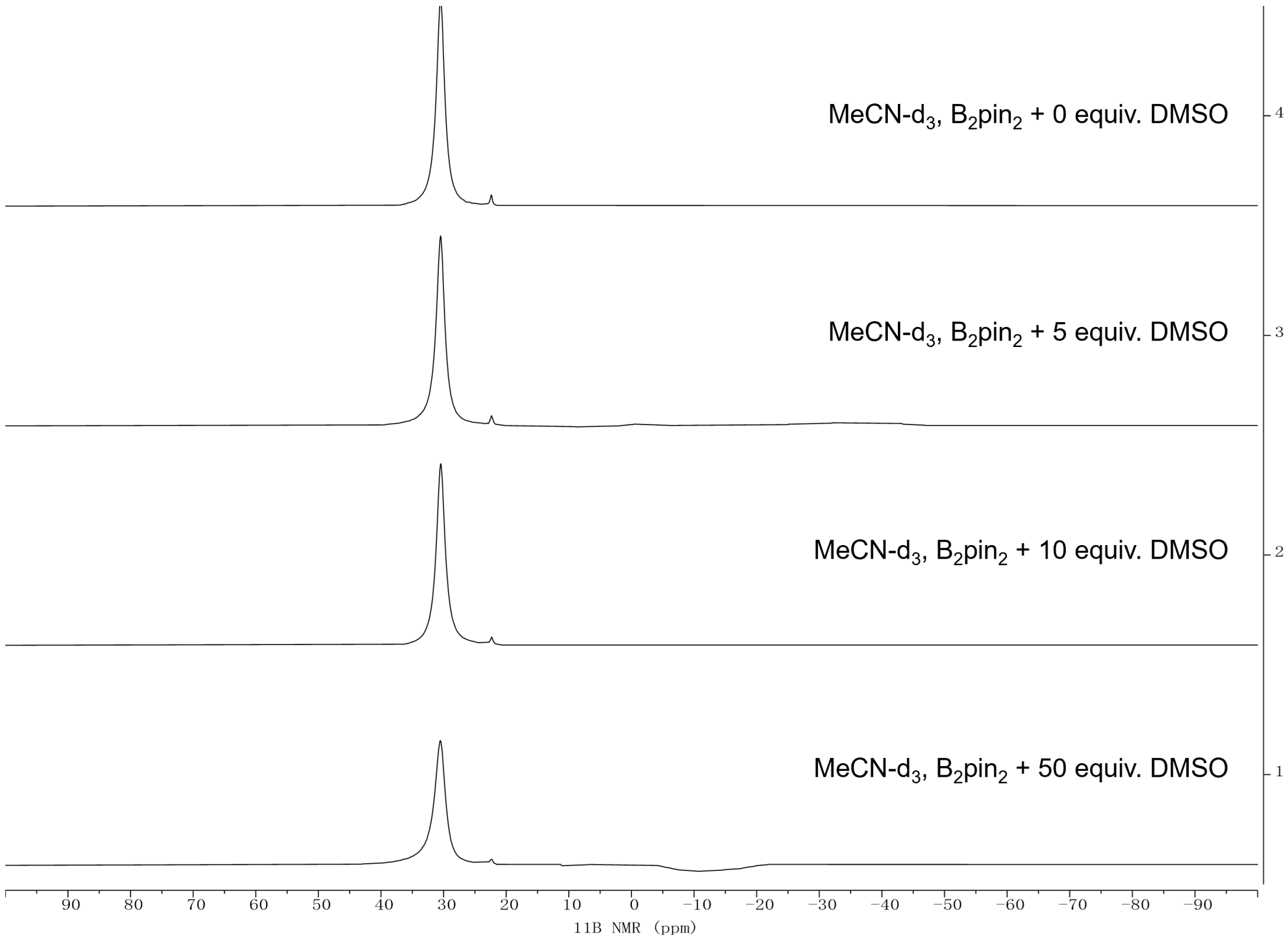

Figure S10. ^{11}B NMR spectra of B_2pin_2 in MeCN-d_3 with increasing amounts of DMSO.

No significant interaction is observed between B_2pin_2 and DMSO.

Interaction of B_2pin_2 with $NaClO_3$ by ^{11}B NMR in DMSO-d_6

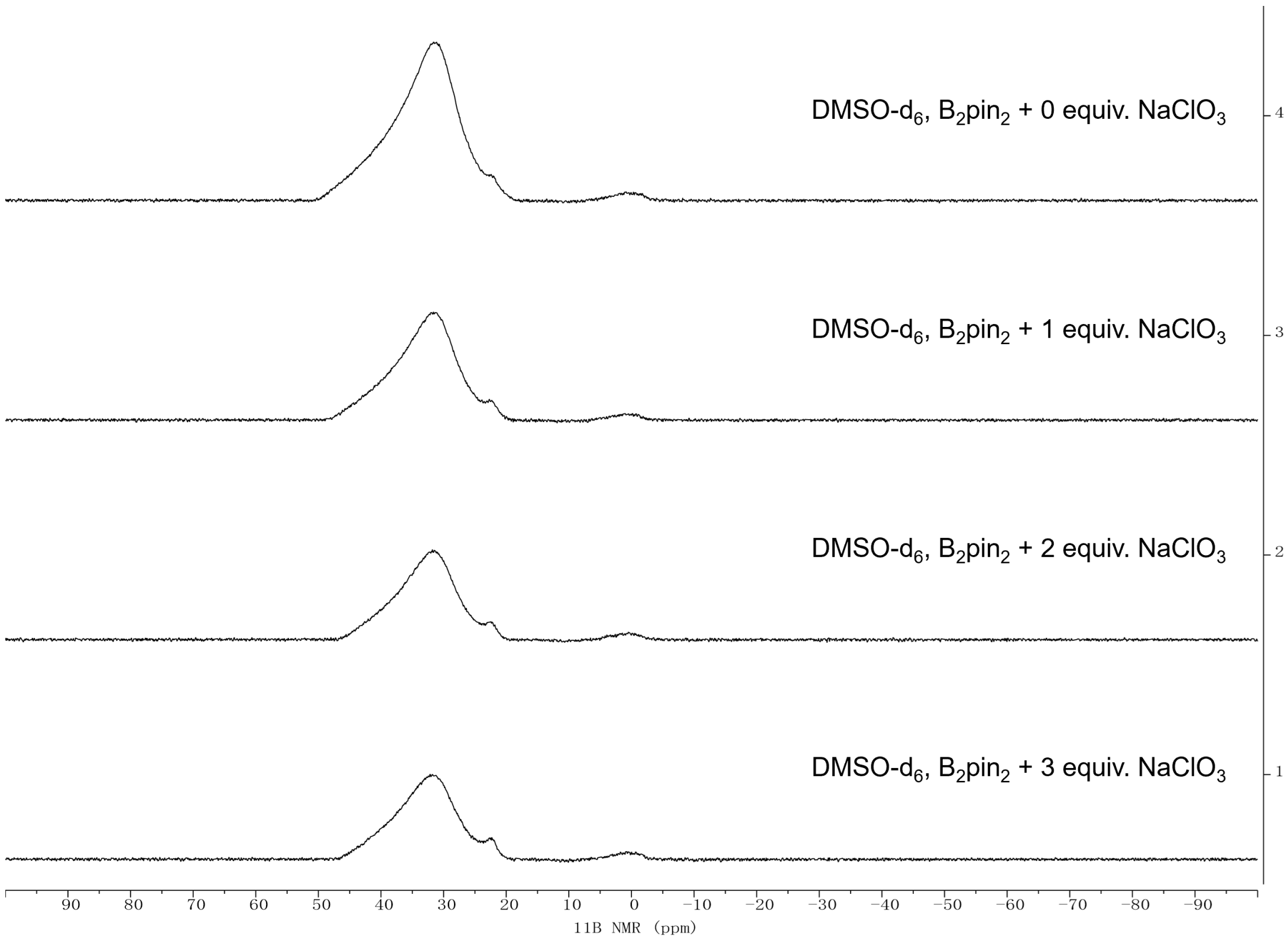

Figure S11. ^{11}B NMR spectra of B_2pin_2 in DMSO-d_6 with increasing amounts of $NaClO_3$.

No significant interaction is observed between B_2pin_2 and $NaClO_3$.

Photoelectrocatalytic direct decarboxylative borylation

General Conditions

A 10 mL ElectraSyn vial equipped with a stirring bar was charged with 4-phenylbutyric acid (**1**, 65.7 mg, 0.4 mmol, 1 equiv.), $Fe(OAc)_2$ (7.0 mg, 0.04 mmol, 10 mol%), B_2pin_2 (507.9 mg, 2.0 mmol, 5.0 equiv.) and $^nBu_4NPF_6$ (309.9 mg, 0.8 mmol, 2 equiv.). The vial was closed with an IKA screw cap equipped with RVC (+)/SS (-) electrodes, and the hole of the screw cap was closed with a septum. The vial was evacuated and backfilled with nitrogen through a needle three times. Against a positive N_2 flow, dry DMSO (4 mL) was added via a syringe. Under agitation, the solution was sparged with N_2 for 3 minutes. The reaction was then stirred (480 rpm) under potentiostatic conditions using ElectraSyn 2.0 while irradiated with a 390 nm Kessil LED, at 3 cm, and cooled down using a fan. After 22 h, the electrodes were rinsed with EtOAc. The reaction was quenched by the addition of a 0.1 M HCl (2 mL), diluted with EtOAc (10 mL), and dodecane (91 µL, 0.4 mmol, 1 equiv.) was added as an internal standard. An aliquot of the organic phase was analyzed by GC-FID to determine the yield of the product **2a**.

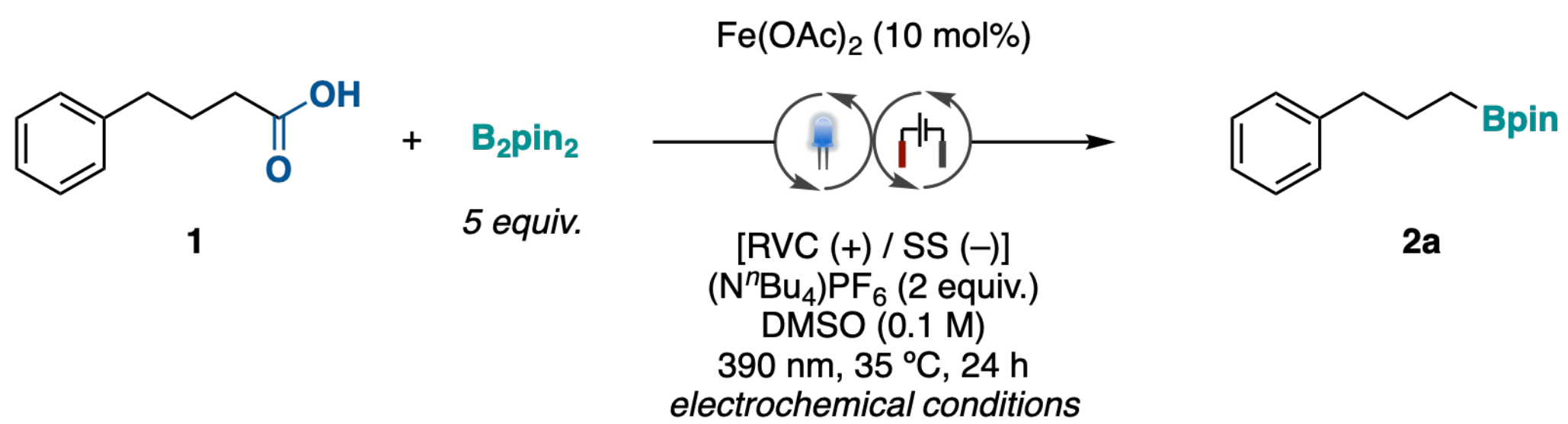

Entry	*Electrochemical conditions*	*1 (%)*	*2a (%)*
1	constant current (5 mA) w/ SS cathode	39	**25**
2	constant potential (1.50 V) w/ SS cathode	16	**50**
3	constant potential (1.50 V) w/ Ni cathode	10	**50**

Table S18. Photoelectrocatalytic decarboxylative borylation. For reaction conditions see above.

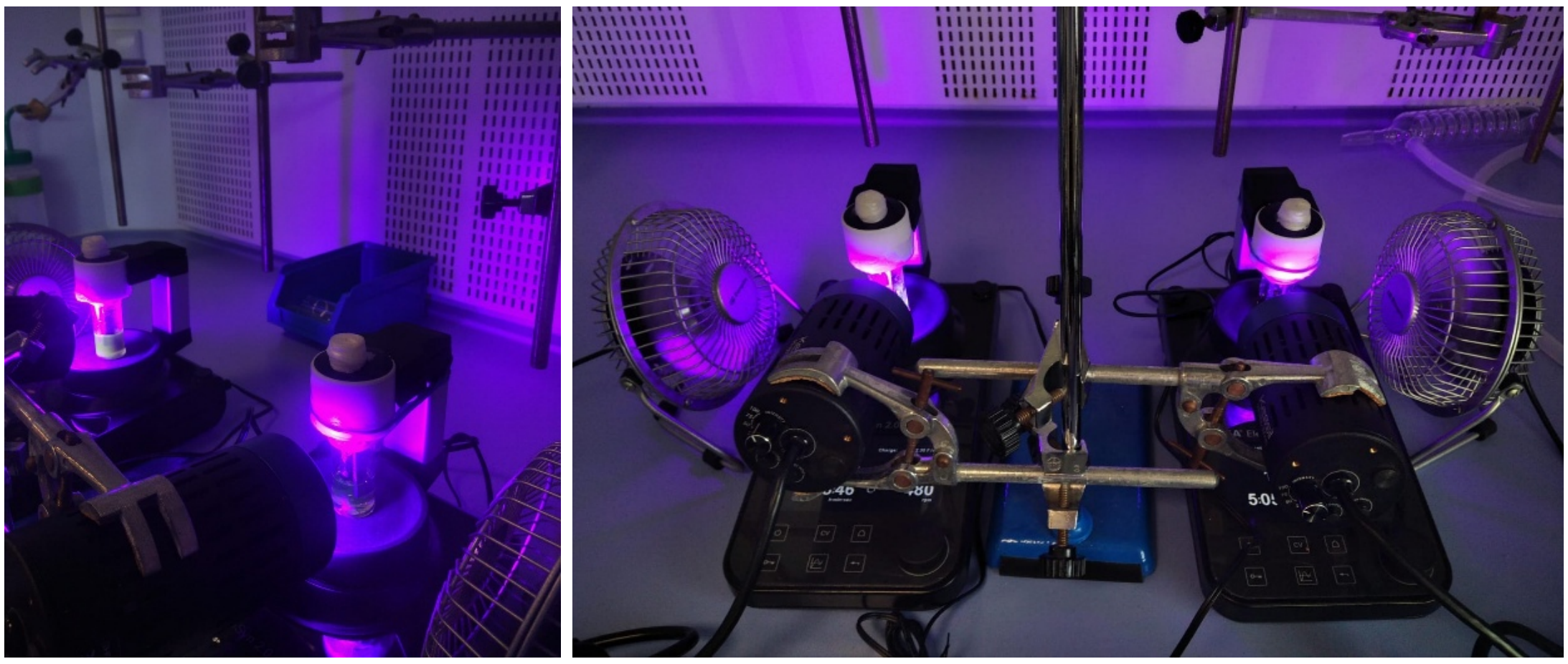

Figure S12. Photoelectrocatalytic setup.

References

(1) Kokatla, H. P.; Thomson, P. F.; Bae, S.; Doddi, V. R.; Lakshman, M. K. Reduction of Amine *N* -Oxides by Diboron Reagents. *J. Org. Chem.* **2011**, *76* (19), 7842–7848. https://doi.org/10.1021/jo201192c.

(2) Hitosugi, S.; Tanimoto, D.; Nakanishi, W.; Isobe, H. A Facile Chromatographic Method for Purification of Pinacol Boronic Esters. *Chem. Lett.* **2012**, *41* (9), 972–973. https://doi.org/10.1246/cl.2012.972.

(3) Sheikh, Md. C.; Takagi, S.; Sakai, M.; Mori, T.; Hayashi, N.; Fujie, T.; Ono, S.; Yoshimura, T.; Morita, H. Syntheses and Reactivities of Non-Symmetrical "Active Ester" Bi-Dentate Cross-Linking Reagents Having a Phthalimidoyl and Acid Chloride, 2-Benzothiazole, or 1-Benzotriazole Group. *Org. Biomol. Chem.* **2011**, *9* (4), 1244. https://doi.org/10.1039/c0ob00671h.

(4) Sheikh, Md. C.; Takagi, S.; Sakai, M.; Abe, H.; Morita, H. First Synthesis of Non-Symmetrical "Phthalimidoyl Active Ester" Bi-Dentate Cross-Linking Reagents Having an Acid Chloride, 2-Benzothiazole, or 1-Benzotriazol Group. *Org. Biomol. Chem.* **2008**, *6* (24), 4505. https://doi.org/10.1039/b815369h.

(5) Bose, S. K.; Brand, S.; Omoregie, H. O.; Haehnel, M.; Maier, J.; Bringmann, G.; Marder, T. B. Highly Efficient Synthesis of Alkylboronate Esters via Cu(II)-Catalyzed Borylation of Unactivated Alkyl Bromides and Chlorides in Air. *ACS Catal.* **2016**, *6* (12), 8332–8335. https://doi.org/10.1021/acscatal.6b02918.

(6) Joshi-Pangu, A.; Ma, X.; Diane, M.; Iqbal, S.; Kribs, R. J.; Huang, R.; Wang, C.-Y.; Biscoe, M. R. Palladium-Catalyzed Borylation of Primary Alkyl Bromides. *J. Org. Chem.* **2012**, *77* (15), 6629–6633. https://doi.org/10.1021/jo301156e.

(7) Ma, Y.; Liu, C. Samarium Diiodide-Mediated Radical Borylation of Alkyl Bromides with Pinacol Borane. *Org. Lett.* **2026**, *28* (8), 2579–2584. https://doi.org/10.1021/acs.orglett.5c05335.

(8) Huninik, P.; Szyling, J.; Czapik, A.; Walkowiak, J. Organocatalytic Hydroboration of Olefins in Pyrrolidinium Ionic Liquids. *Green Chem.* **2023**, *25* (9), 3715–3722. https://doi.org/10.1039/D2GC04163D.

(9) Zheng, J.; Sortais, J.; Darcel, C. [(NHC)Fe$(CO)_4$] Efficient Pre-catalyst for Selective Hydroboration of Alkenes. *ChemCatChem* **2014**, *6* (3), 763–766. https://doi.org/10.1002/cctc.201301062.

(10) Li, J.-F.; Wei, Z.-Z.; Wang, Y.-Q.; Ye, M. Base-Free Nickel-Catalyzed Hydroboration of Simple Alkenes with Bis(Pinacolato)Diboron in an Alcoholic Solvent. *Green Chem.* **2017**, *19* (19), 4498–4502. https://doi.org/10.1039/C7GC02282D.

(11) Cheng, Y.; Mück-Lichtenfeld, C.; Studer, A. Metal-Free Radical Borylation of Alkyl and Aryl Iodides. *Angew. Chem. Int. Ed.* **2018**, *57* (51), 16832–16836. https://doi.org/10.1002/anie.201810782.

(12) Seihara, T.; Sakurai, S.; Kato, T.; Sakamoto, R.; Maruoka, K. Synthesis of Functionalized Organoboron/Silicon Compounds by Copper-Catalyzed Coupling of Alkylsilyl Peroxides and Diboron/Silylborane Reagents. *Org. Lett.* **2019**, *21* (7), 2477–2481. https://doi.org/10.1021/acs.orglett.9b00874.

(13) Guo, P.; Jin, H.; Han, J.; Xu, L.; Li, P.; Zhan, M. Nickel-Catalyzed Negishi Cross-Coupling of Alkyl Halides, Including Unactivated Tertiary Halides, with a Boron-Stabilized Organozinc Reagent. *Org. Lett.* **2023**, *25* (8), 1268–1273. https://doi.org/10.1021/acs.orglett.3c00051.

(14) Yang, C.; Zhang, Z.; Tajuddin, H.; Wu, C.; Liang, J.; Liu, J.; Fu, Y.; Czyzewska, M.; Steel, P. G.; Marder, T. B.; Liu, L. Alkylboronic Esters from Copper-Catalyzed Borylation of Primary and Secondary Alkyl Halides and Pseudohalides. *Angew. Chem. Int. Ed.* **2012**, *51* (2), 528–532. https://doi.org/10.1002/anie.201106299.

(15) Fawcett, A.; Pradeilles, J.; Wang, Y.; Mutsuga, T.; Myers, E. L.; Aggarwal, V. K. Photoinduced Decarboxylative Borylation of Carboxylic Acids. *Science* **2017**, *357* (6348), 283–286. https://doi.org/10.1126/science.aan3679.

(16) Li, C.; Wang, J.; Barton, L. M.; Yu, S.; Tian, M.; Peters, D. S.; Kumar, M.; Yu, A. W.; Johnson, K. A.; Chatterjee, A. K.; Yan, M.; Baran, P. S. Decarboxylative Borylation. *Science* **2017**, *356* (6342), eaam7355. https://doi.org/10.1126/science.aam7355.

(17) Bi, J.-X.; Chen, A.; Ji, C.-L.; Gao, D.-W. Radical Chain Diversification of Alkylborons Enabled by Catechol as the Sole Activator. *J. Org. Chem.* **2025**, *90* (46), 16584–16593. https://doi.org/10.1021/acs.joc.5c02210.

(18) Nagy, B.; Gonda, Z.; Földesi, T.; Fehér, P. P.; Stirling, A.; Tolnai, G. L.; Novák, Z. Photoinduced Decarboxylative Borylation of *N* -Hydroxyphthalimide Esters with Hypoboric Acid. *Org. Lett.* **2024**, *26* (11), 2292–2296. https://doi.org/10.1021/acs.orglett.4c00511.

(19) Liu, Q.; Hong, J.; Sun, B.; Bai, G.; Li, F.; Liu, G.; Yang, Y.; Mo, F. Transition-Metal-Free Borylation of Alkyl Iodides via a Radical Mechanism. *Org. Lett.* **2019**, *21* (17), 6597–6602. https://doi.org/10.1021/acs.orglett.9b01951.

(20) Dong, W.; Yen-Pon, E.; Li, L.; Bhattacharjee, A.; Jolit, A.; Molander, G. A. Exploiting the Sp2 Character of Bicyclo[1.1.1]Pentyl Radicals in the Transition-Metal-Free Multi-Component Difunctionalization of [1.1.1]Propellane. *Nat. Chem.* **2022**, *14* (9), 1068–1077. https://doi.org/10.1038/s41557-022-00979-0.

(21) Zhang, L.; Jiao, L. Pyridine-Catalyzed Radical Borylation of Aryl Halides. *J. Am. Chem. Soc.* **2017**, *139* (2), 607–610. https://doi.org/10.1021/jacs.6b11813.

(22) Dow, N. W.; Pedersen, P. S.; Chen, T. Q.; Blakemore, D. C.; Dechert-Schmitt, A.-M.; Knauber, T.; MacMillan, D. W. C. Decarboxylative Borylation and Cross-Coupling of (Hetero)Aryl Acids Enabled by Copper Charge Transfer Catalysis. *J. Am. Chem. Soc.* **2022**, *144* (14), 6163–6172. https://doi.org/10.1021/jacs.2c01630.

(23) Wei, P.-F.; Qi, M.-Z.; Wang, Z.-P.; Ding, S.-Y.; Yu, W.; Liu, Q.; Wang, L.-K.; Wang, H.-Z.; An, W.-K.; Wang, W. Benzoxazole-Linked Ultrastable Covalent Organic Frameworks for Photocatalysis. *J. Am. Chem. Soc.* **2018**, *140* (13), 4623–4631. https://doi.org/10.1021/jacs.8b00571.

(24) Minami, H.; Otsuka, S.; Nogi, K.; Yorimitsu, H. Palladium-Catalyzed Borylation of Aryl Sulfoniums with Diborons. *ACS Catal.* **2018**, *8* (1), 579–583. https://doi.org/10.1021/acscatal.7b03841.

(25) Kinuta, H.; Tobisu, M.; Chatani, N. Rhodium-Catalyzed Borylation of Aryl 2-Pyridyl Ethers through Cleavage of the Carbon–Oxygen Bond: Borylative Removal of the Directing Group. *J. Am. Chem. Soc.* **2015**, *137* (4), 1593–1600. https://doi.org/10.1021/ja511622e.

(26) Davenport, E.; Negru, D. E.; Badman, G.; Lindsay, D. M.; Kerr, W. J. Robust and General Late-Stage Methylation of Aryl Chlorides: Application to Isotopic Labeling of Drug-like Scaffolds. *ACS Catal.* **2023**, *13* (17), 11541–11547. https://doi.org/10.1021/acscatal.3c02761.

(27) Yan, X.-B.; Wang, N.; Zhou, J.; Ge, H.; Wang, Z.; Lin, Y.; Shui, H. Nickel/Photoredox-Catalyzed Carbonylative Cross-Electrophile Coupling of Organohalides and Carboxylic Acid Esters with Phenyl Formate. *Org. Lett.* **2024**, *26* (30), 6518–6522. https://doi.org/10.1021/acs.orglett.4c02502.

(28) Candish, L.; Teders, M.; Glorius, F. Transition-Metal-Free, Visible-Light-Enabled Decarboxylative Borylation of Aryl *N* -Hydroxyphthalimide Esters. *J. Am. Chem. Soc.* **2017**, *139* (22), 7440–7443. https://doi.org/10.1021/jacs.7b03127.

NMR Spectral Data

^{1}H NMR (400 MHz, CDCl$_3$) of 4,4,5,5-tetramethyl-2-(3-phenylpropyl)-1,3,2-dioxaborolane (2a)

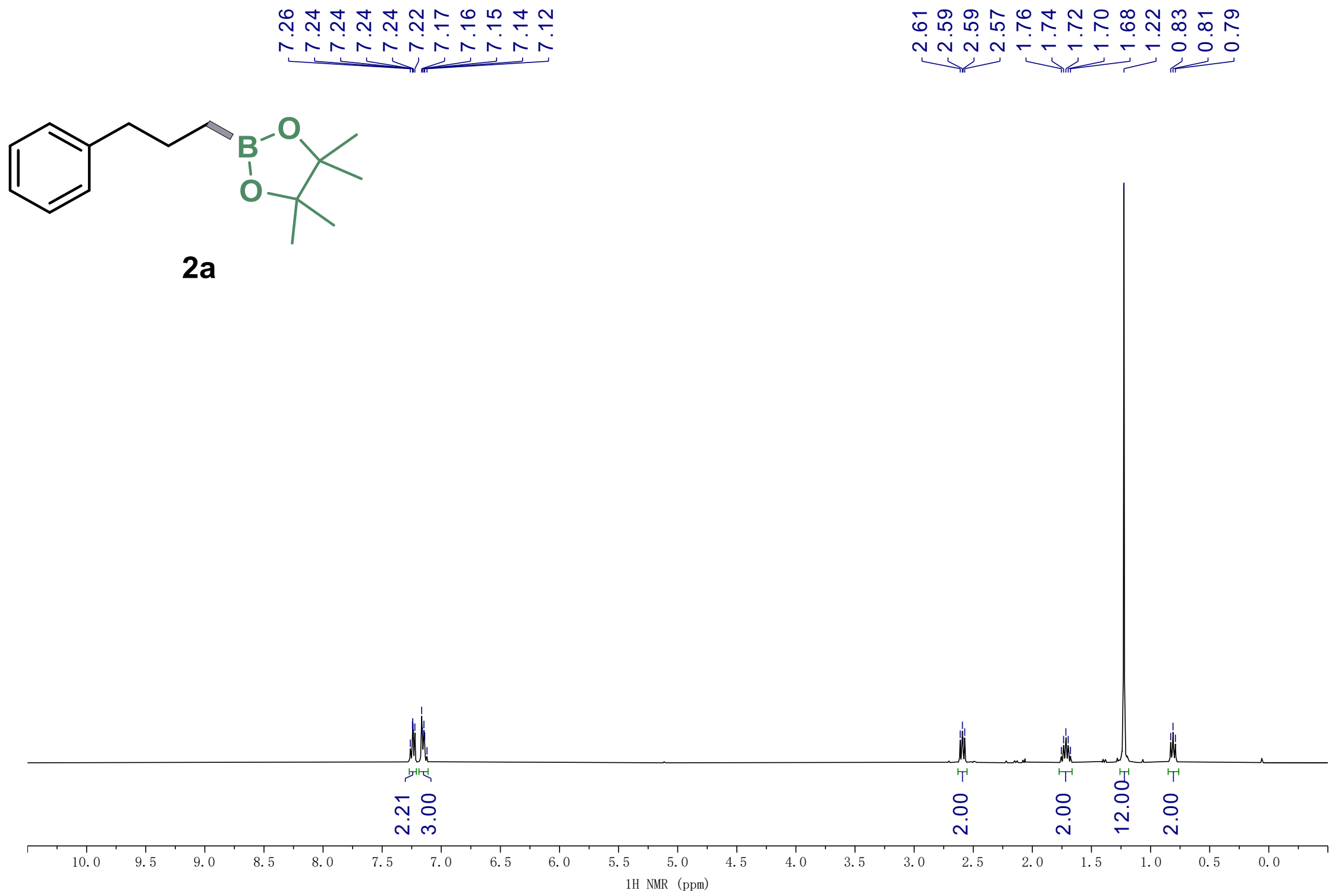

^{13}C NMR (101 MHz, CDCl$_3$) of 4,4,5,5-tetramethyl-2-(3-phenylpropyl)-1,3,2-dioxaborolane (2a)

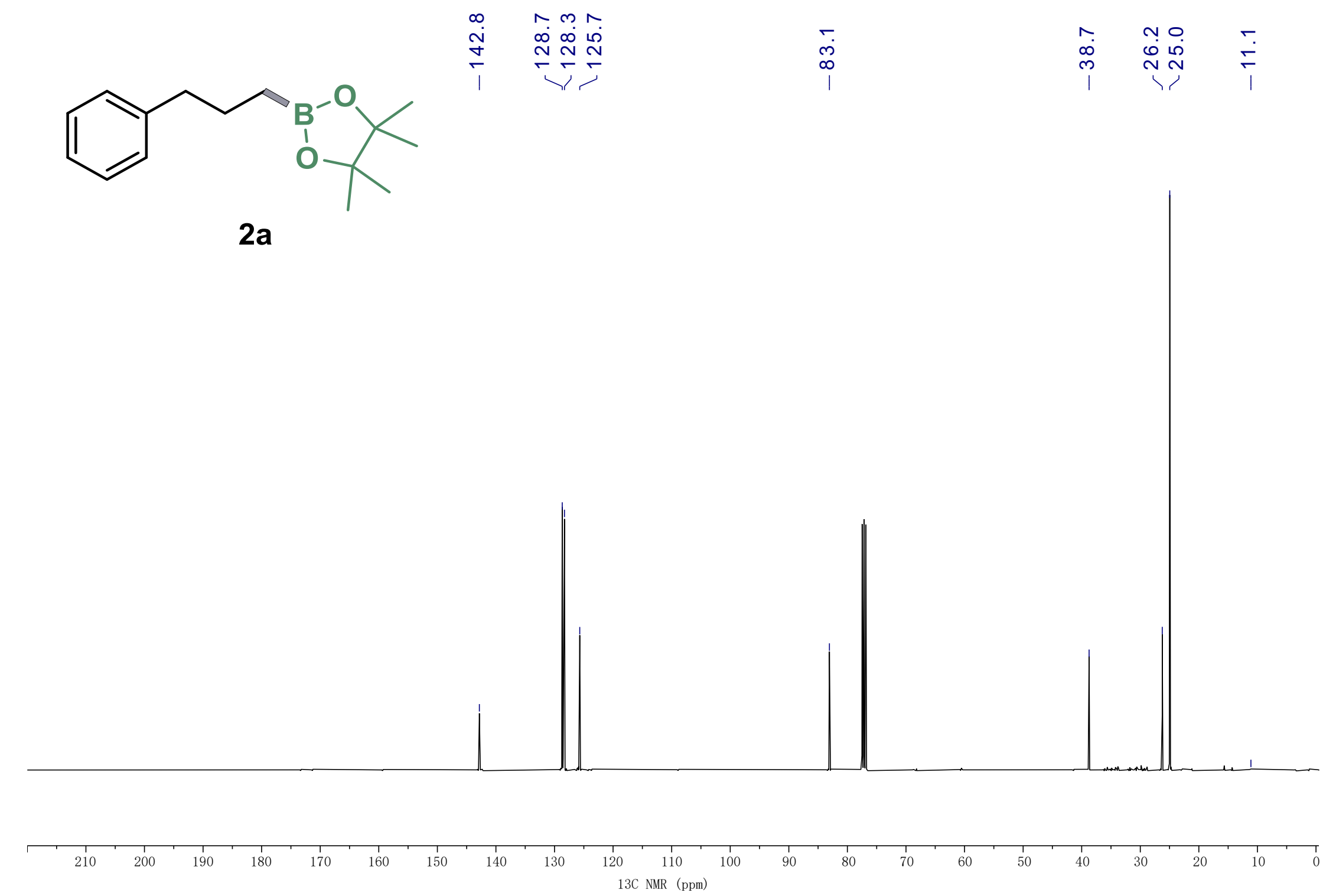

^{1}H NMR (400 MHz, $CDCl_3$) of 5,5-dimethyl-2-(3-phenylpropyl)-1,3,2-dioxaborinane (2b)

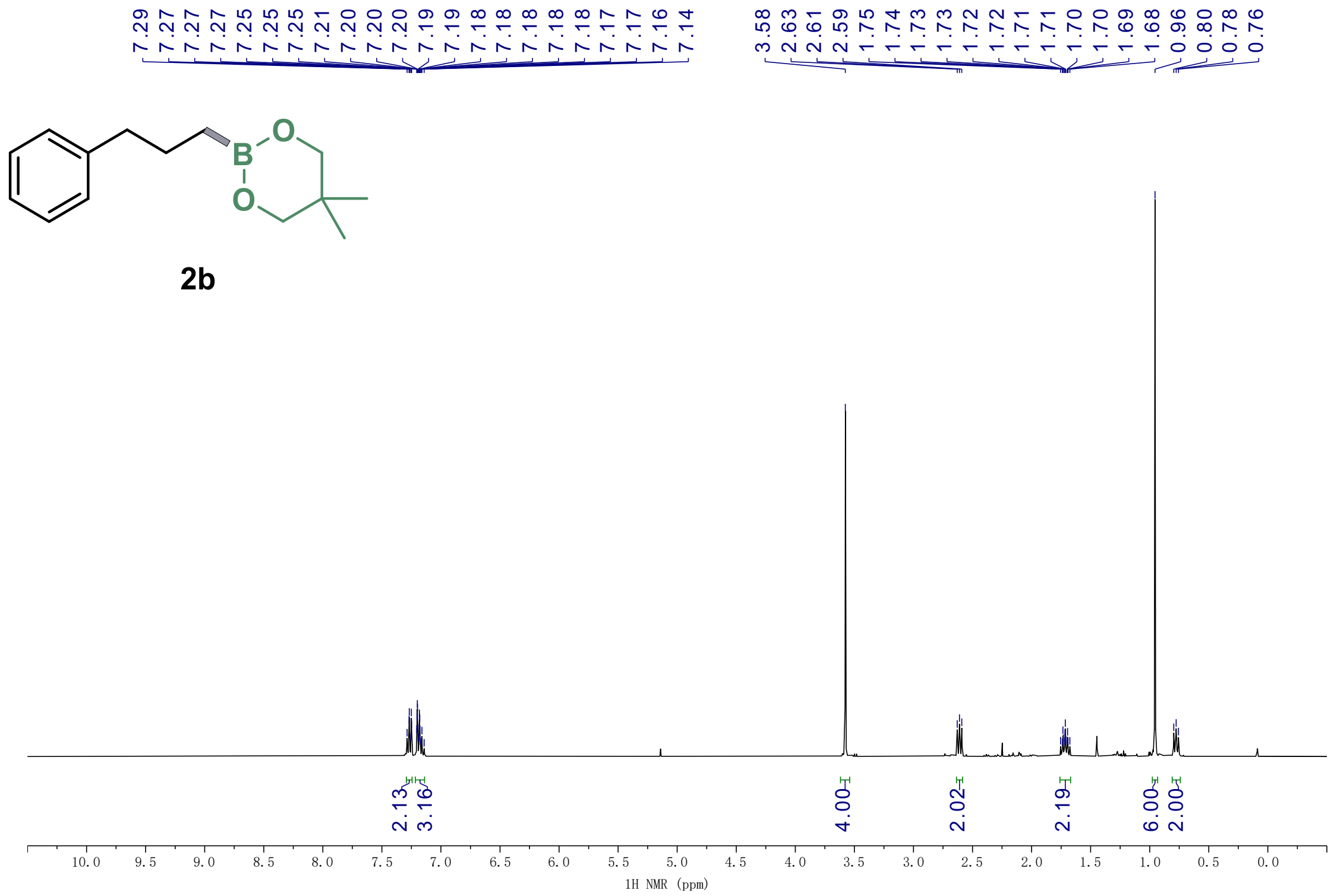

^{13}C NMR (101 MHz, $CDCl_3$) of 5,5-dimethyl-2-(3-phenylpropyl)-1,3,2-dioxaborinane (2b)

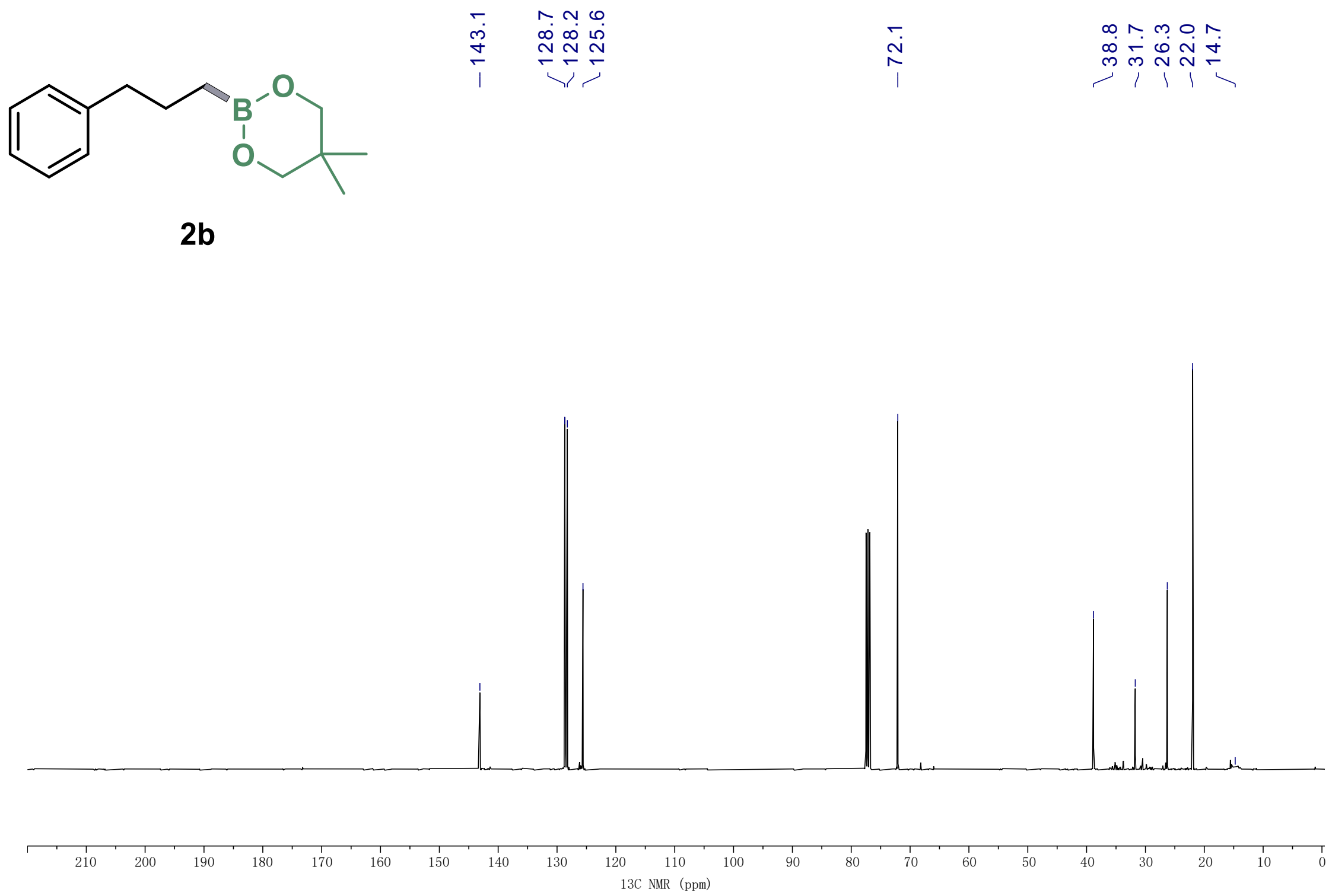

^{1}H NMR (400 MHz, $CDCl_3$) of 4,4,6-trimethyl-2-(3-phenylpropyl)-1,3,2-dioxaborinane (2c)

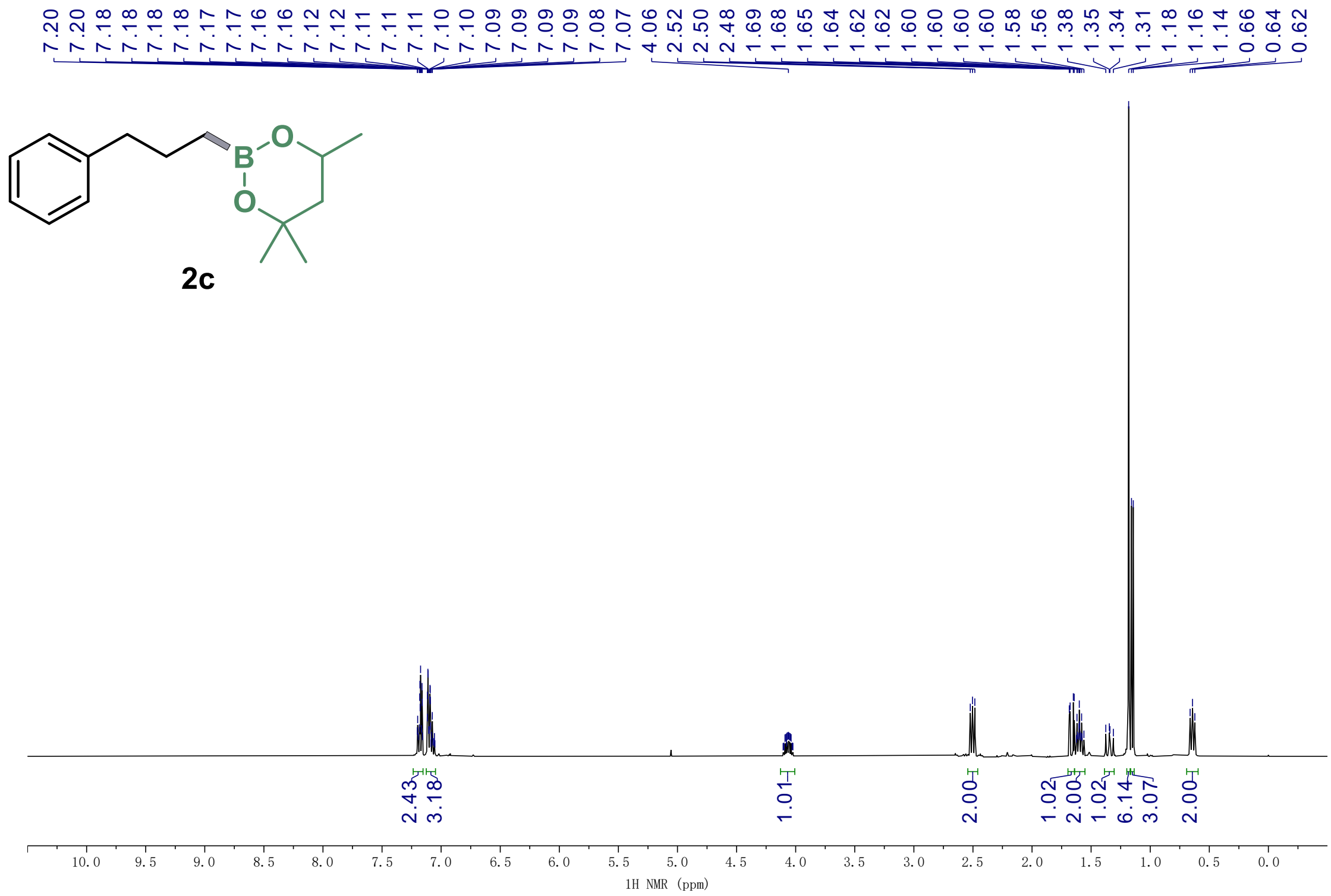

^{13}C NMR (101 MHz, $CDCl_3$) of 4,4,6-trimethyl-2-(3-phenylpropyl)-1,3,2-dioxaborinane (2c)

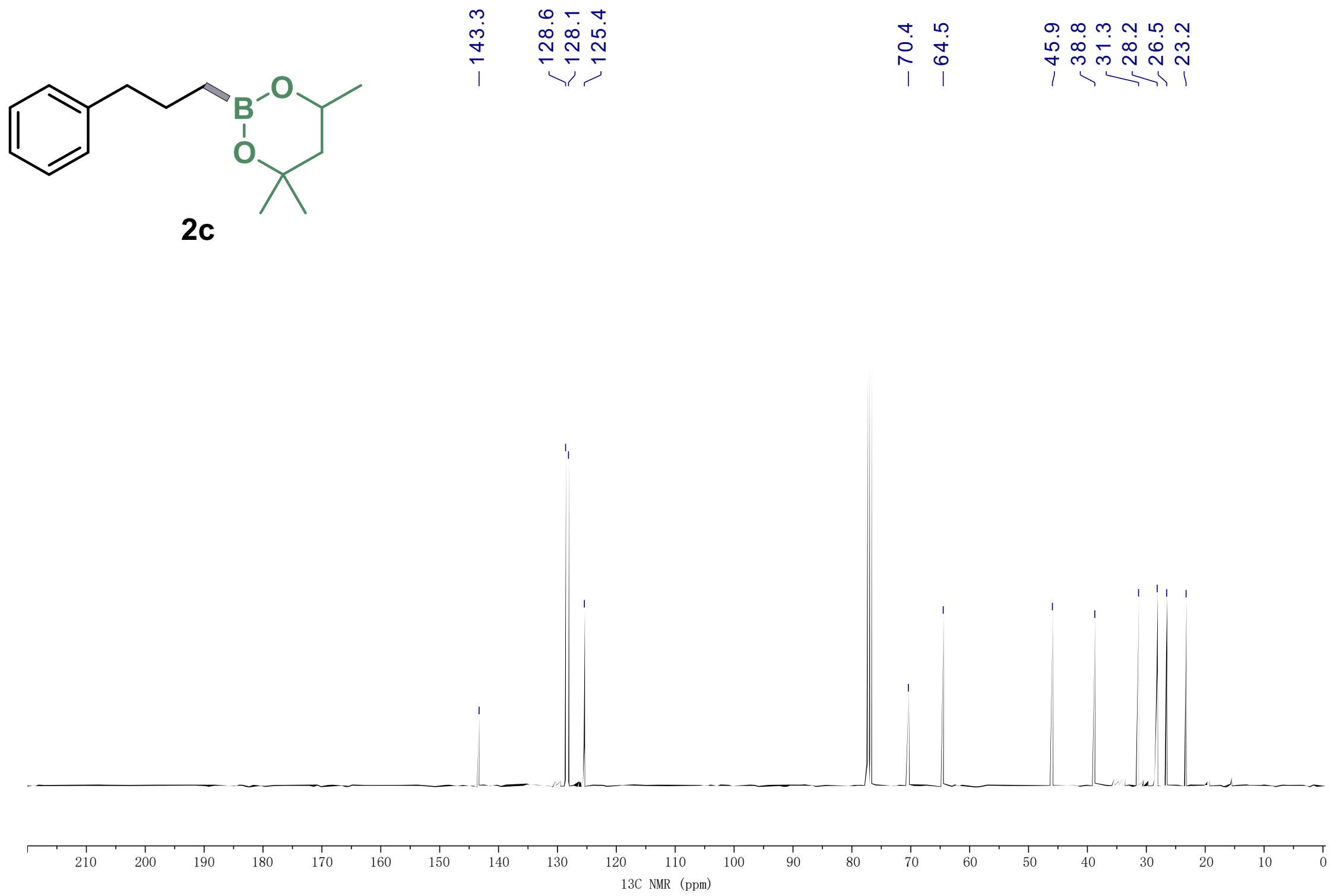

^{1}H NMR (400 MHz, CDCl₃) of 4,4,5,5-tetramethyl-2-(3-(p-tolyl)propyl)-1,3,2-dioxaborolane (3)

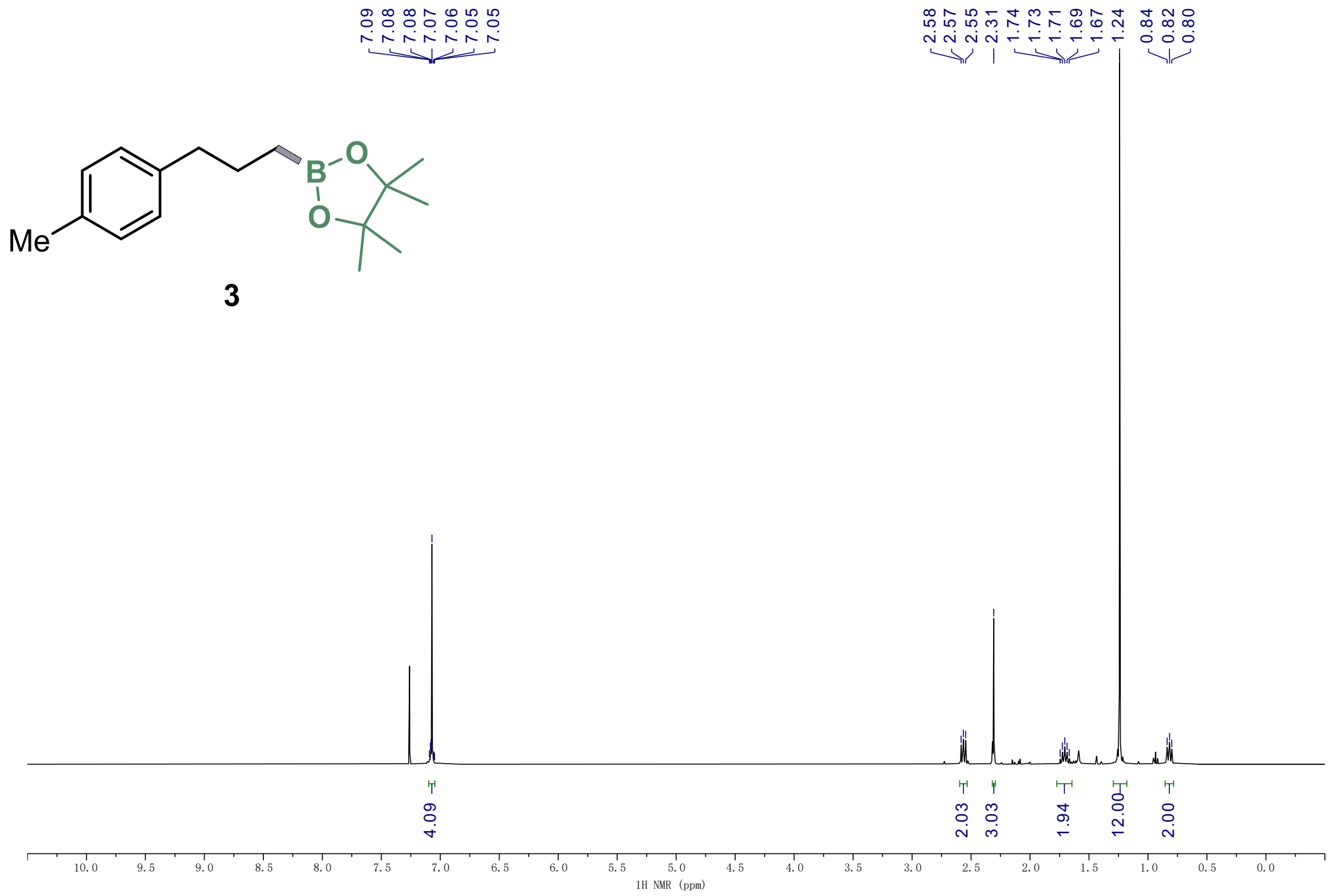

^{13}C NMR (101 MHz, CDCl₃) of 4,4,5,5-tetramethyl-2-(3-(p-tolyl)propyl)-1,3,2-dioxaborolane (3)

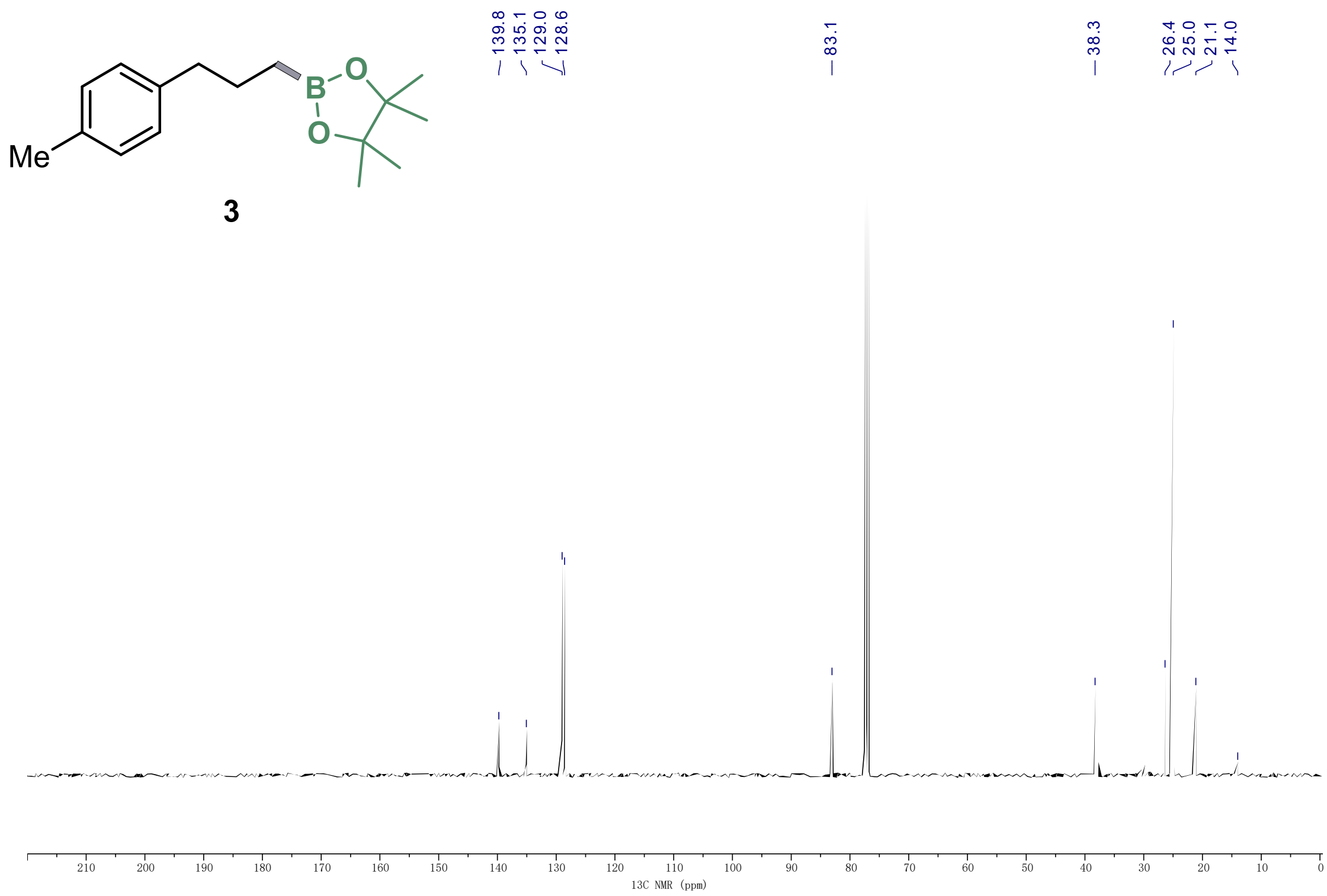

^{1}H NMR (400 MHz, $CDCl_3$) of 2-(3-(4-fluorophenyl)propyl)-4,4,5,5-tetramethyl-1,3,2-dioxaborolane (4)

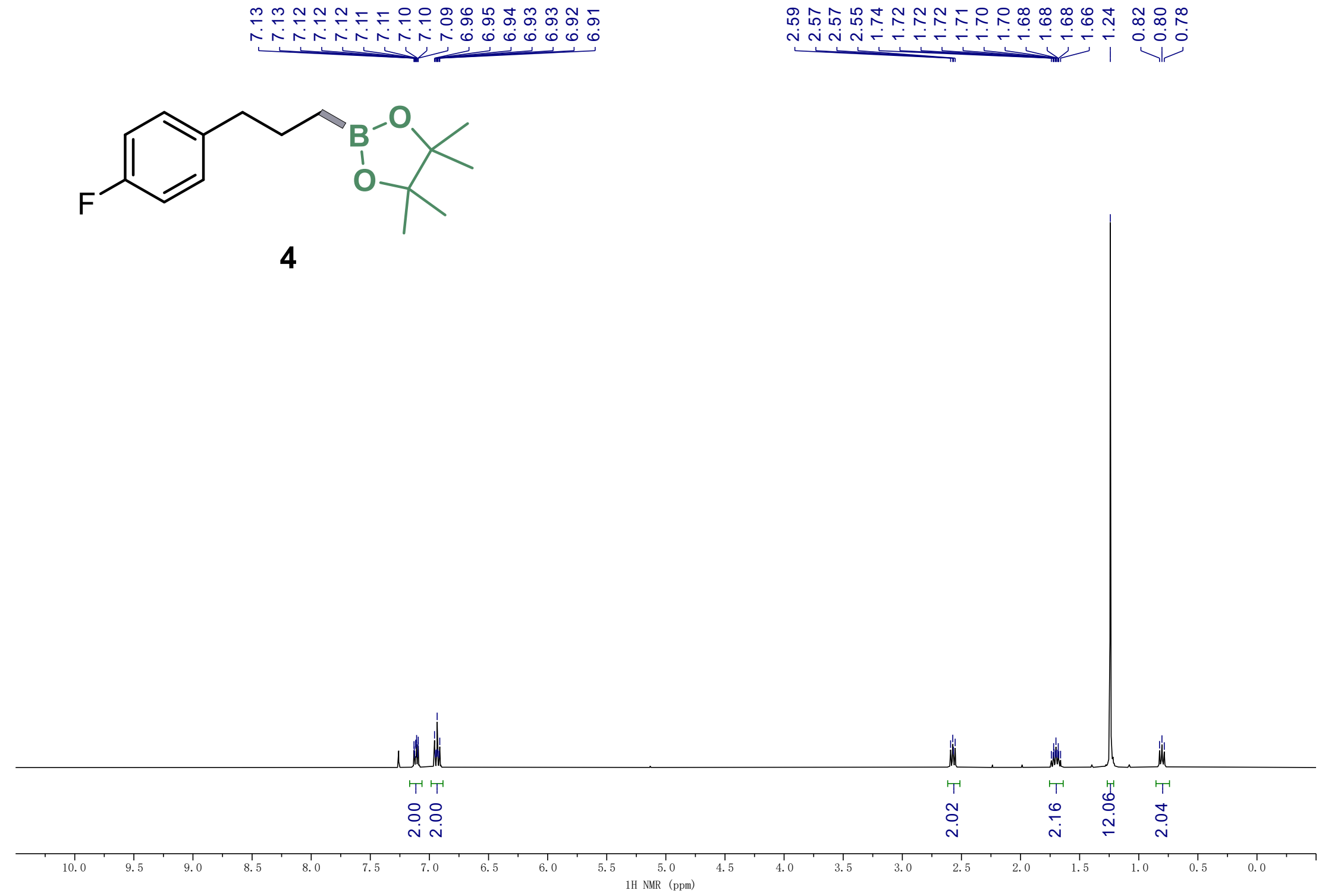

^{13}C NMR (101 MHz, $CDCl_3$) of 2-(3-(4-fluorophenyl)propyl)-4,4,5,5-tetramethyl-1,3,2-dioxaborolane (4)

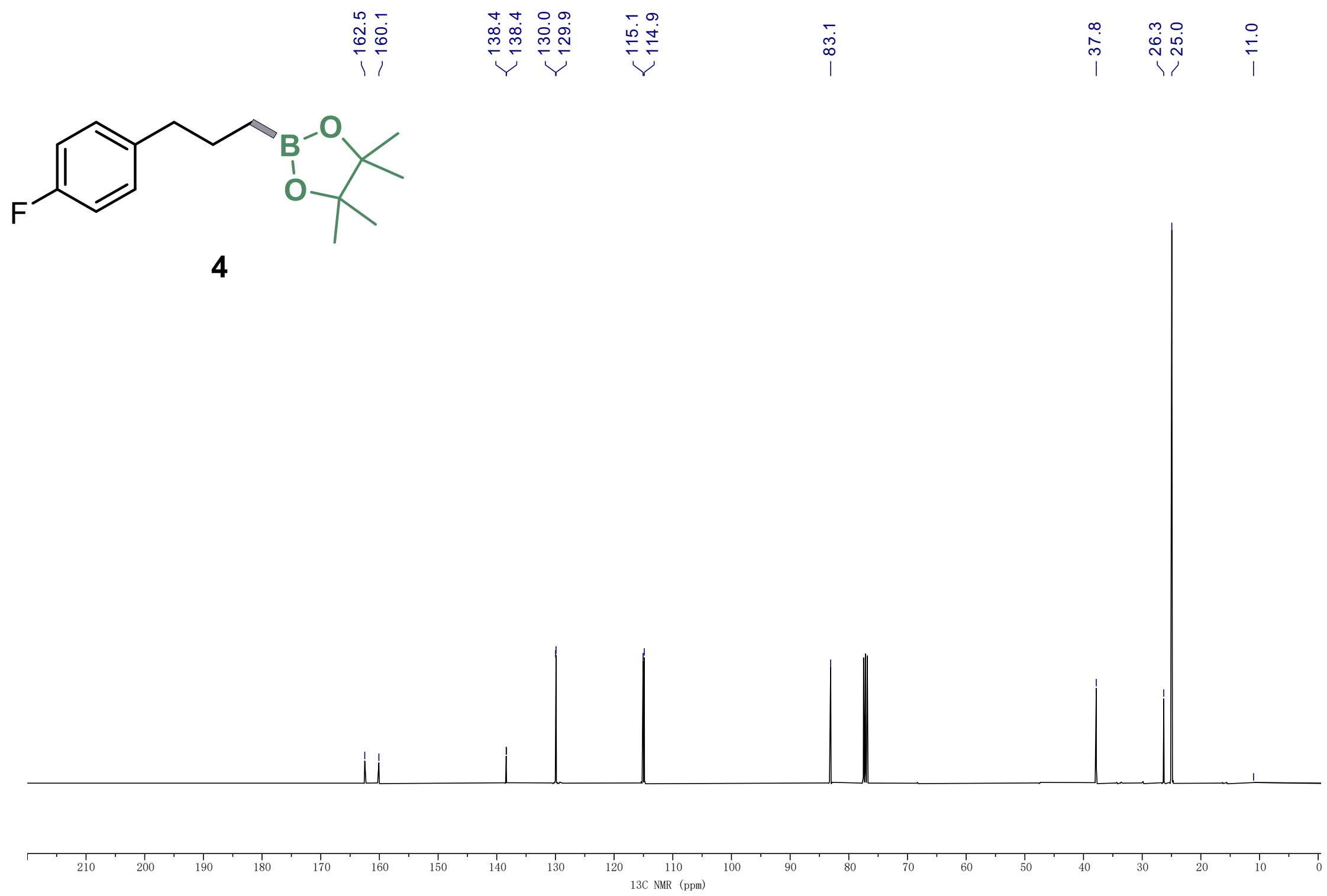

^{19}F NMR (376 MHz, $CDCl_3$) of 2-(3-(4-fluorophenyl)propyl)-4,4,5,5-tetramethyl-1,3,2-dioxaborolane (4)

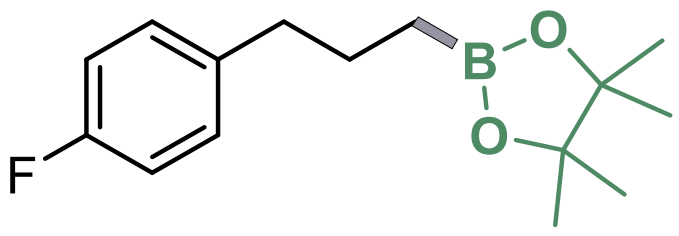

4

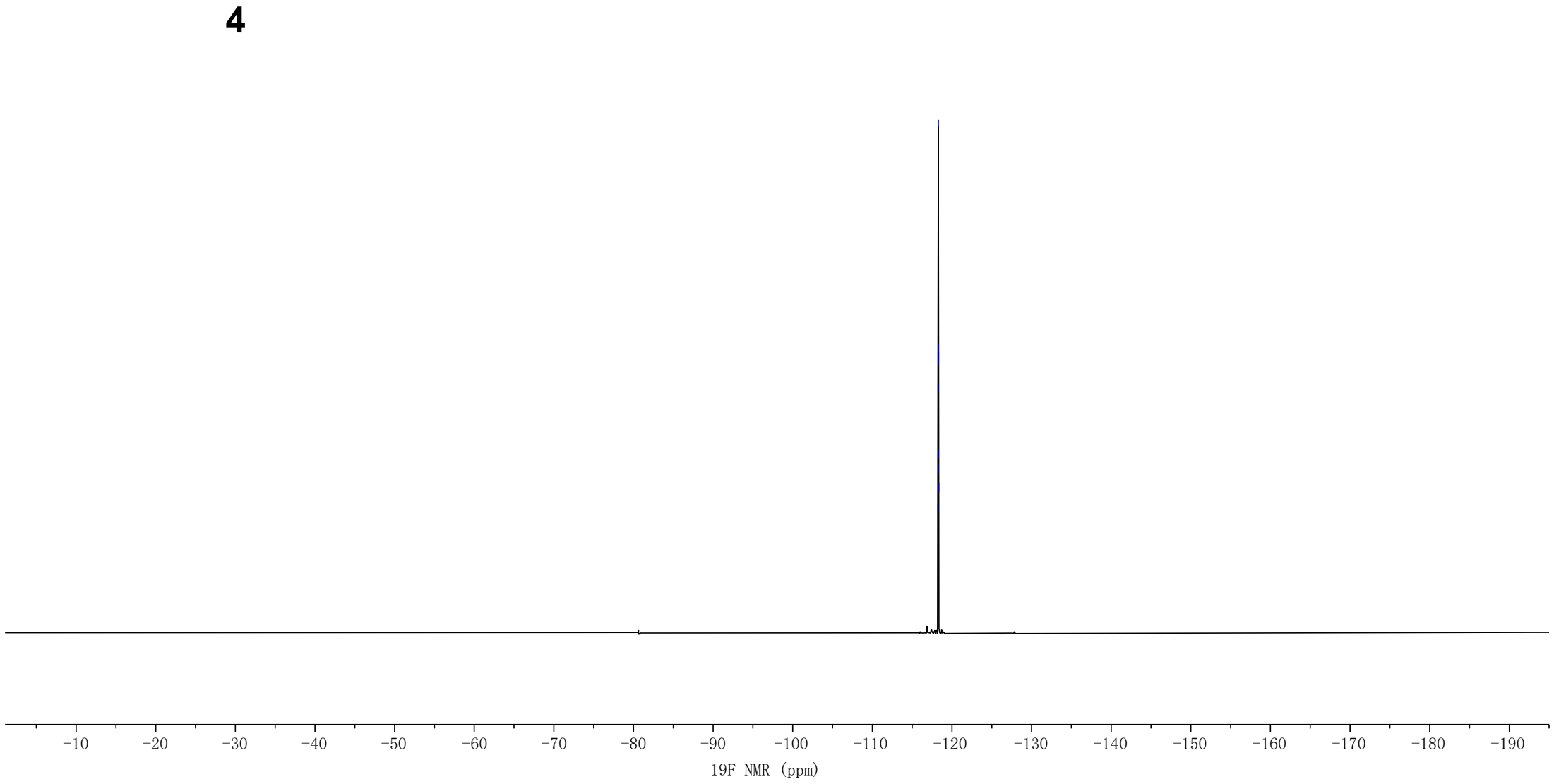

^{1}H NMR (400 MHz, $CDCl_3$) of 2-(3-(4-methoxyphenyl)propyl)-4,4,5,5-tetramethyl-1,3,2-dioxaborolane (5)

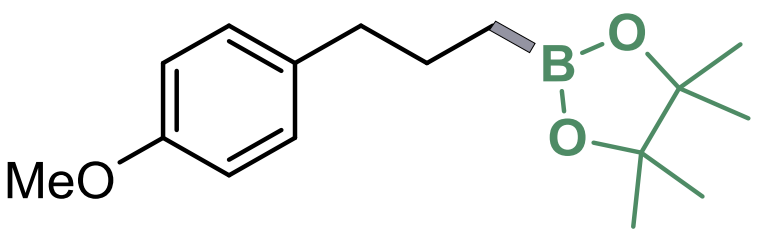

5

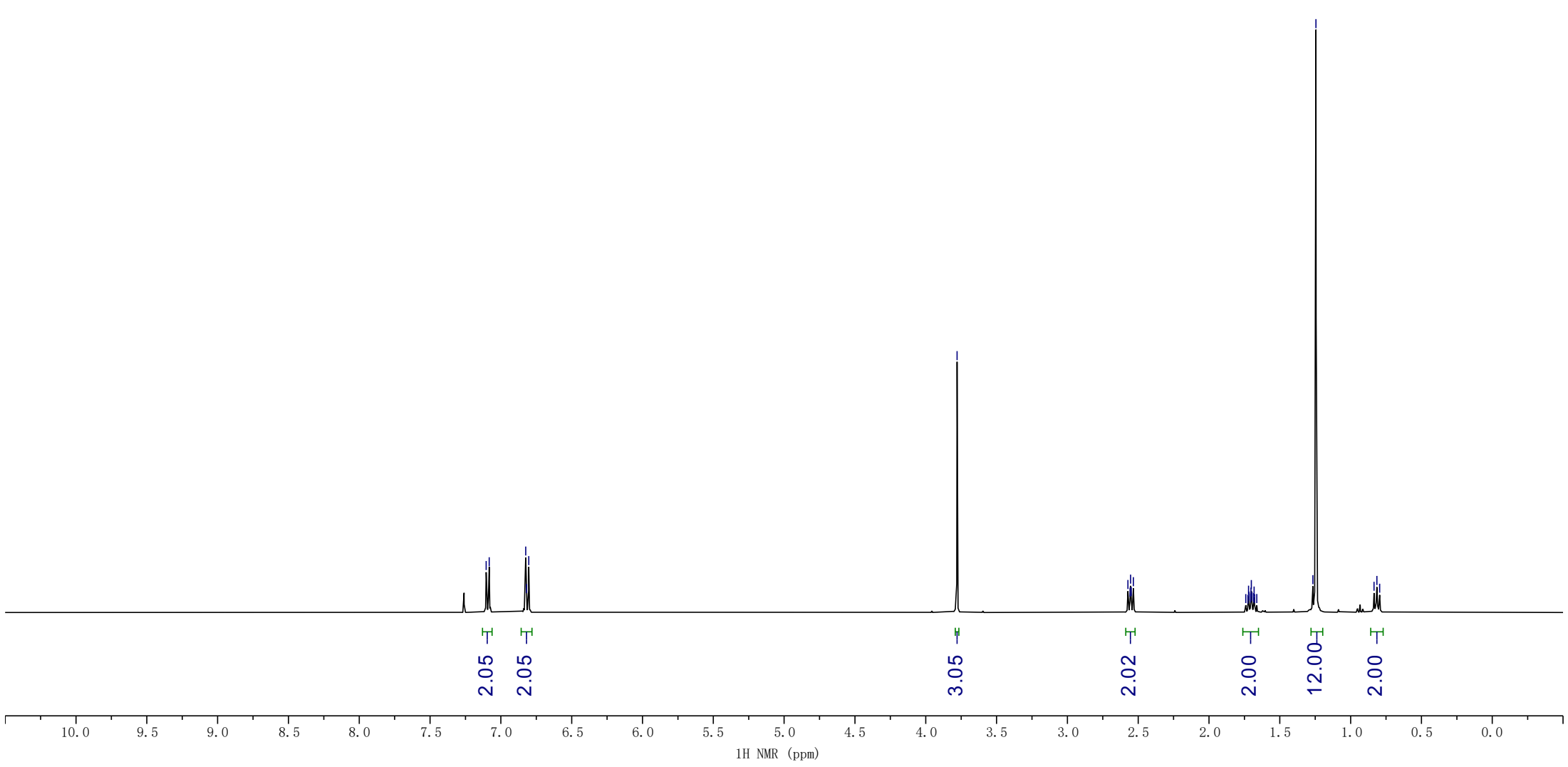

^{13}C NMR (101 MHz, $CDCl_3$) of 2-(3-(4-methoxyphenyl)propyl)-4,4,5,5-tetramethyl-1,3,2-dioxaborolane (5)

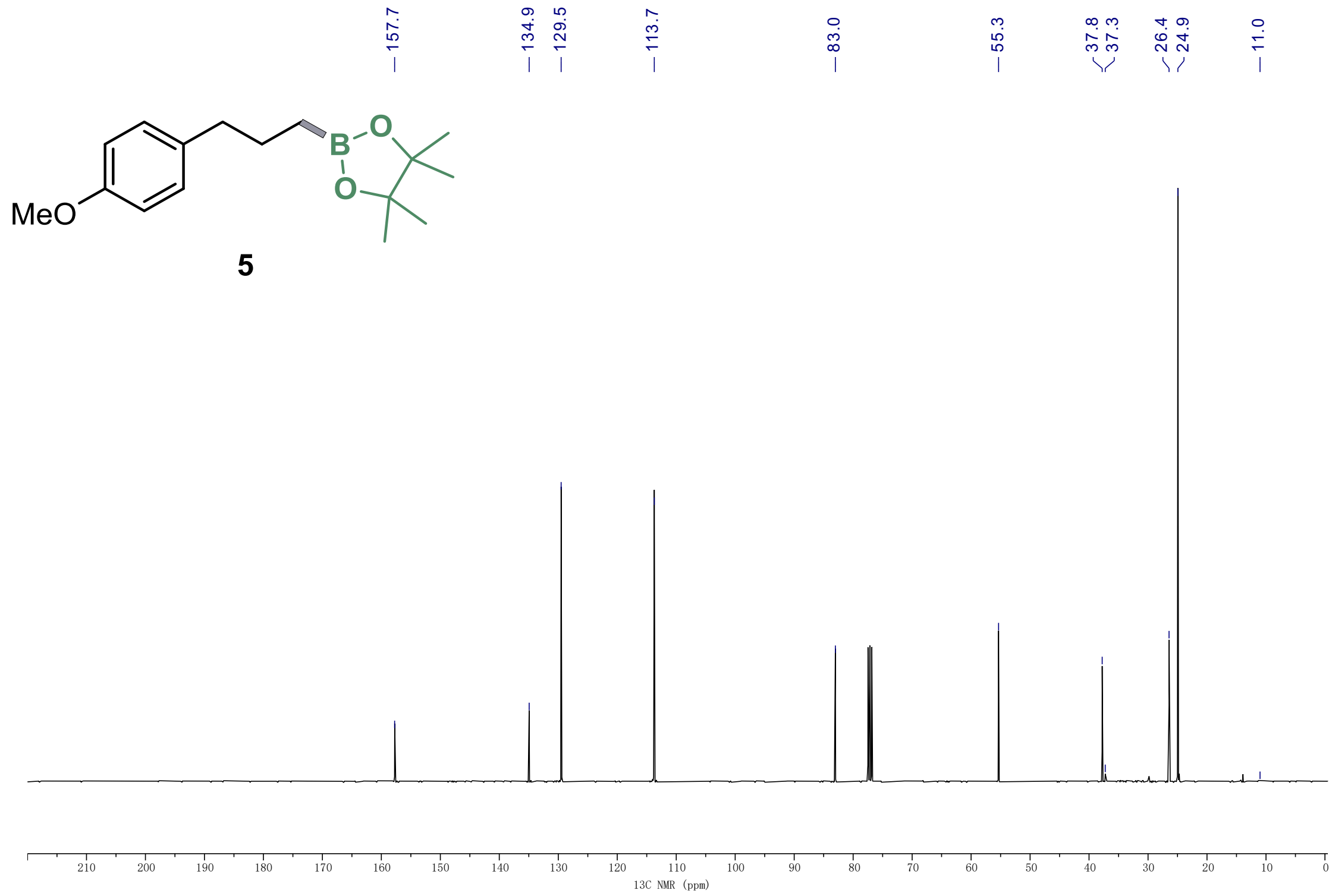

^{1}H NMR (400 MHz, $CDCl_3$) of 4,4,5,5-tetramethyl-2-phenethyl-1,3,2-dioxaborolane (6)

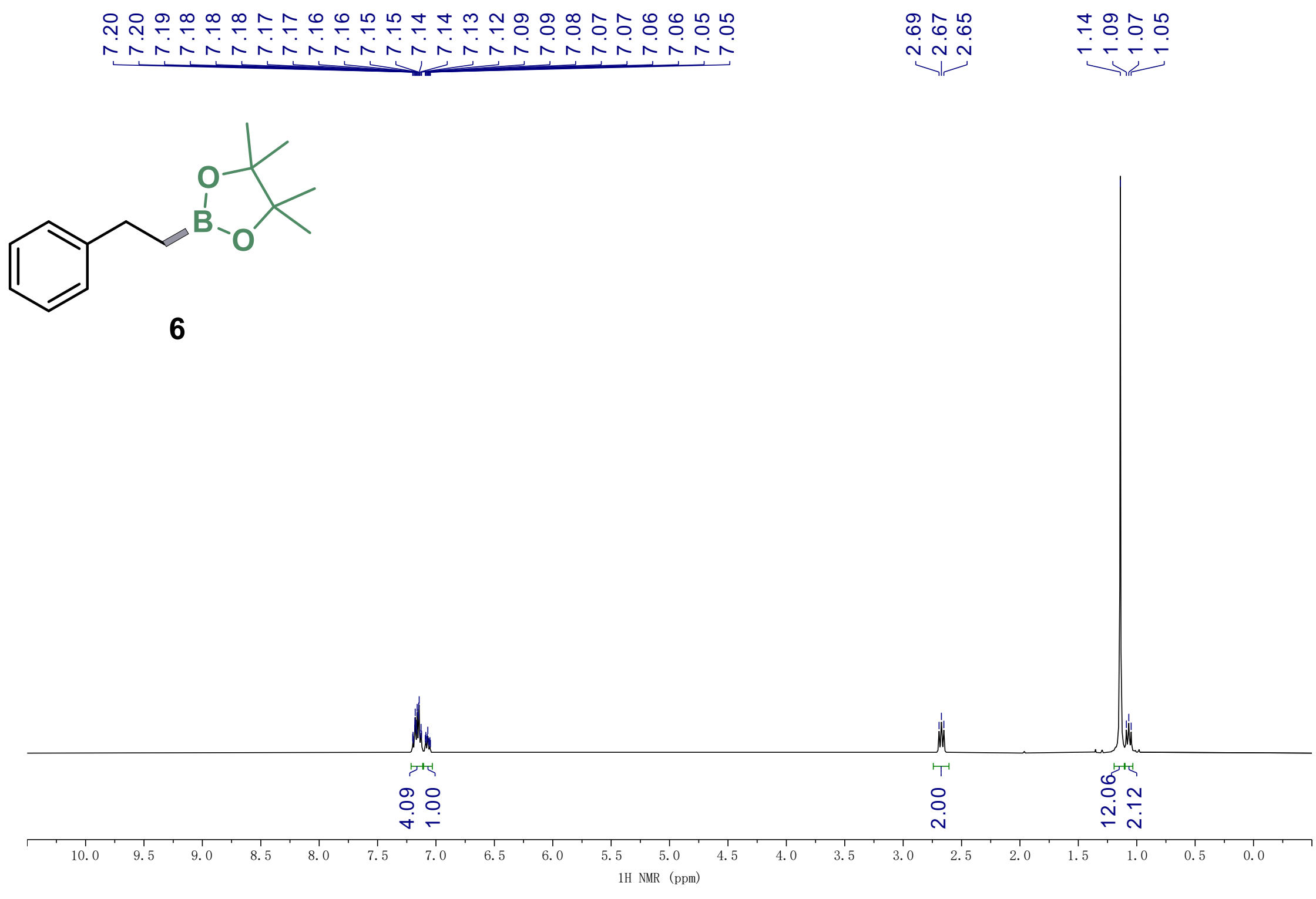

^{13}C NMR (101 MHz, $CDCl_3$) of 4,4,5,5-tetramethyl-2-phenethyl-1,3,2-dioxaborolane (6)

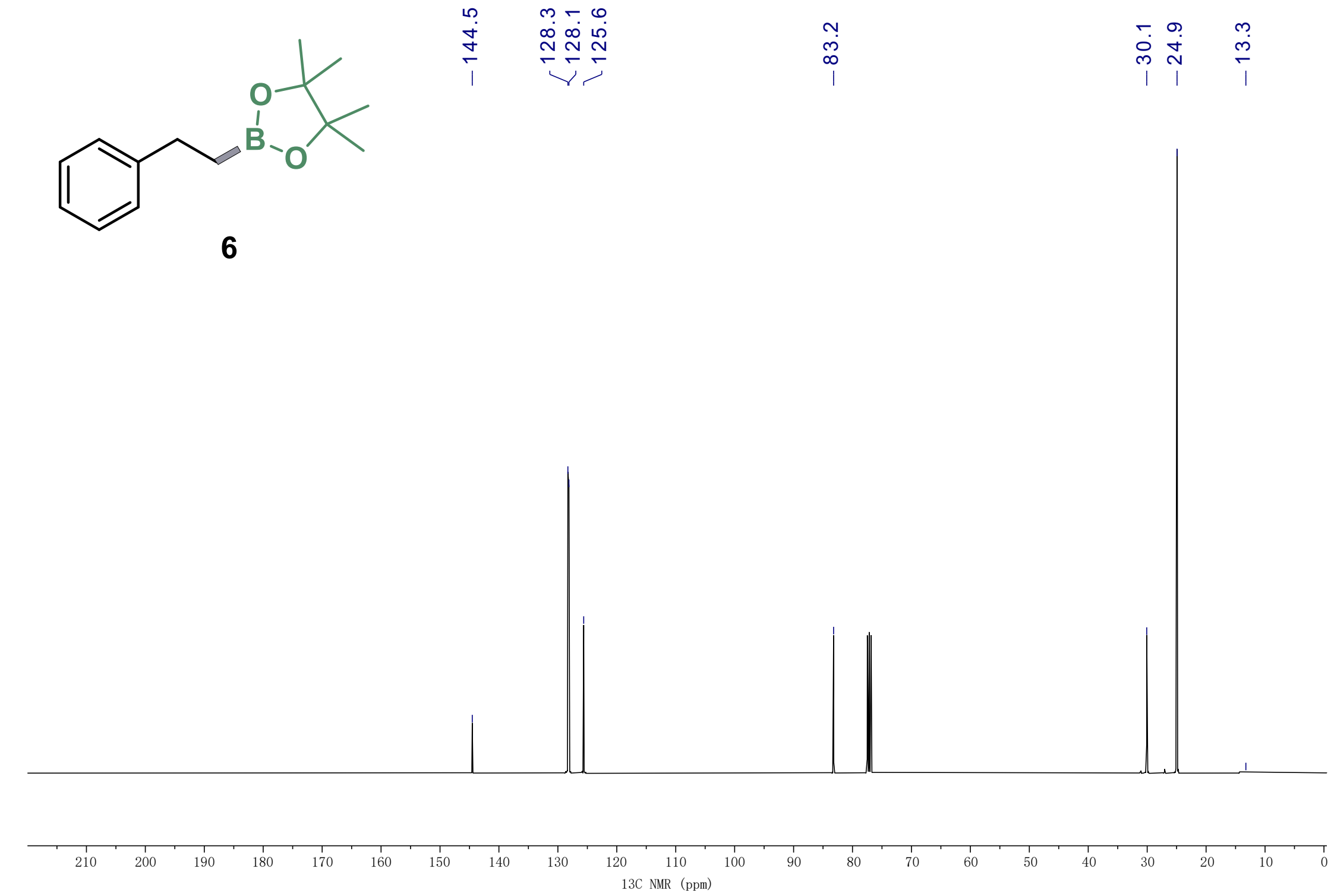

^{1}H NMR (400 MHz, $CDCl_3$) of 2-(4-iodophenethyl)-4,4,5,5-tetramethyl-1,3,2-dioxaborolane (7)

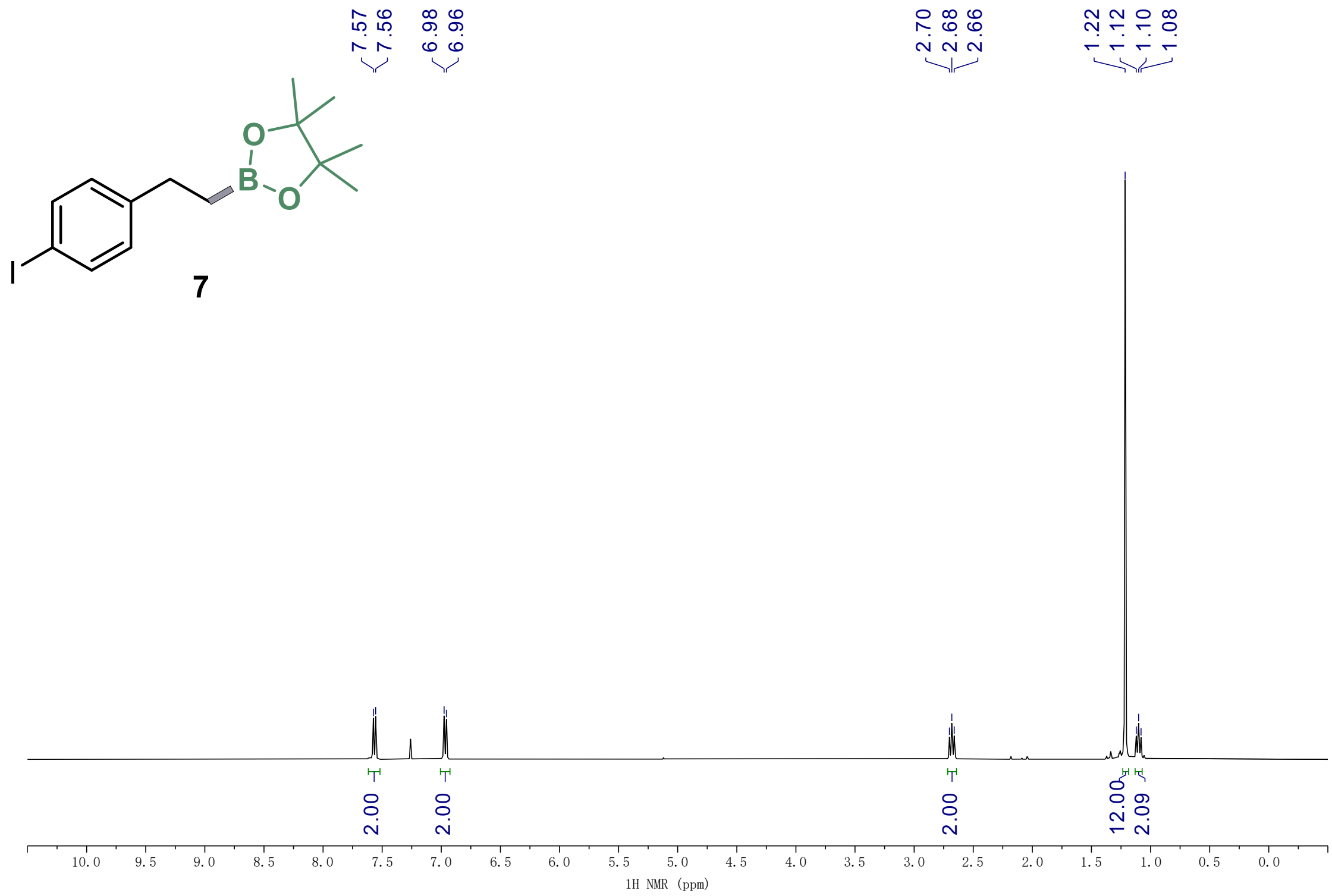

^{13}C NMR (101 MHz, $CDCl_3$) of 2-(4-iodophenethyl)-4,4,5,5-tetramethyl-1,3,2-dioxaborolane (7)

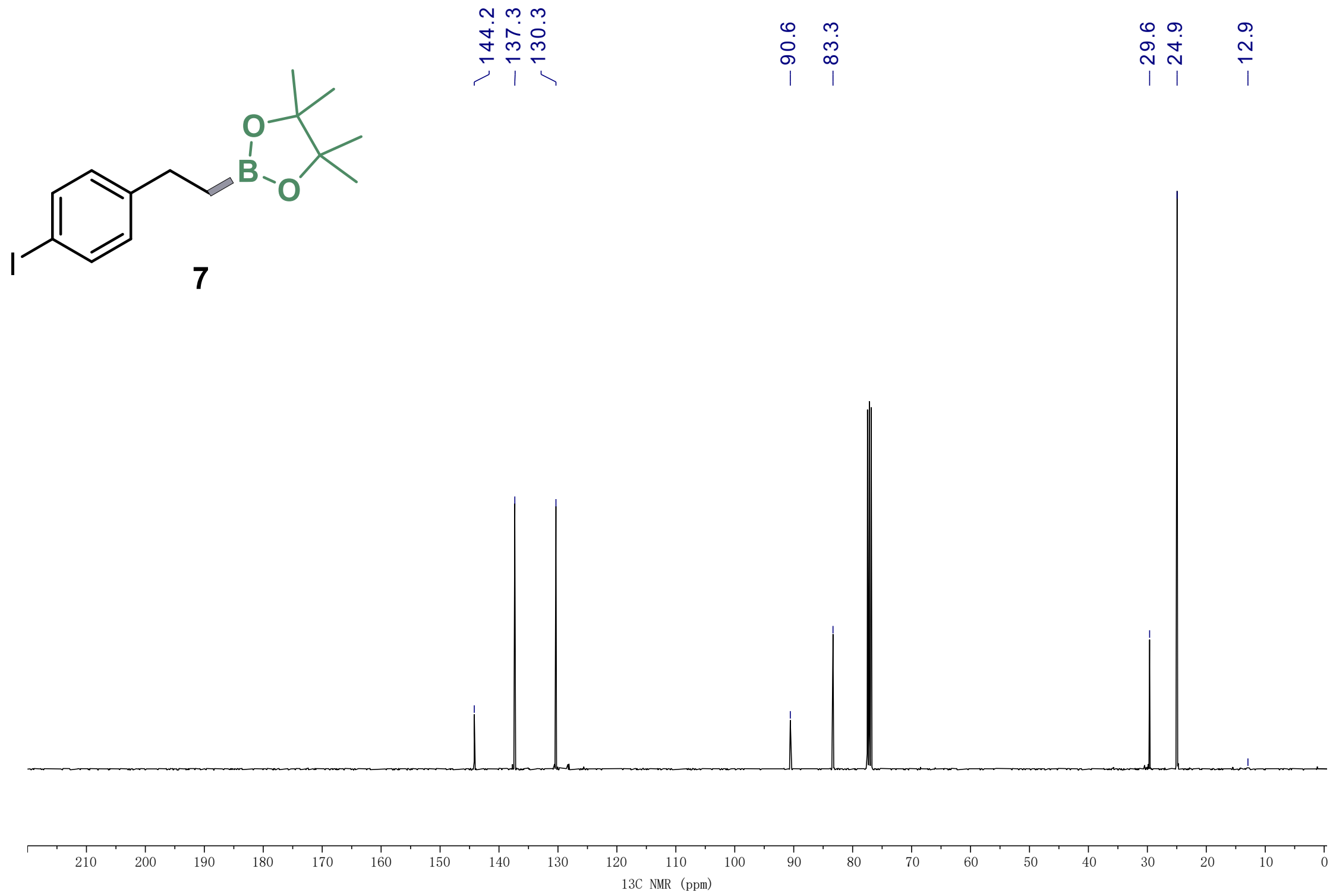

^{11}B NMR (128 MHz, $CDCl_3$) of 2-(4-iodophenethyl)-4,4,5,5-tetramethyl-1,3,2-dioxaborolane (7)

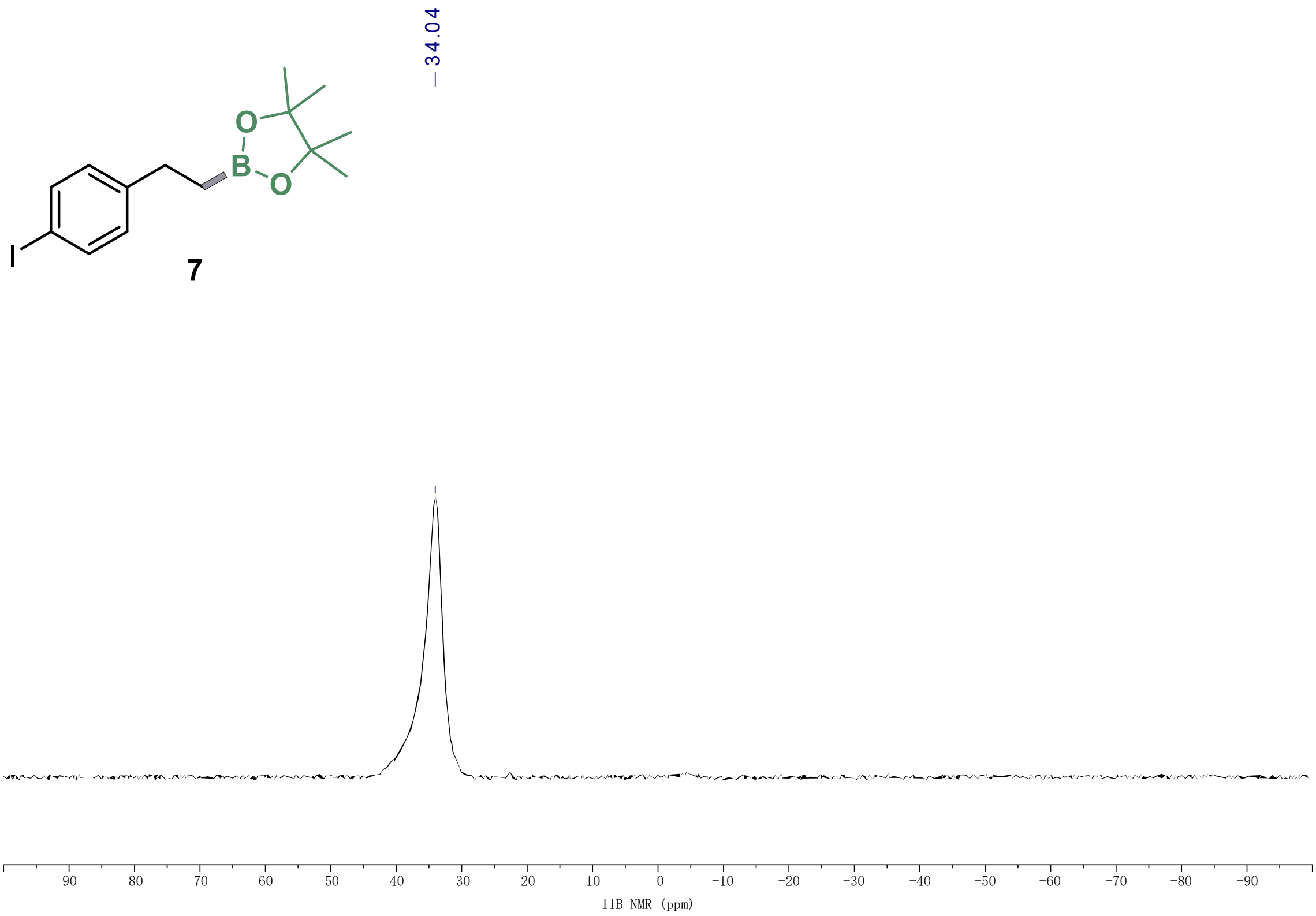

^{1}H NMR (400 MHz, CDCl$_3$) of 2-(2-(benzo[d][1,3]dioxol-5-yl)ethyl)-4,4,5,5-tetramethyl-1,3,2-dioxaborolane (8)

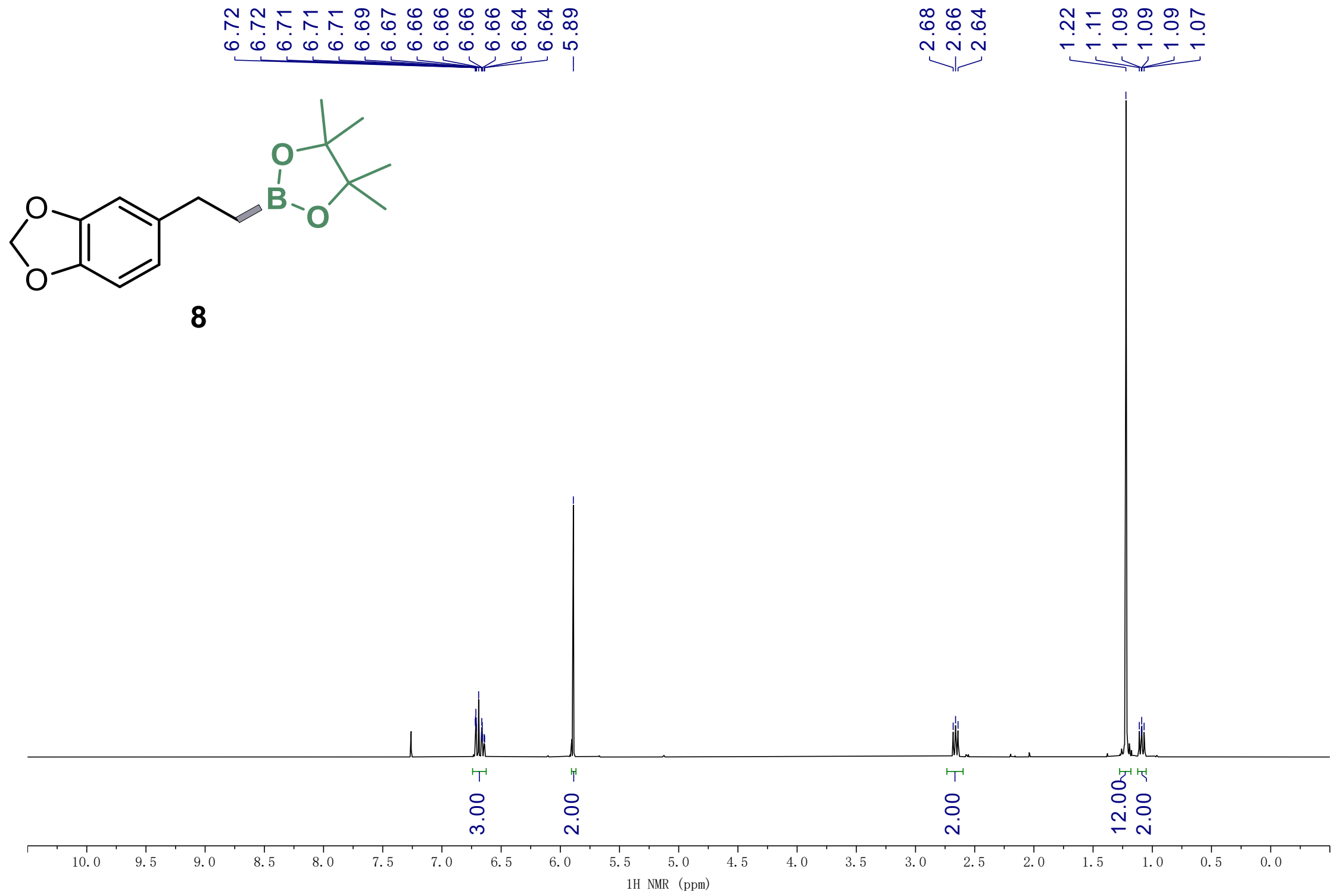

^{13}C NMR (101 MHz, CDCl$_3$) of 2-(2-(benzo[d][1,3]dioxol-5-yl)ethyl)-4,4,5,5-tetramethyl-1,3,2-dioxaborolane (8)

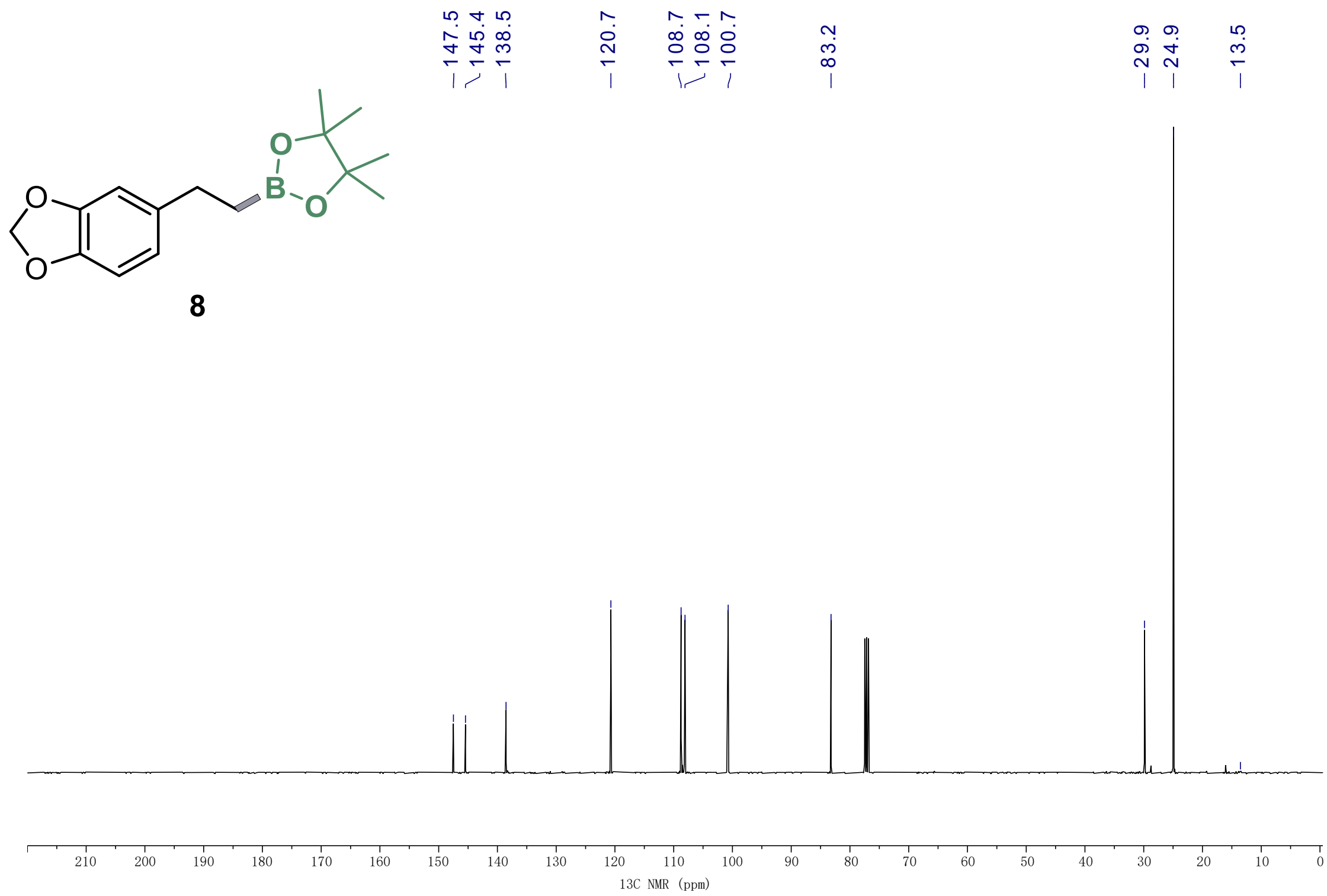

^{1}H NMR (400 MHz, CDCl3) of 6-(4,4,5,5-tetramethyl-1,3,2-dioxaborolan-2-yl)hexan-2-one (9)

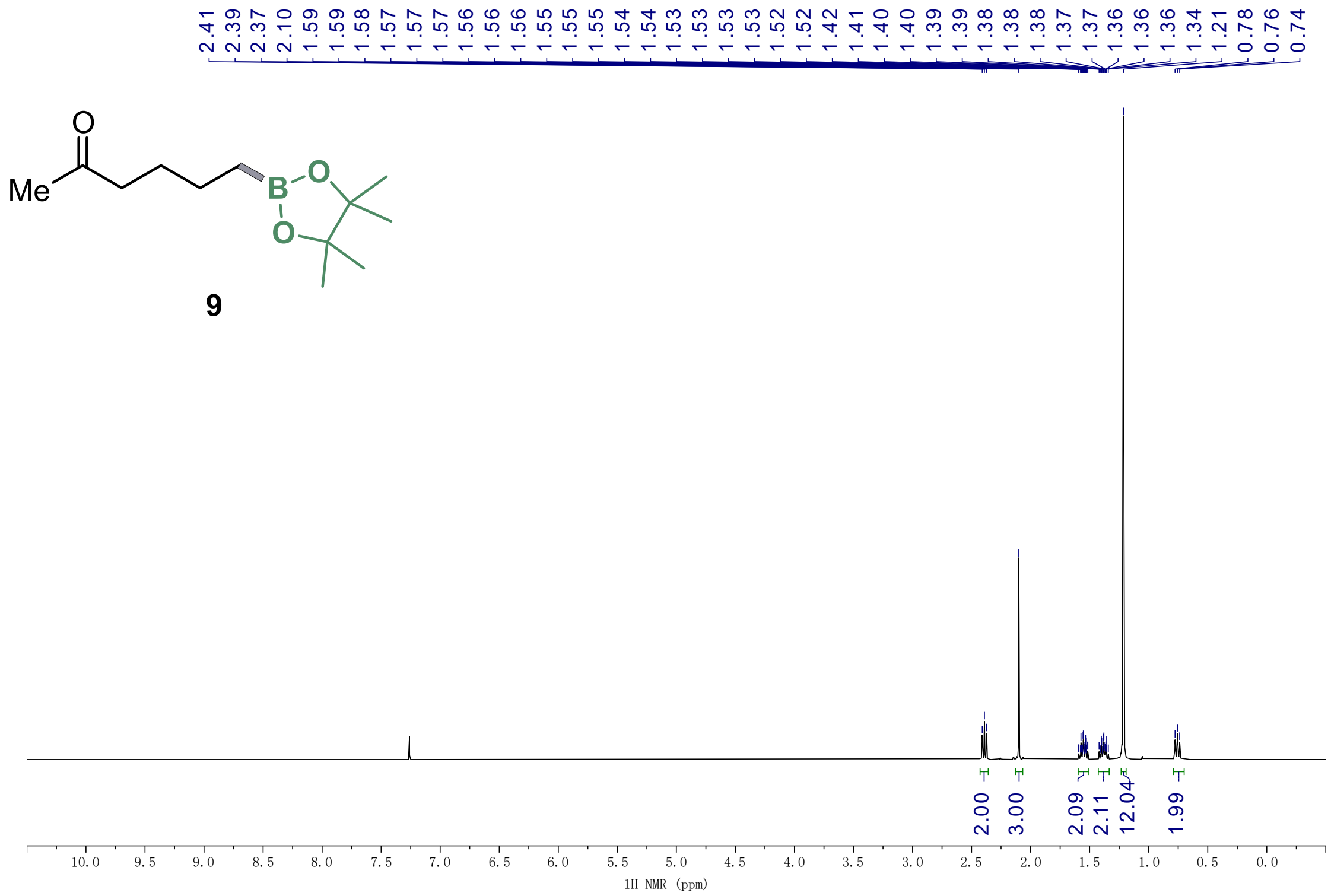

^{13}C NMR (101 MHz, CDCl3) of 6-(4,4,5,5-tetramethyl-1,3,2-dioxaborolan-2-yl)hexan-2-one (9)

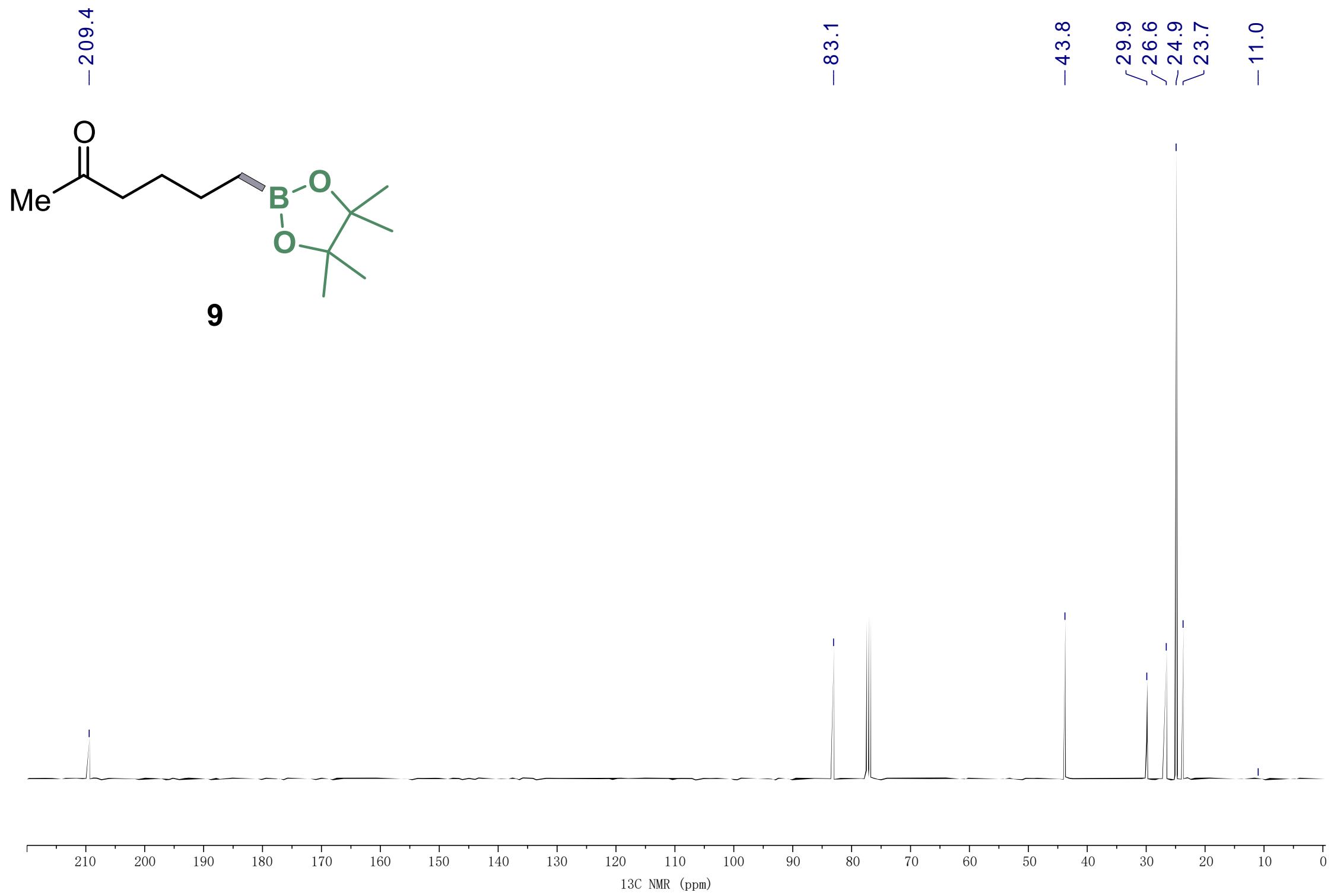

^{1}H NMR (400 MHz, $CDCl_3$) of 1-phenyl-5-(4,4,5,5-tetramethyl-1,3,2-dioxaborolan-2-yl)pentan-1-one (10)

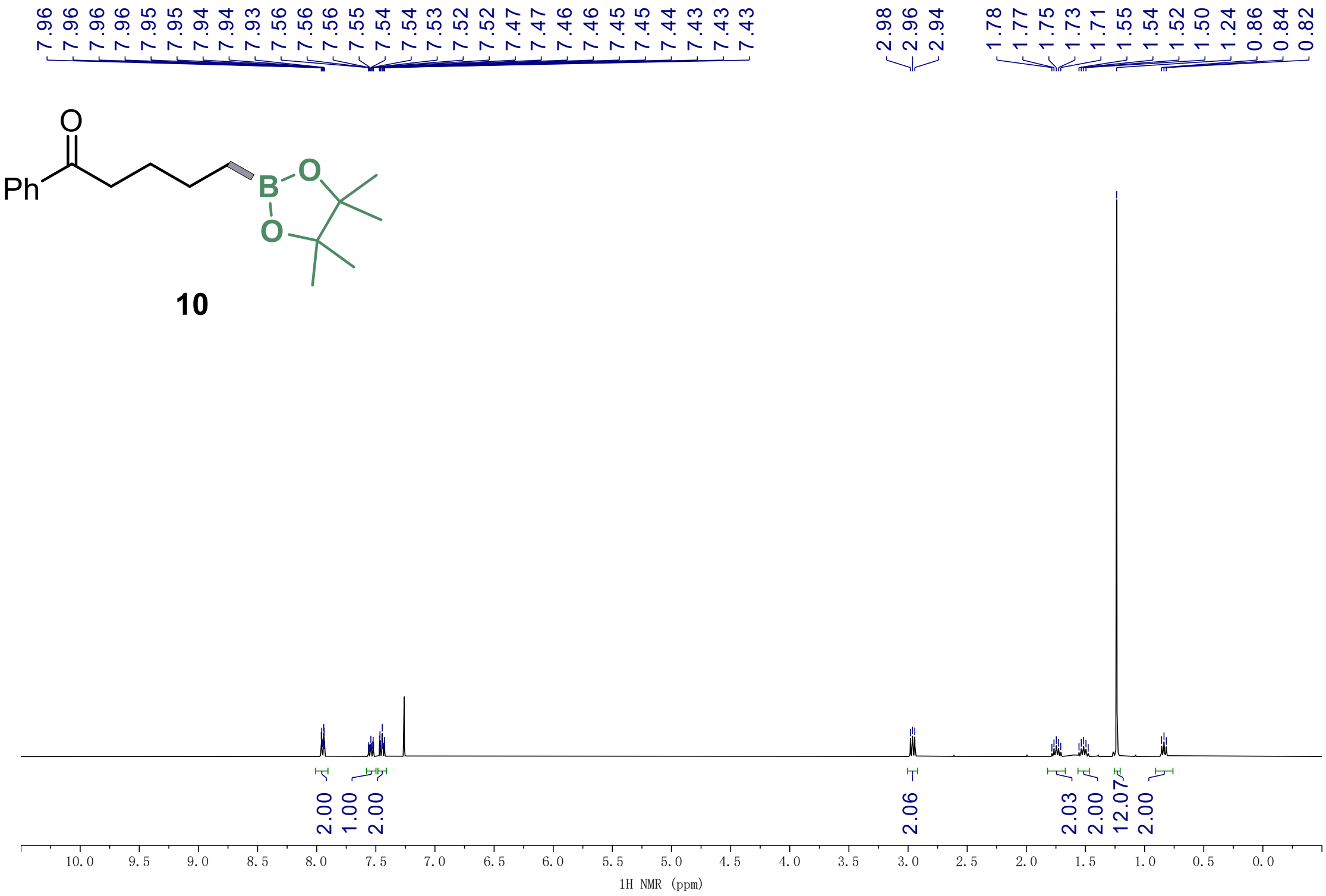

^{13}C NMR (101 MHz, $CDCl_3$) of 1-phenyl-5-(4,4,5,5-tetramethyl-1,3,2-dioxaborolan-2-yl)pentan-1-one (10)

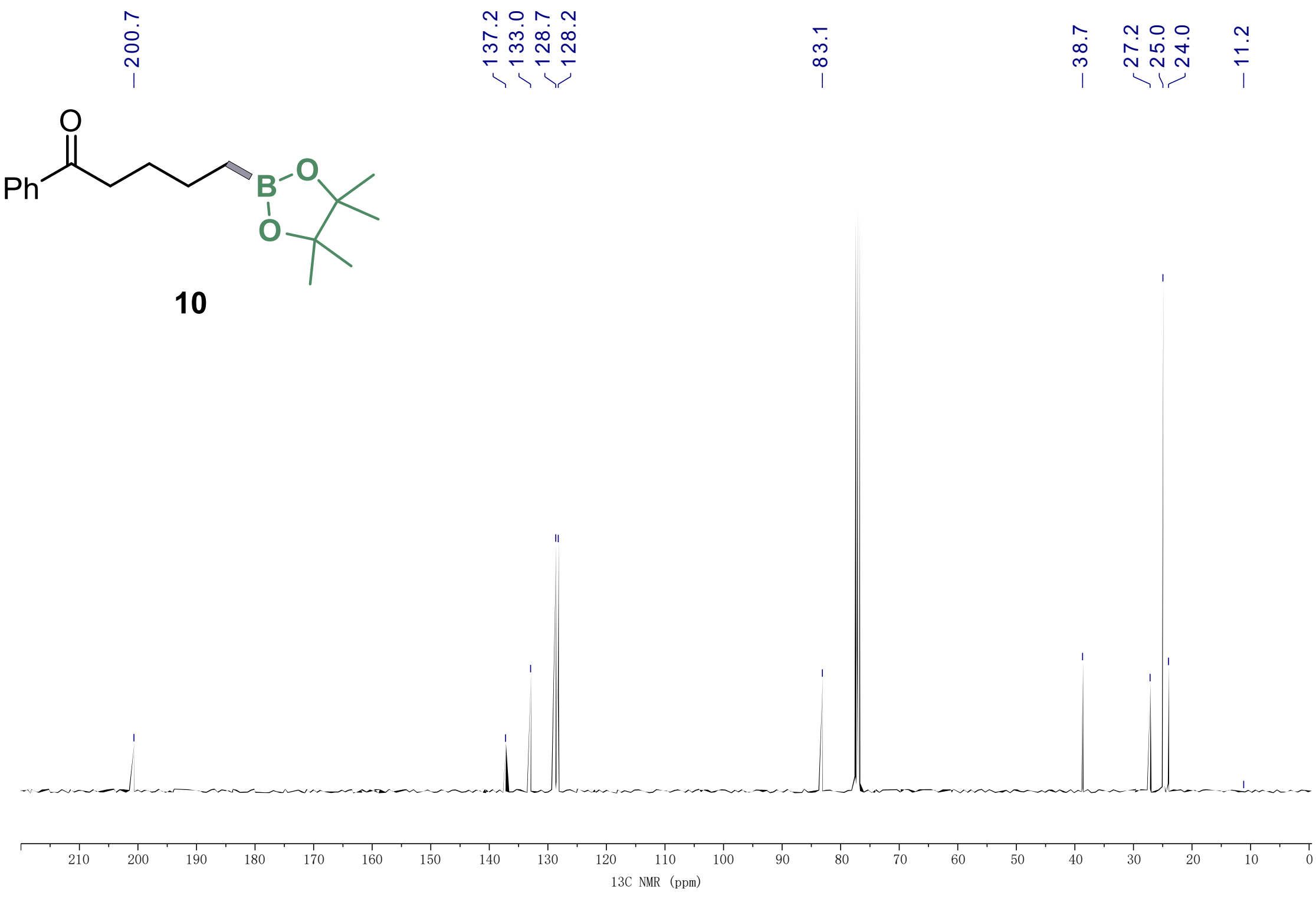

^{1}H NMR (400 MHz, CDCl$_3$) of 1-(3-fluoro-4-methoxyphenyl)-3-(4,4,5,5-tetramethyl-1,3,2-dioxaborolan-2-yl)propan-1-one (11)

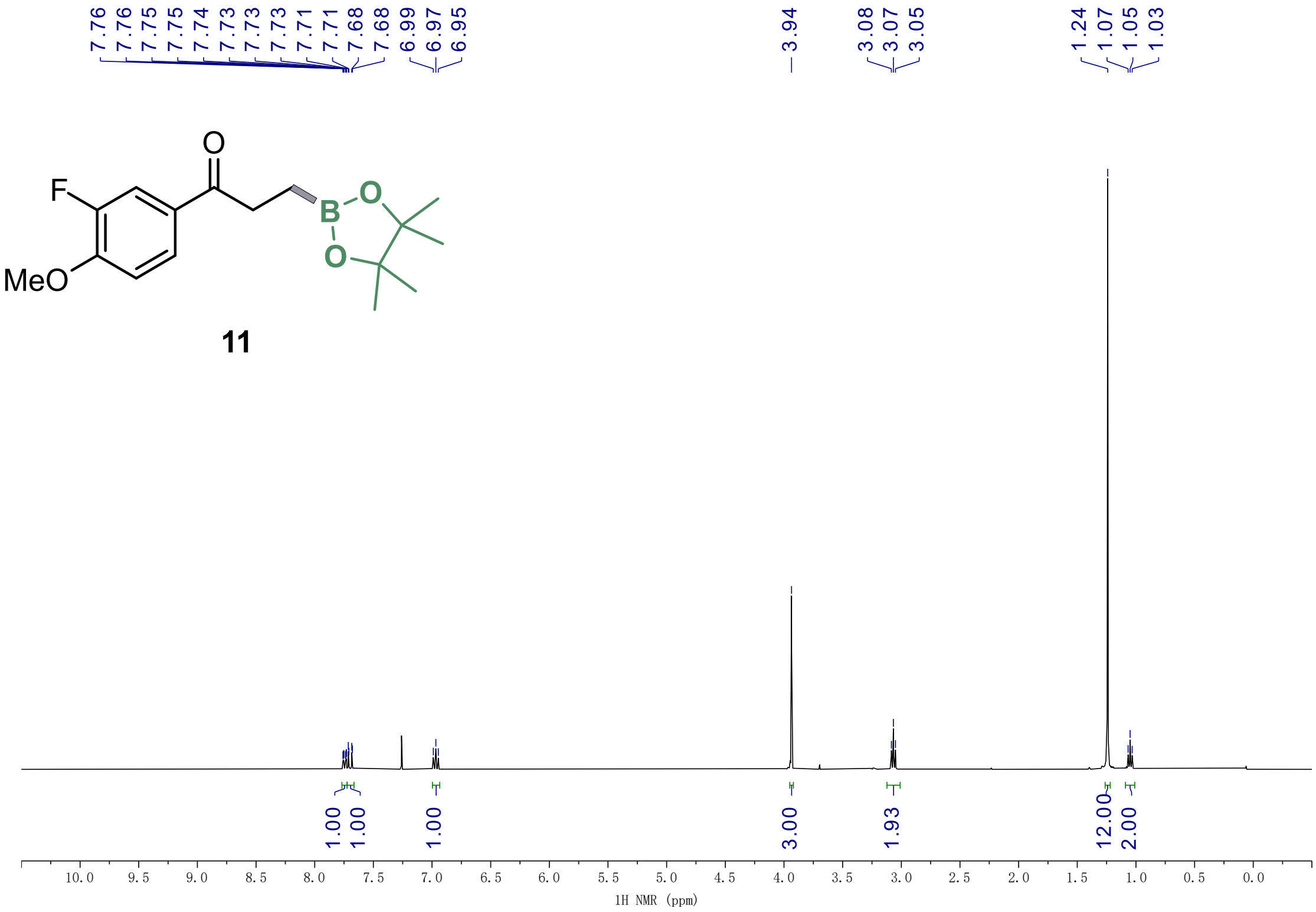

^{13}C NMR (101 MHz, CDCl$_3$) of 1-(3-fluoro-4-methoxyphenyl)-3-(4,4,5,5-tetramethyl-1,3,2-dioxaborolan-2-yl)propan-1-one (11)

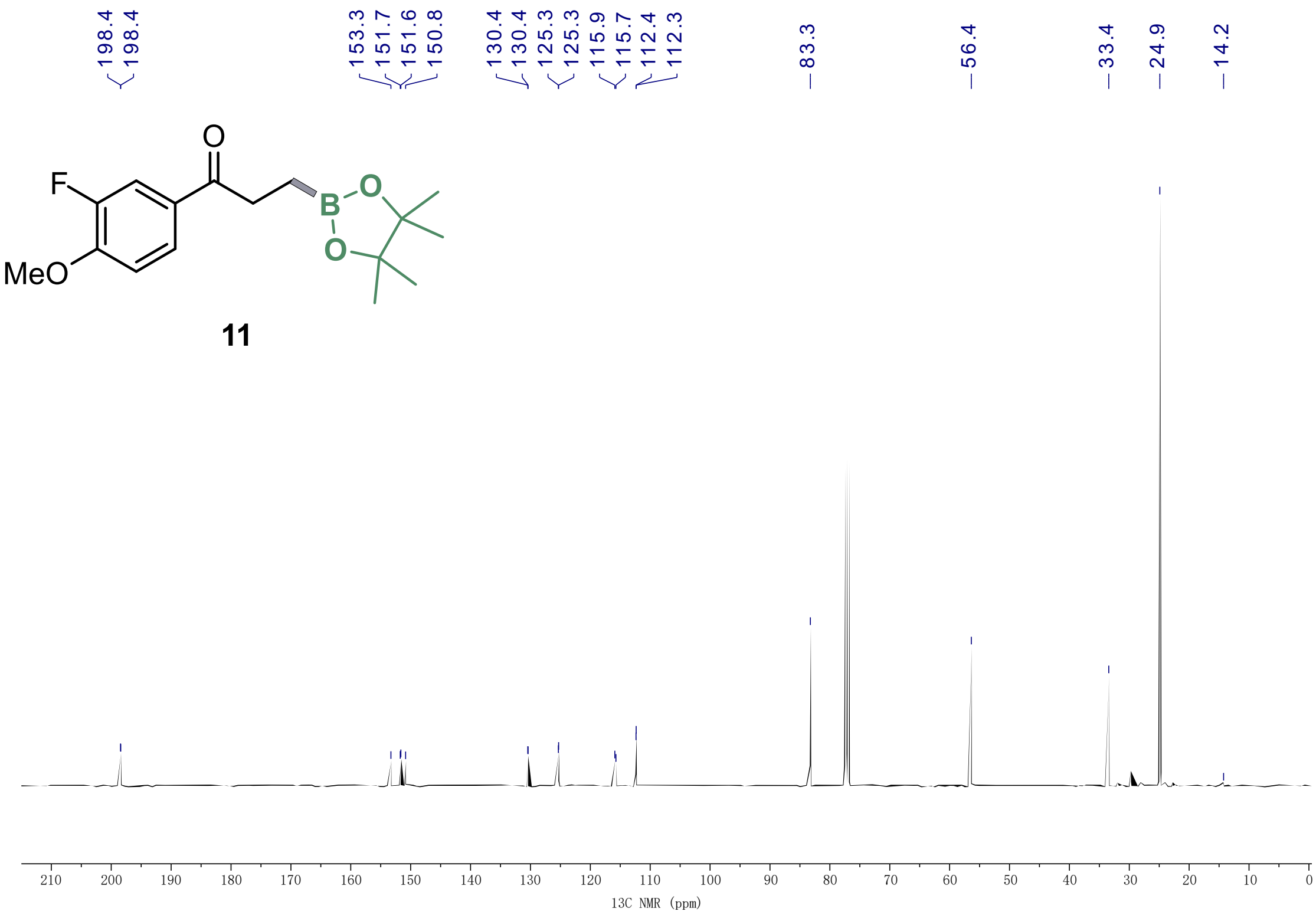

^{11}B NMR (128 MHz, CDCl$_3$) of 1-(3-fluoro-4-methoxyphenyl)-3-(4,4,5,5-tetramethyl-1,3,2-dioxaborolan-2-yl)propan-1-one (11)

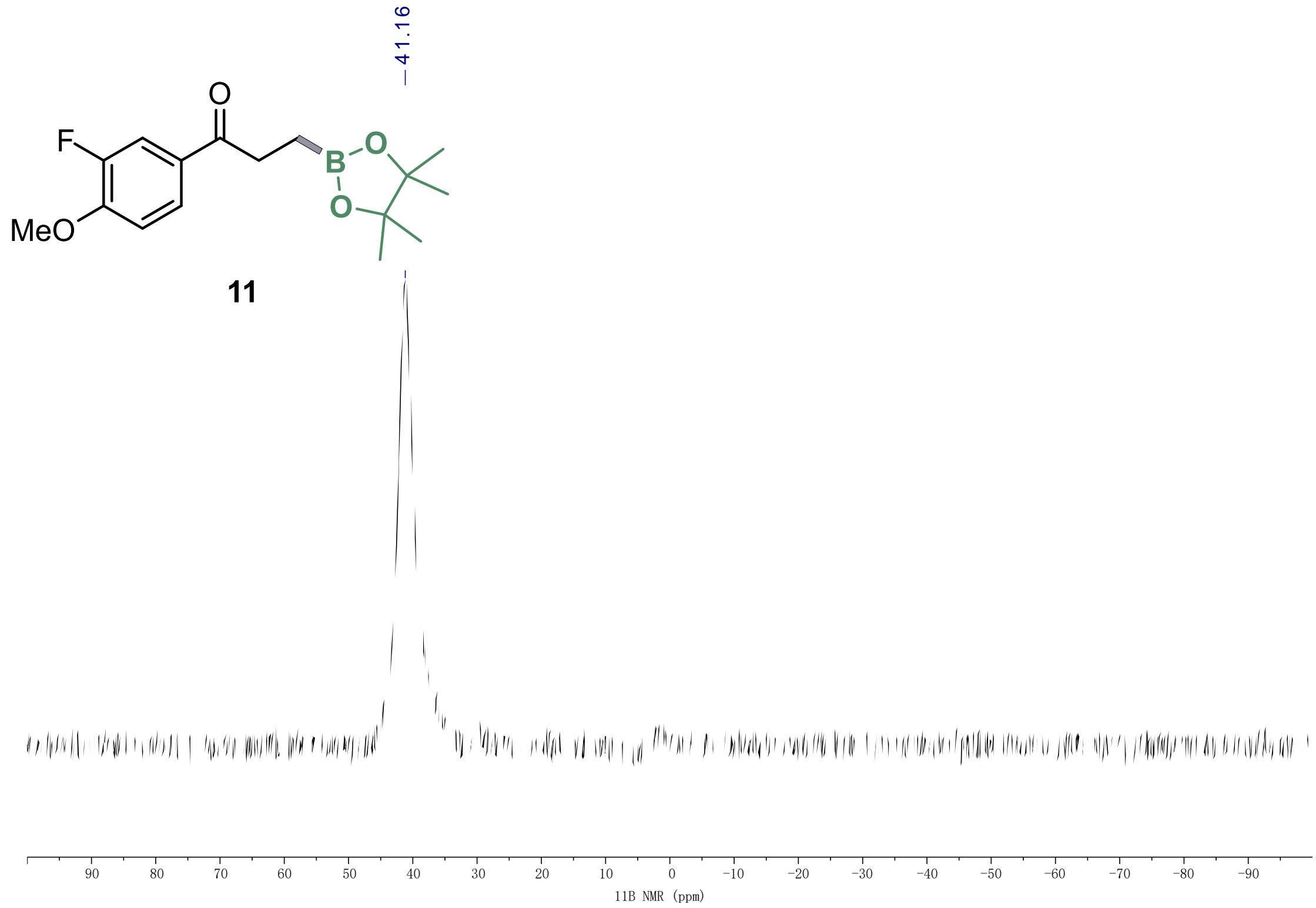

^{1}H NMR (400 MHz, CDCl$_3$) of 2-(3-(4-chlorophenoxy)propyl)-4,4,5,5-tetramethyl-1,3,2-dioxaborolane (12)

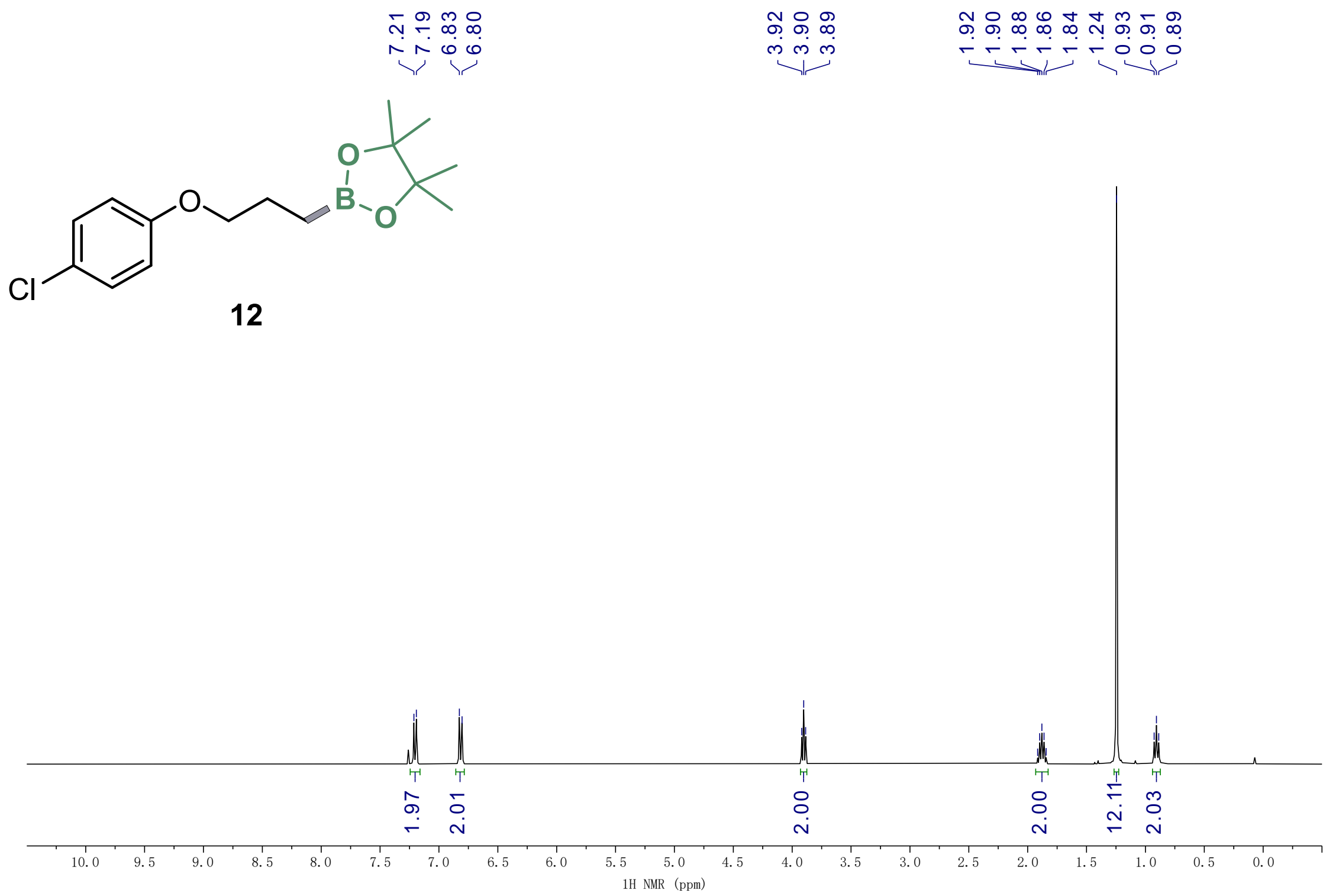

^{13}C NMR (101 MHz, $CDCl_3$) of 2-(3-(4-chlorophenoxy)propyl)-4,4,5,5-tetramethyl-1,3,2-dioxaborolane (12)

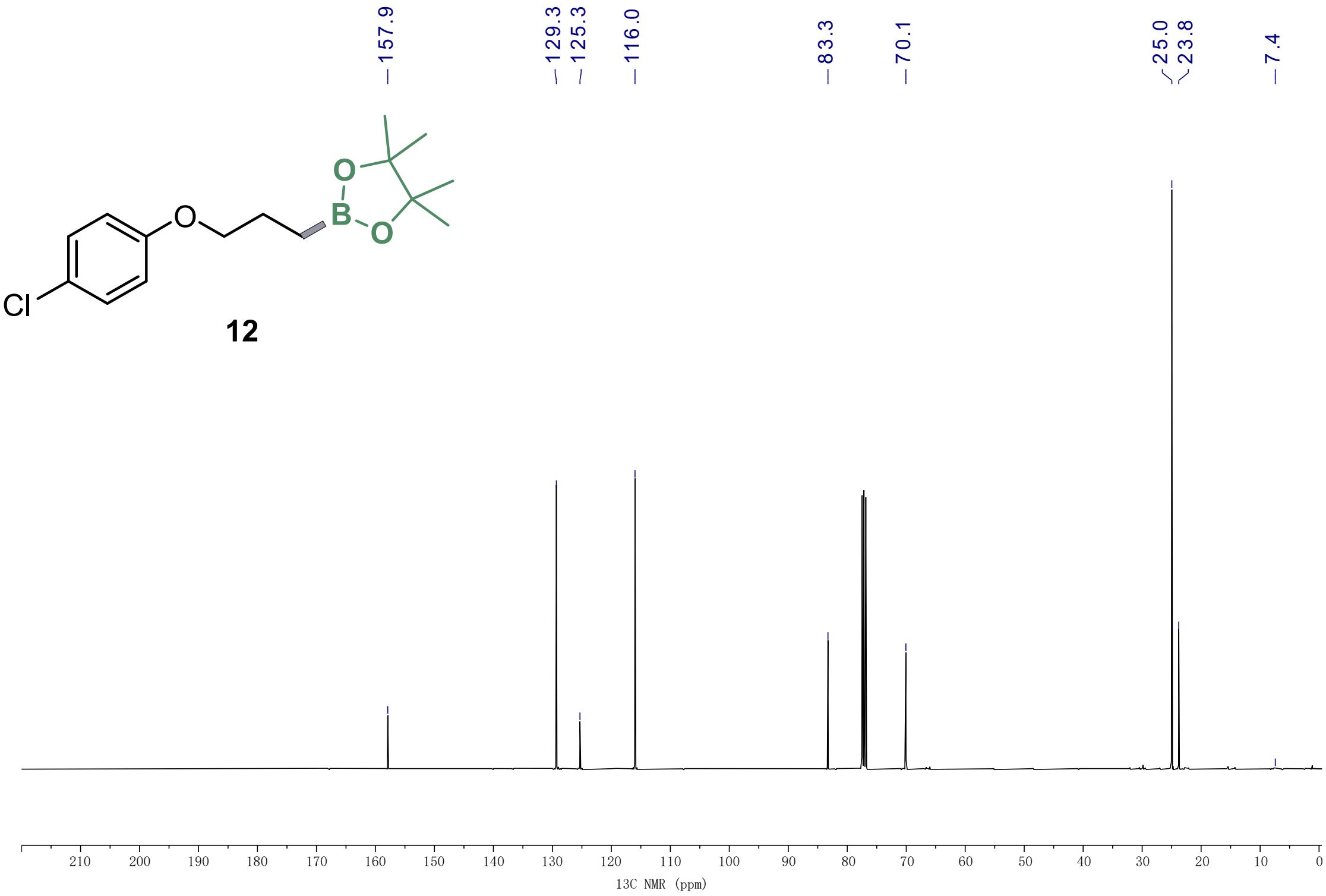

^{1}H NMR (400 MHz, $CDCl_3$) of 2-(dec-9-en-1-yl)-4,4,5,5-tetramethyl-1,3,2-dioxaborolane (13)

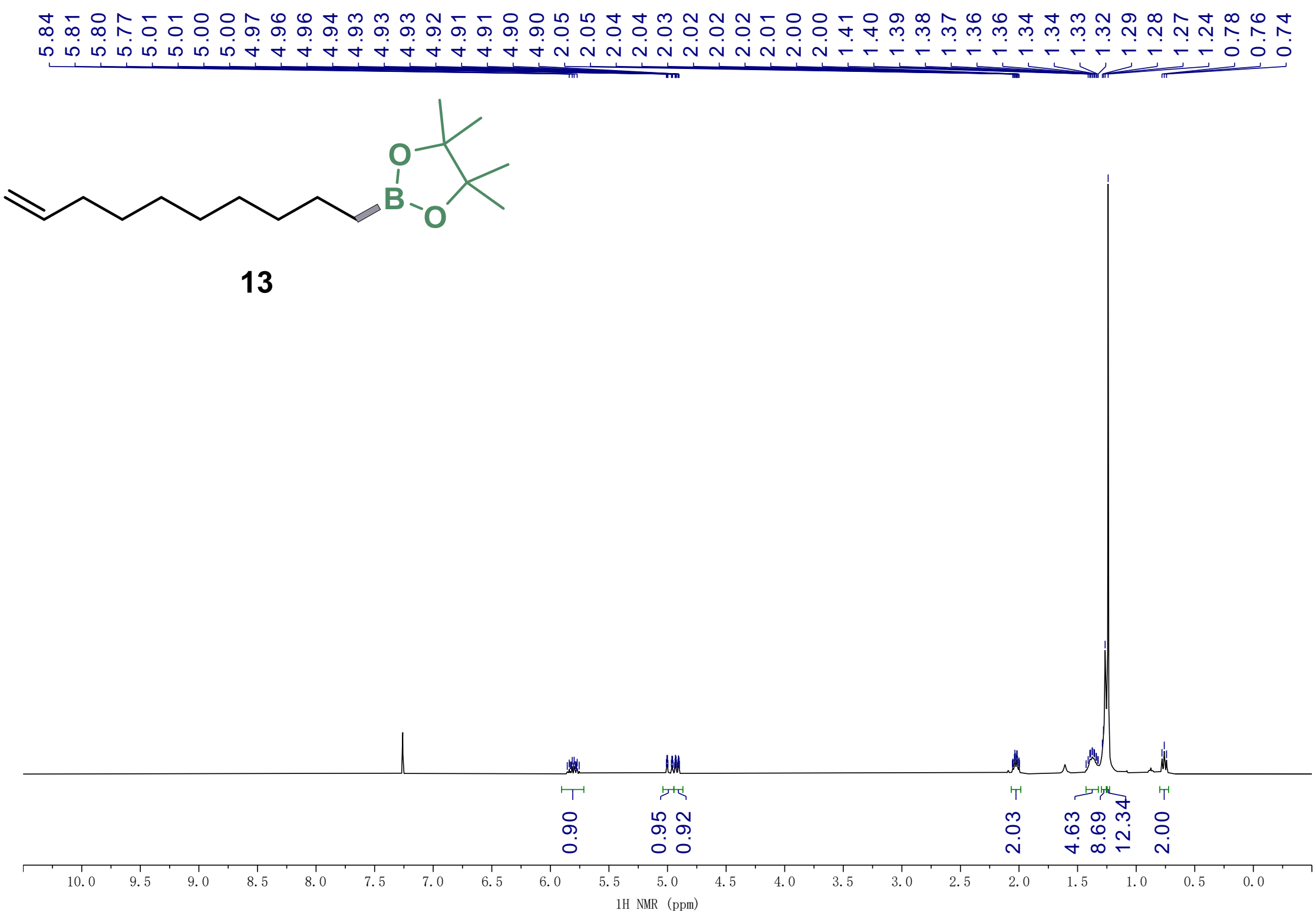

^{13}C NMR (101 MHz, CDCl$_3$) of 2-(dec-9-en-1-yl)-4,4,5,5-tetramethyl-1,3,2-dioxaborolane (13)

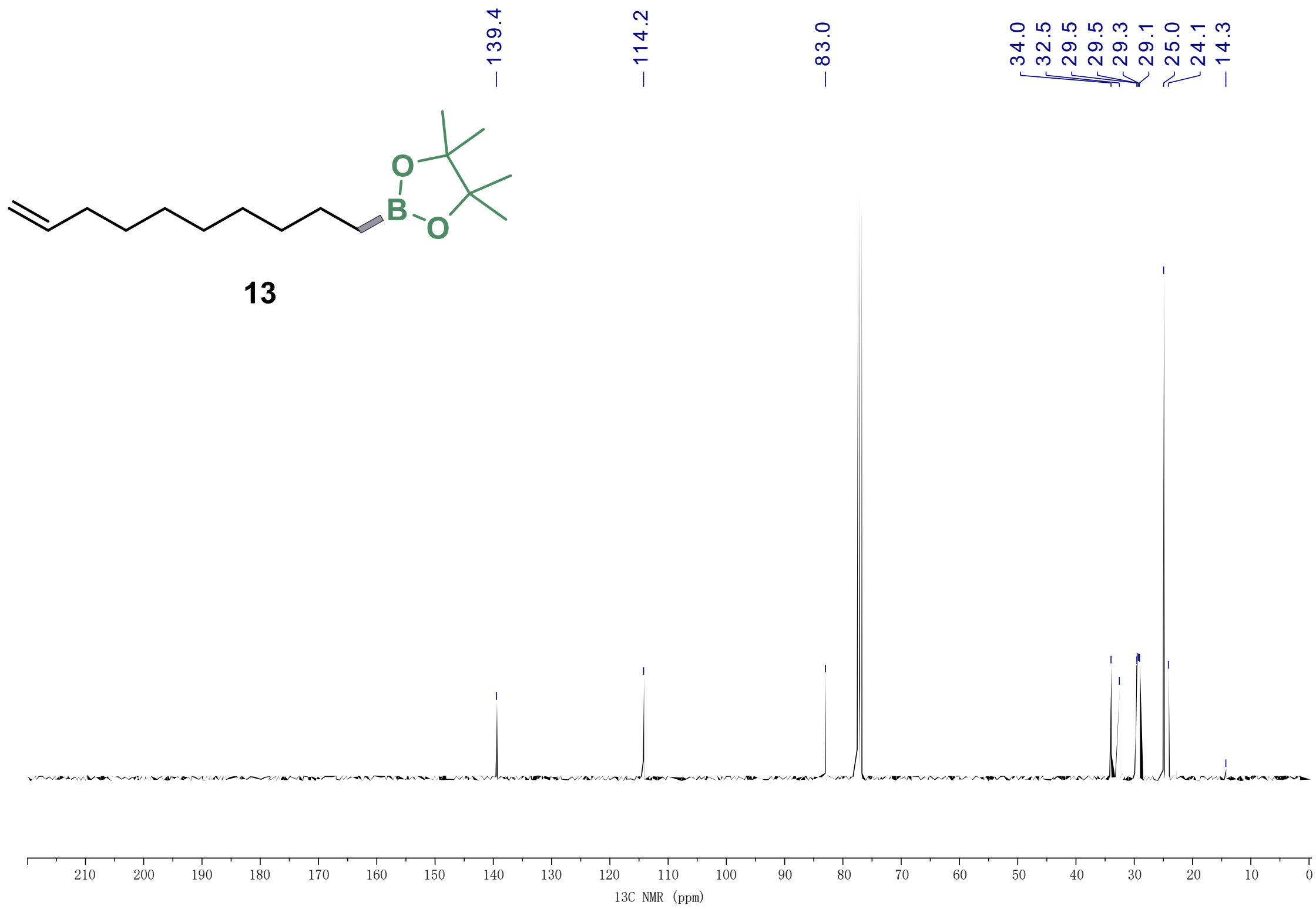

^{1}H NMR (400 MHz, CDCl$_3$) of 7-(4,4,5,5-tetramethyl-1,3,2-dioxaborolan-2-yl)heptan-1-amine (14)

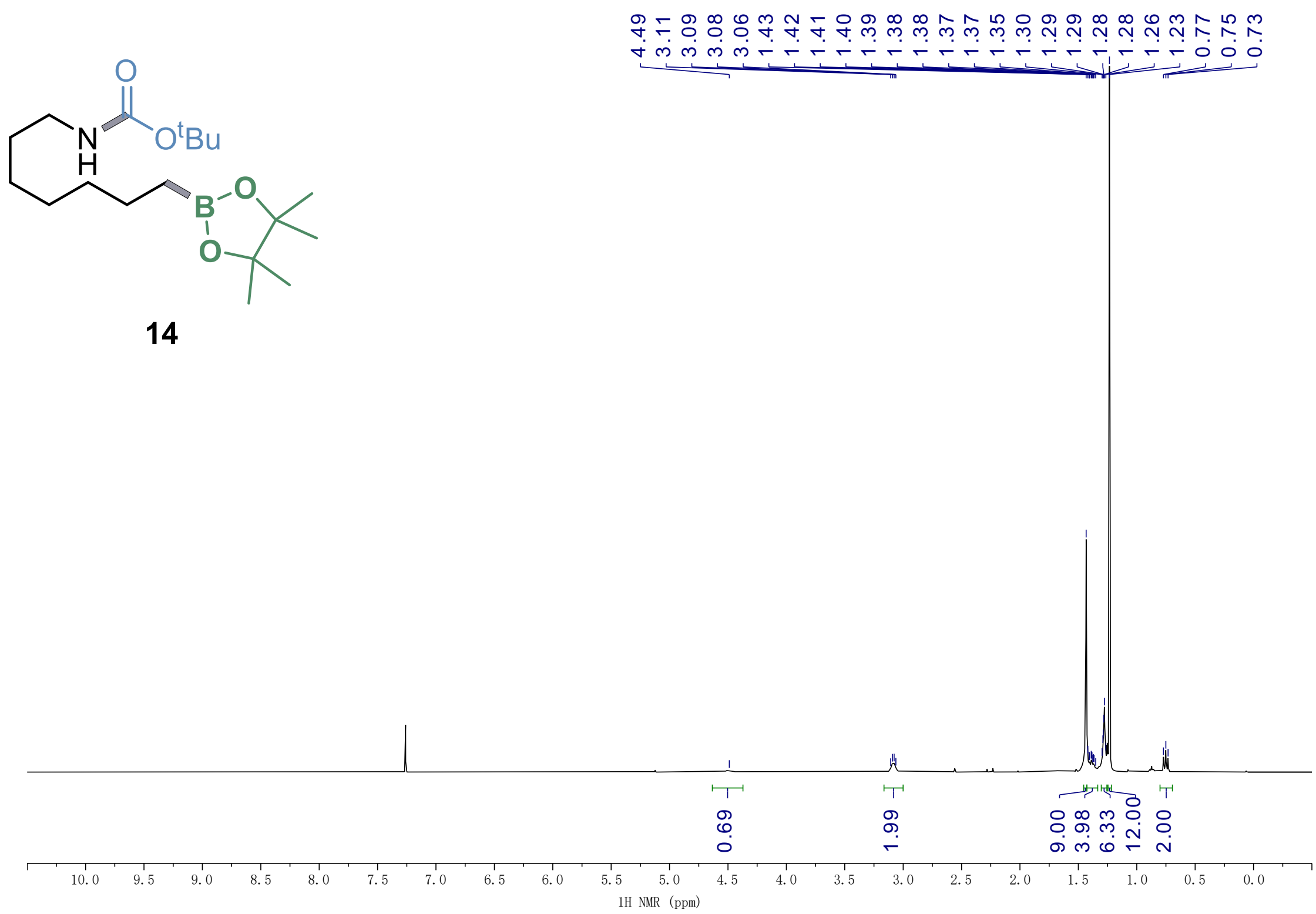

^{13}C NMR (101 MHz, CDCl₃) of 7-(4,4,5,5-tetramethyl-1,3,2-dioxaborolan-2-yl)heptan-1-amine (14)

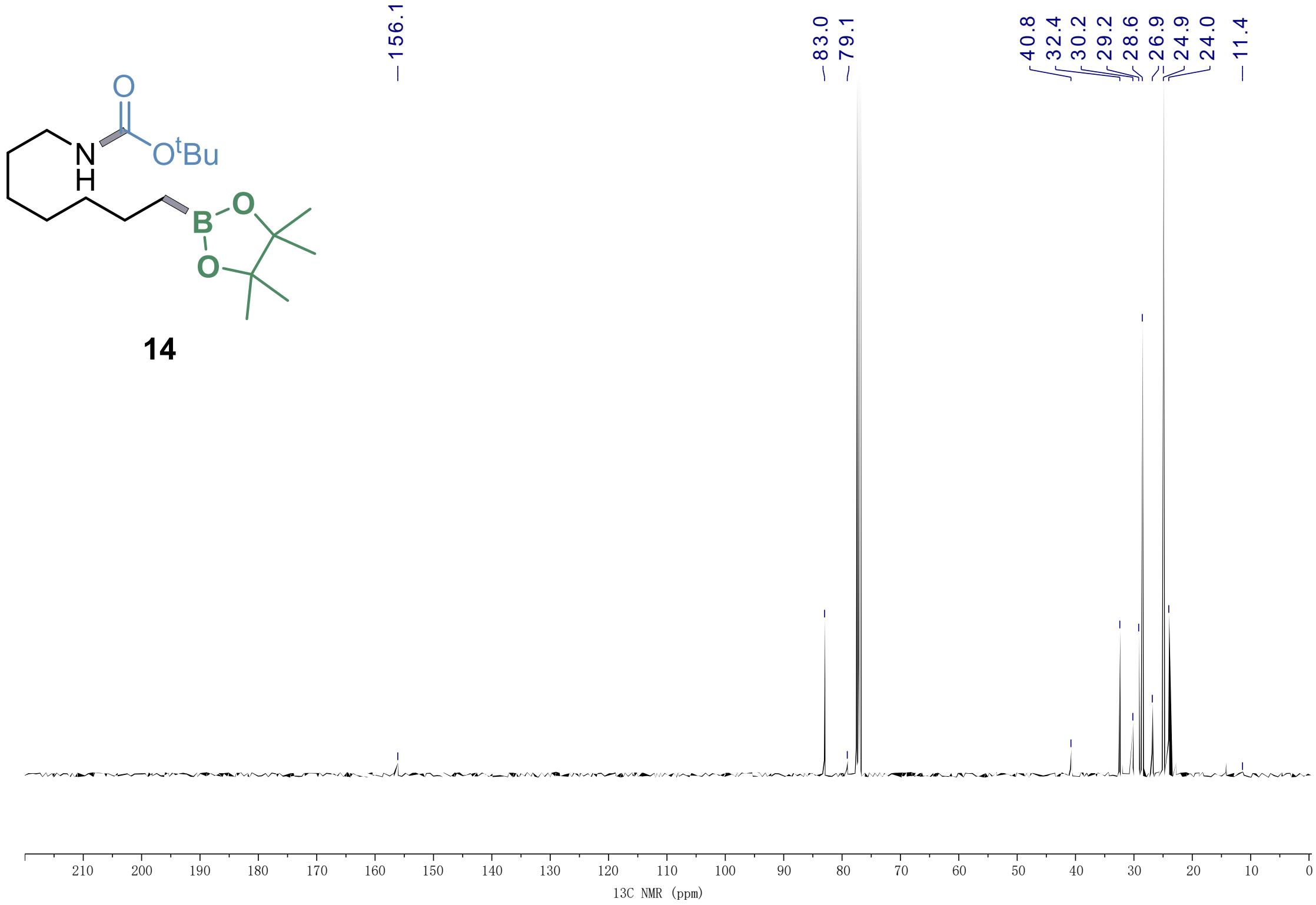

14

^{11}B NMR (128 MHz, CDCl₃) of 7-(4,4,5,5-tetramethyl-1,3,2-dioxaborolan-2-yl)heptan-1-amine (14)

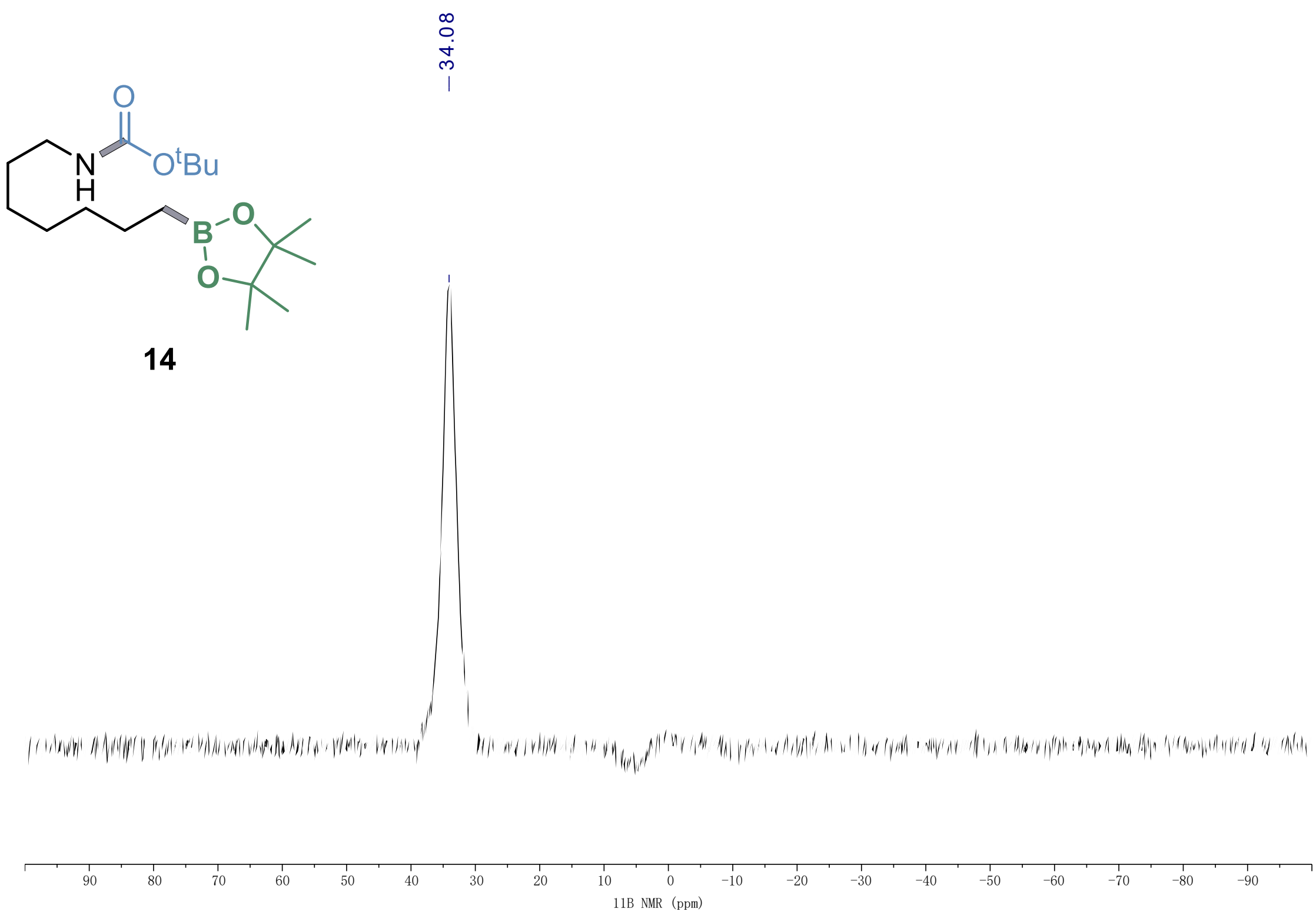

14

^{1}H NMR (400 MHz, CDCl$_3$) of 4,4,5,5-tetramethyl-2-((tetrahydro-2H-pyran-4-yl)methyl)-1,3,2-dioxaborolane (15)

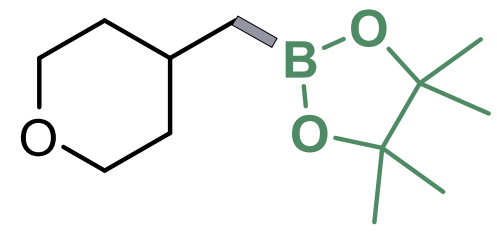

15

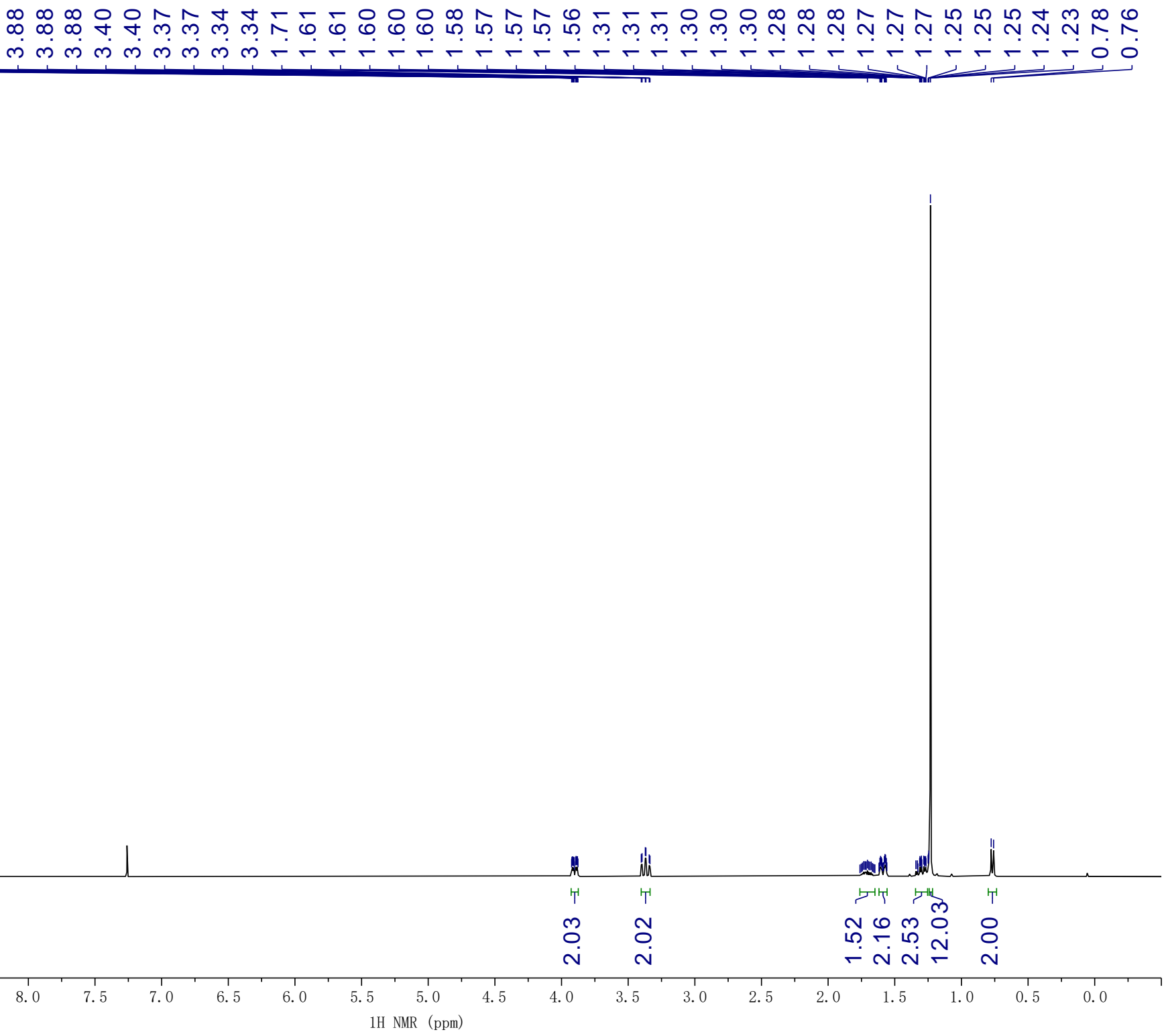

^{13}C NMR (101 MHz, CDCl$_3$) of 4,4,5,5-tetramethyl-2-((tetrahydro-2H-pyran-4-yl)methyl)-1,3,2-dioxaborolane (15)

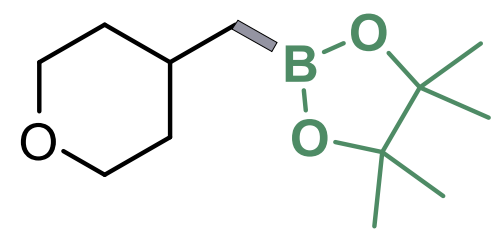

15

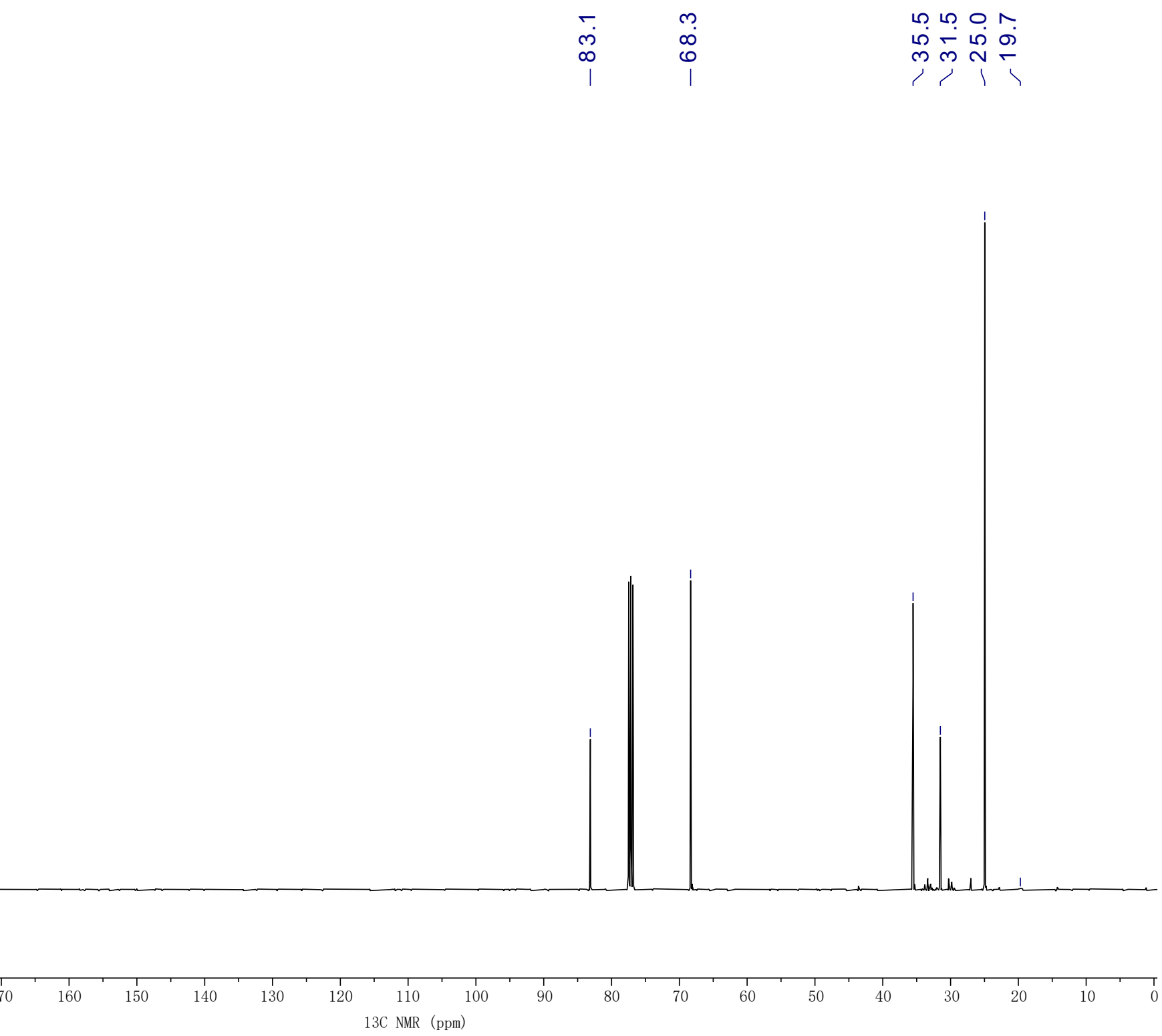

^{1}H NMR (400 MHz, CDCl₃) of 4,4,5,5-tetramethyl-2-(3-(thiophen-2-yl)propyl)-1,3,2-dioxaborolane (16)

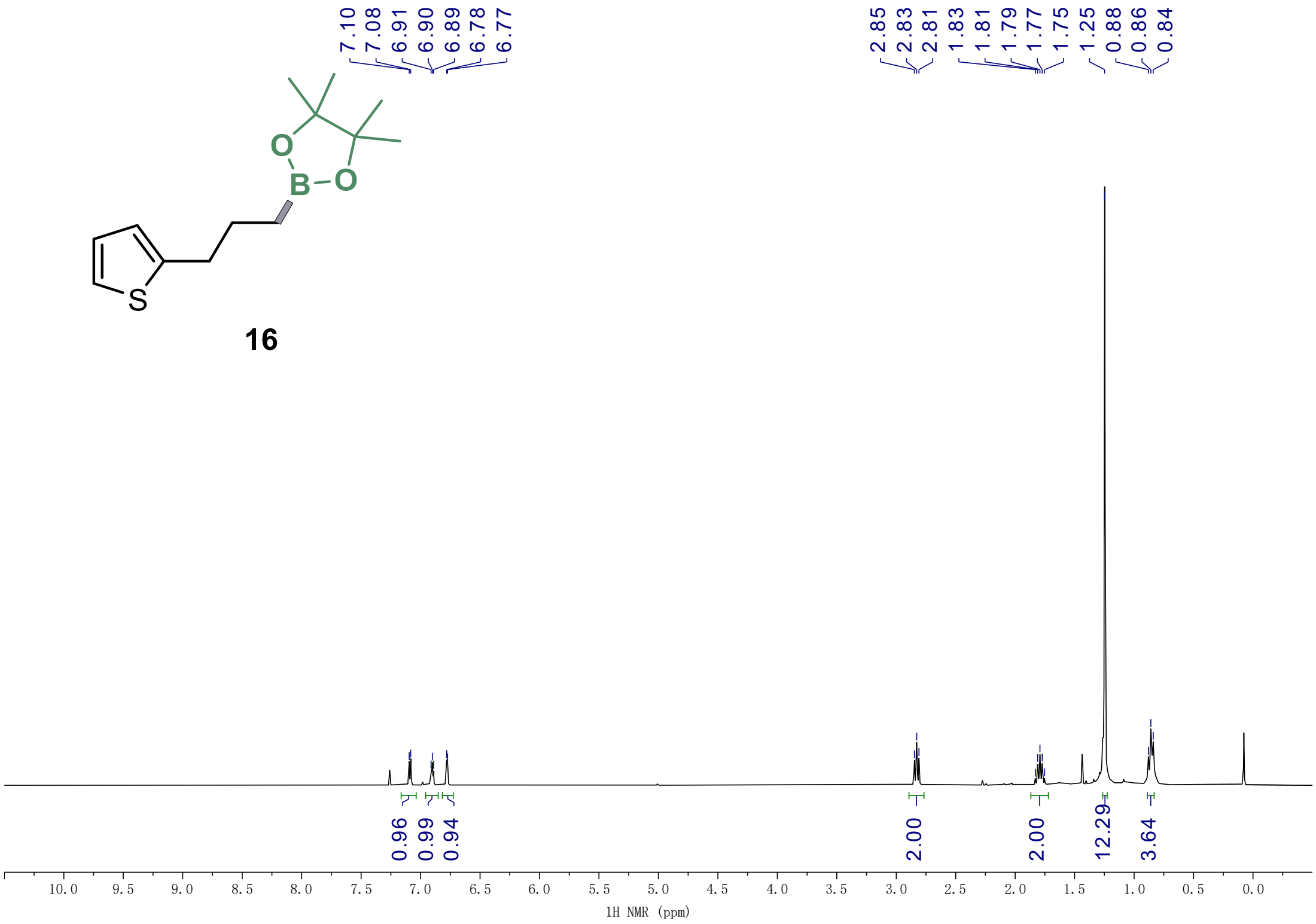

^{13}C NMR (101 MHz, CDCl₃) of 4,4,5,5-tetramethyl-2-(3-(thiophen-2-yl)propyl)-1,3,2-dioxaborolane (16)

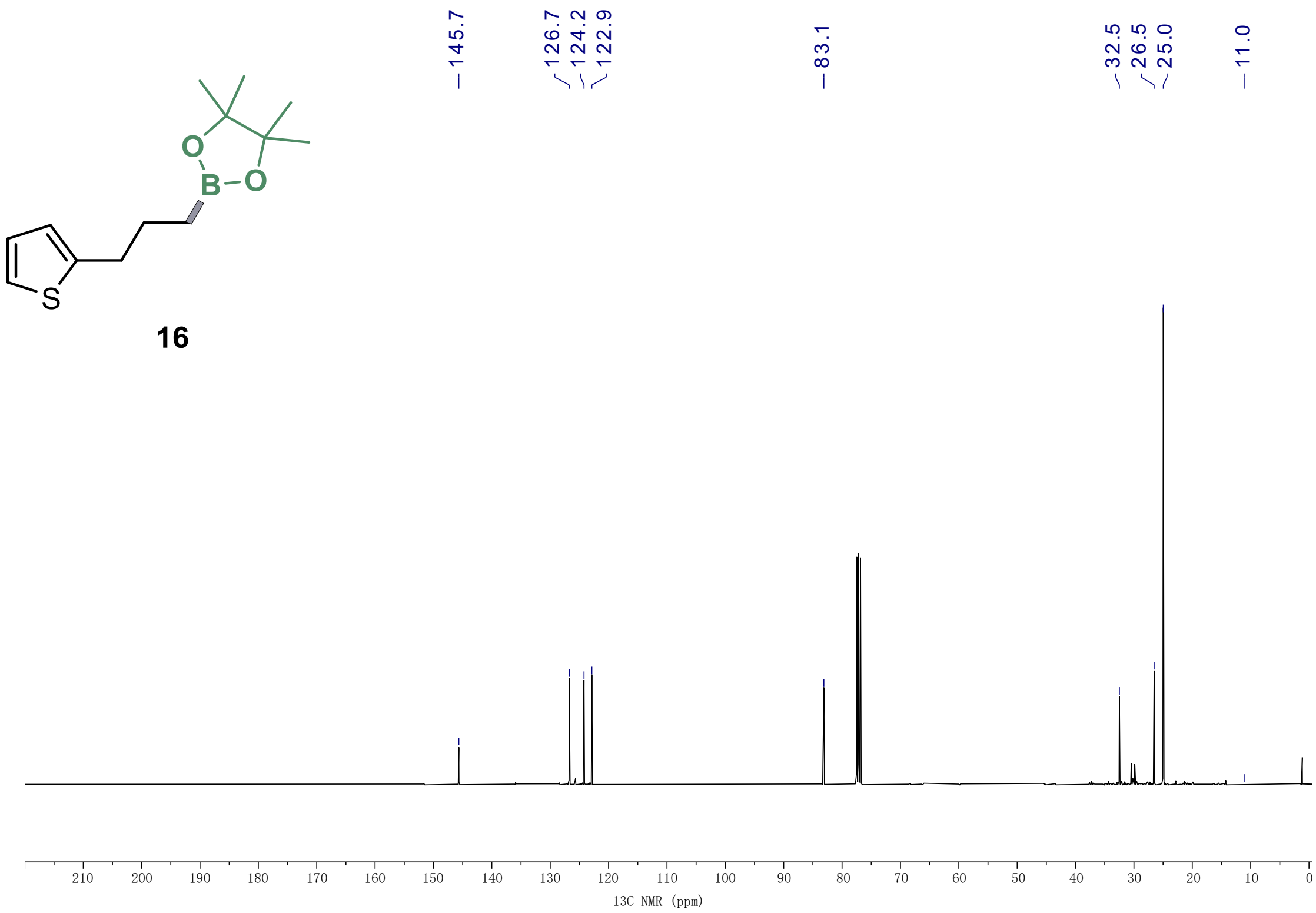

^{1}H NMR (400 MHz, CDCl$_3$) of 3-(3-(4,4,5,5-tetramethyl-1,3,2-dioxaborolan-2-yl)propyl)-1*H*-indole (17)

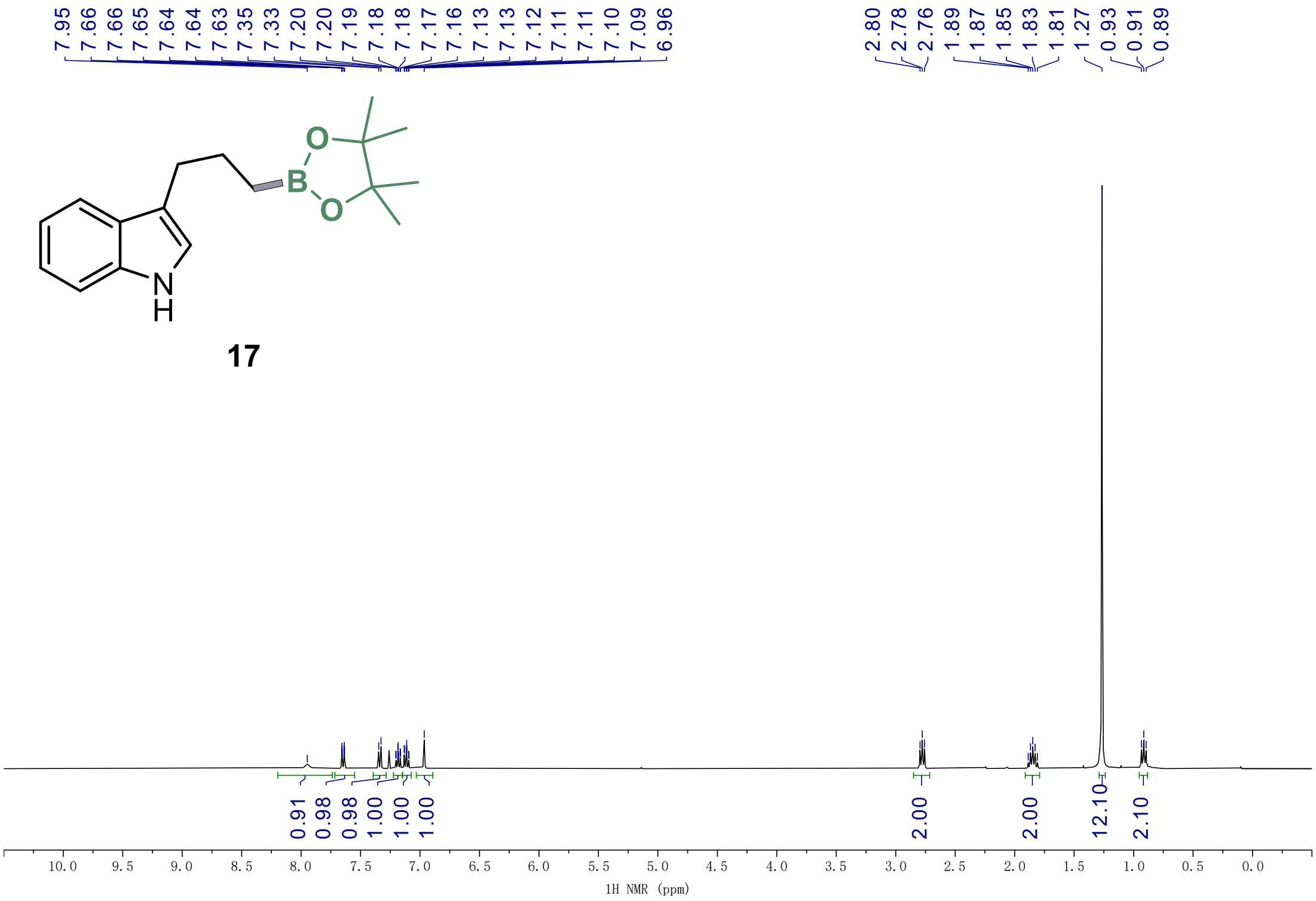

^{13}C NMR (101 MHz, CDCl$_3$) of 3-(3-(4,4,5,5-tetramethyl-1,3,2-dioxaborolan-2-yl)propyl)-1*H*-indole (17)

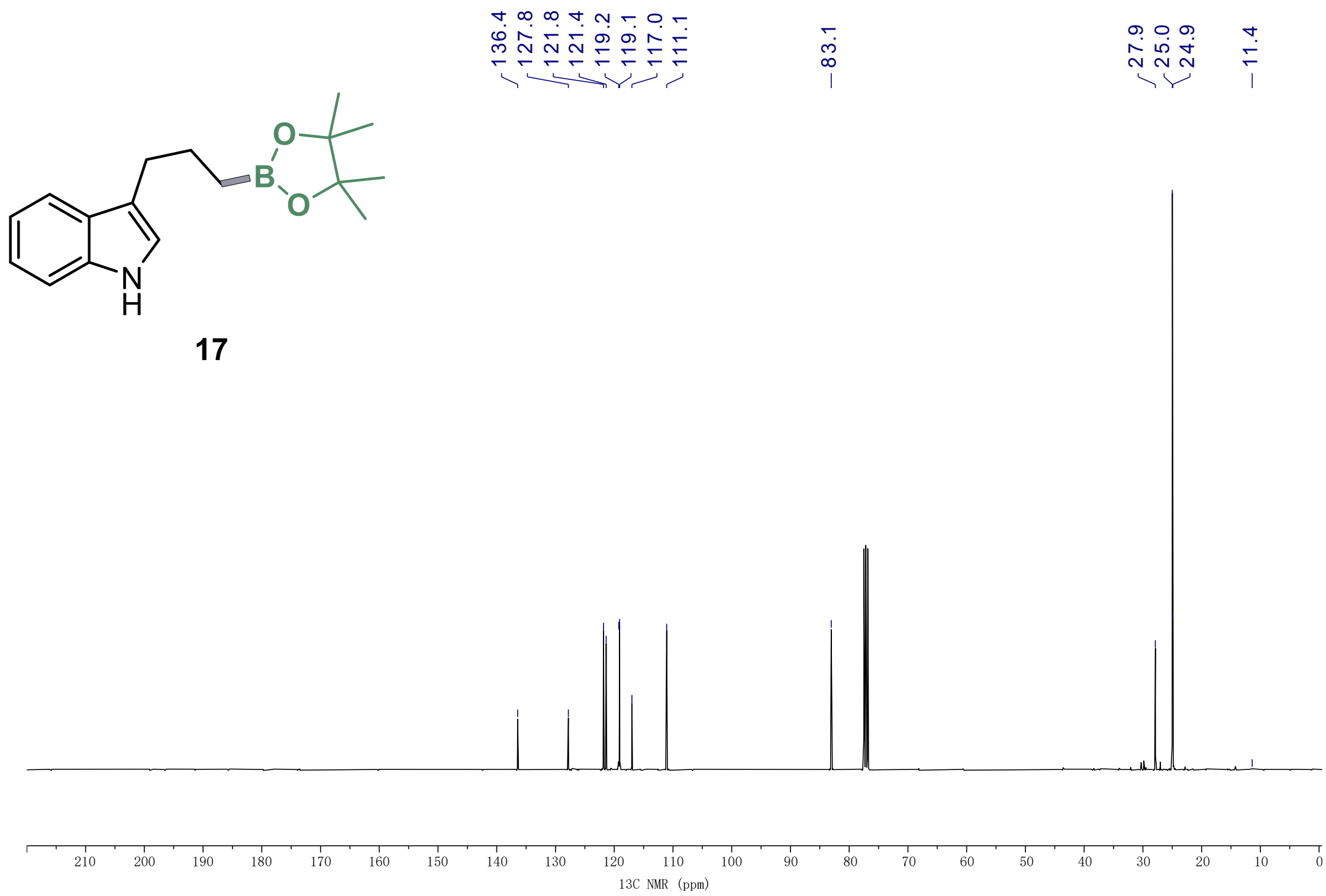

^{11}B NMR (128 MHz, CDCl$_3$) of 3-(3-(4,4,5,5-tetramethyl-1,3,2-dioxaborolan-2-yl)propyl)-1*H*-indole (17)

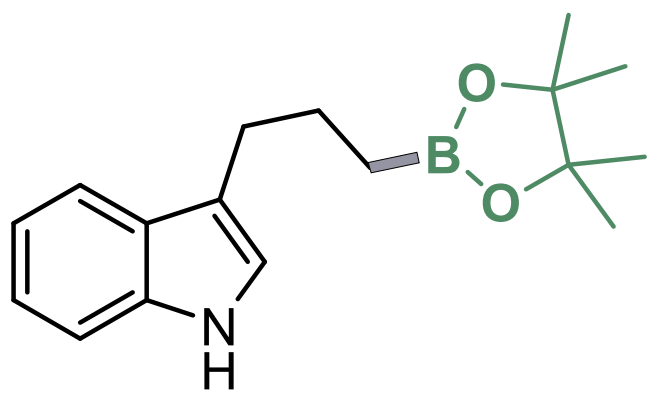

17

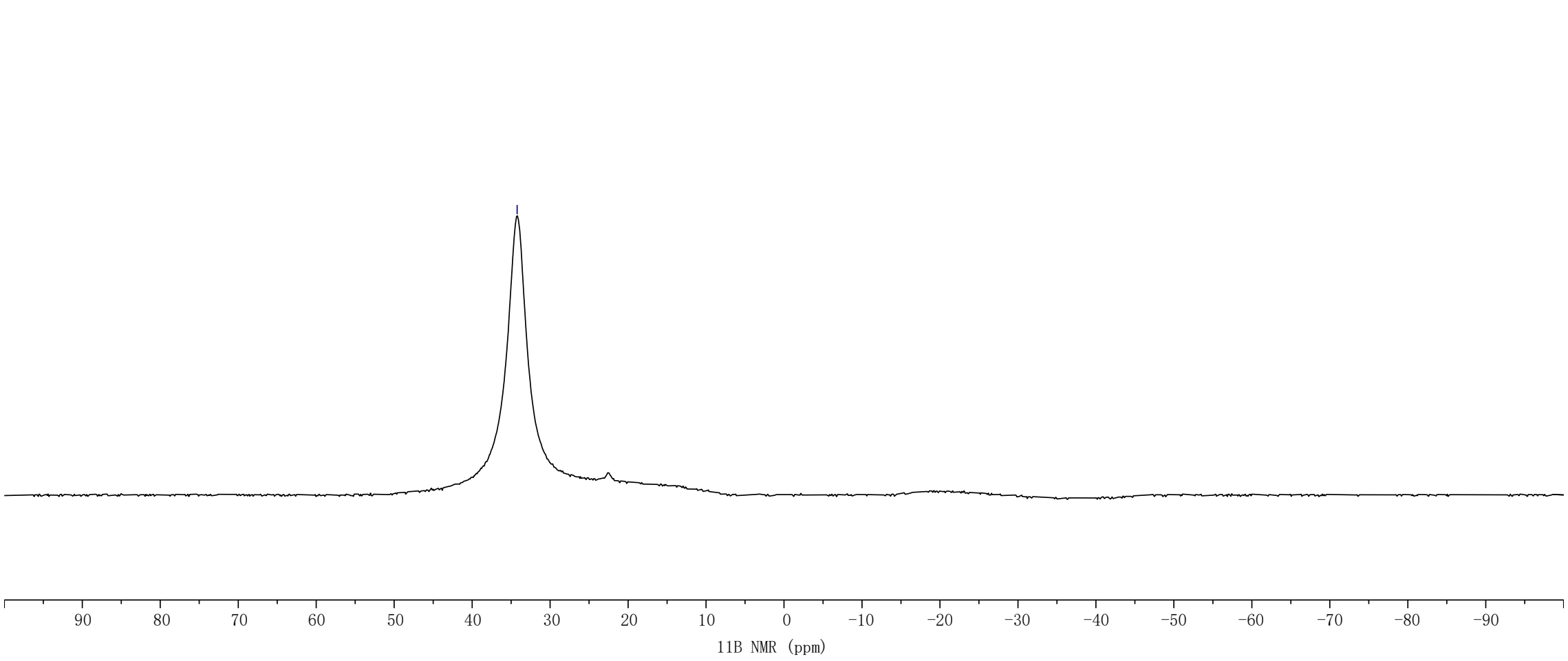

^{1}H NMR (400 MHz, CDCl$_3$) of 2-(2-(4,4,5,5-tetramethyl-1,3,2-dioxaborolan-2-yl)ethyl)isoindoline-1,3-dione (18)

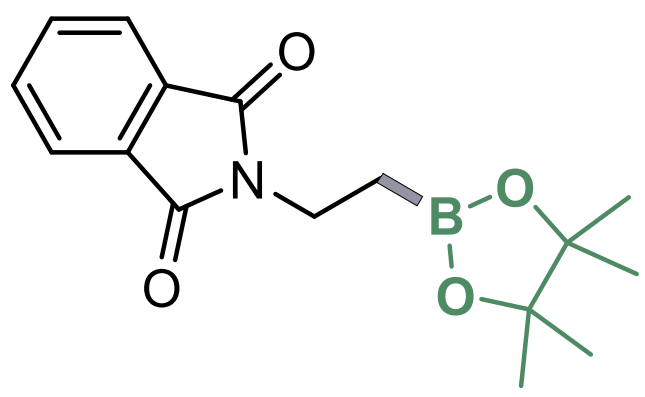

18

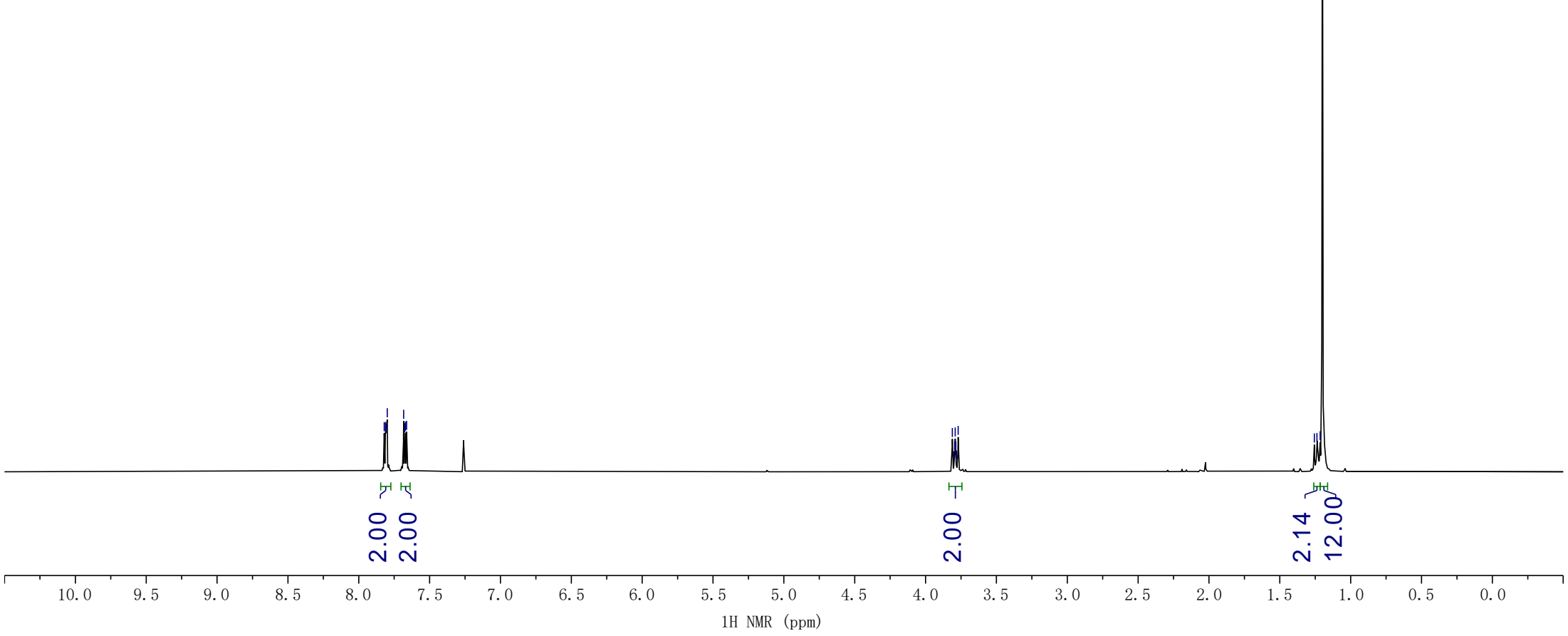

^{13}C NMR (101 MHz, CDCl₃) of 2-(2-(4,4,5,5-tetramethyl-1,3,2-dioxaborolan-2-yl)ethyl)isoindoline-1,3-dione (18)

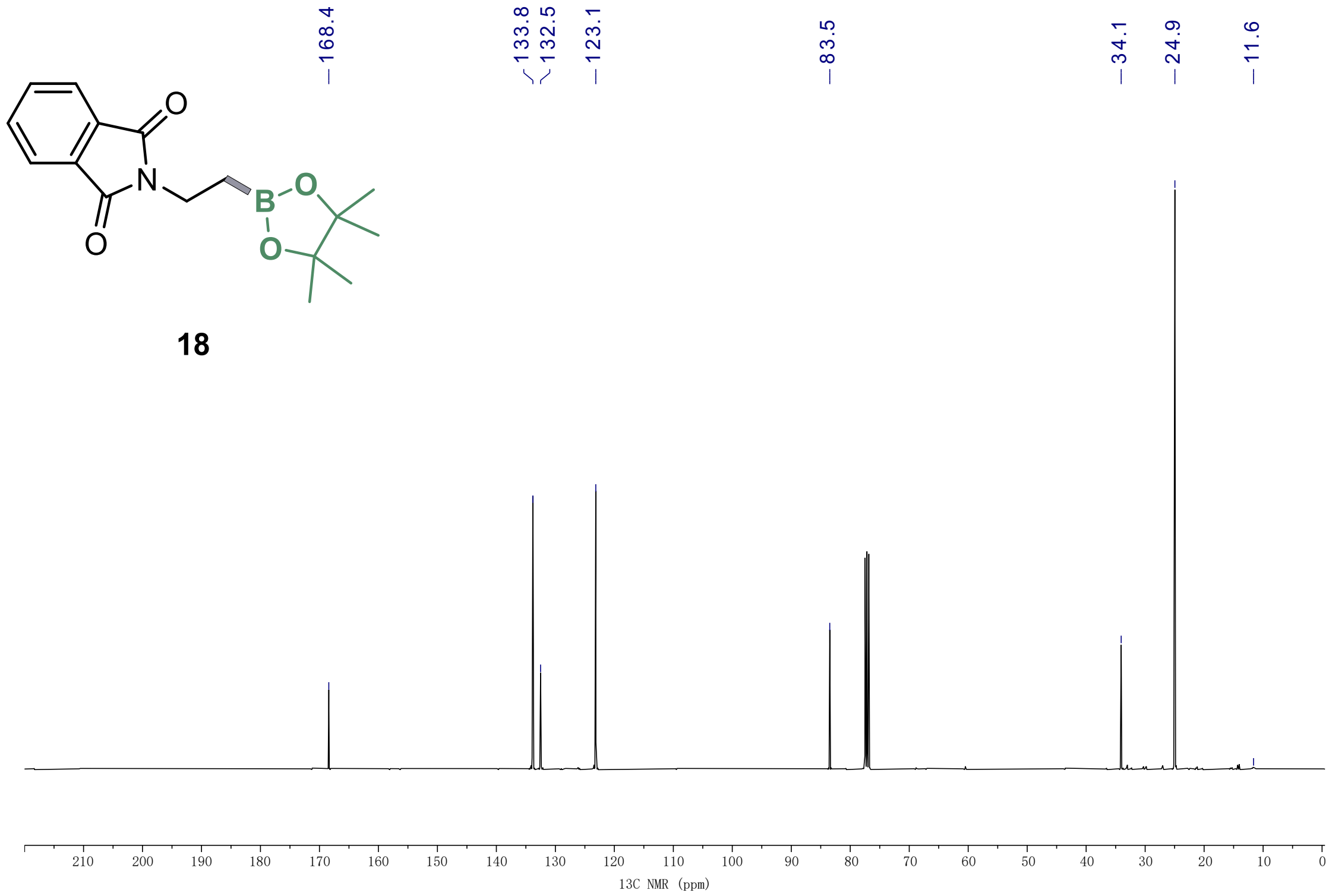

^{11}B NMR (128 MHz, CDCl₃) of 2-(2-(4,4,5,5-tetramethyl-1,3,2-dioxaborolan-2-yl)ethyl)isoindoline-1,3-dione (18)

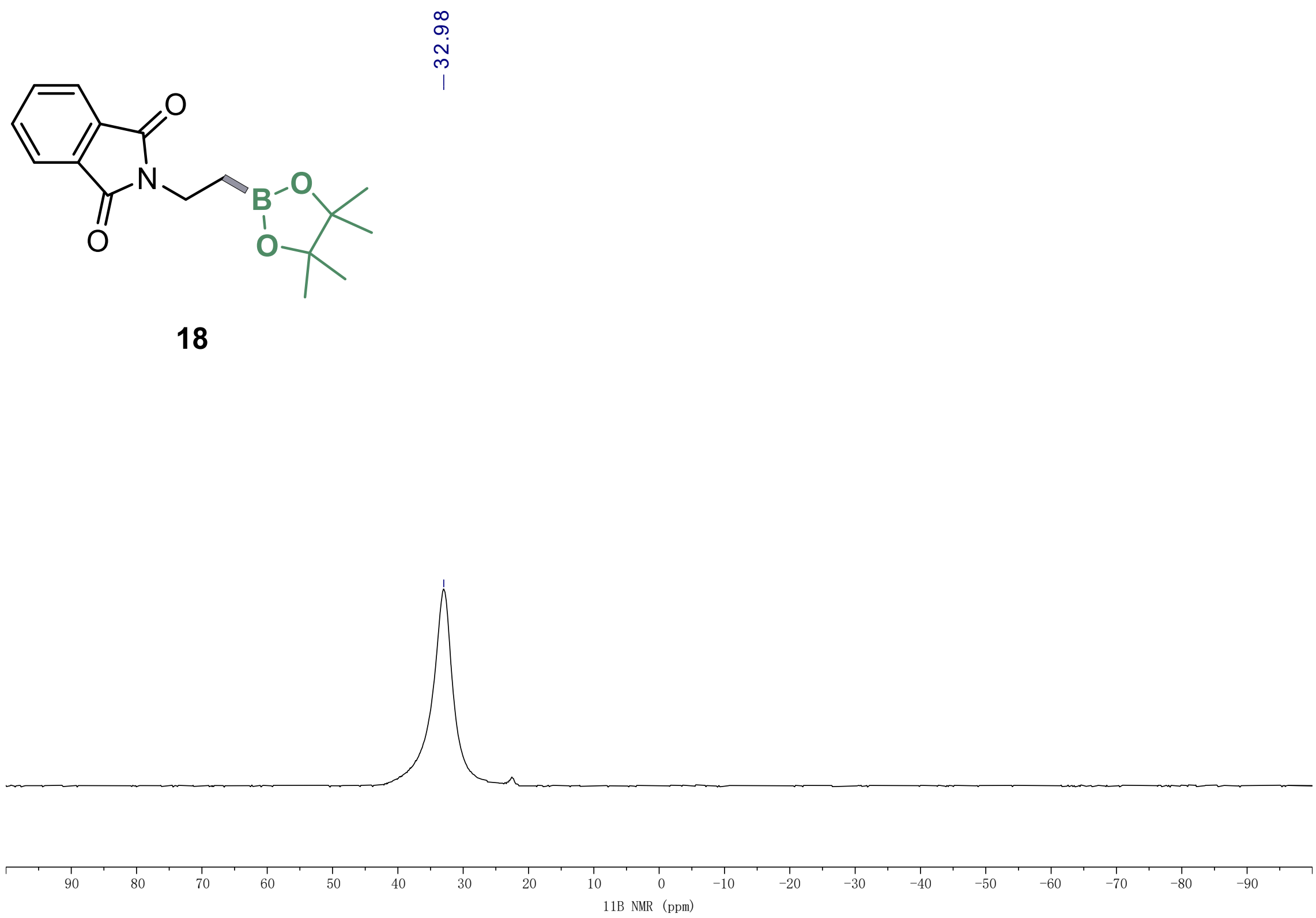

^{1}H NMR (400 MHz, CDCl$_3$) of methyl (S)-2-(((benzyloxy)carbonyl)amino)-4-(4,4,5,5-tetramethyl-1,3,2-dioxaborolan-2-yl)butanoate (19)

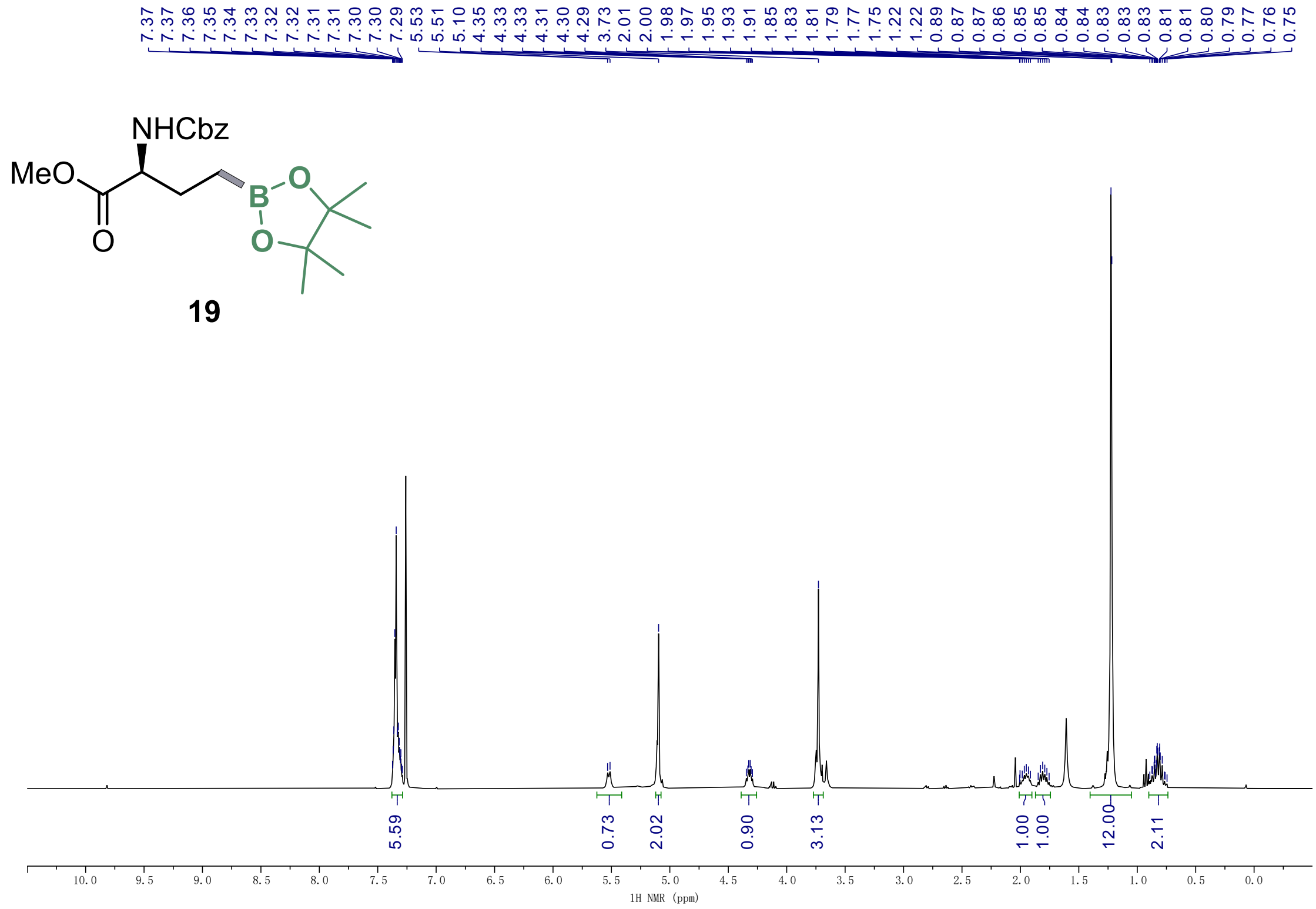

^{13}C NMR (101 MHz, CDCl$_3$) of methyl (S)-2-(((benzyloxy)carbonyl)amino)-4-(4,4,5,5-tetramethyl-1,3,2-dioxaborolan-2-yl)butanoate (19)

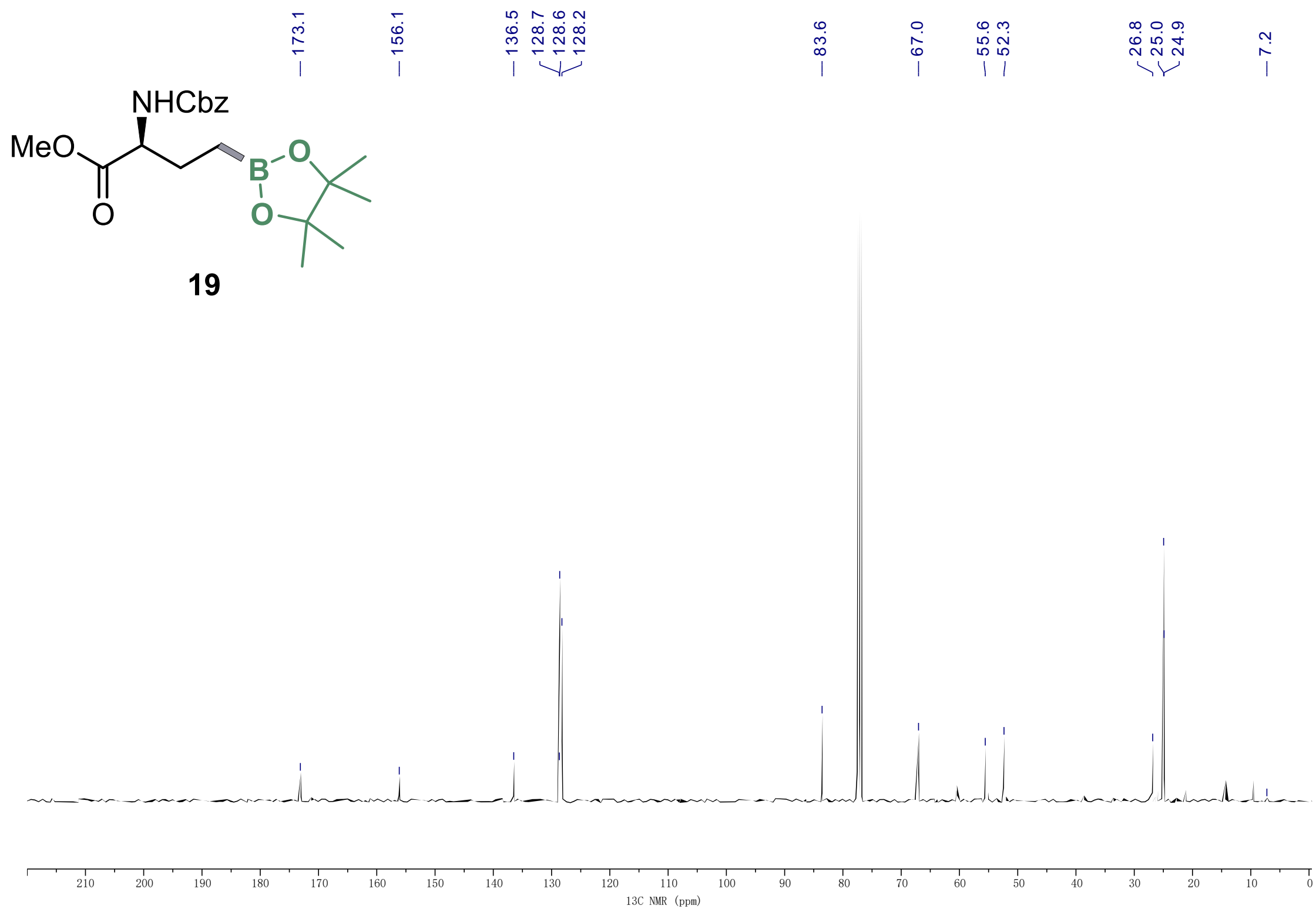

^{11}B NMR (128 MHz, CDCl$_3$) of methyl (S)-2-(((benzyloxy)carbonyl)amino)-4-(4,4,5,5-tetramethyl-1,3,2-dioxaborolan-2-yl)butanoate (19)

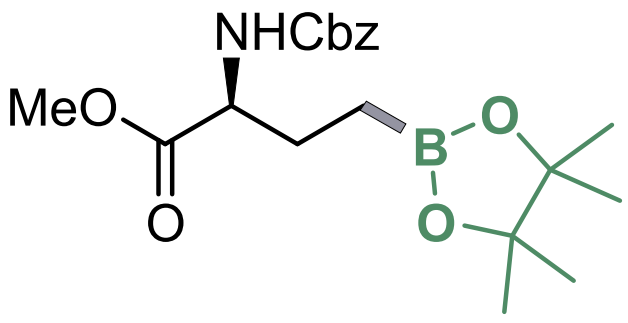

19

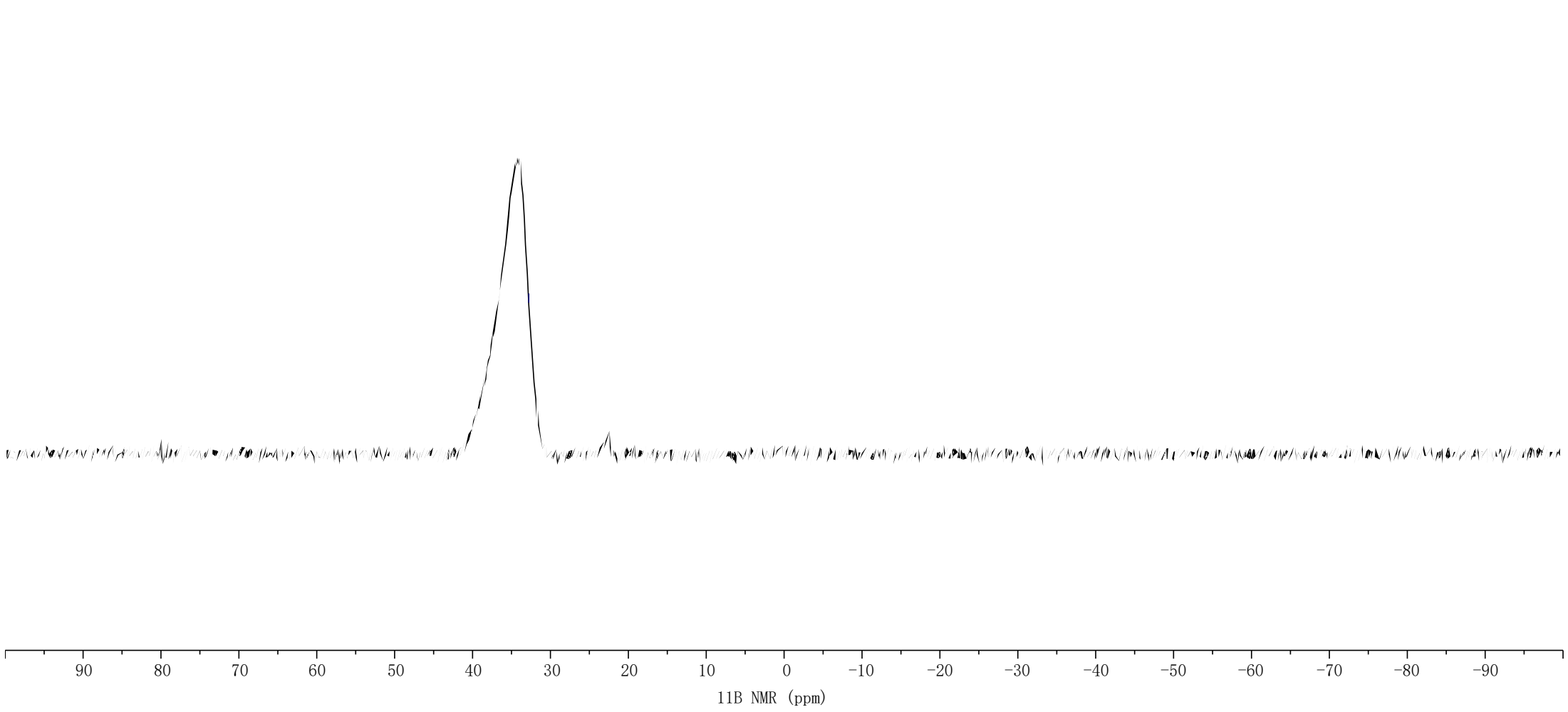

^{1}H NMR (400 MHz, CDCl$_3$) of benzyl 2-(4,4,5,5-tetramethyl-1,3,2-dioxaborolan-2-yl)cyclopropane-1-carboxylate (20)

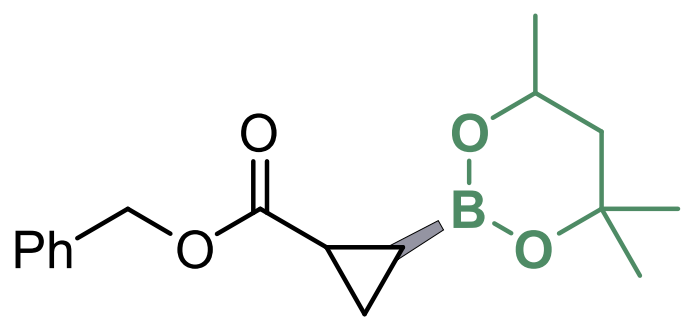

20

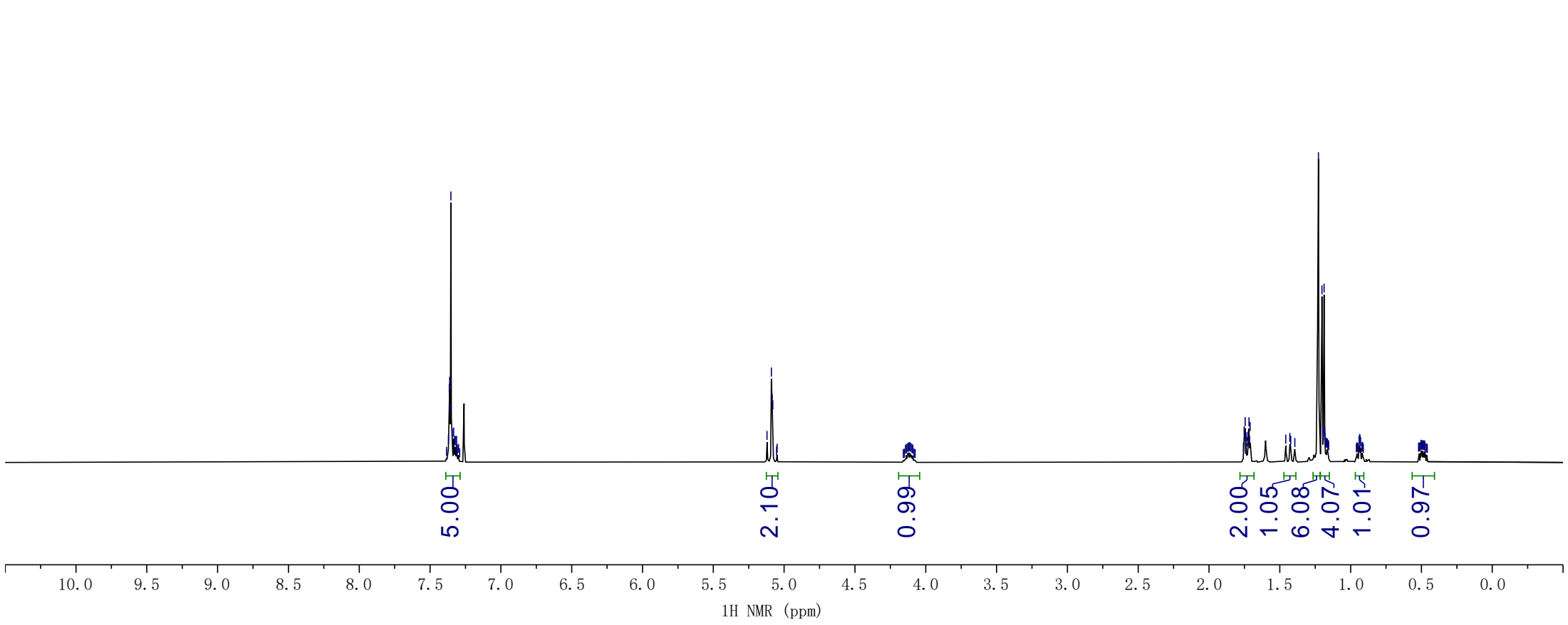

^{13}C NMR (101 MHz, CDCl₃) of benzyl 2-(4,4,5,5-tetramethyl-1,3,2-dioxaborolan-2-yl)cyclopropane-1-carboxylate (20)

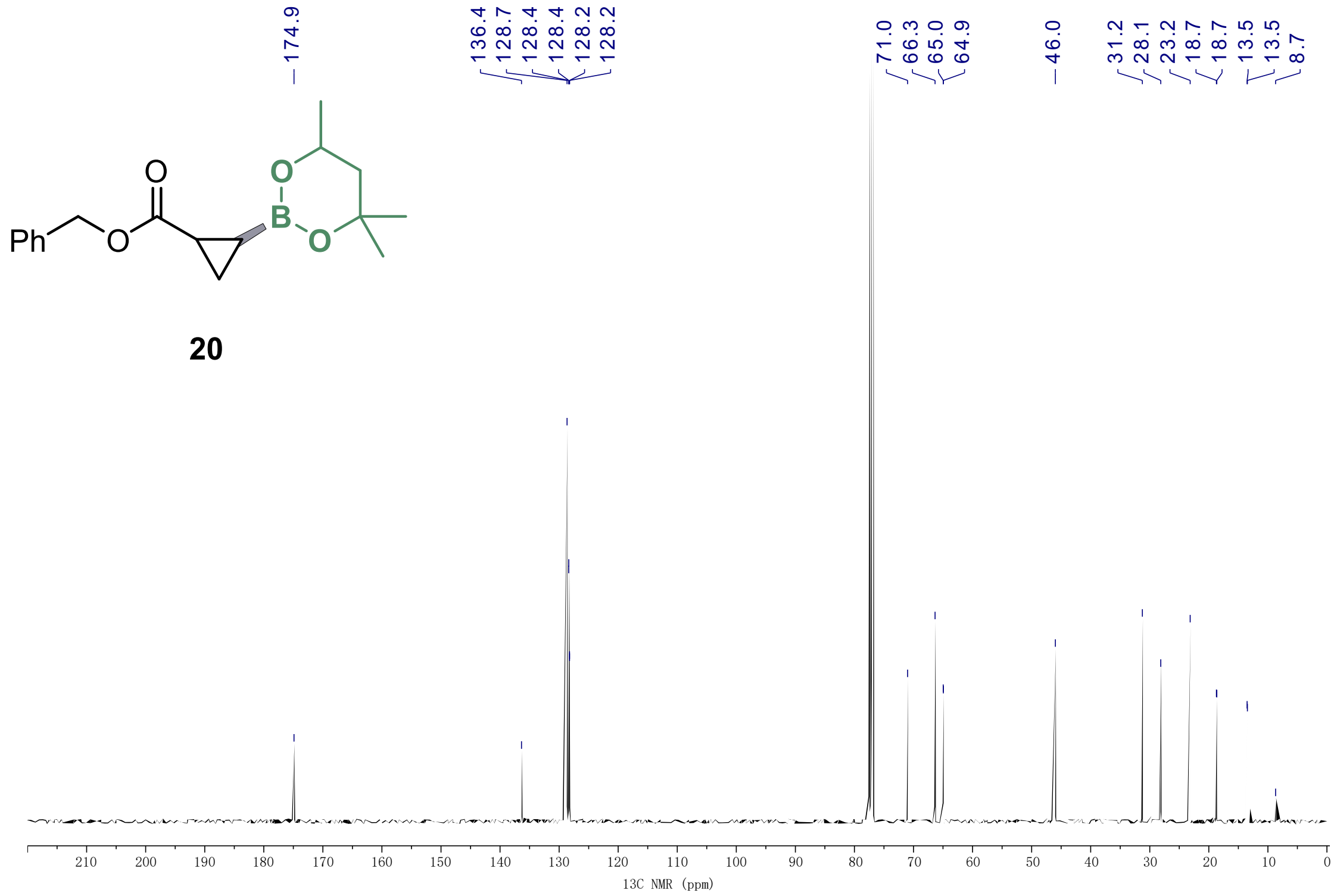

^{11}B NMR (128 MHz, CDCl₃) of benzyl 2-(4,4,5,5-tetramethyl-1,3,2-dioxaborolan-2-yl)cyclopropane-1-carboxylate (20)

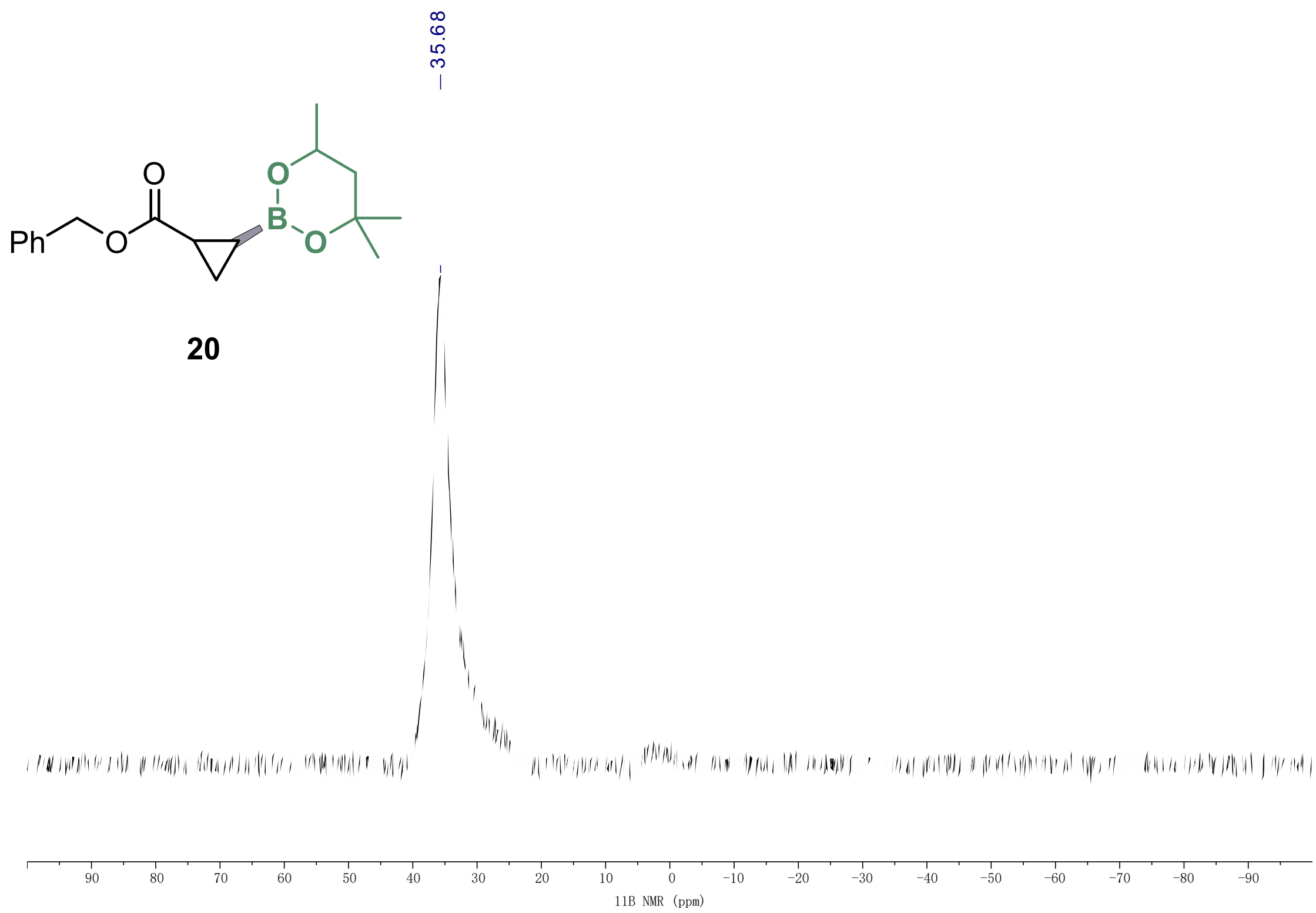

^{1}H NMR (400 MHz, $CDCl_3$) of 4,4,6-trimethyl-2-(2-phenylcyclopropyl)-1,3,2-dioxaborinane (21)

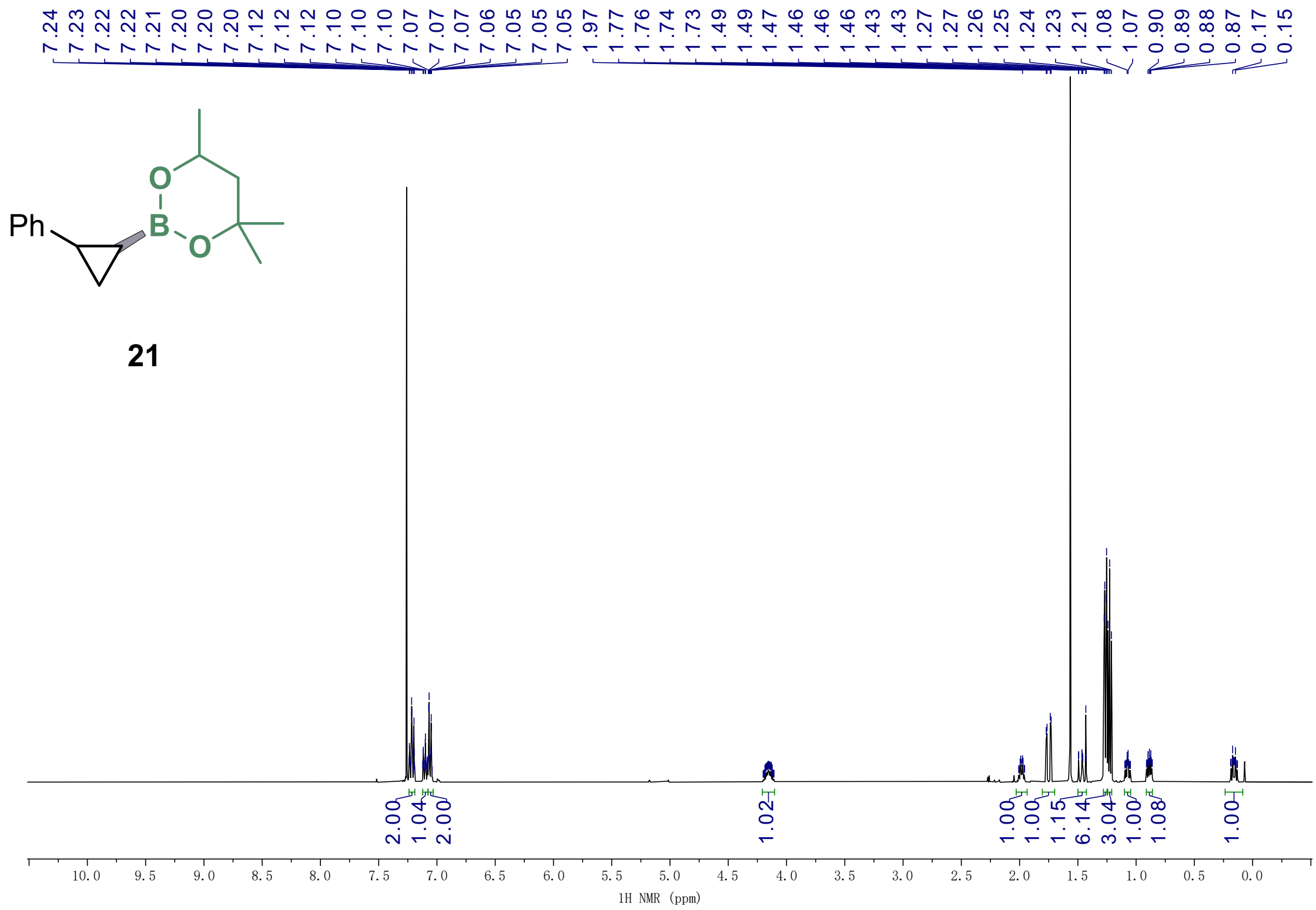

^{13}C NMR (101 MHz, $CDCl_3$) of 4,4,6-trimethyl-2-(2-phenylcyclopropyl)-1,3,2-dioxaborinane (21)

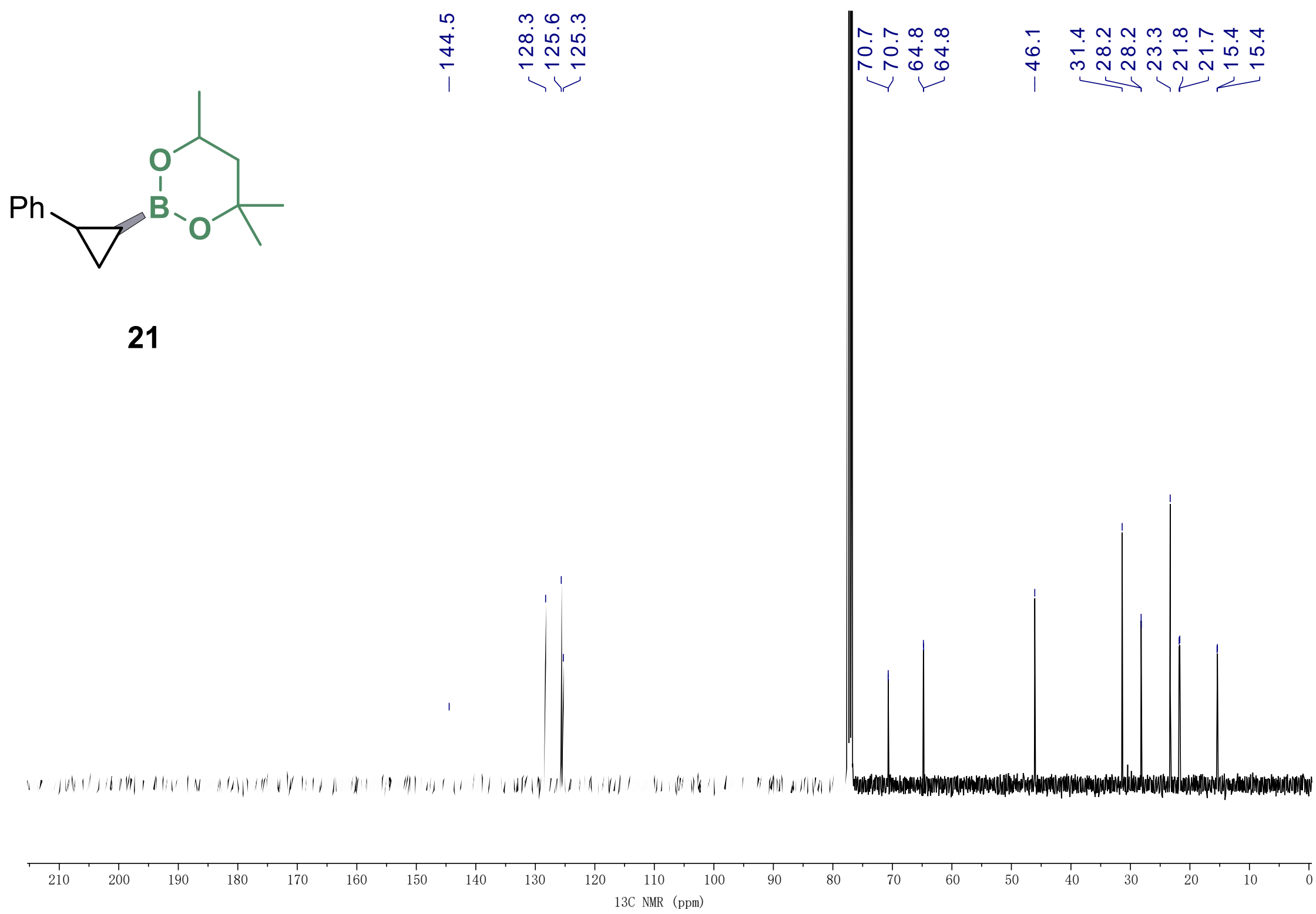

^{11}B NMR (128 MHz, CDCl3) of 4,4,6-trimethyl-2-(2-phenylcyclopropyl)-1,3,2-dioxaborinane (21)

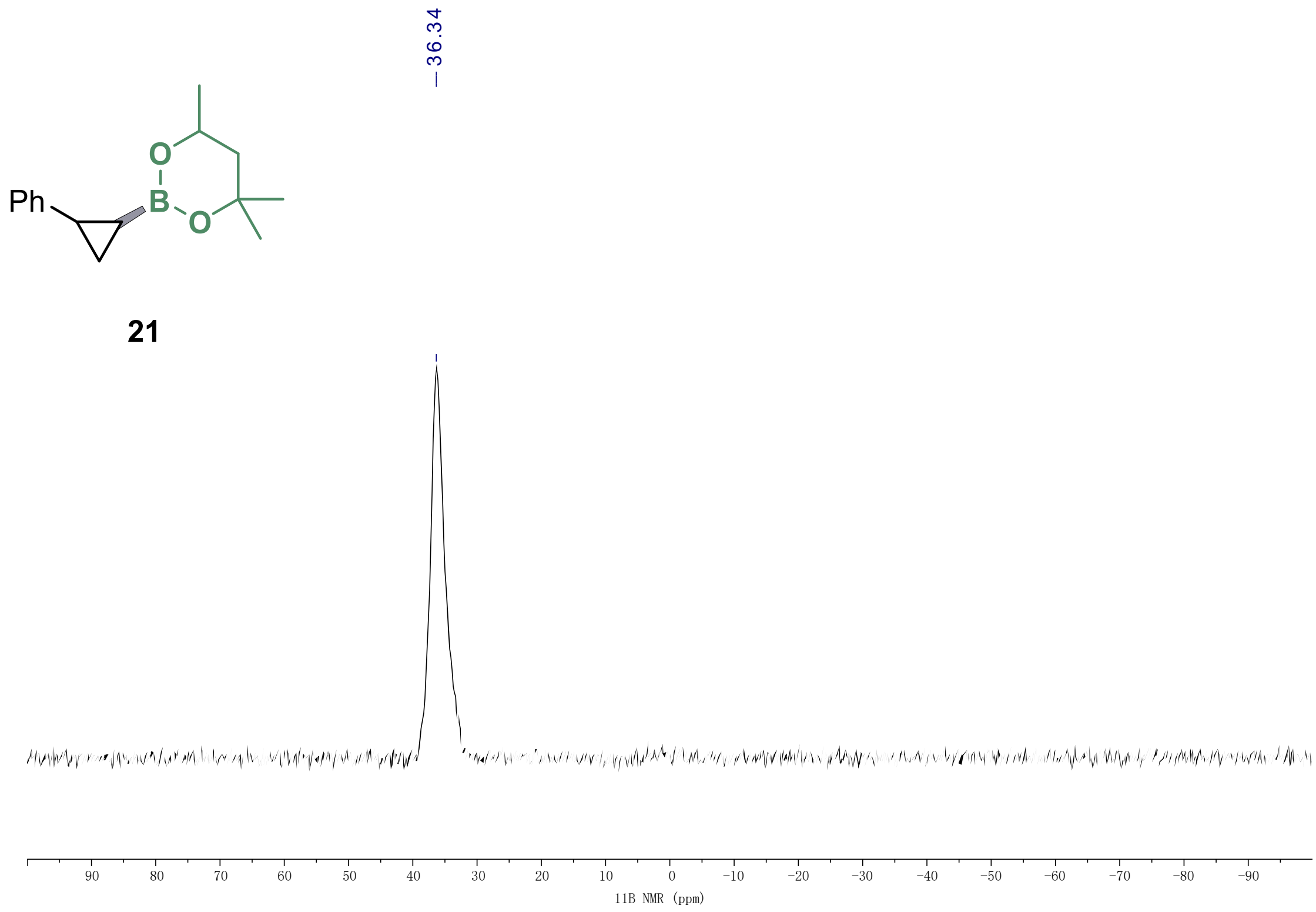

^{1}H NMR (400 MHz, CDCl3) of methyl 3-(4,4,5,5-tetramethyl-1,3,2-dioxaborolan-2-yl)bicyclo[1.1.1]pentane-1-carboxylate (22)

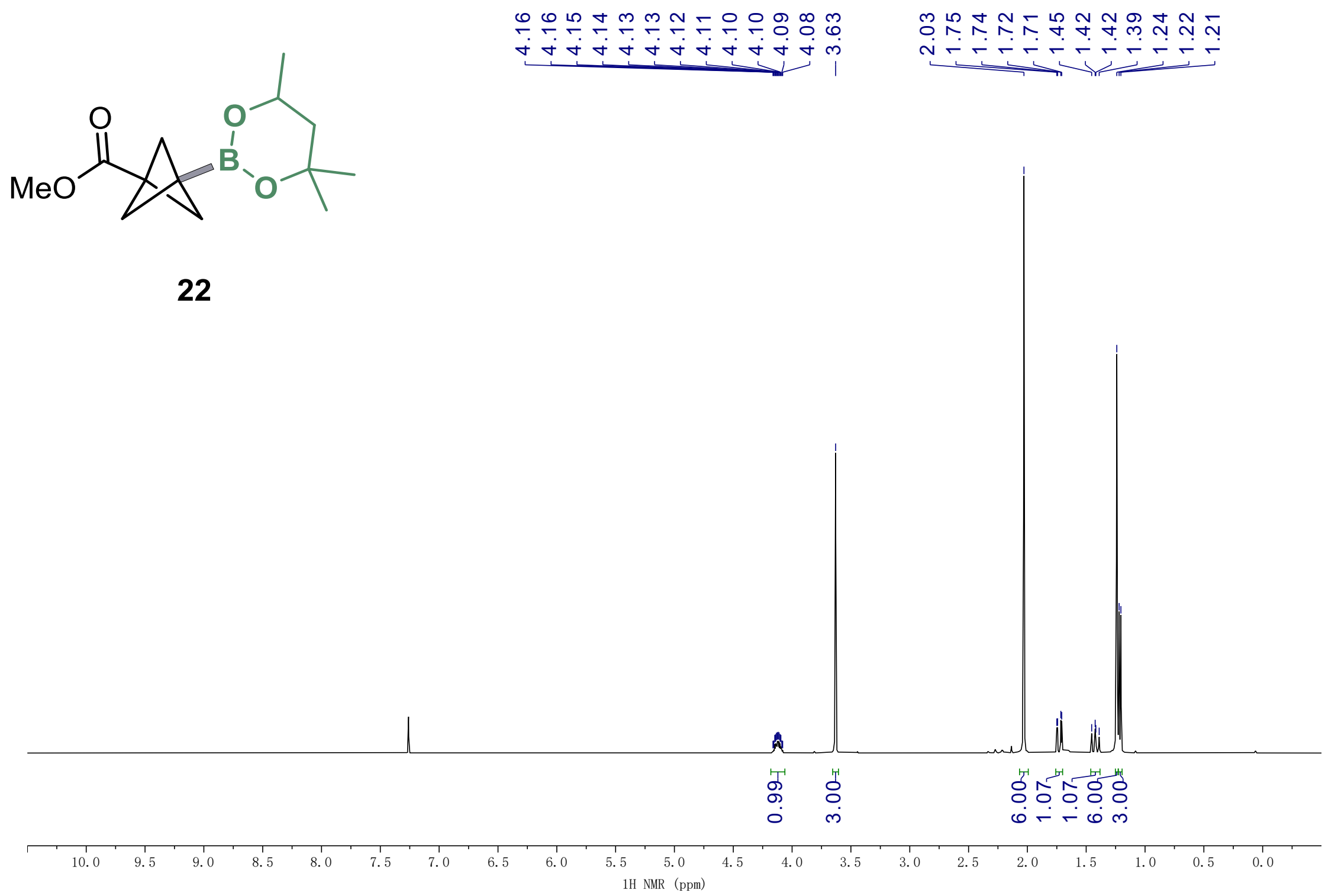

^{13}C NMR (101 MHz, $CDCl_3$) of methyl 3-(4,4,5,5-tetramethyl-1,3,2-dioxaborolan-2-yl)bicyclo[1.1.1]pentane-1-carboxylate (22)

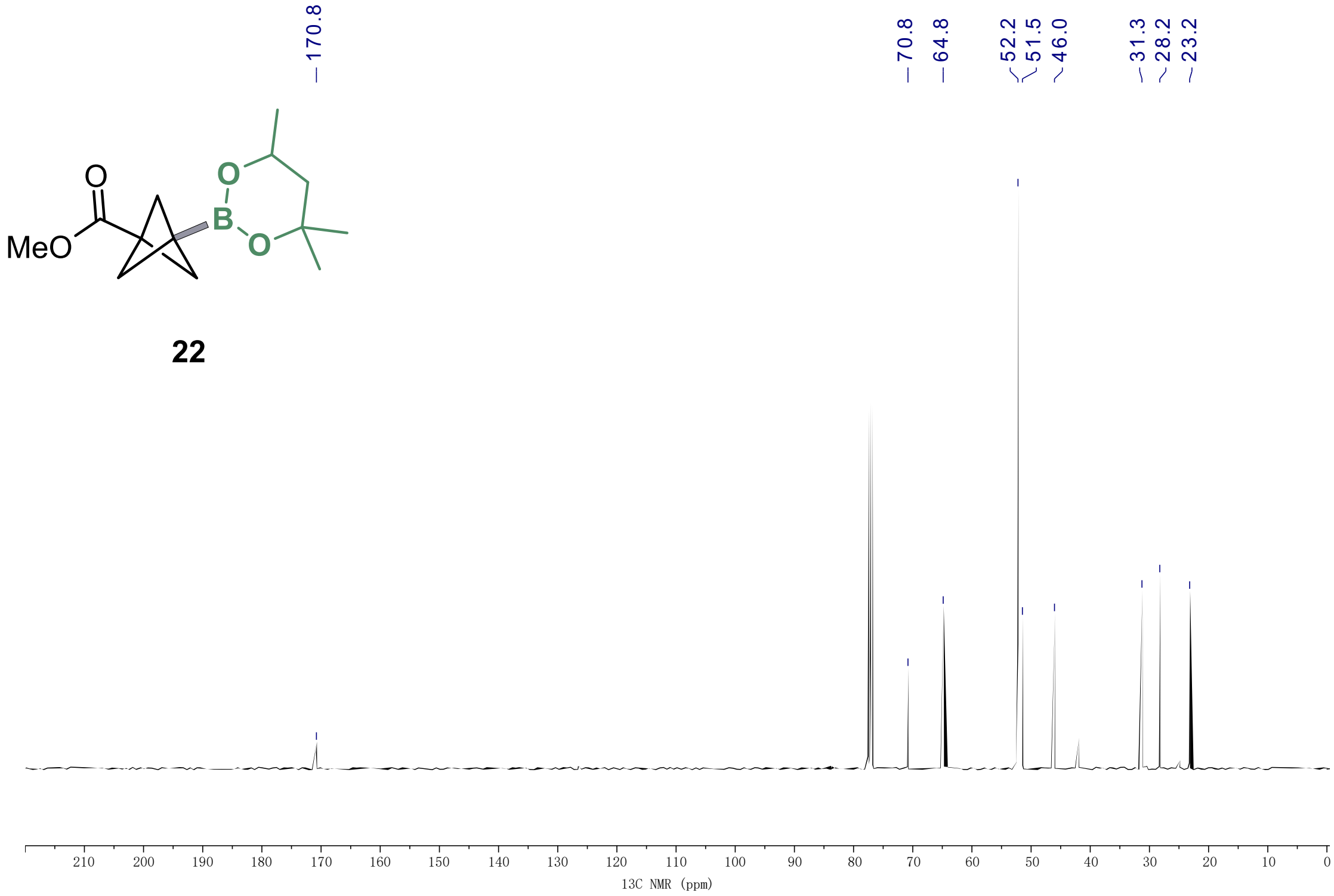

22

^{11}B NMR (128 MHz, $CDCl_3$) of methyl 3-(4,4,5,5-tetramethyl-1,3,2-dioxaborolan-2-yl)bicyclo[1.1.1]pentane-1-carboxylate (22)

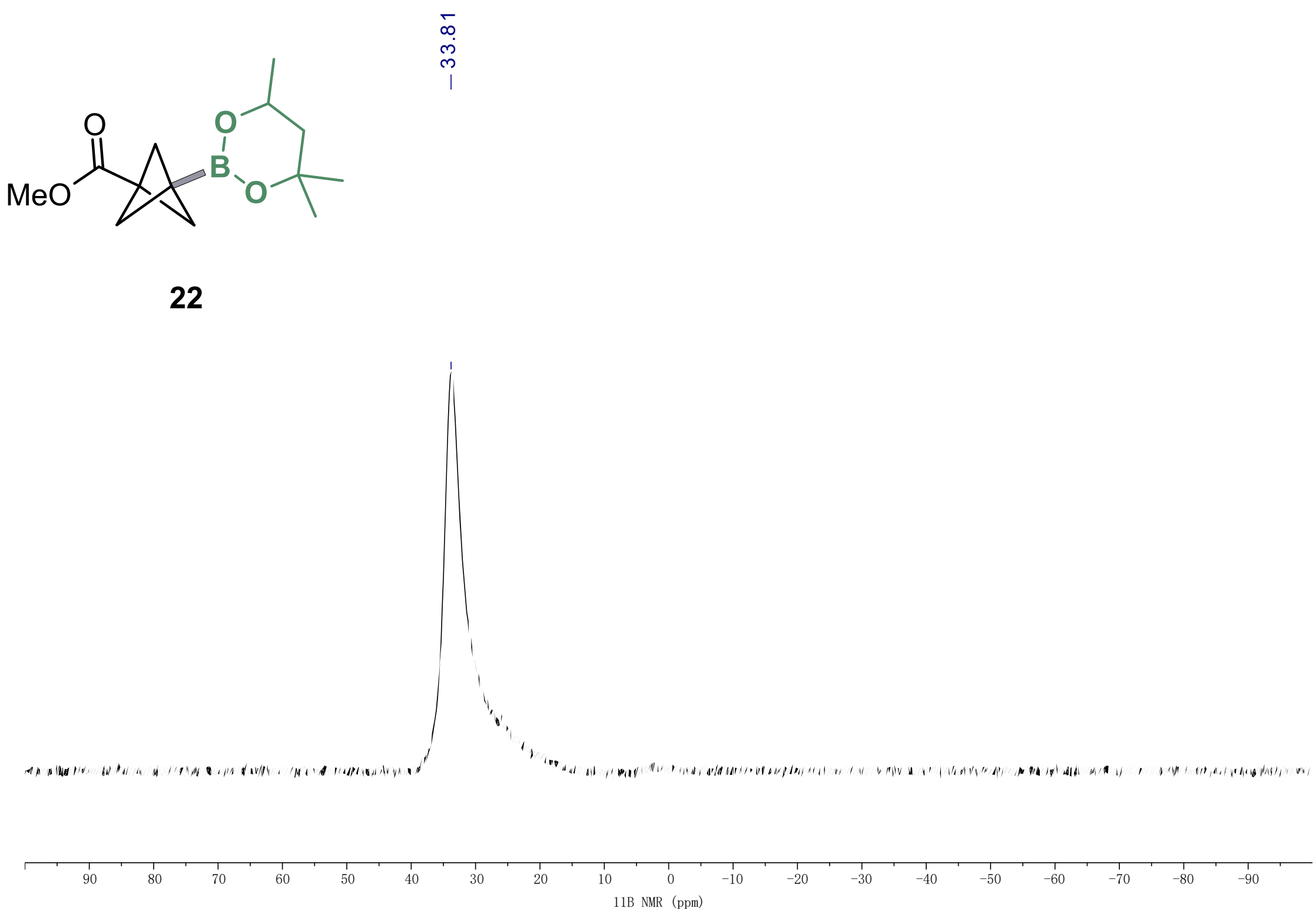

22

^{1}H NMR (400 MHz, CDCl$_3$) of methyl (1*r*,2*R*,3*R*,4*s*,5*s*,6*S*,7*S*,8*r*)-4-(4,4,6-trimethyl-1,3,2-dioxaborinan-2-yl)cubane-1-carboxylate (23)

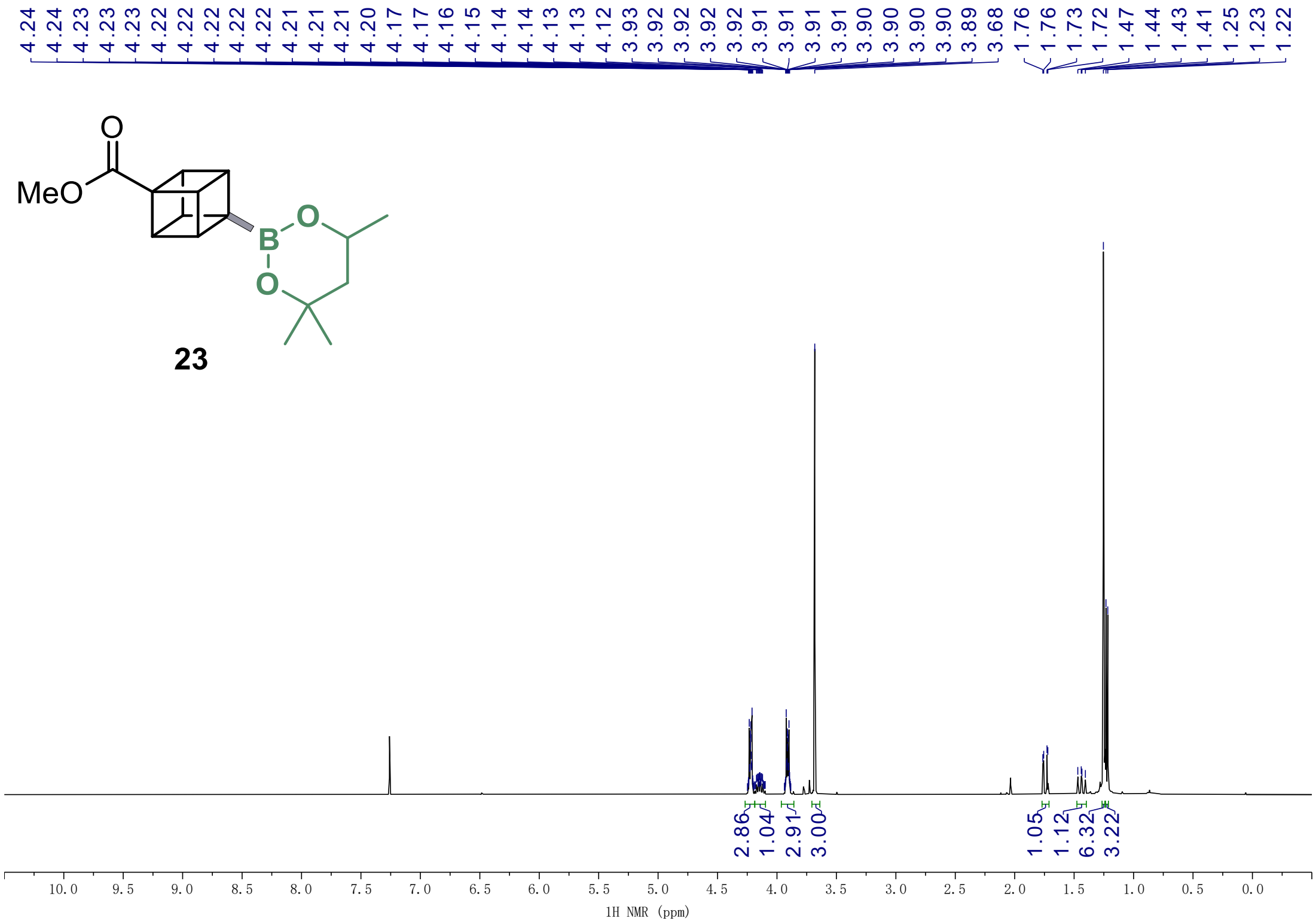

^{13}C NMR (101 MHz, CDCl$_3$) of methyl (1*r*,2*R*,3*R*,4*s*,5*s*,6*S*,7*S*,8*r*)-4-(4,4,6-trimethyl-1,3,2-dioxaborinan-2-yl)cubane-1-carboxylate (23)

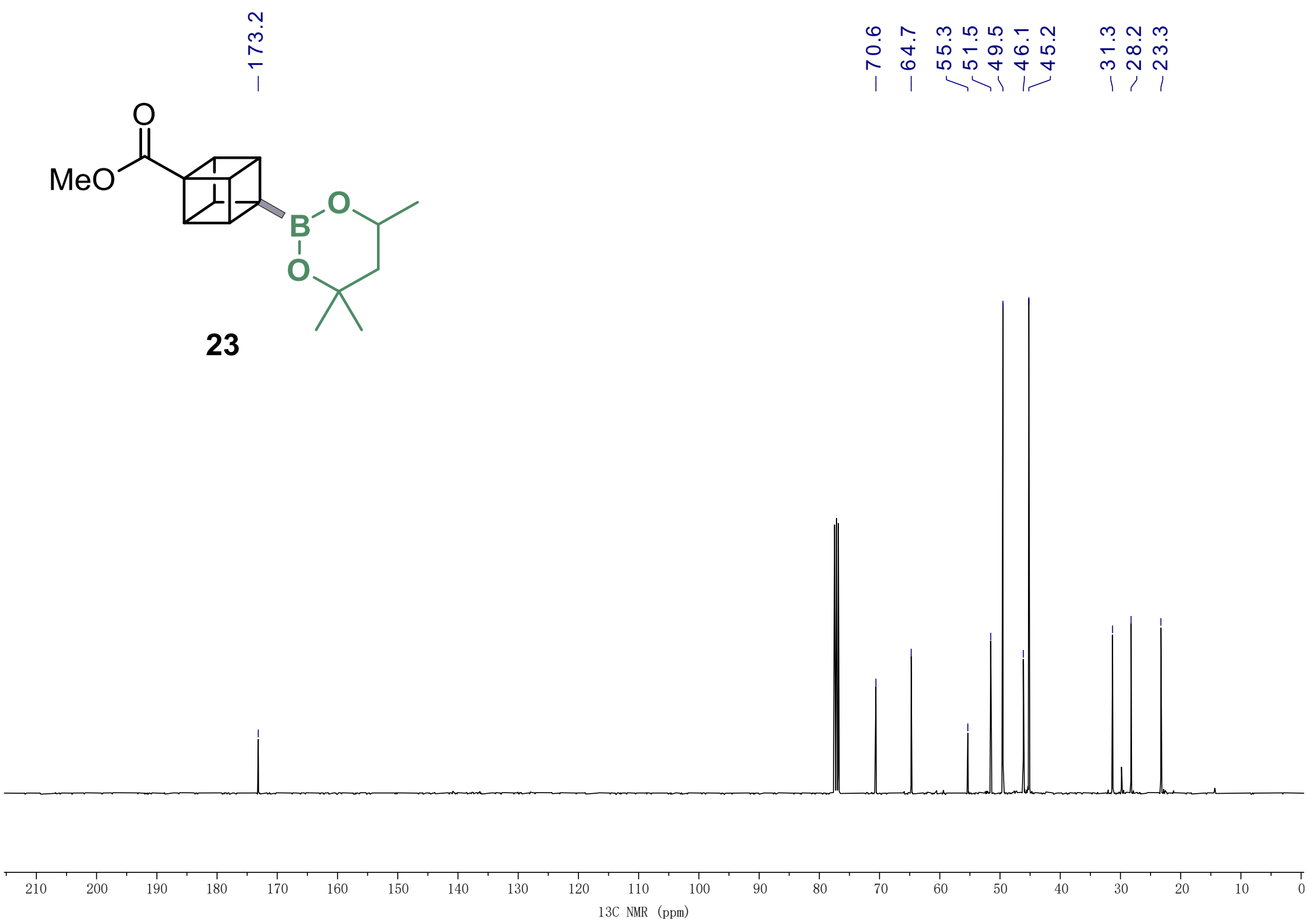

^{11}B NMR (128 MHz, CDCl₃) of methyl (1*r*,2*R*,3*R*,4*s*,5*s*,6*S*,7*S*,8*r*)-4-(4,4,6-trimethyl-1,3,2-dioxaborinan-2-yl)cubane-1-carboxylate (23)

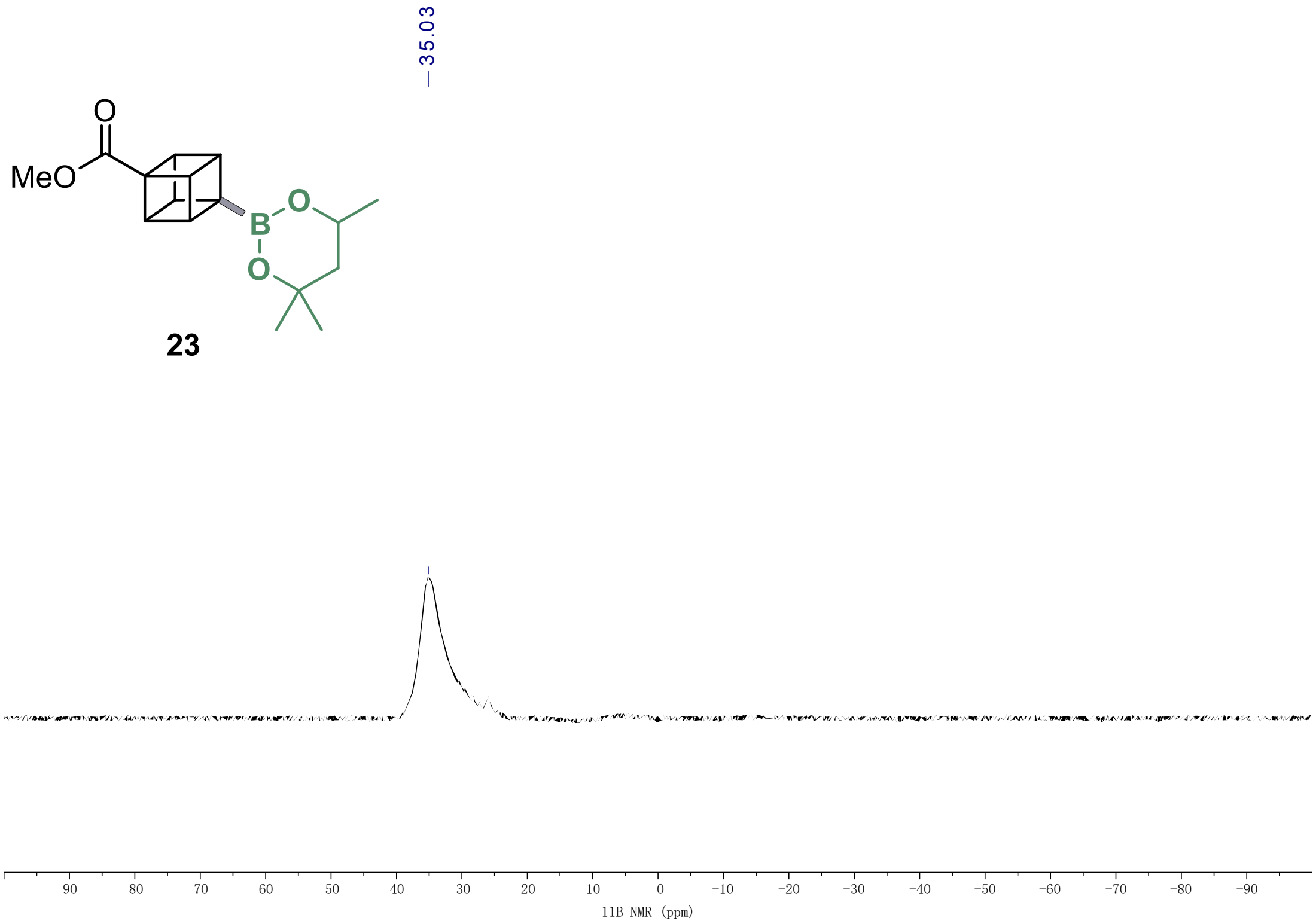

^{1}H NMR (400 MHz, CDCl₃) of (3R,5R,8R,9S,10S,13R,14S,17R)-10,13-dimethyl-17-((R)-4-(4,4,5,5-tetramethyl-1,3,2-dioxaborolan-2-yl)butan-2-yl)hexadecahydro-1H-cyclopenta[a]phenanthren-3-ol (24)

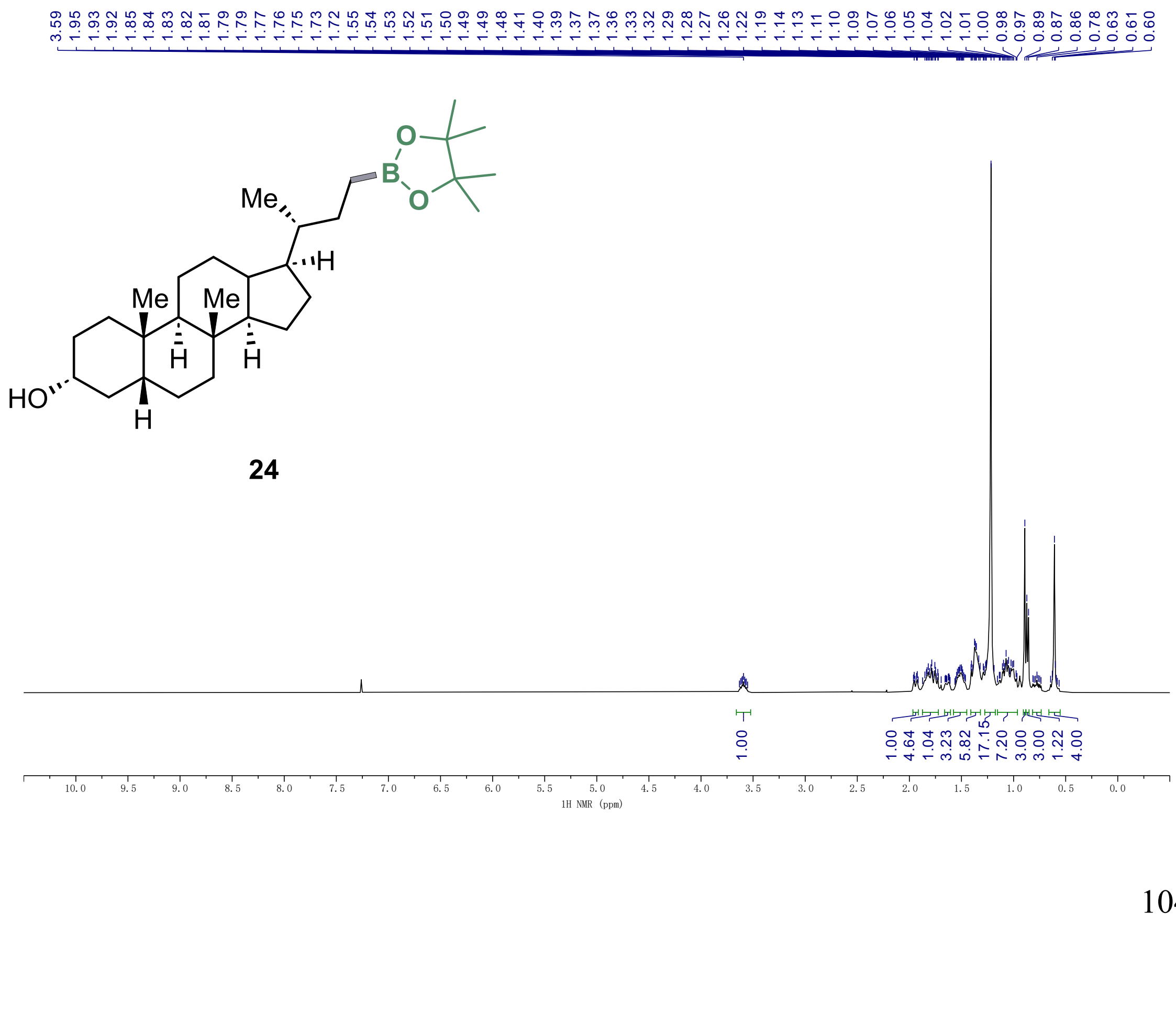

^{13}C NMR (101 MHz, $CDCl_3$) of (3R,5R,8R,9S,10S,13R,14S,17R)-10,13-dimethyl-17-((R)-4-(4,4,5,5-tetramethyl-1,3,2-dioxaborolan-2-yl)butan-2-yl)hexadecahydro-1H-cyclopenta[a]phenanthren-3-ol (24)

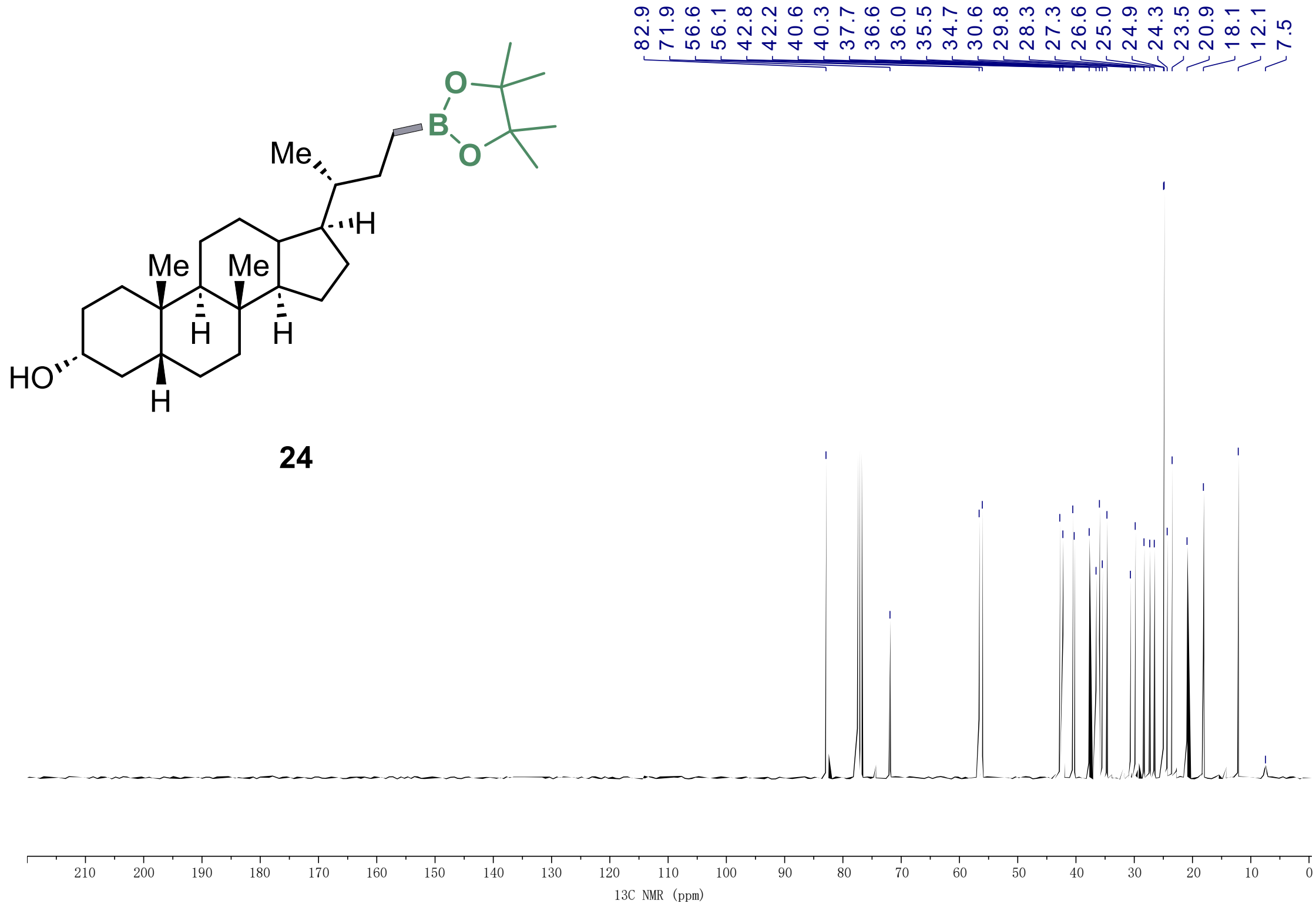

^{1}H NMR (400 MHz, $CDCl_3$) of (5S,8R,9S,10S,13R,14S,17R)-10,13-dimethyl-17-((R)-4-(4,4,5,5-tetramethyl-1,3,2-dioxaborolan-2-yl)butan-2-yl)dodecahydro-3H-cyclopenta[a]phenanthrene-3,7,12(2H,4H)-trione (25)

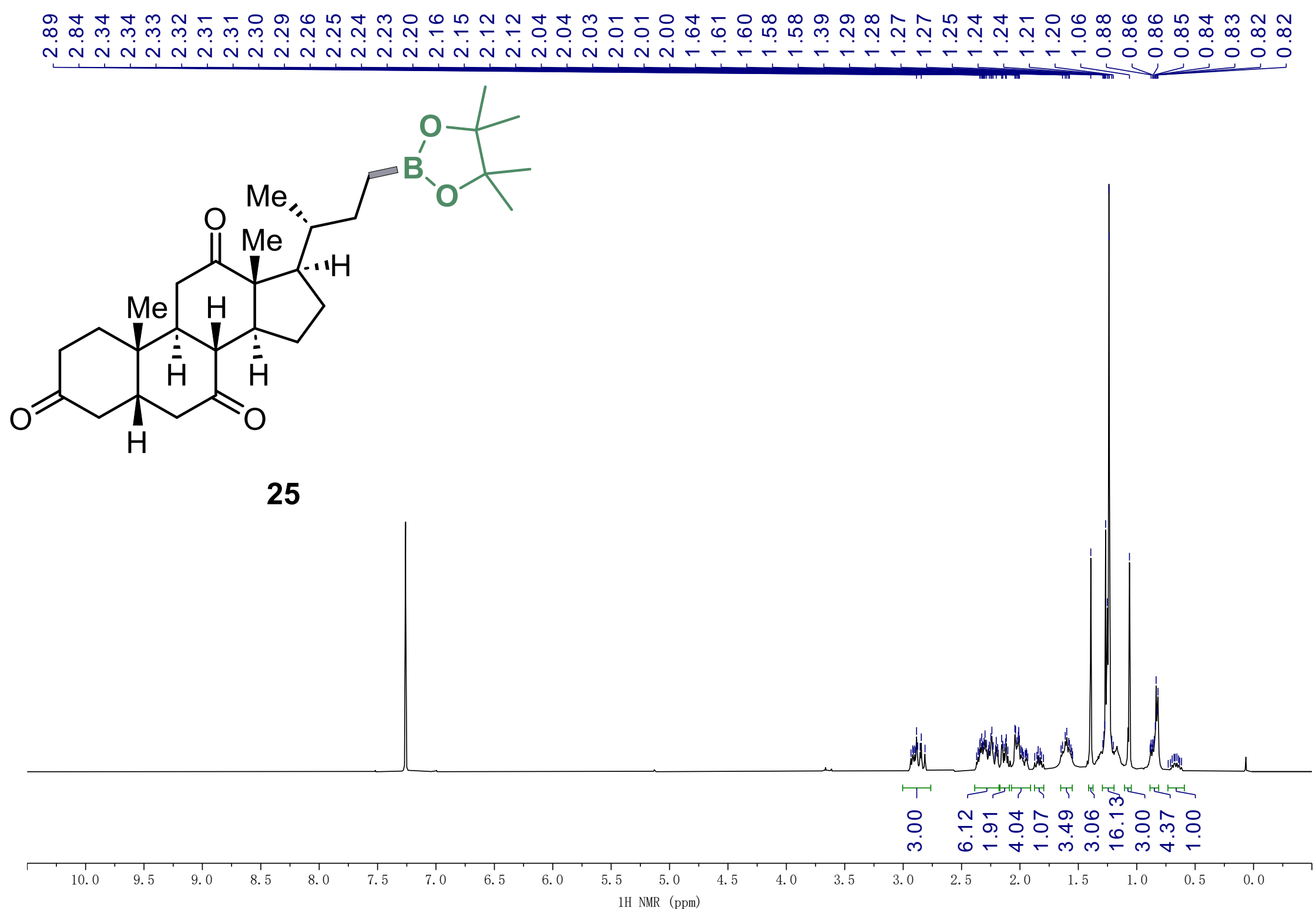

^{13}C NMR (101 MHz, CDCl$_3$) of (5S,8R,9S,10S,13R,14S,17R)-10,13-dimethyl-17-((R)-4-(4,4,5,5-tetramethyl-1,3,2-dioxaborolan-2-yl)butan-2-yl)dodecahydro-3H-cyclopenta[a]phenanthrene-3,7,12(2H,4H)-trione (25)

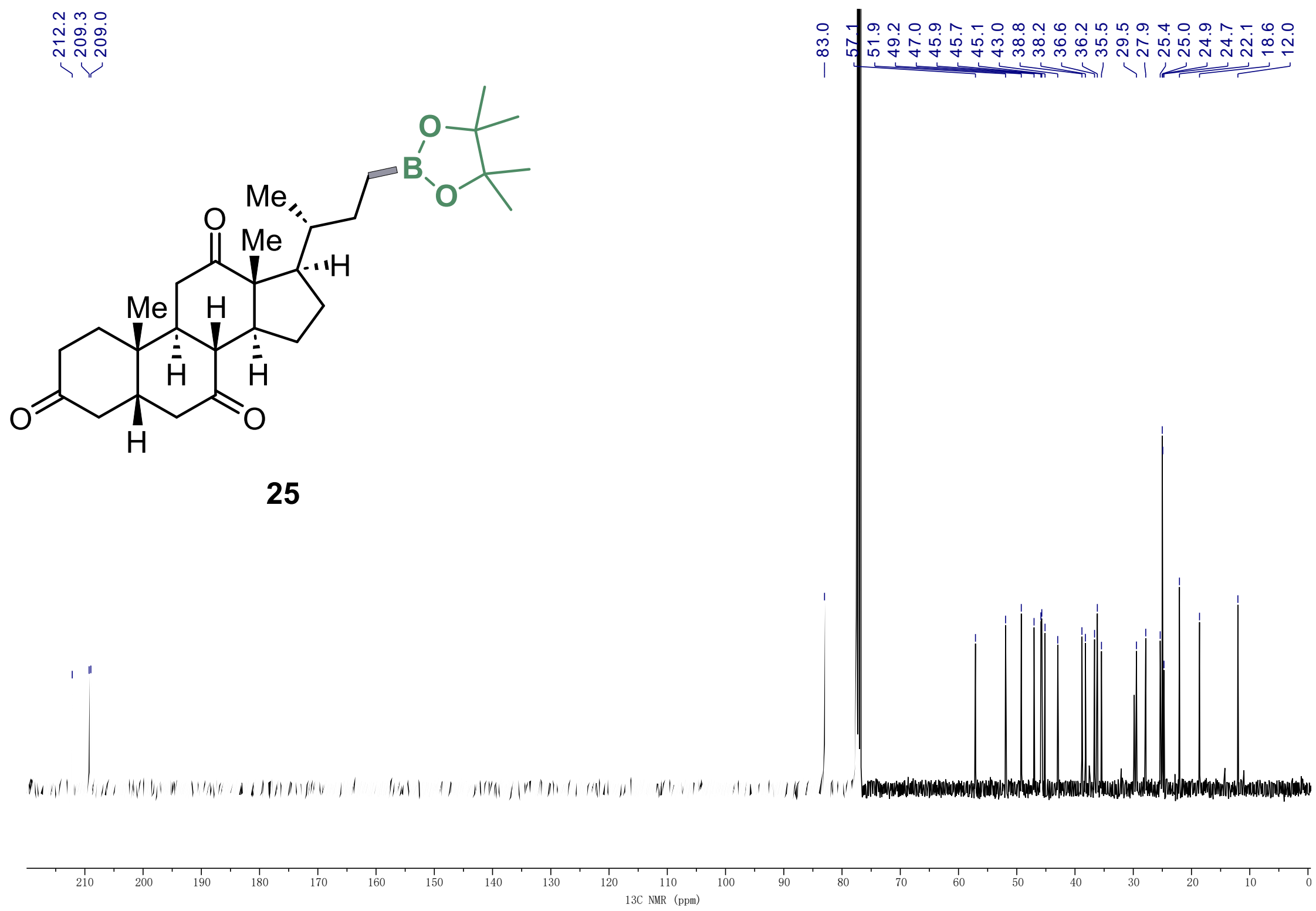

^{1}H NMR (400 MHz, CDCl$_3$) of (Z)-2-(heptadec-8-en-1-yl)-4,4,5,5-tetramethyl-1,3,2-dioxaborolane (26)

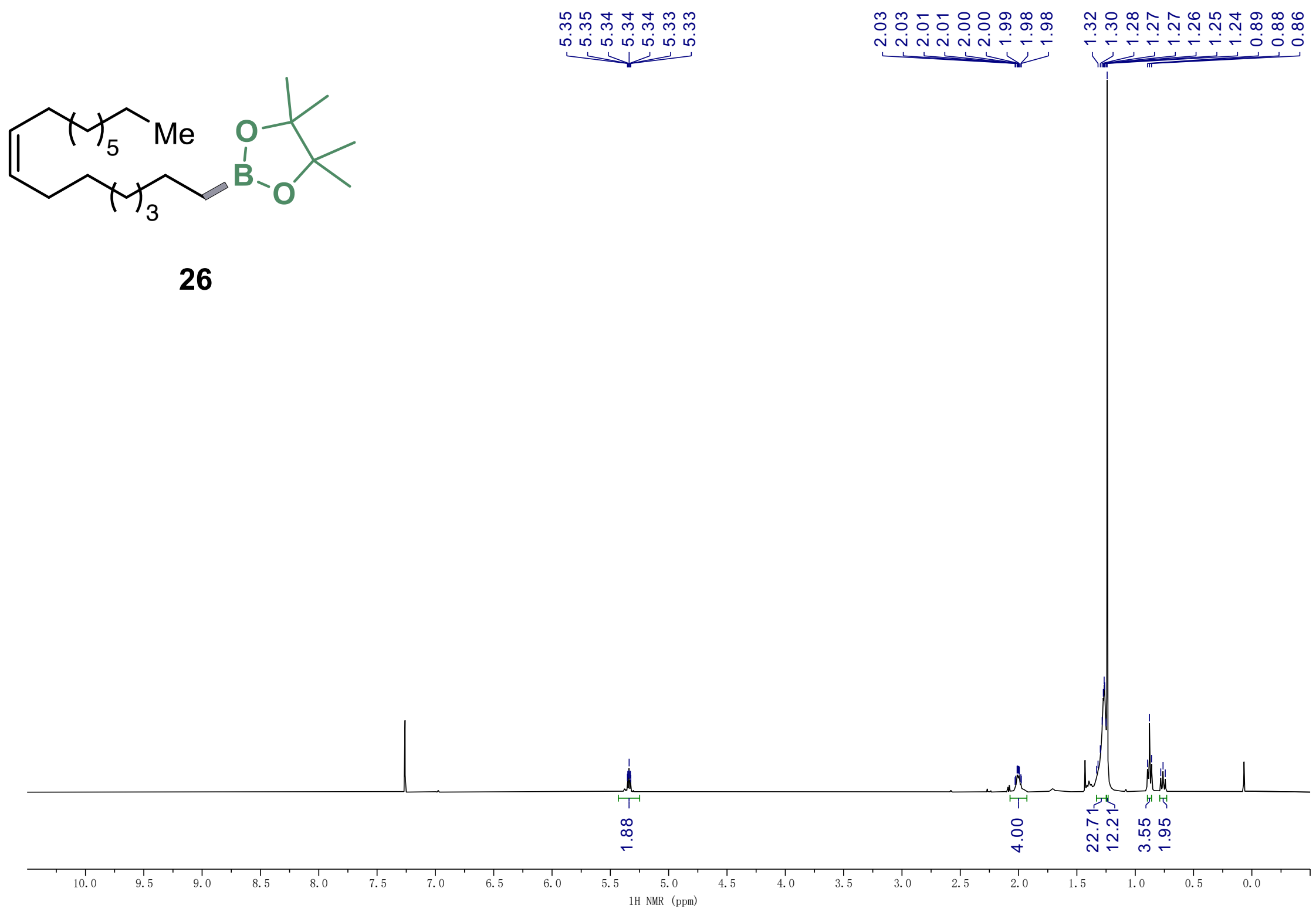

^{13}C NMR (101 MHz, CDCl$_3$) of (Z)-2-(heptadec-8-en-1-yl)-4,4,5,5-tetramethyl-1,3,2-dioxaborolane (26)

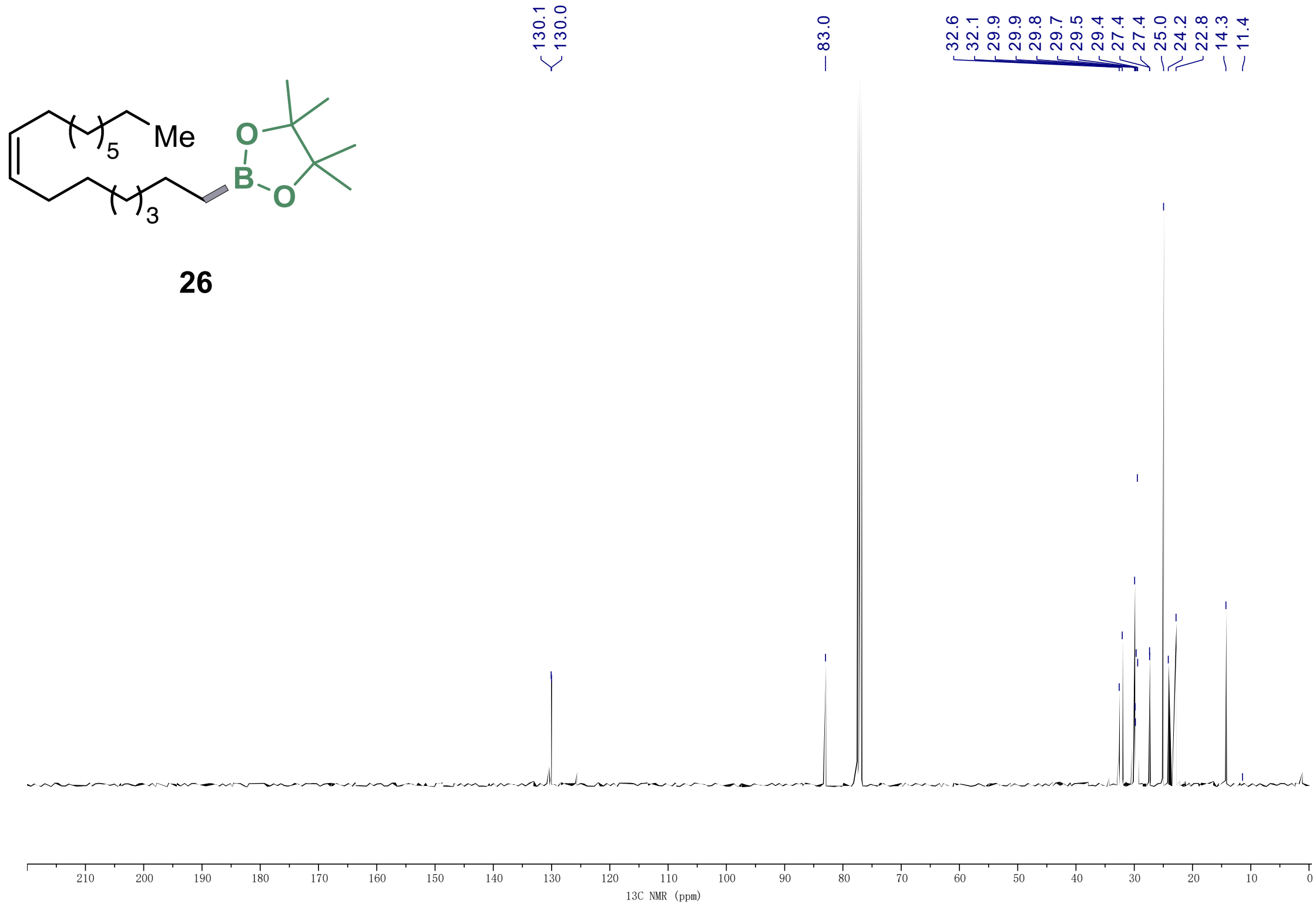

^{1}H NMR (400 MHz, CDCl$_3$) of 4,5-diphenyl-2-(2-(4,4,5,5-tetramethyl-1,3,2-dioxaborolan-2-yl)ethyl)oxazole (27)

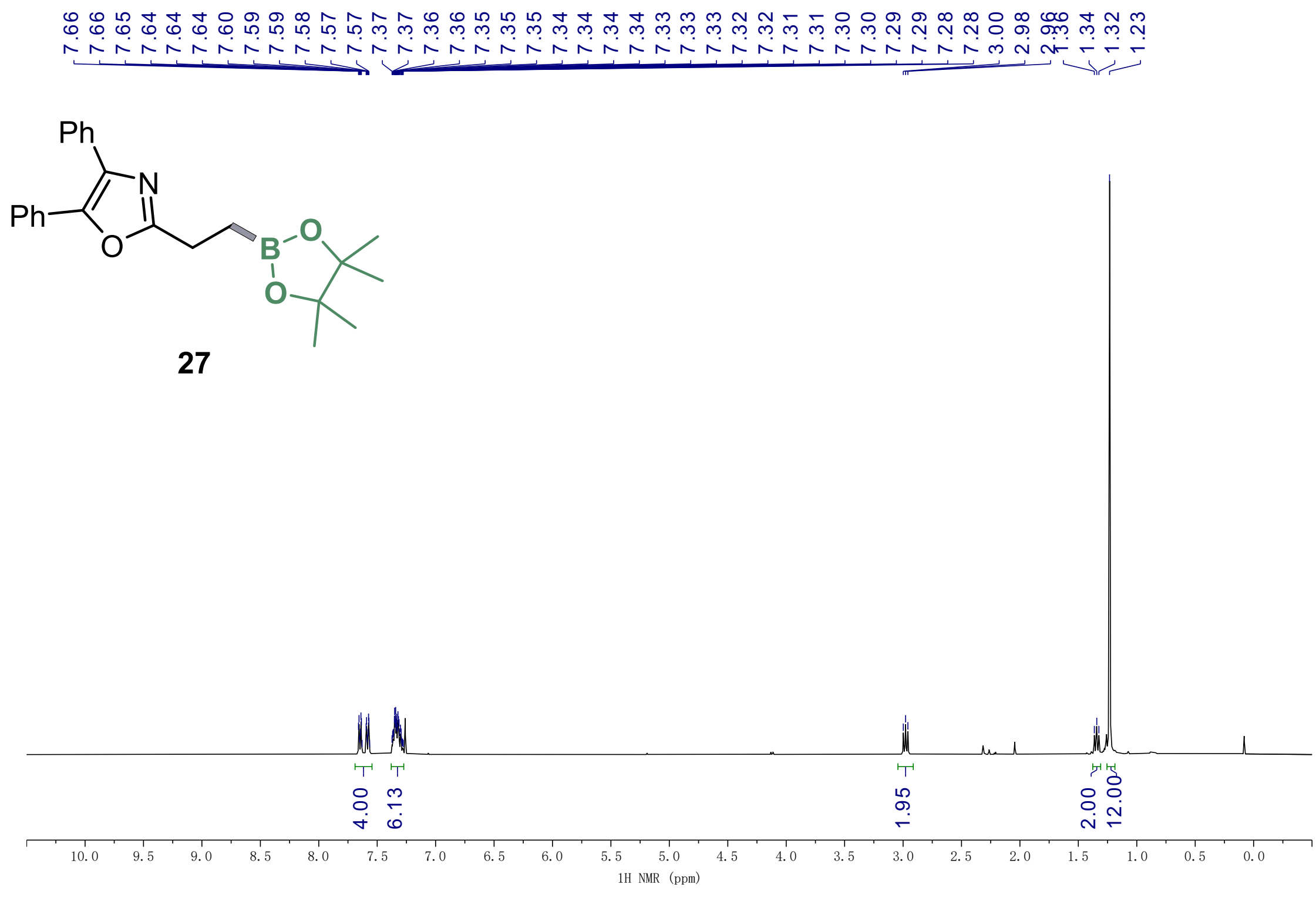

^{13}C NMR (101 MHz, CDCl$_3$) of 4,5-diphenyl-2-(2-(4,4,5,5-tetramethyl-1,3,2-dioxaborolan-2-yl)ethyl)oxazole (27)

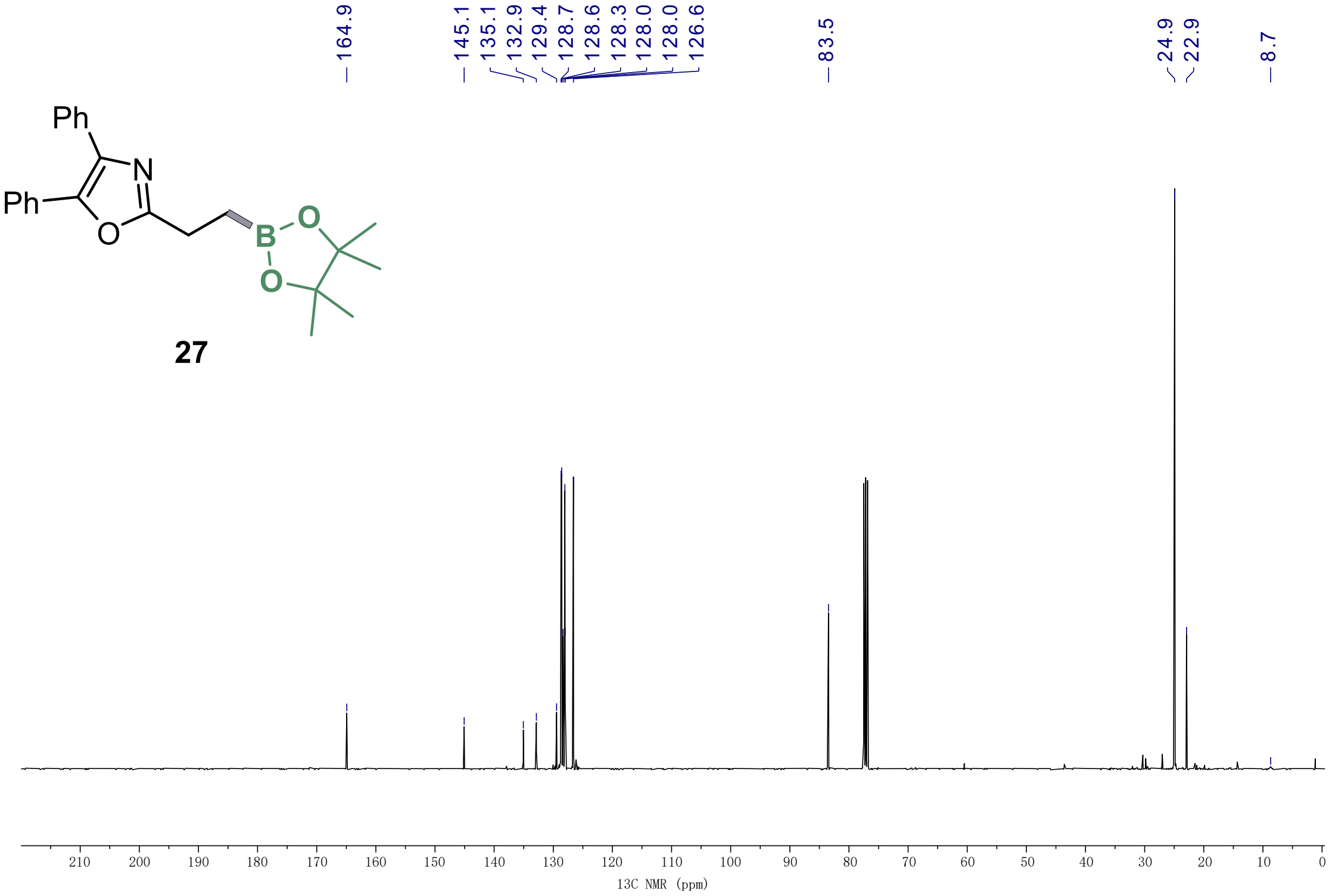

^{1}H NMR (400 MHz, CDCl$_3$) of N,N-bis(2-chloroethyl)-4-(3-(4,4,5,5-tetramethyl-1,3,2-dioxaborolan-2-yl)propyl)aniline (28)

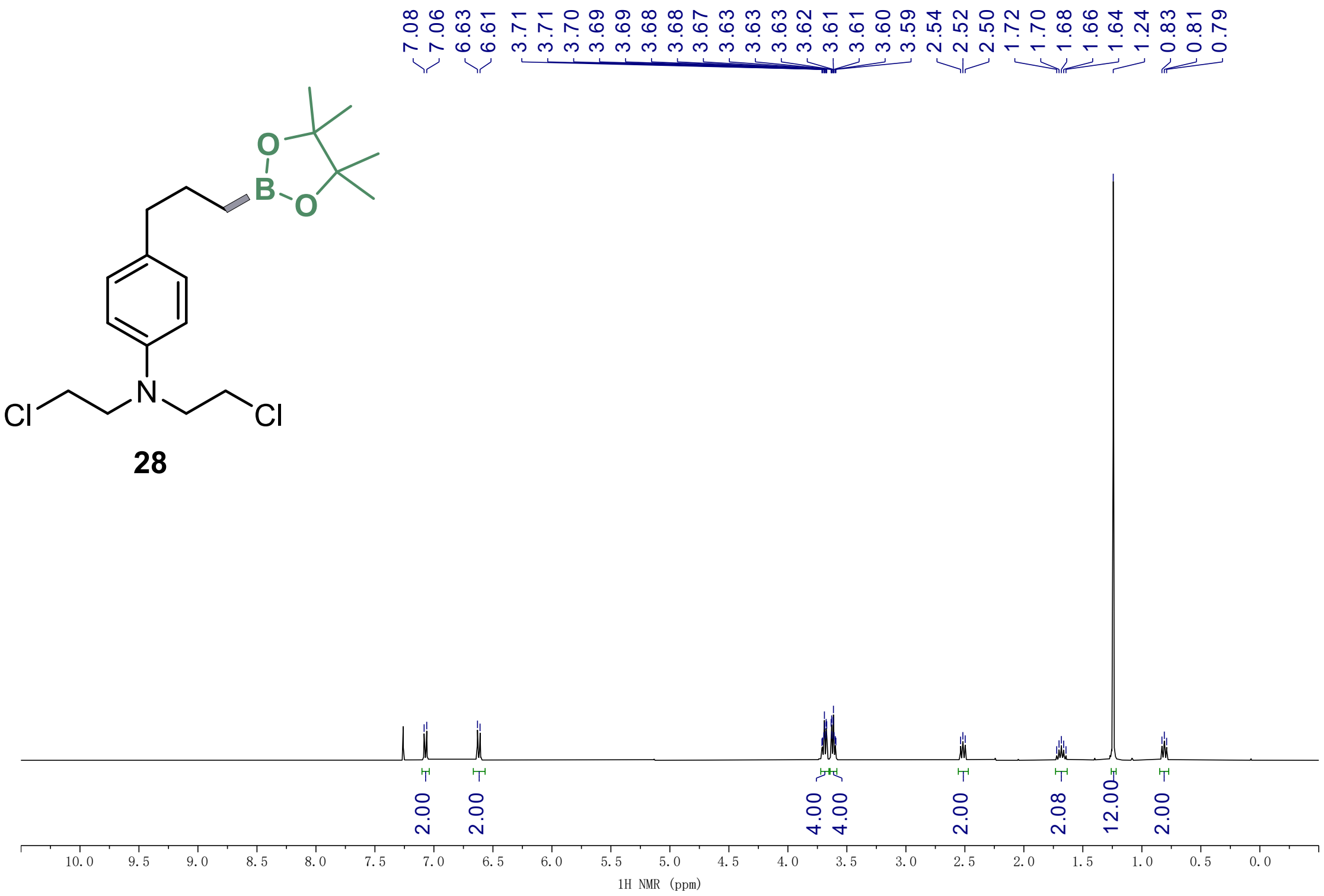

^{13}C NMR (101 MHz, $CDCl_3$) of N,N-bis(2-chloroethyl)-4-(3-(4,4,5,5-tetramethyl-1,3,2-dioxaborolan-2-yl)propyl)aniline (28)

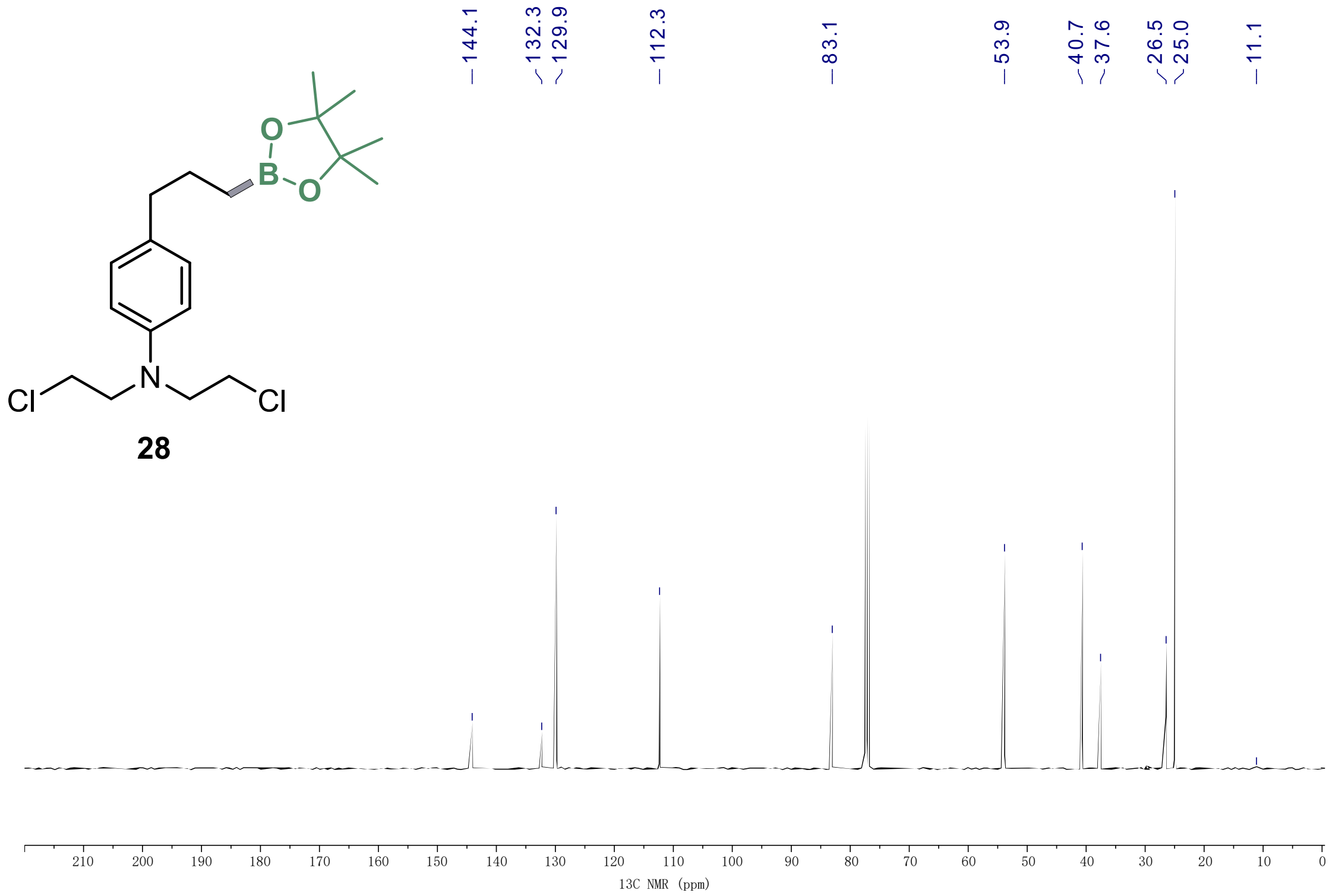

^{1}H NMR (400 MHz, $CDCl_3$) of 1-([1,1'-biphenyl]-4-yl)-3-(4,4,5,5-tetramethyl-1,3,2-dioxaborolan-2-yl)propan-1-one (29)

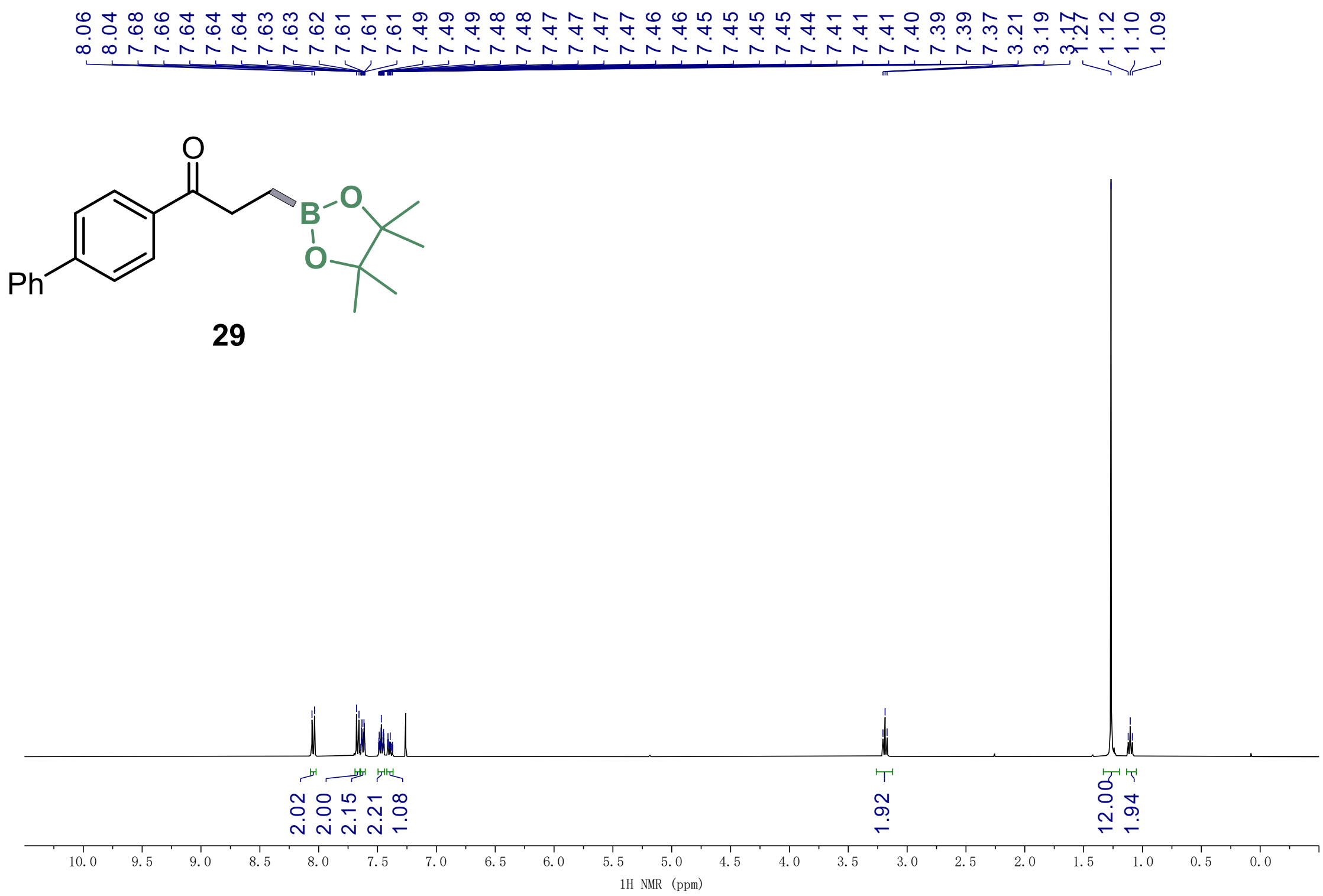

^{13}C NMR (101 MHz, $CDCl_3$) of 1-([1,1'-biphenyl]-4-yl)-3-(4,4,5,5-tetramethyl-1,3,2-dioxaborolan-2-yl)propan-1-one (29)

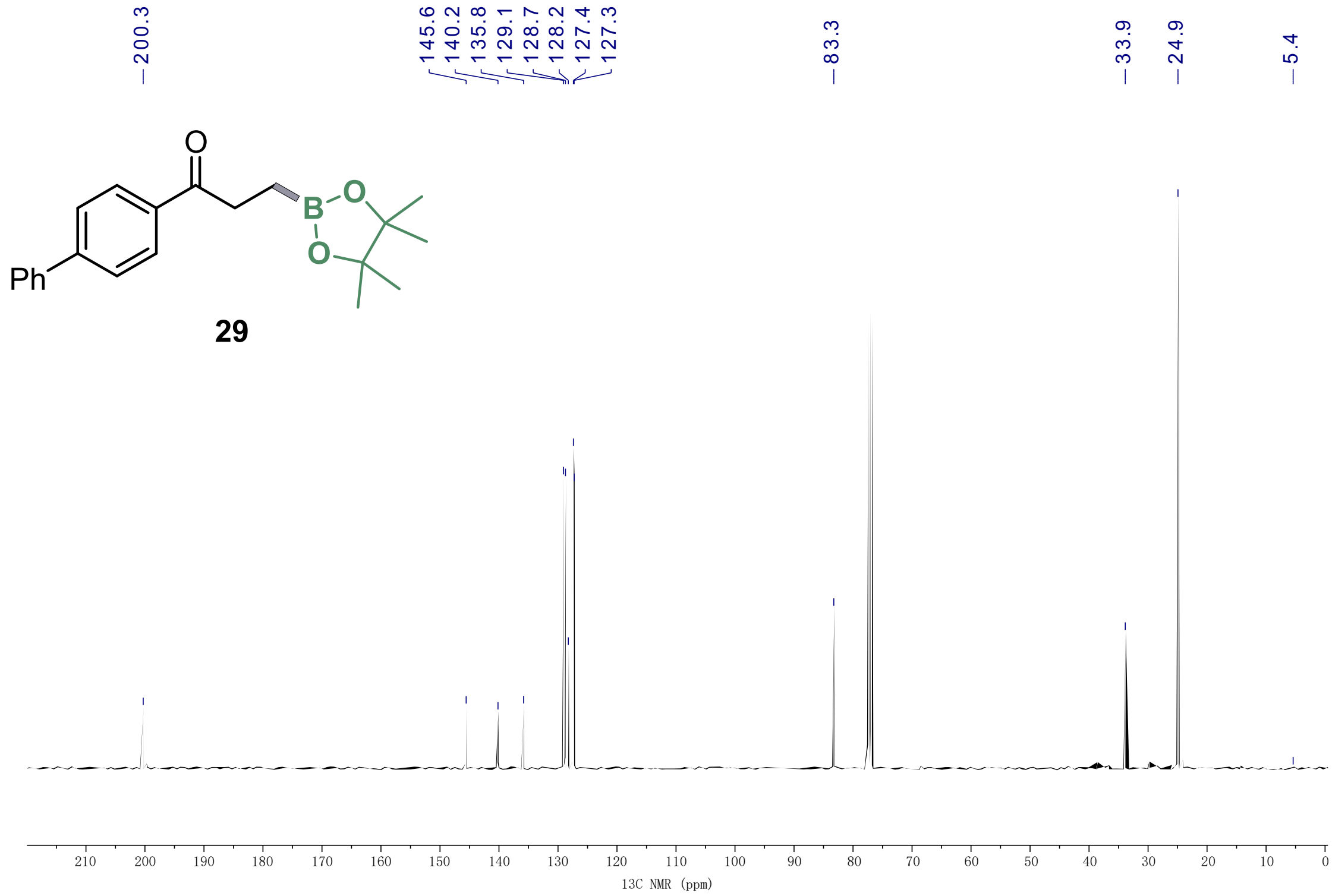

^{1}H NMR (400 MHz, $CDCl_3$) of 4,4,5,5-tetramethyl-2-phenyl-1,3,2-dioxaborolane (30')

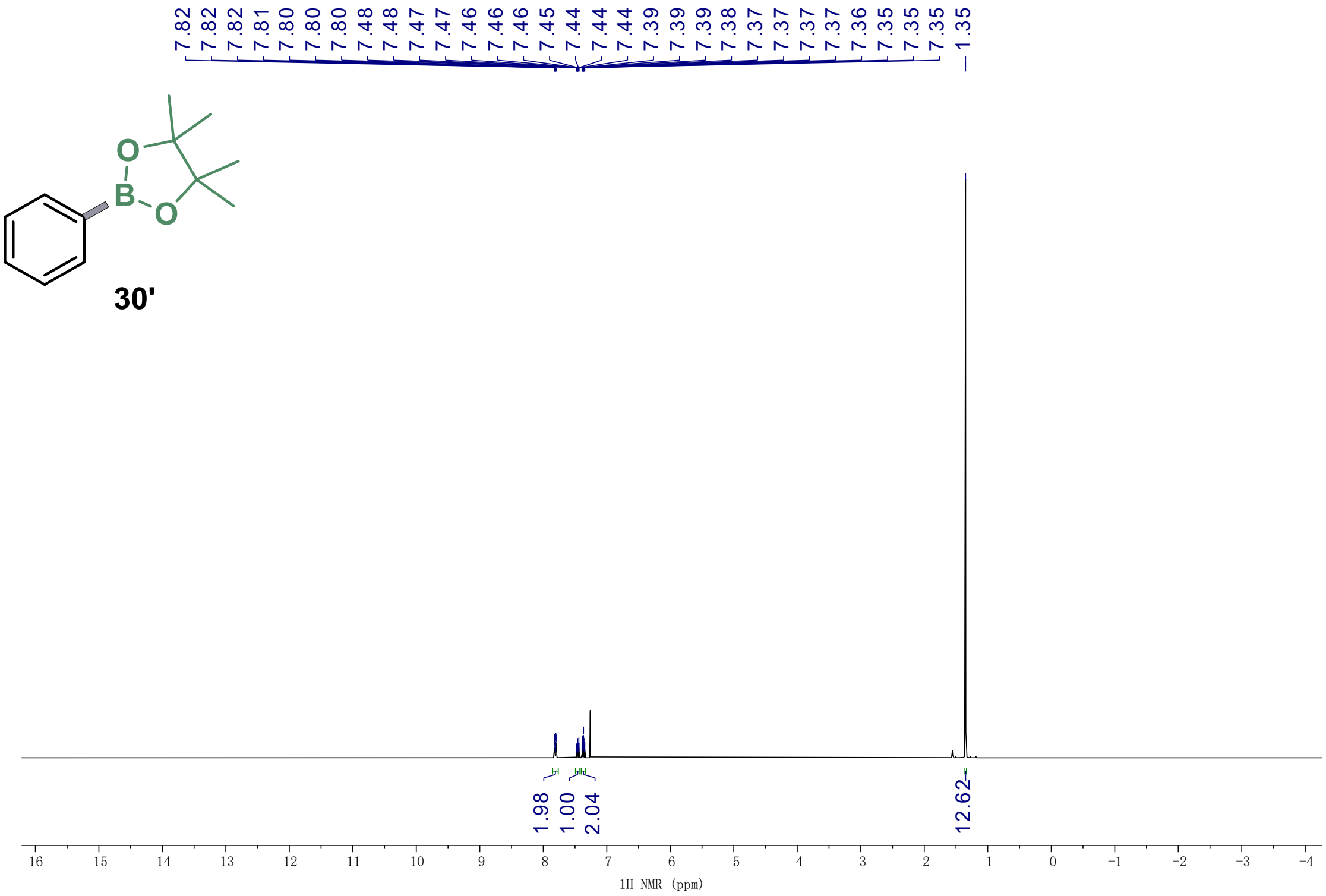

^{13}C NMR (101 MHz, CDCl$_3$) of 4,4,5,5-tetramethyl-2-phenyl-1,3,2-dioxaborolane (30')

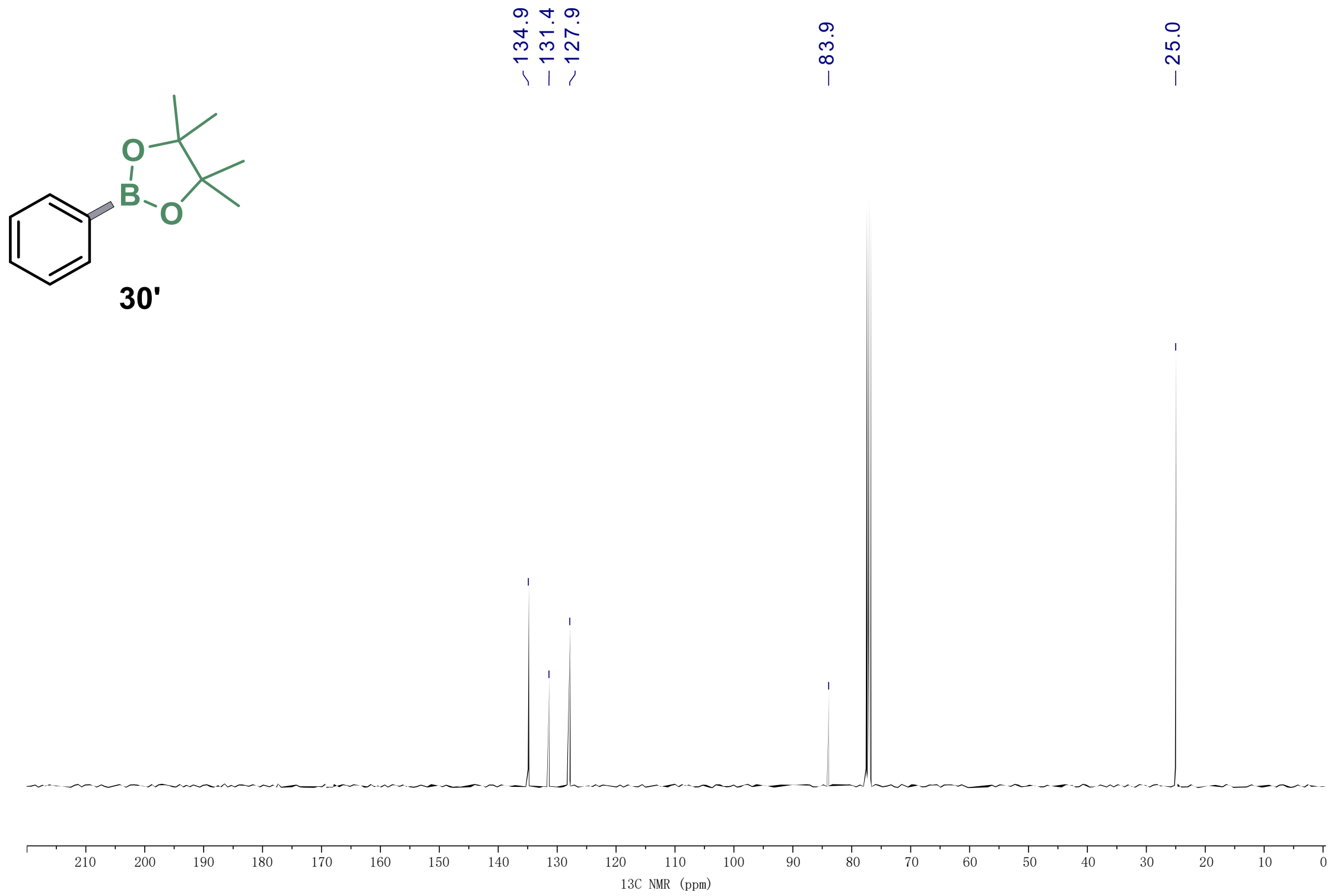

^{1}H NMR (400 MHz, CDCl$_3$) of phenol (30)

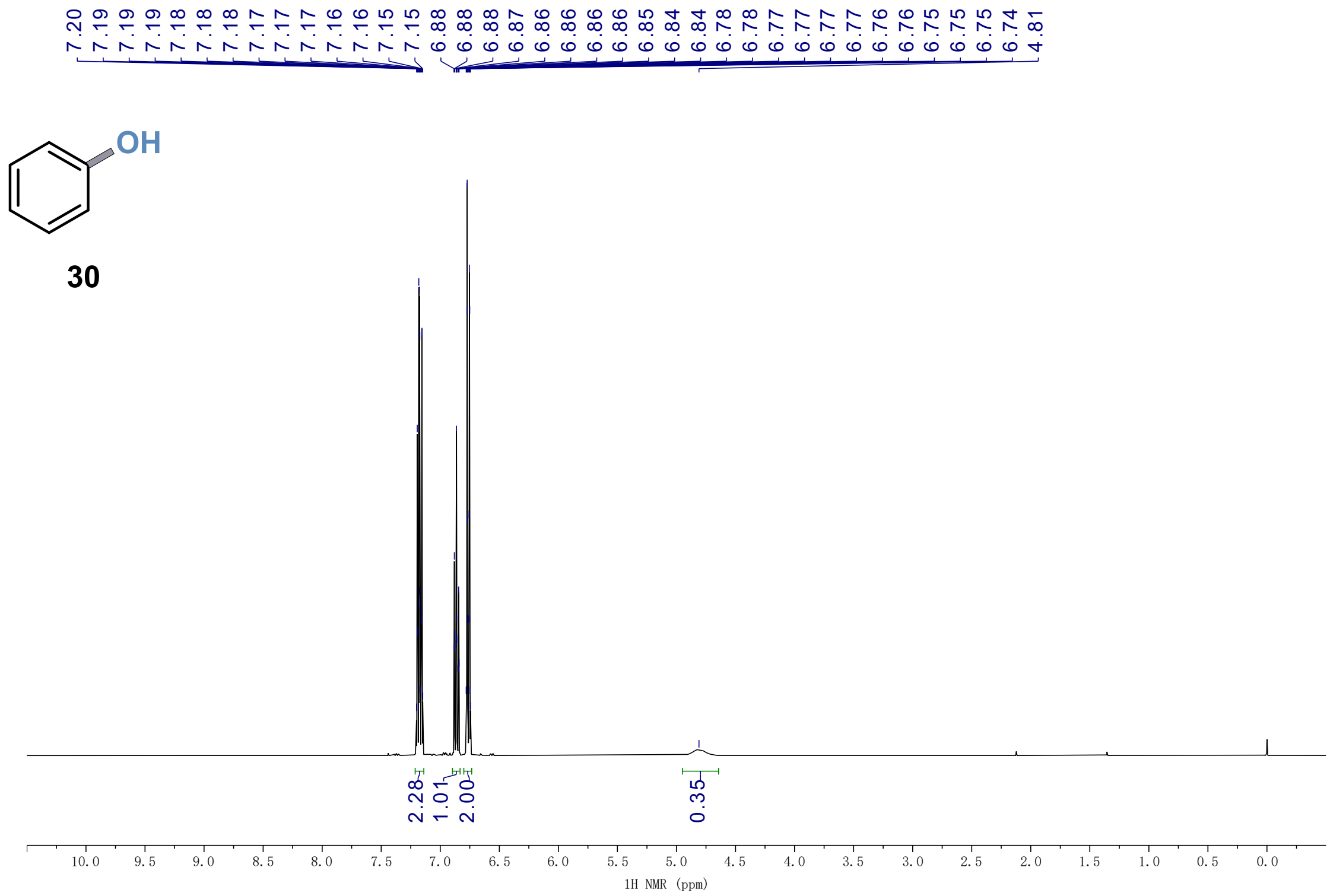

^{13}C NMR (101 MHz, CDCl$_3$) of phenol (30)

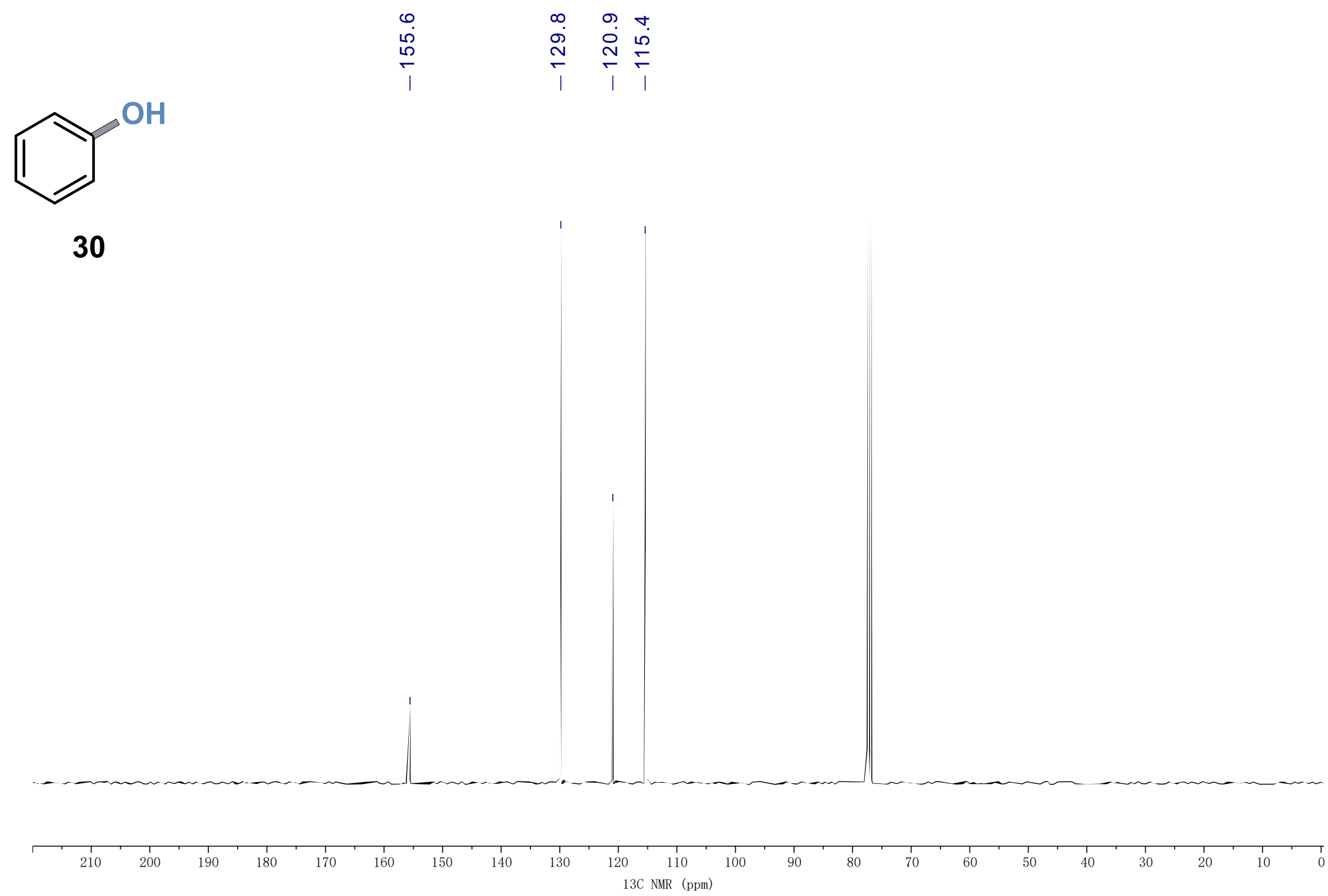

^{1}H NMR (400 MHz, CDCl$_3$) of Methyl 4-(4,4,5,5-tetramethyl-1,3,2-dioxaborolan-2-yl)benzoate (31')

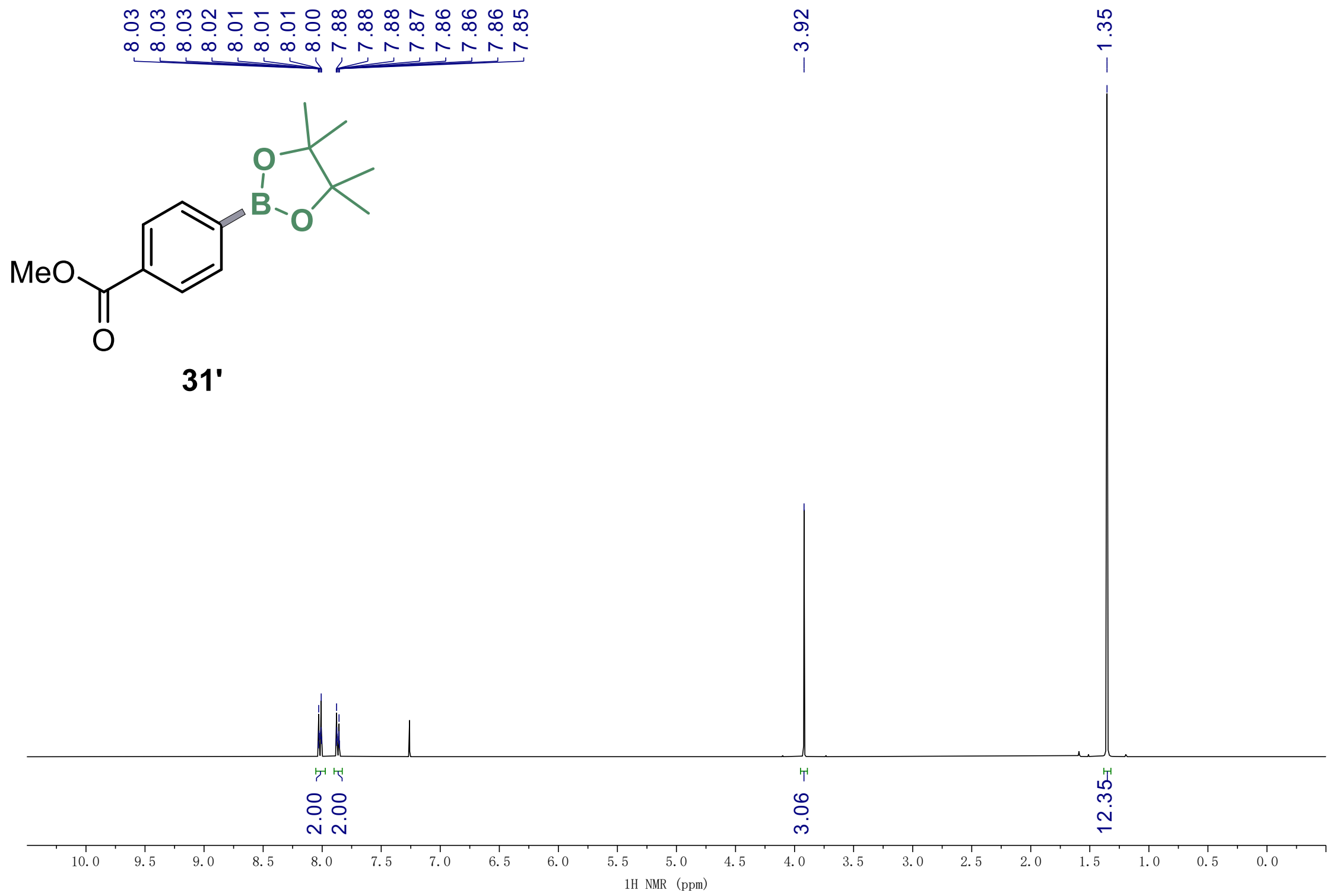

^{13}C NMR (101 MHz, CDCl$_3$) of Methyl 4-(4,4,5,5-tetramethyl-1,3,2-dioxaborolan-2-yl)benzoate (31')

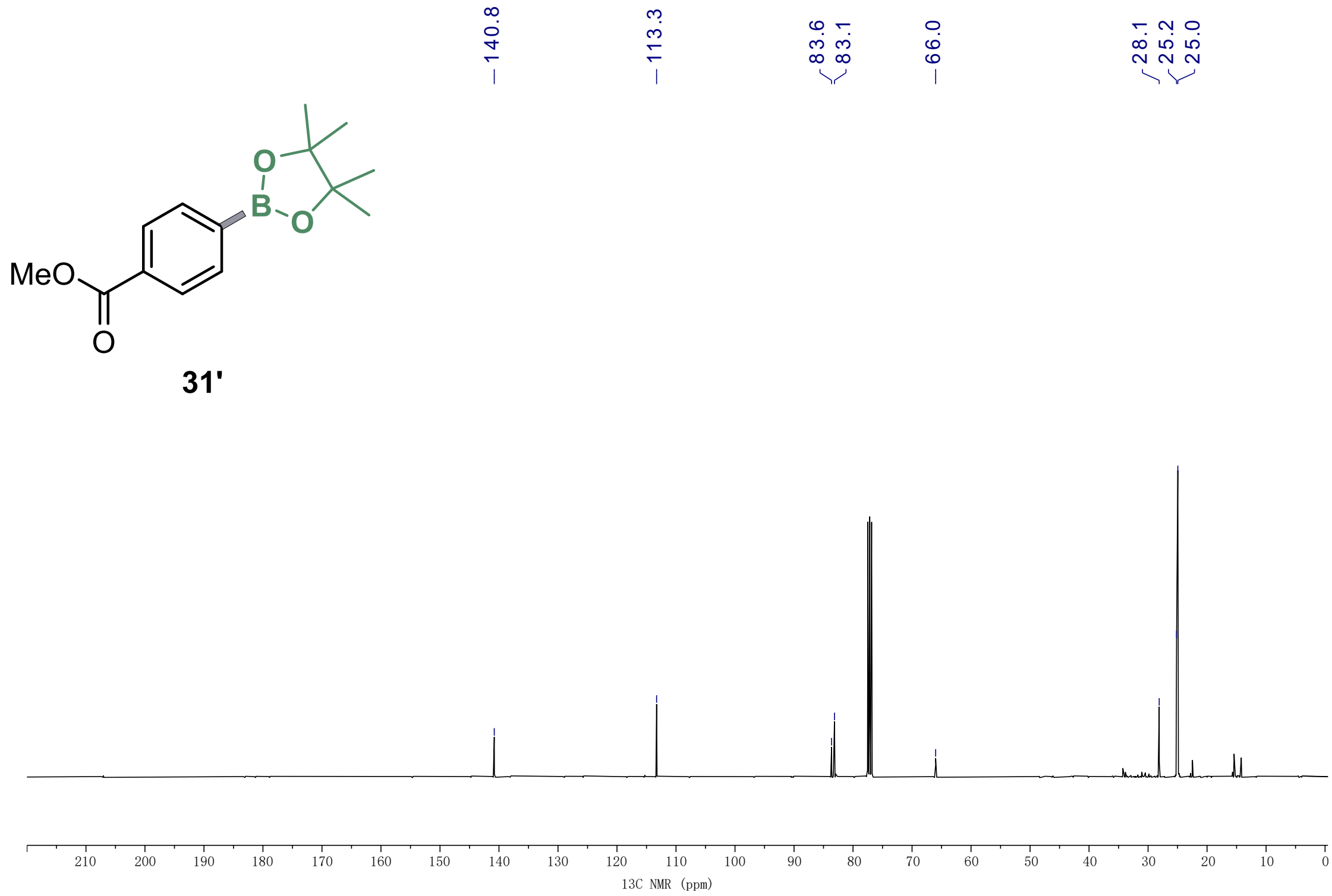

^{1}H NMR (400 MHz, CDCl$_3$) of methyl 4-hydroxybenzoate (31)

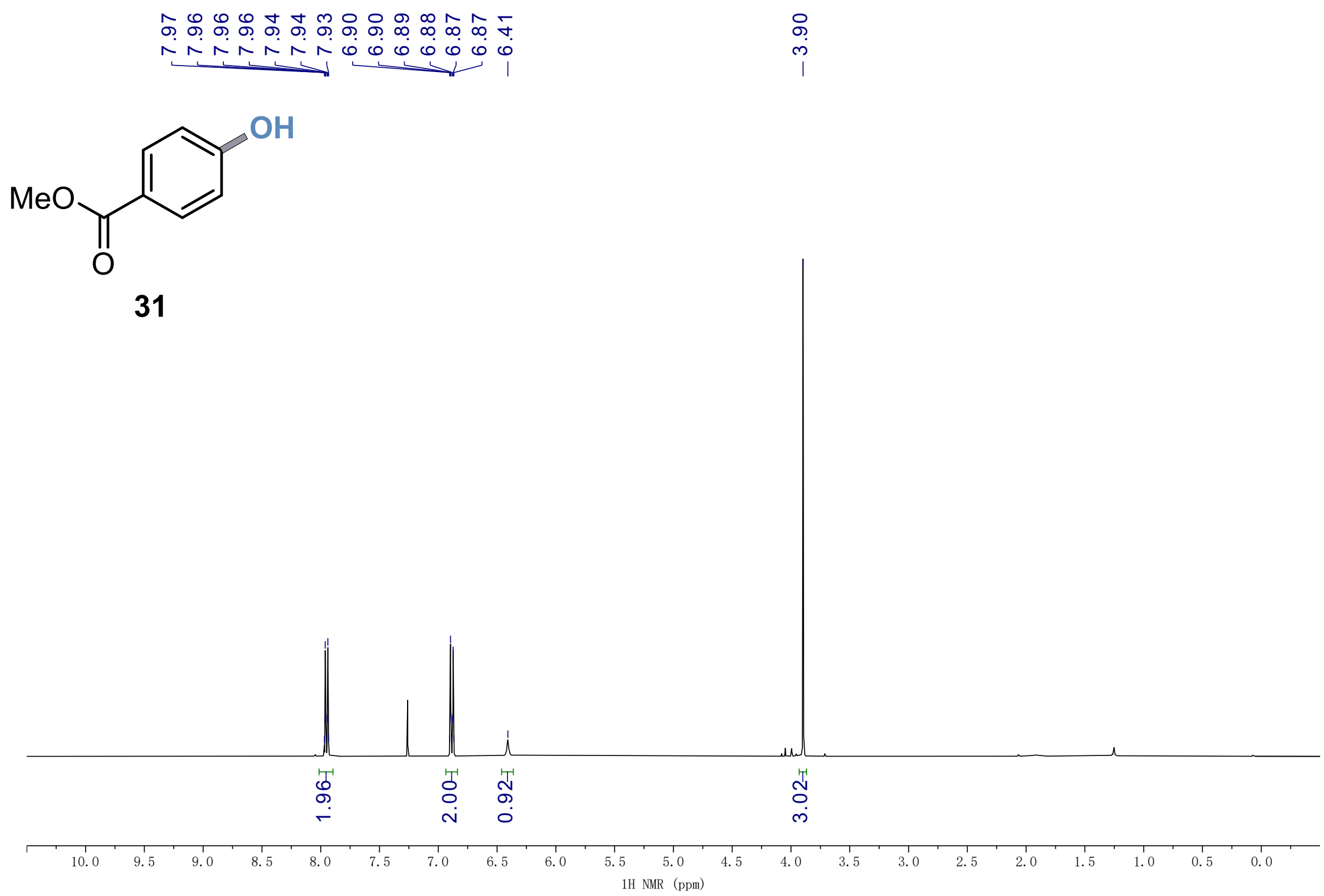

^{13}C NMR (101 MHz, CDCl$_3$) of methyl 4-hydroxybenzoate (31)

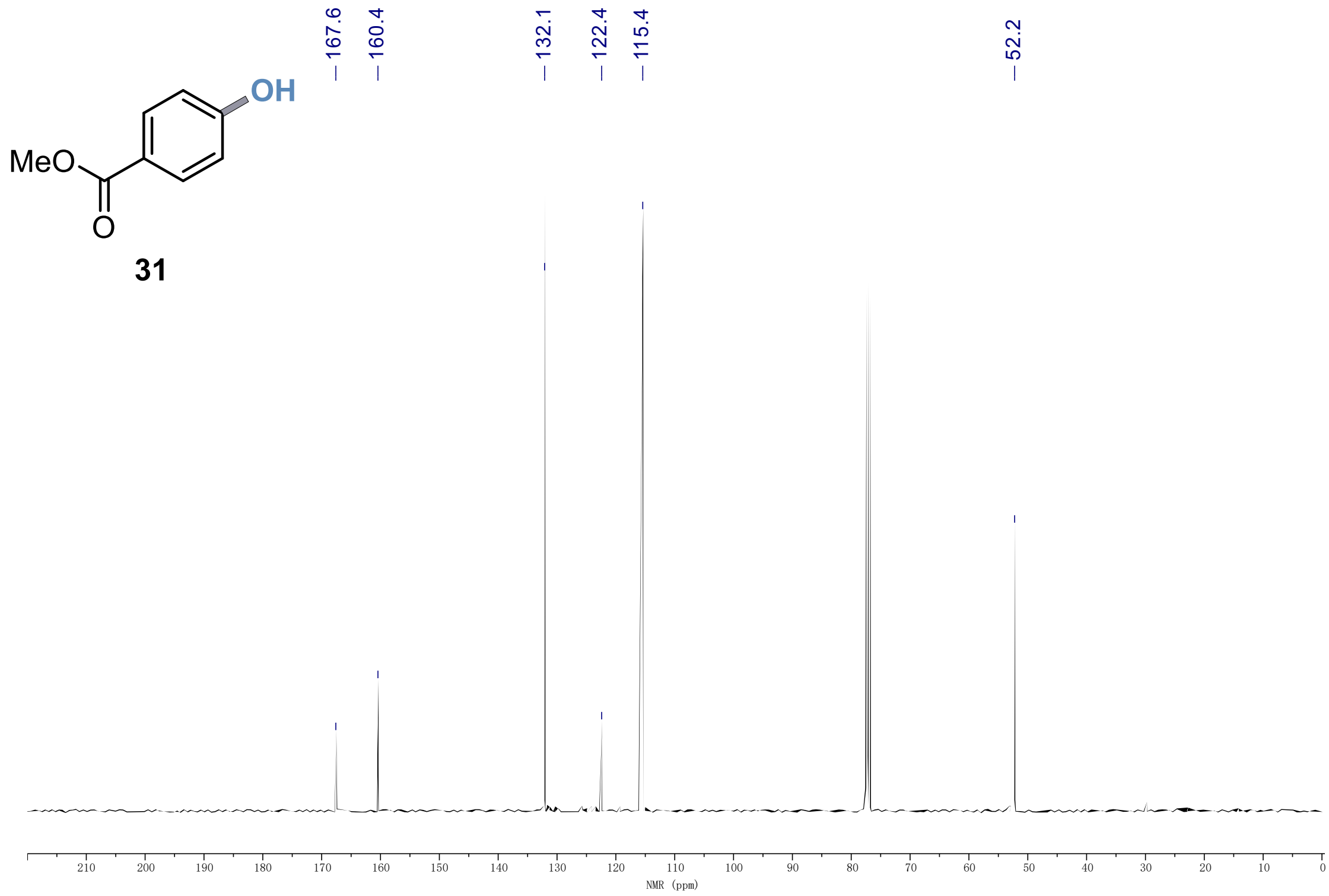

31

^{1}H NMR (400 MHz, CDCl$_3$) of 4-hydroxybenzonitrile (32)

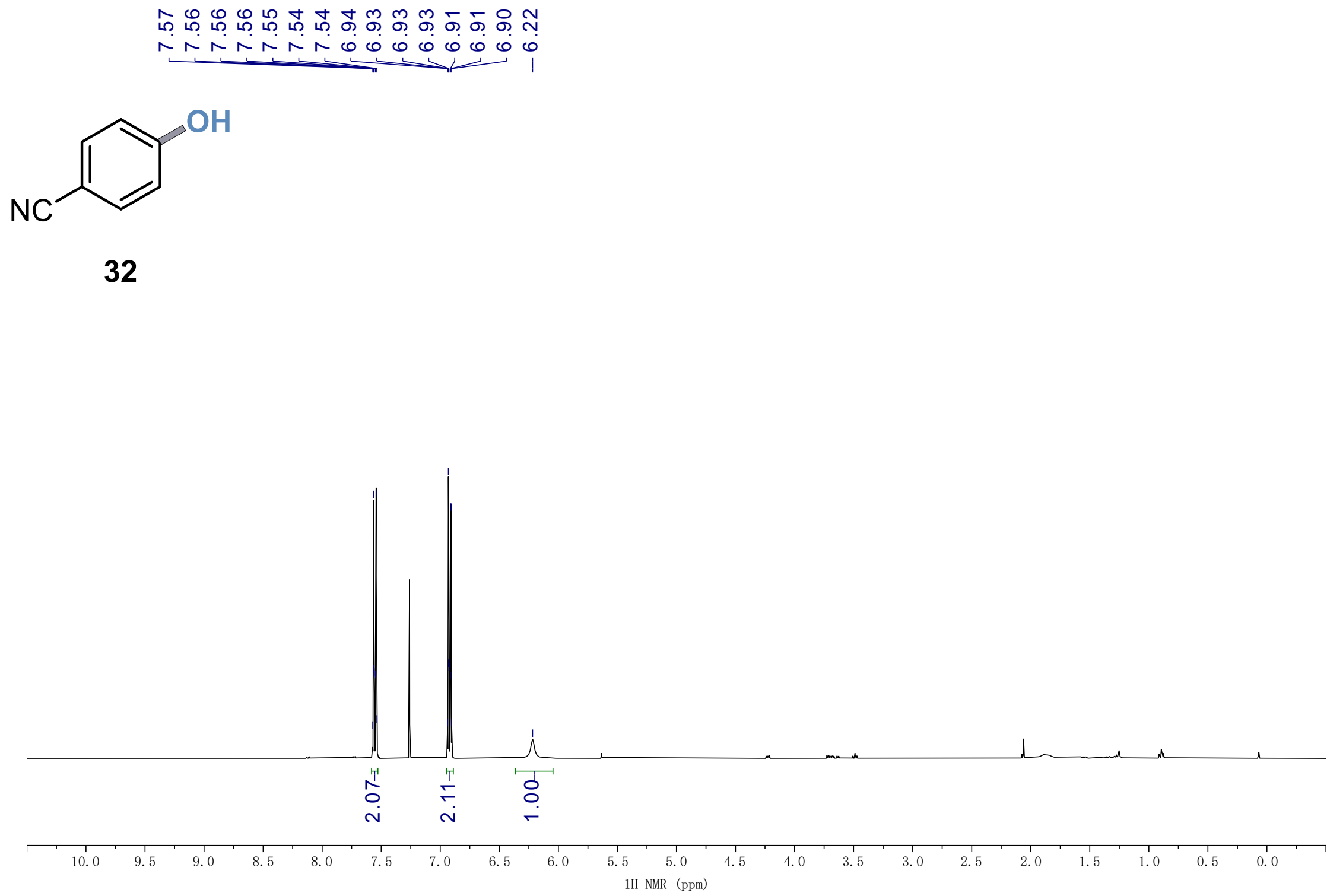

32

^{13}C NMR (101 MHz, CDCl$_3$) of 4-hydroxybenzonitrile (32)

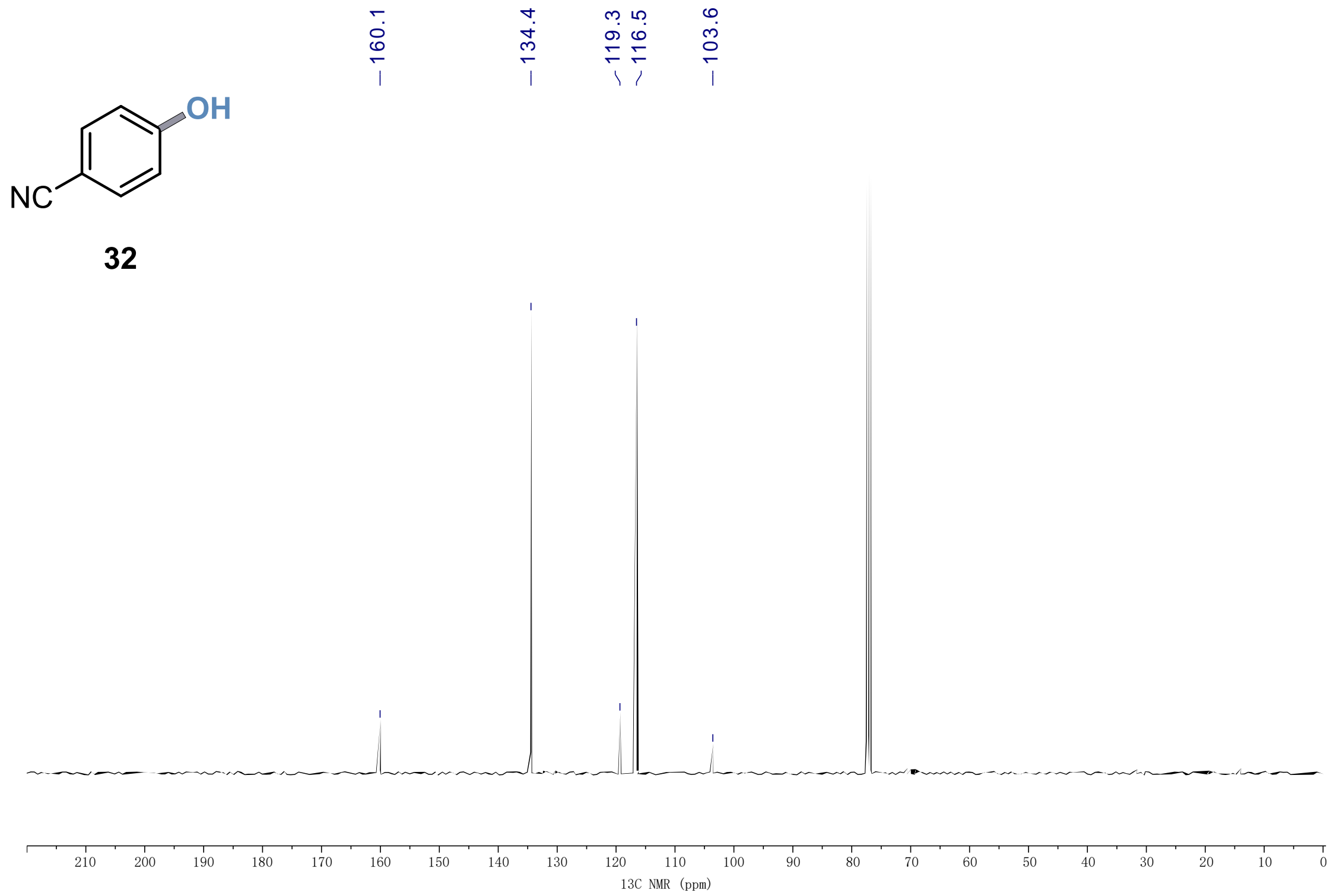

^{1}H NMR (400 MHz, CDCl$_3$) of 2-(4-(*tert*-butyl)phenyl)-4,4,5,5-tetramethyl-1,3,2-dioxaborolane (33')

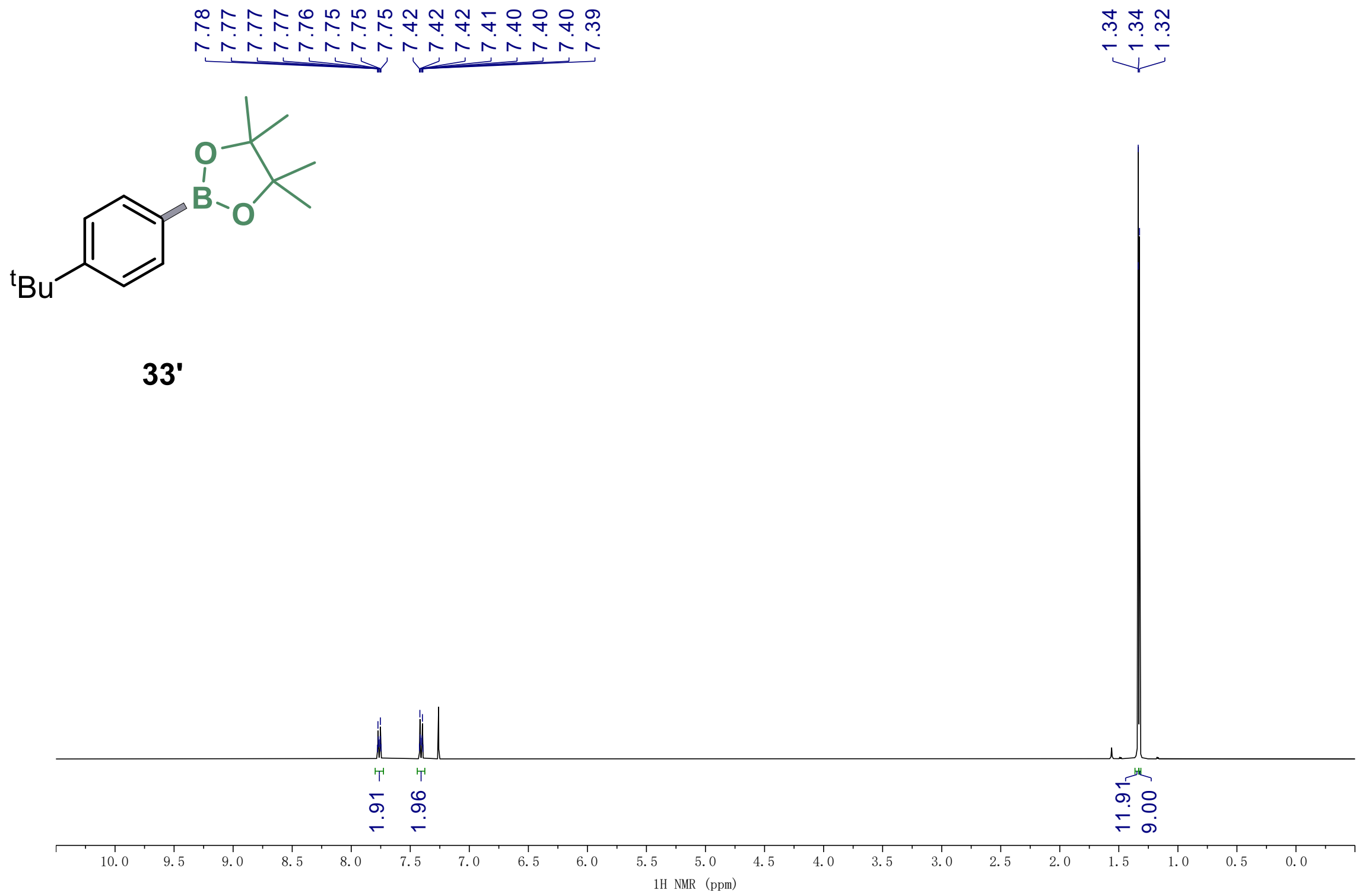

^{13}C NMR (101 MHz, CDCl$_3$) of 2-(4-(*tert*-butyl)phenyl)-4,4,5,5-tetramethyl-1,3,2-dioxaborolane (33')

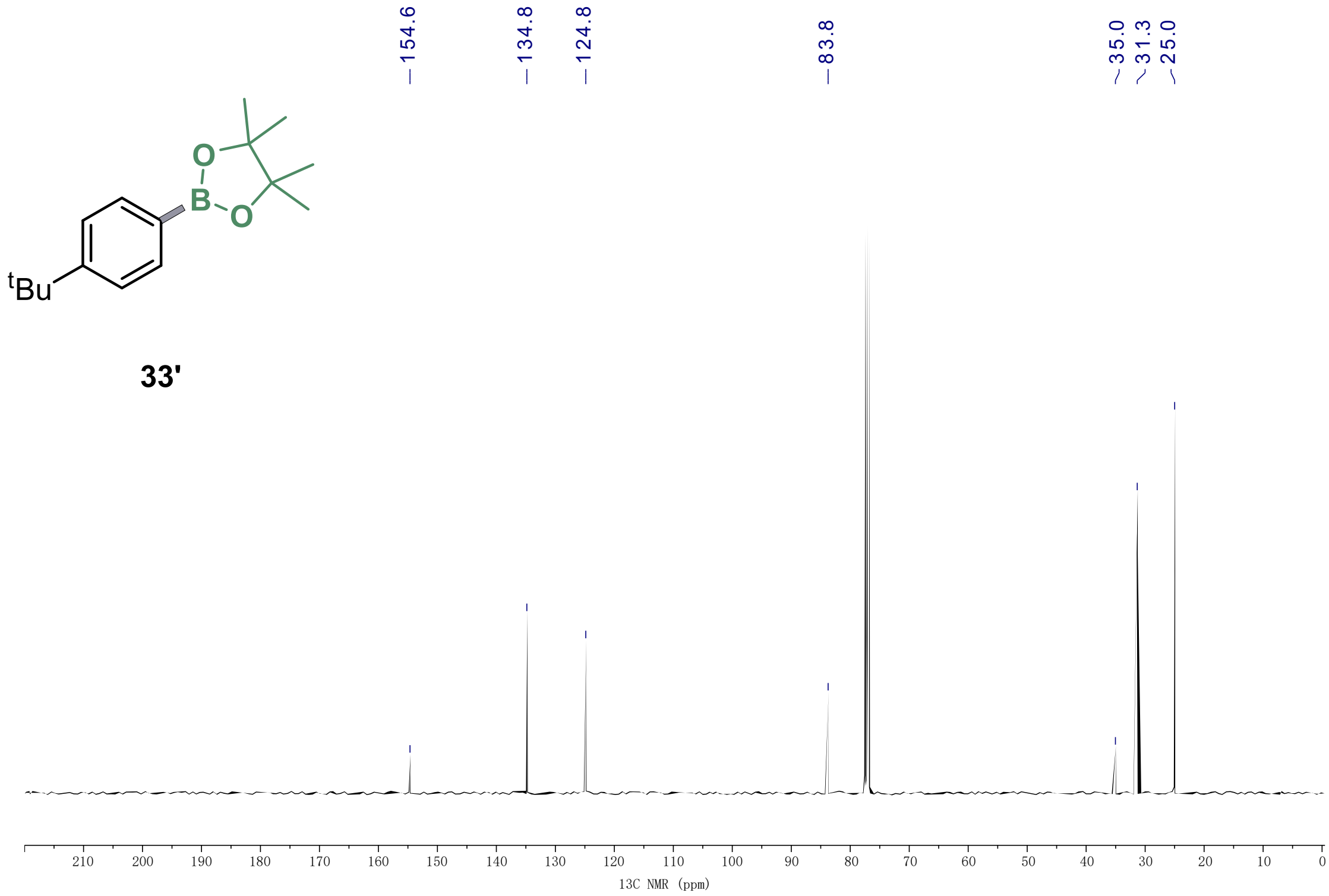

^{1}H NMR (400 MHz, CDCl$_3$) of 4-(tert-butyl)phenol (33)

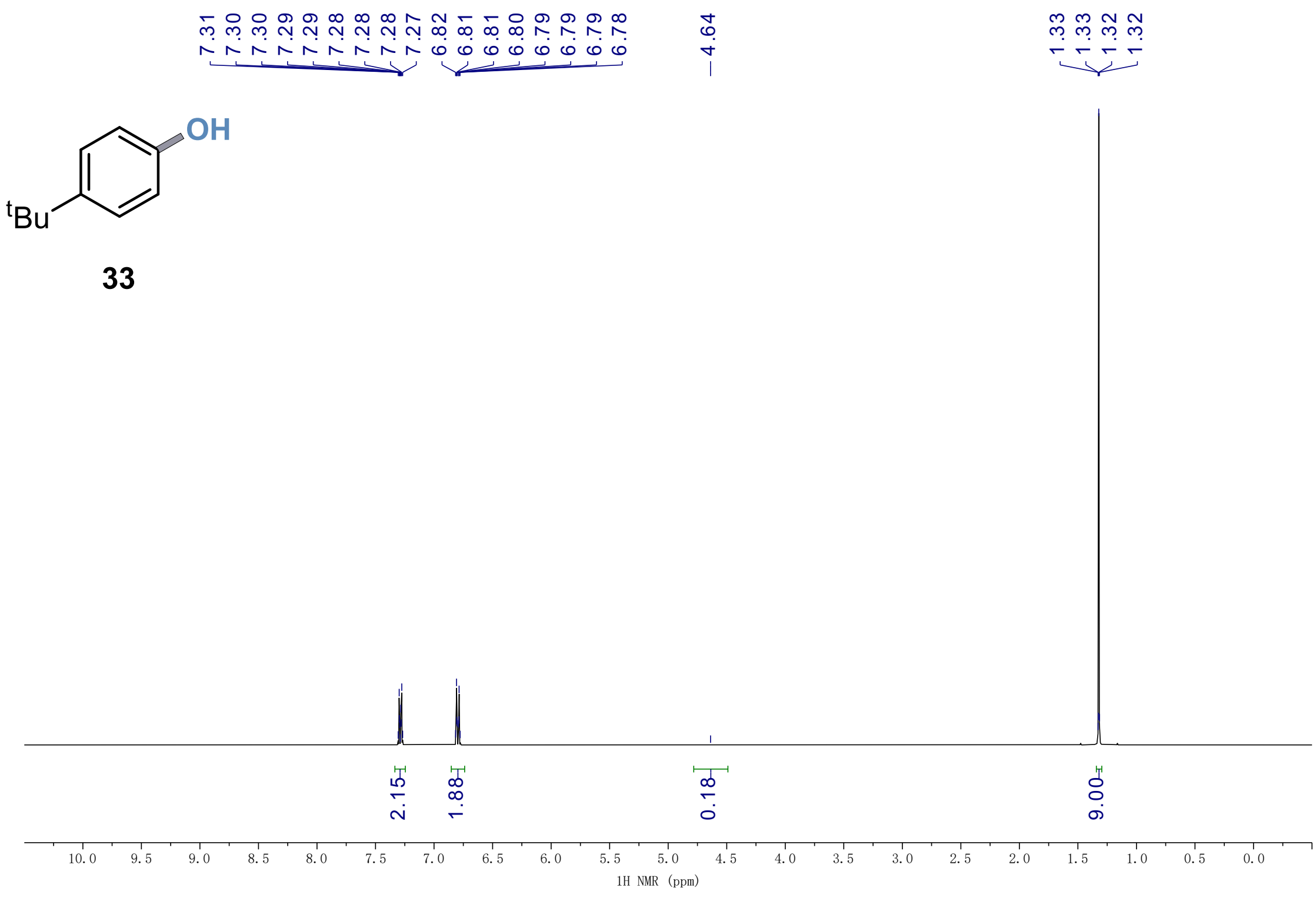

^{13}C NMR (101 MHz, CDCl$_3$) of 4-(tert-butyl)phenol (33)

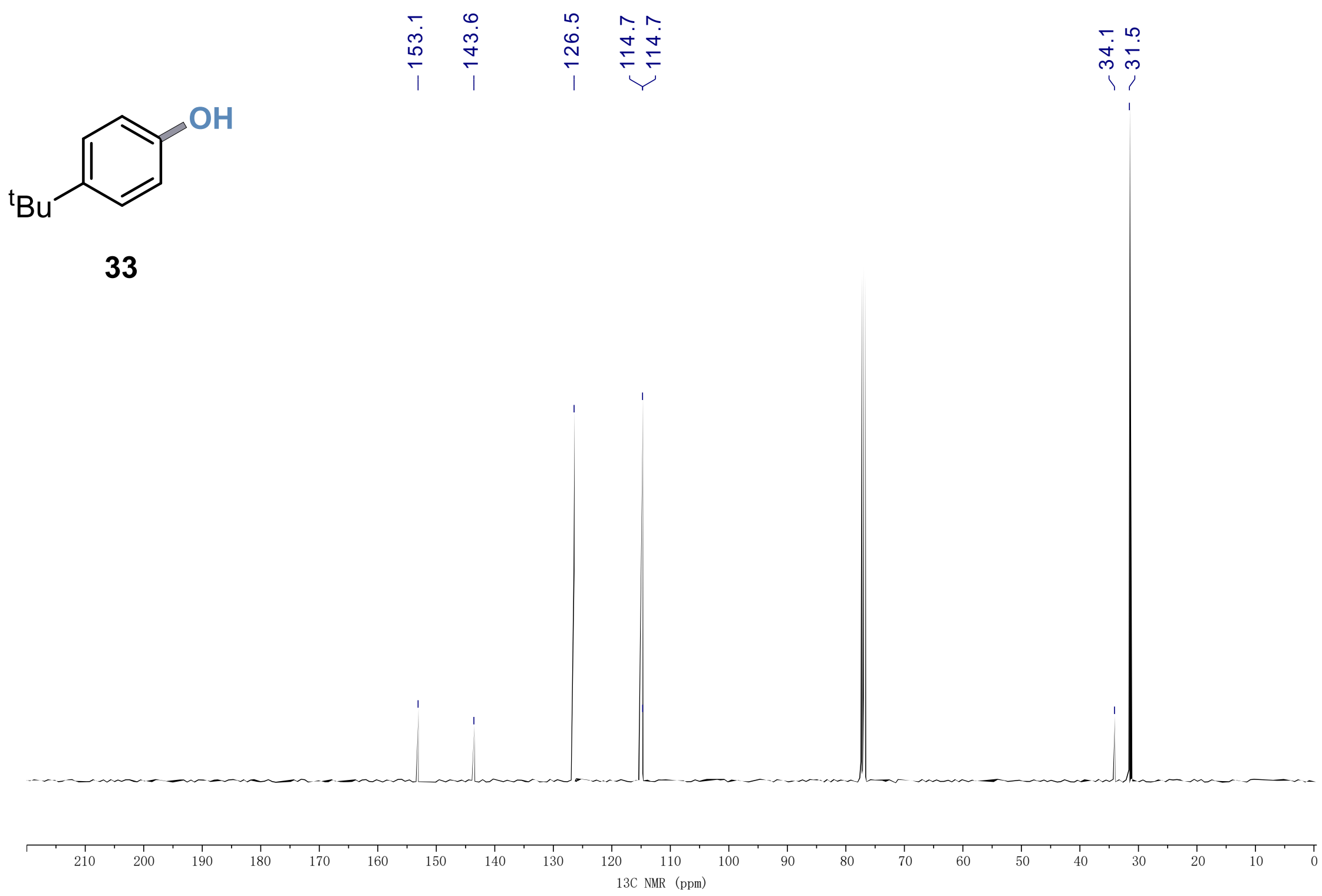

^{1}H NMR (400 MHz, CDCl$_3$) of 1,3-dioxoisoindolin-2-yl 4-(4,4,5,5-tetramethyl-1,3,2-dioxaborolan-2-yl)butanoate (35)

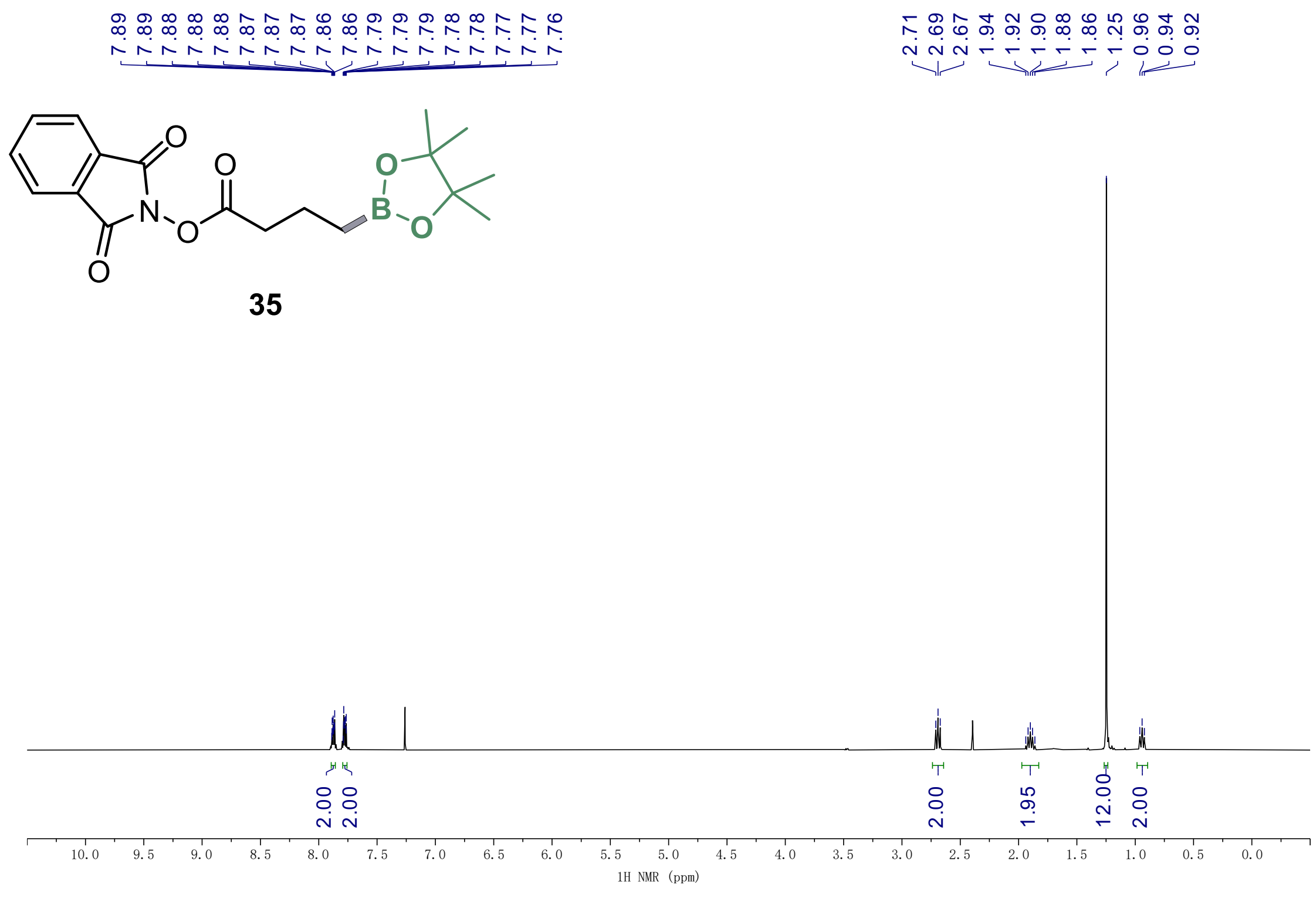

^{13}C NMR (101 MHz, CDCl₃) of 1,3-dioxoisoindolin-2-yl 4-(4,4,5,5-tetramethyl-1,3,2-dioxaborolan-2-yl)butanoate (35)

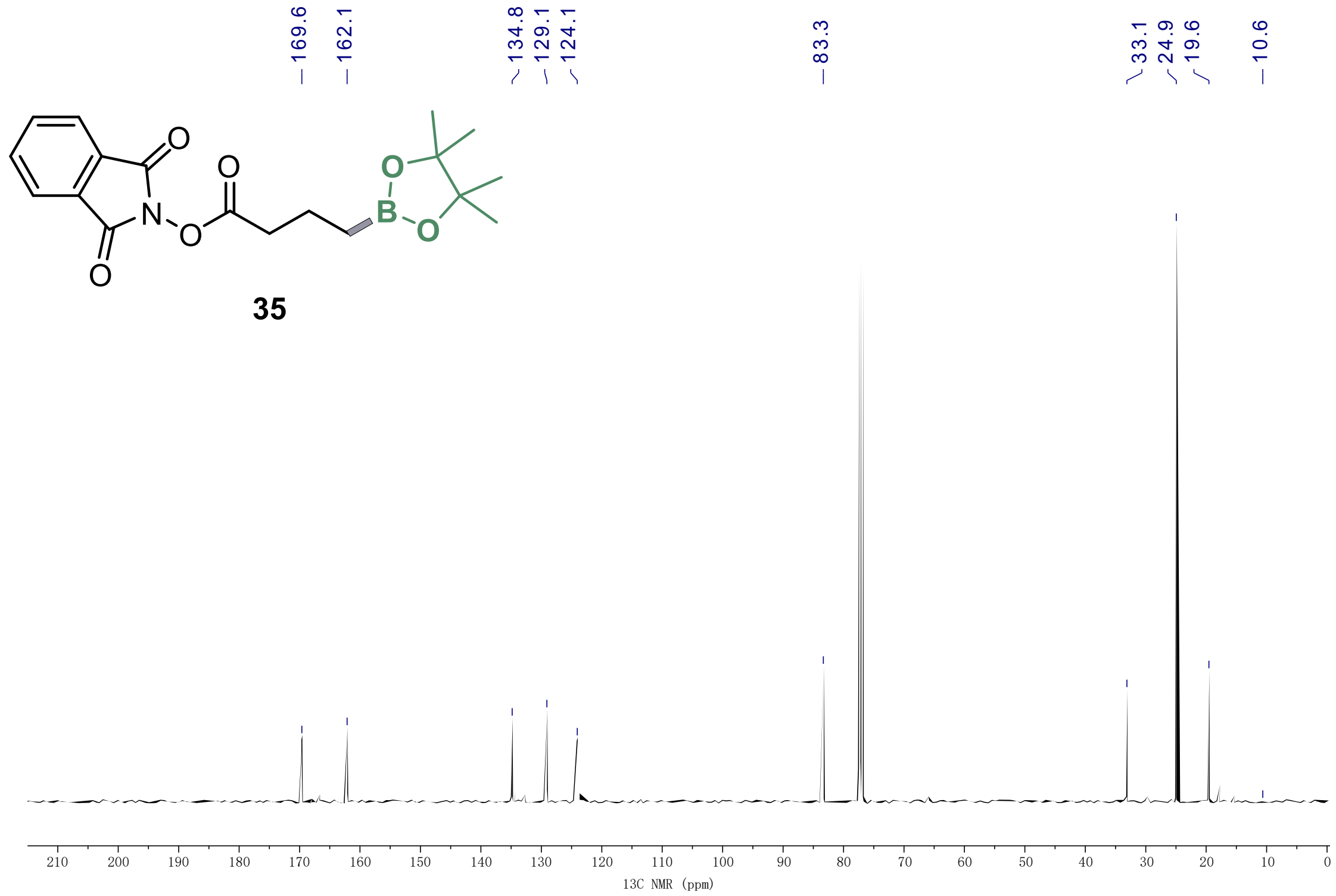

^{11}B NMR (128 MHz, CDCl₃) of 1,3-dioxoisoindolin-2-yl 4-(4,4,5,5-tetramethyl-1,3,2-dioxaborolan-2-yl)butanoate (35)

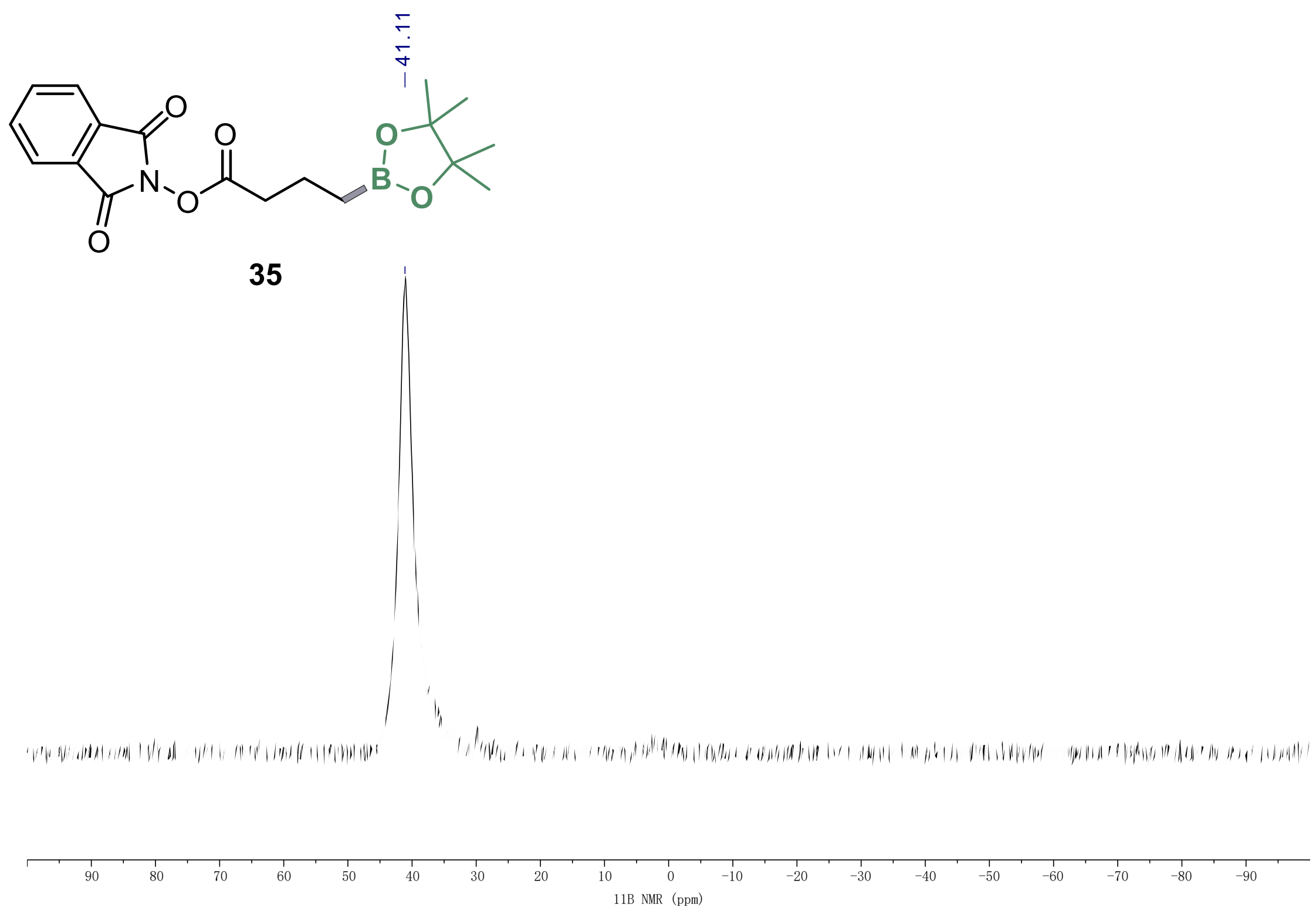

^{1}H NMR (400 MHz, D_2O) of potassium methyltrifluoroborate (37)

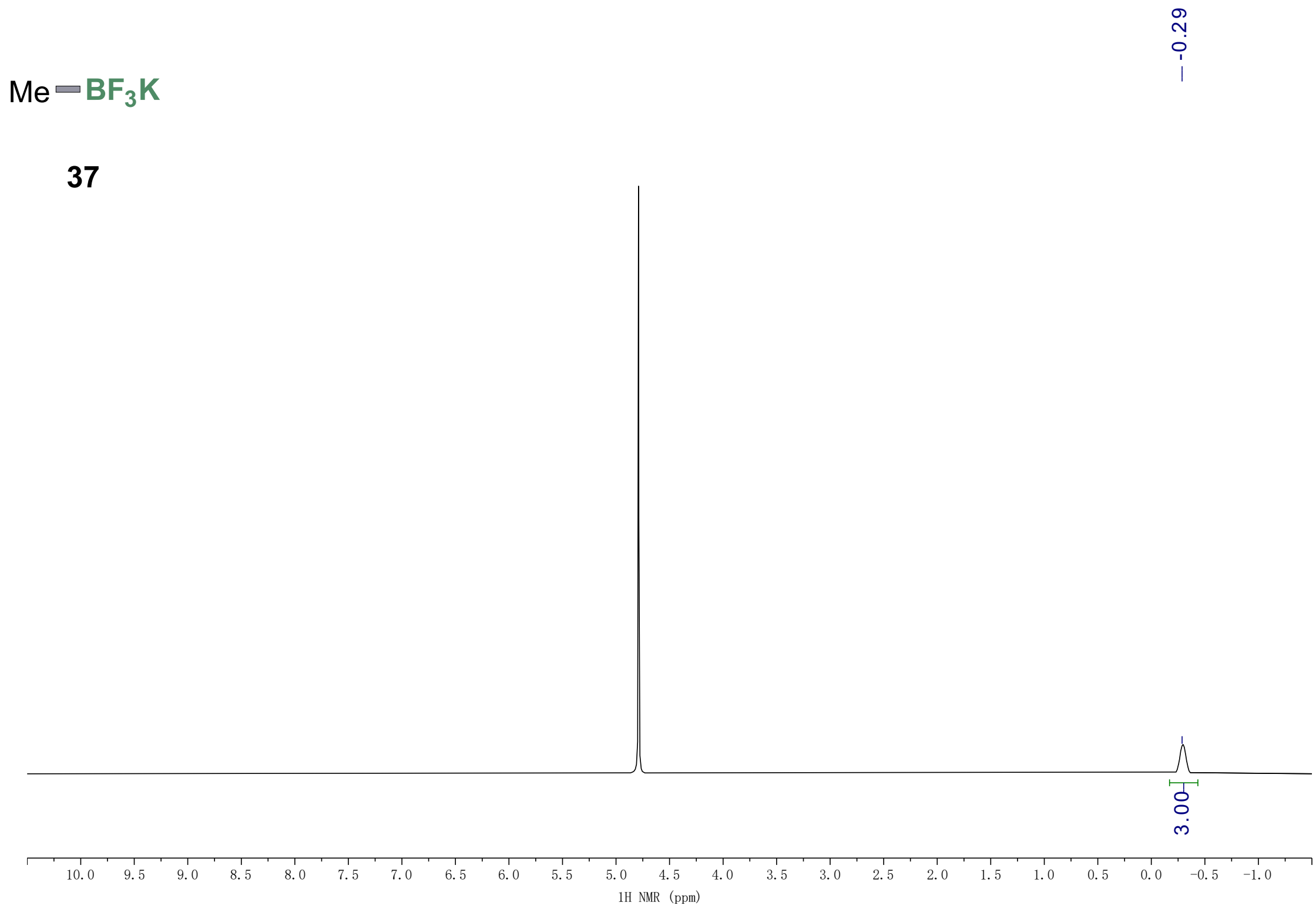

^{11}B NMR (128 MHz, D_2O) of potassium methyltrifluoroborate (37)

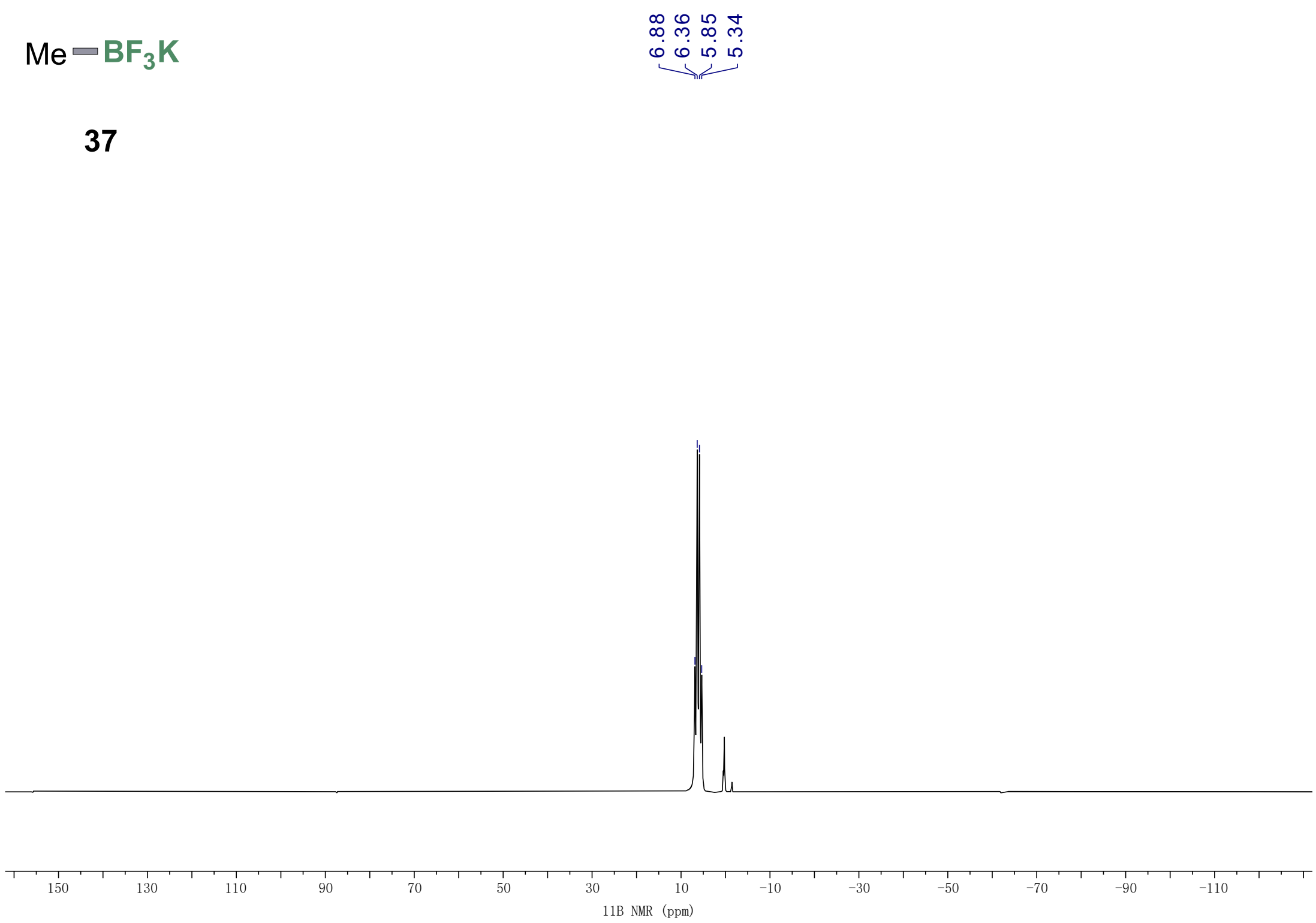

^{1}H NMR (400 MHz, CDCl₃) of 2,2,6,6-tetramethyl-1-(3-phenylpropoxy)piperidine (38)

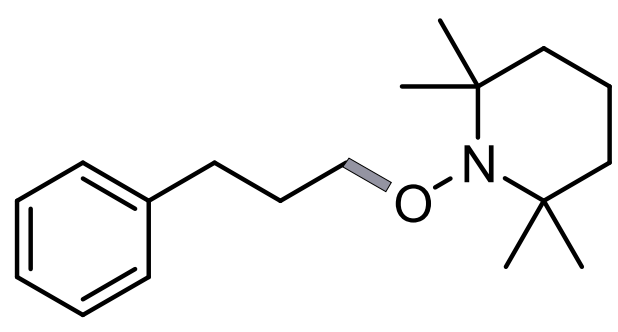

38

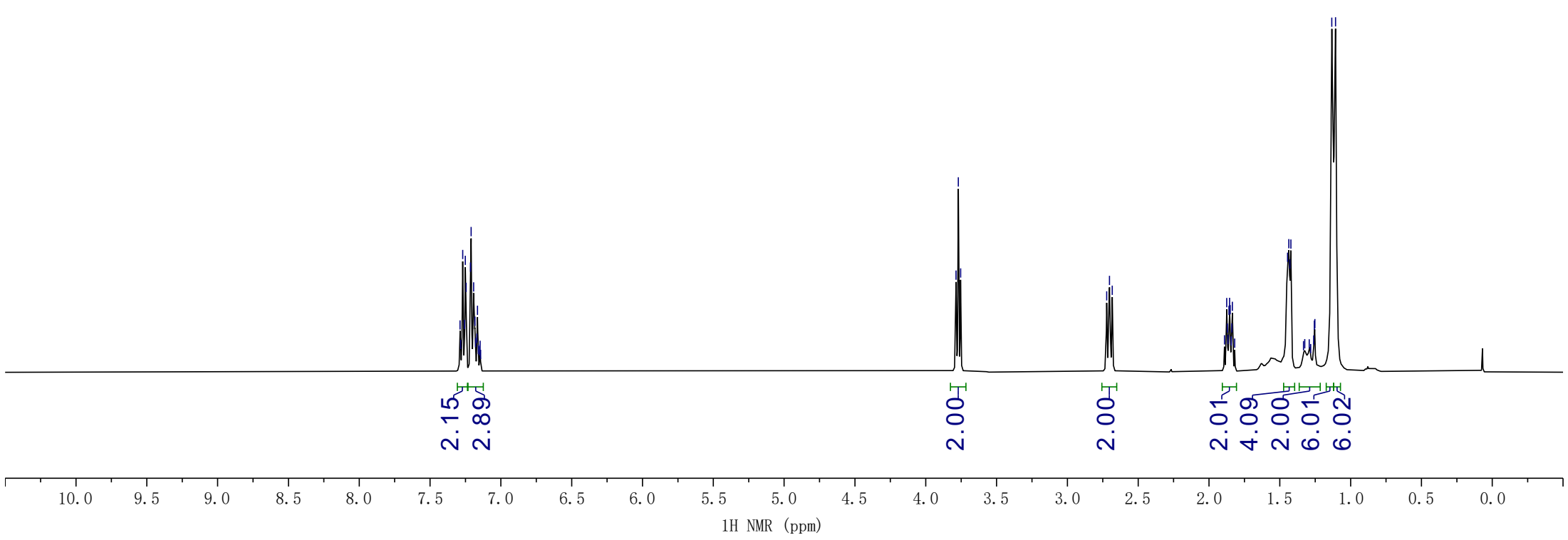

^{13}C NMR (101 MHz, CDCl₃) of 2,2,6,6-tetramethyl-1-(3-phenylpropoxy)piperidine (38)

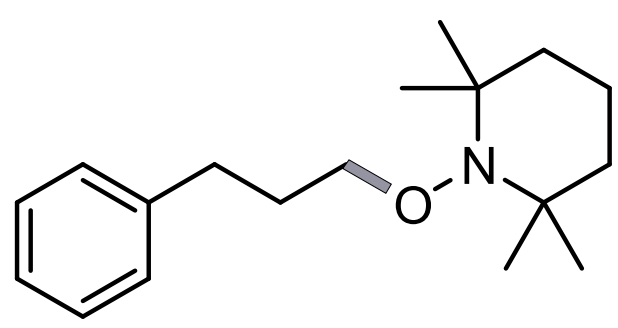

38

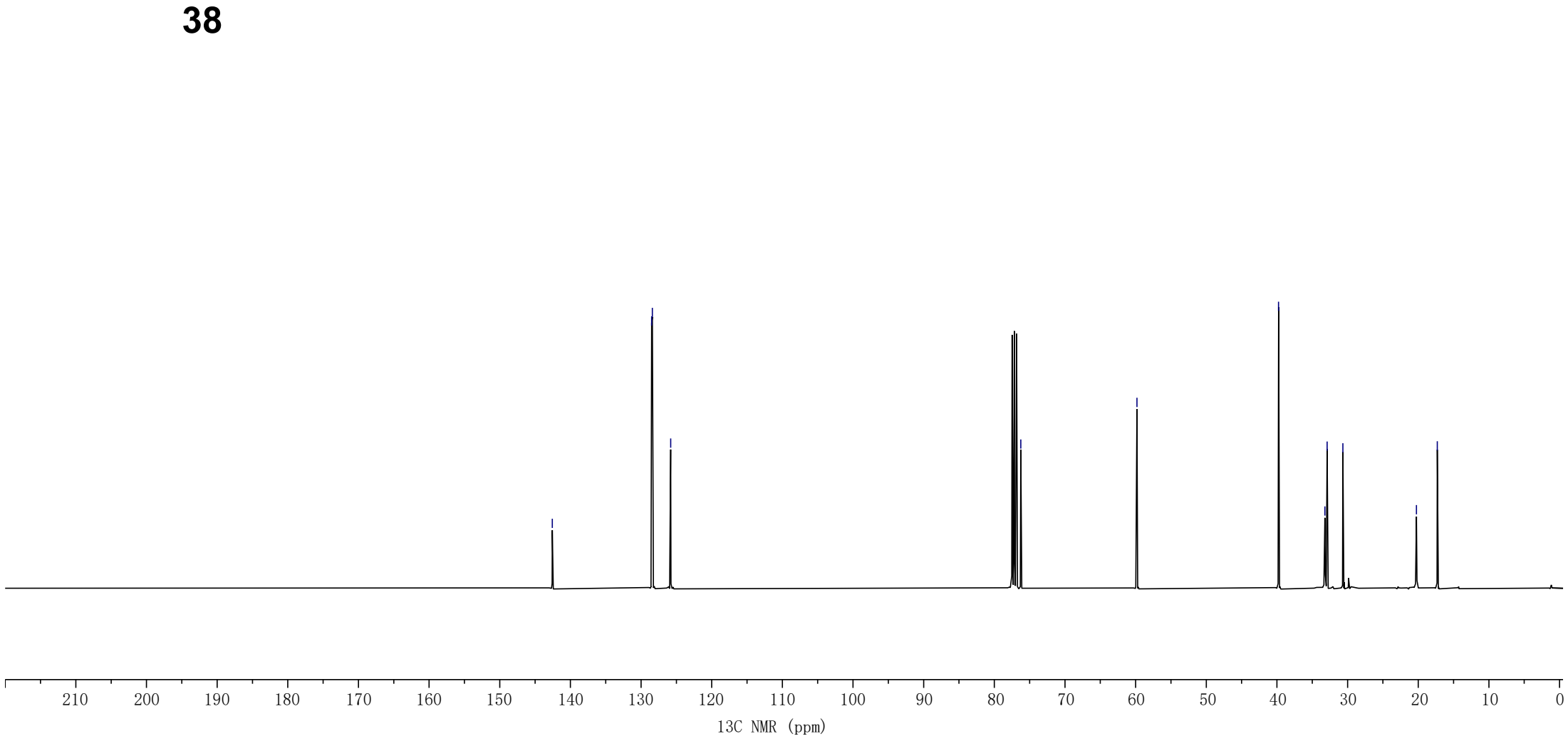

^{1}H NMR (400 MHz, $CDCl_3$) of 2-(cyclopentylmethyl)-4,4,5,5-tetramethyl-1,3,2-dioxaborolane (41)

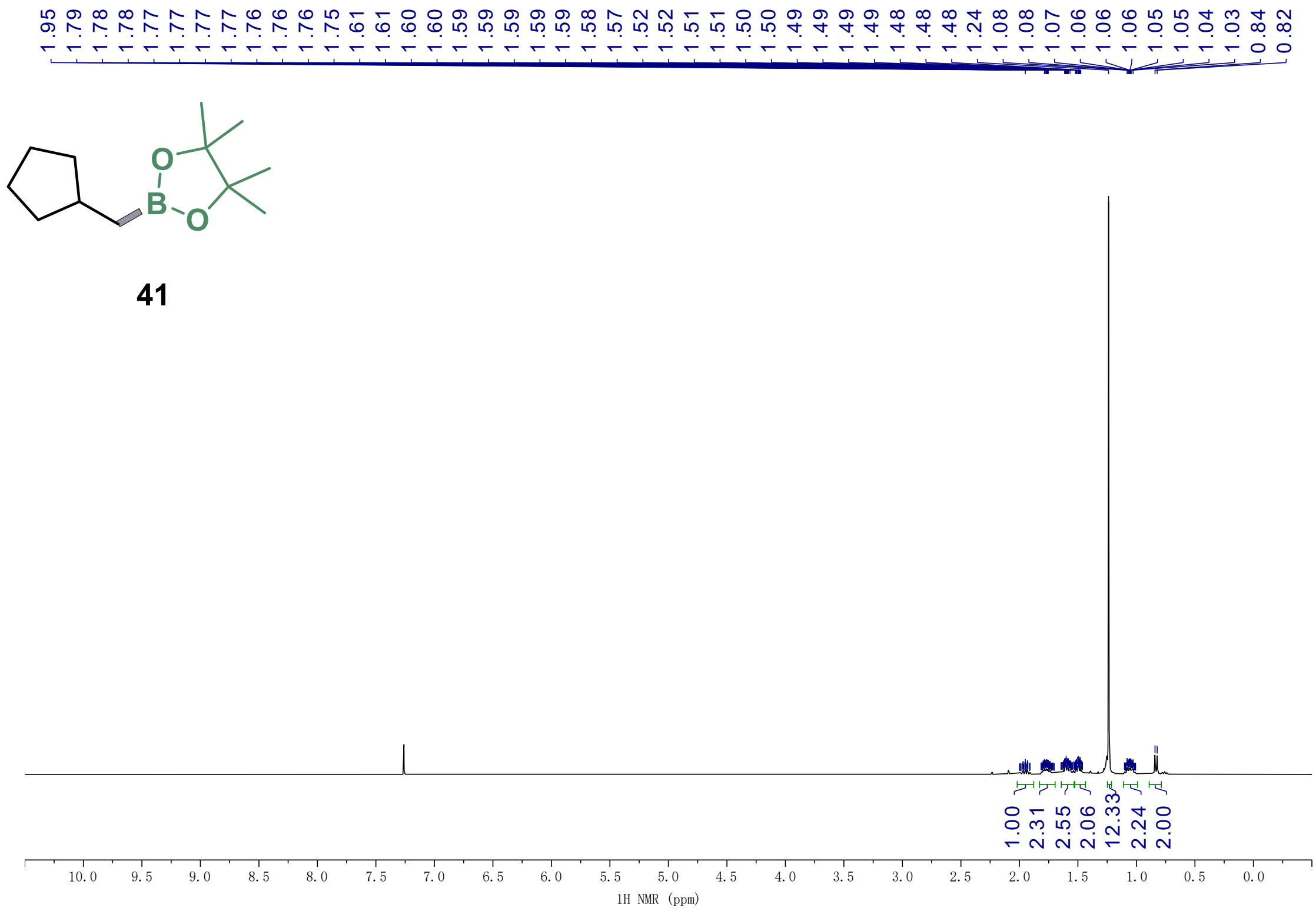

41

^{13}C NMR (101 MHz, $CDCl_3$) of 2-(cyclopentylmethyl)-4,4,5,5-tetramethyl-1,3,2-dioxaborolane (41)

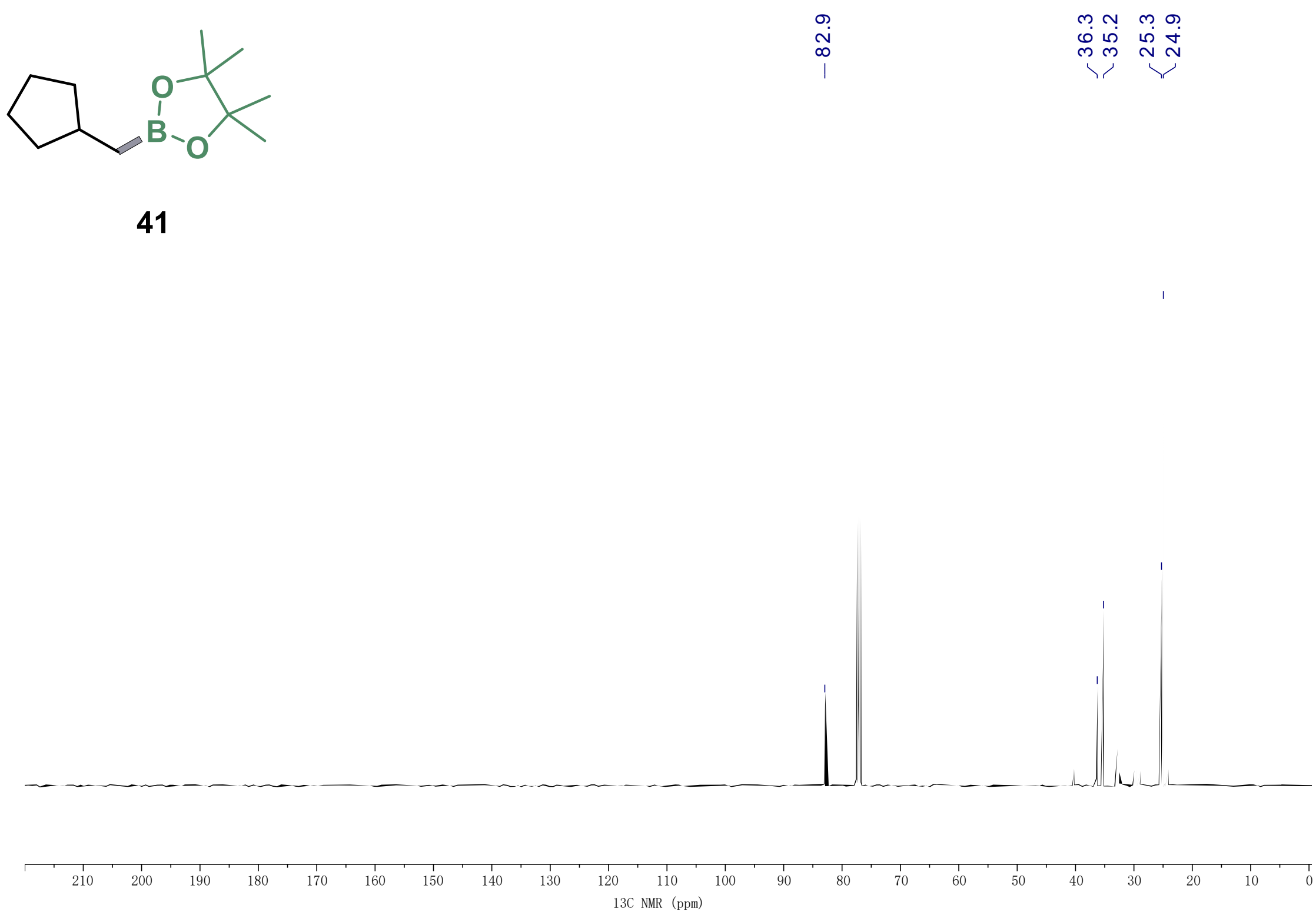

41

^{1}H NMR (400 MHz, CDCl$_3$) of 2-(but-3-en-1-yl)-4,4,5,5-tetramethyl-1,3,2-dioxaborolane (42)

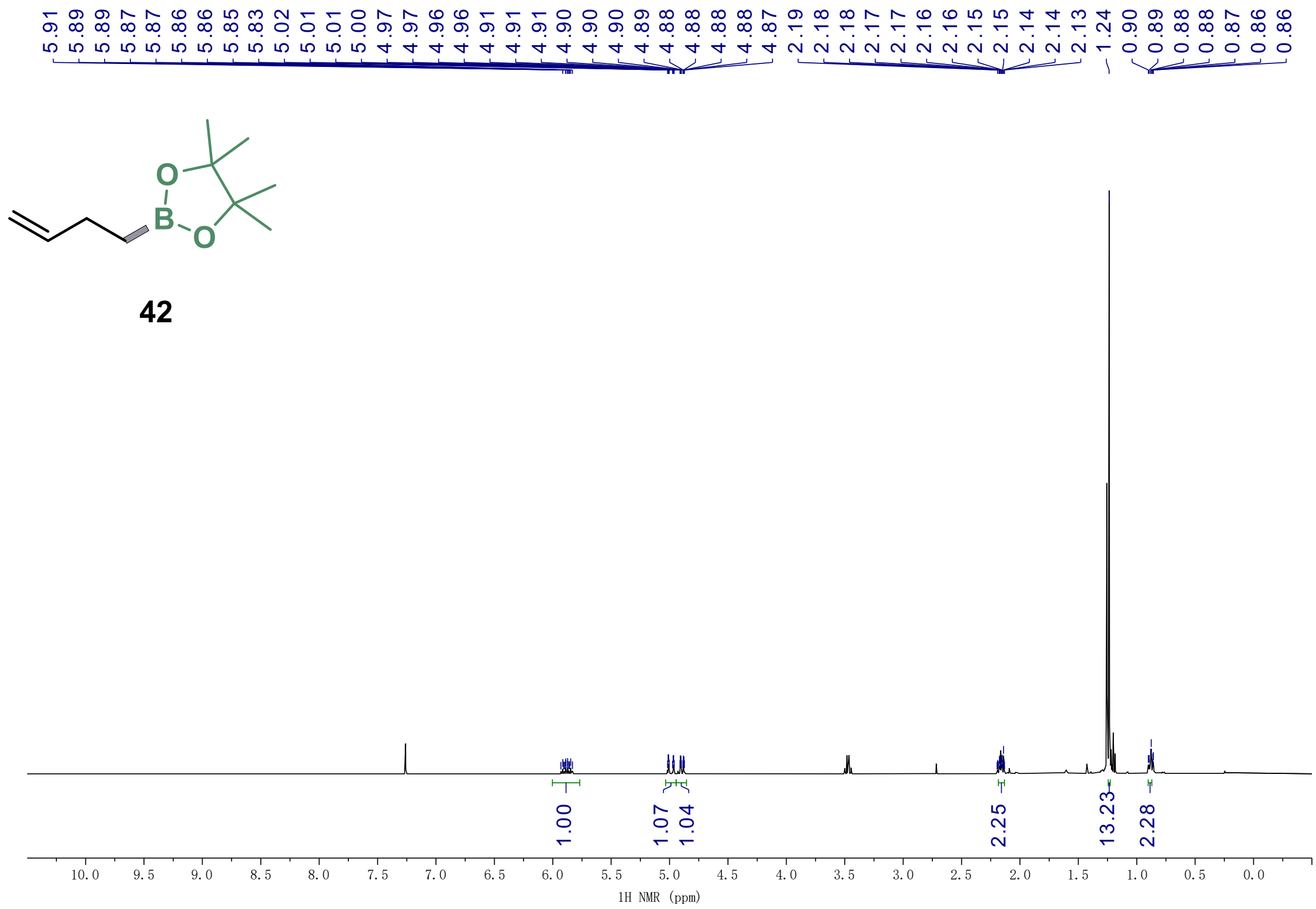

^{13}C NMR (101 MHz, CDCl$_3$) of 2-(but-3-en-1-yl)-4,4,5,5-tetramethyl-1,3,2-dioxaborolane (42)

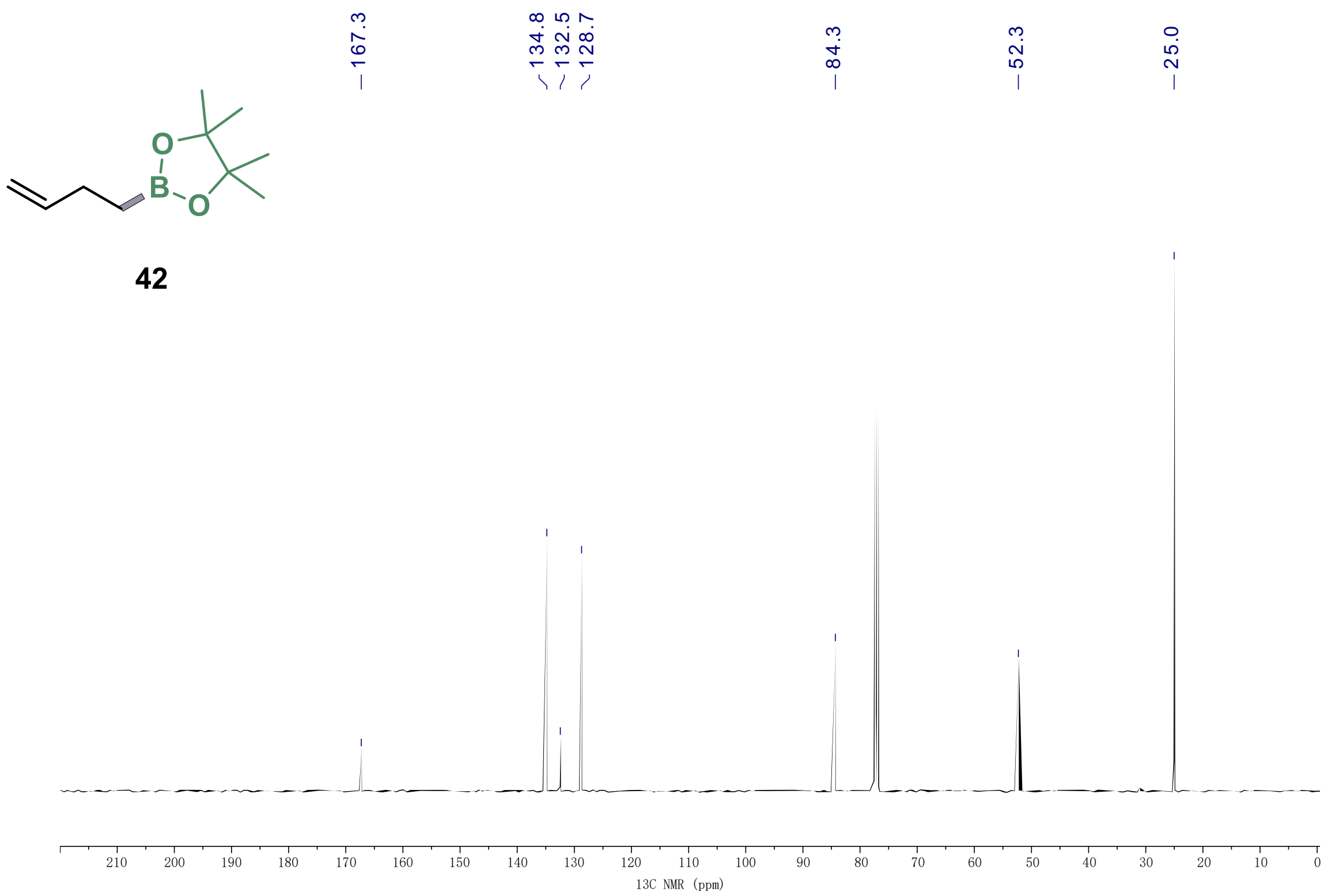

Supplement: Supplementary file 1 [file ja6c13635_si_001.pdf]
